# An Auxiliary Approach for the Stereoselective Synthesis of Topologically Chiral Catenanes

**DOI:** 10.1016/j.chempr.2019.03.008

**Published:** 2019-06-13

**Authors:** Mathieu Denis, James E.M. Lewis, Florian Modicom, Stephen M. Goldup

**Affiliations:** 1 School of Chemistry, University of Southampton, Highfield, Southampton SO17 1BJ, UK; 2 Department of Chemistry, Imperial College London, Molecular Sciences Research Hub, 80 Wood Lane, London W12 0BZ, UK

**Keywords:** SDG9: Industry, innovation, and infrastructure, topology, chirality, catenanes, stereoselective, mechanical bond

## Abstract

Catenanes, molecules in which two rings are threaded through one another like links in a chain, can form as two structures related like an object and its mirror image but otherwise identical if the individual rings lack bilateral symmetry. These structures are described as “topologically chiral” because, unlike most chiral molecules, it is not possible to convert one mirror-image form to the other under the rules of mathematical topology. Although intriguing and discussed as early as 1961, to date all methods of accessing molecules containing only this topological stereogenic element require the separation of the mirror-image forms via chiral stationary phase high-performance liquid chromatography, which has limited their investigation to date. Here, we present a simple method that uses a readily available source of chiral information to allow the stereoselective synthesis of topologically chiral catenanes.

## Highlights

• First stereoselective synthesis of a topologically chiral catenane
• First absolute stereochemical assignment of a topologically chiral catenane
• First example of an auxiliary approach to topologically chiral catenanes

## The Bigger Picture

Chiral molecules have occupied a special place in chemistry since Pasteur reported the painstaking separation of mirror-image crystals of tartaric acid salts in 1848. In the 21^st^ century, chiral molecules remain a major scientific focus because of their importance in biology and their emerging applications in materials science. However, topologically chiral molecules, such as the catenanes described here, have received little attention because they are hard to make; preparative chiral stationary phase high-performance liquid chromatography allows the separation of their mirror-image forms but only on a very small scale. Here, we demonstrate the synthesis of topologically chiral catenanes by using standard synthetic techniques, marking their transition from “inaccessible curiosities” to valid synthetic targets for investigation in catalysis, sensing, medicinal chemistry, and materials science. Furthermore, this work will inspire efforts to access other neglected classes of chiral interlocked molecules.

## Graphical Abstract

**Figure:**
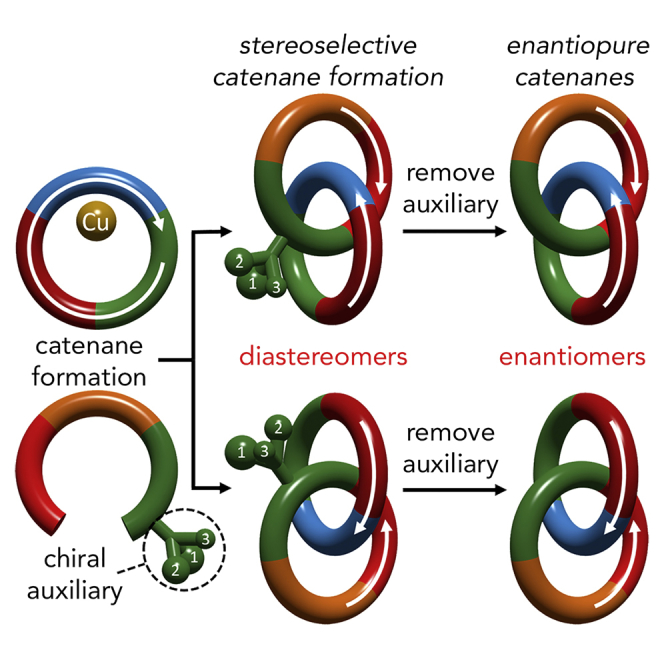

## Teaser

Catenanes, molecules comprising two rings held together like links in a chain, can exist as two mirror-image forms if the rings lack bilateral symmetry. These “topological enantiomers” are unusual because they cannot be interconverted by stretching or bending chemical bonds. To date, their synthesis has required the separation of mirror-image structures by specialist techniques. We present a simple method to allow the synthesis of topologically chiral catenanes, opening them up to investigation in catalysis, sensing, and materials science.

## Introduction

Chiral molecules occupy a special place in synthetic chemistry because of their ubiquity in biological systems and emerging applications in materials science.^1^ A tetrahedral carbon atom bearing four different substituents is the archetypal unit that can give rise to molecular chirality.^2^, ^3^, ^4^ However, chirality in organic molecules can arise because of a number of different covalent structural features in addition to such stereogenic centers, the most common examples of which are in molecules where atoms are arranged suitably around a fixed axis (commonly referred to as “axially chiral”) such that they facially desymmetrize an oriented plane (“planar chiral”) or are displayed in a helical arrangement (“helically chiral”).^5^, ^6^ Regardless of the structural origin of molecular chirality, the key challenge in the synthesis of chiral molecules is the production of pure samples of one mirror-image form (enantiomer) of the product; because the different enantiomers of a chiral molecule by definition have identical properties under most circumstances, they must either be produced selectively or separated by specialist techniques. Thus, a significant amount of effort has been devoted to achieving these goals efficiently over the past century of synthetic chemistry research.

**Figure 1:**
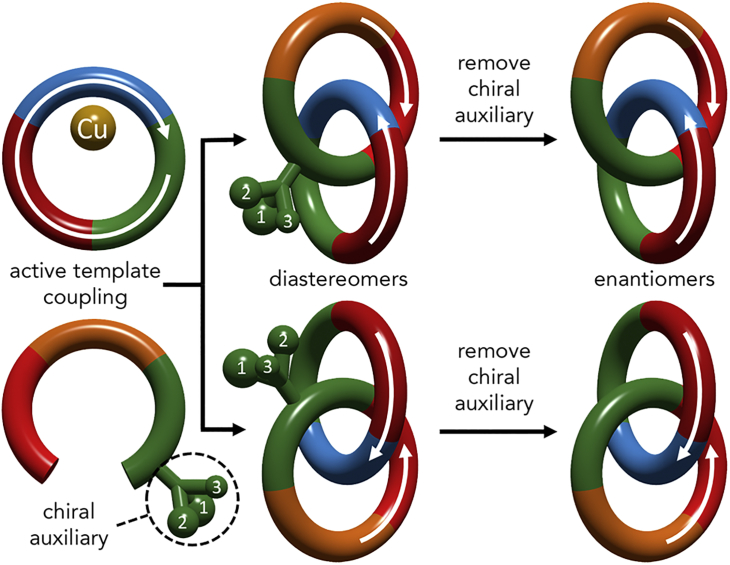
Schematic of Our Proposed Approach to Topologically Chiral Catenanes

Much less widely known, and even less well explored, are the stereogenic elements that can arise in systems where two or more covalent subcomponents with suitable symmetry properties are permanently held together in a defined orientation by threading through one another to create a mechanical bond.^7^, ^8^, ^9^, ^10^ The first of these to be identified, the “topologically chiral” catenanes (Figure 1), were discussed by Wasserman and Frisch in their seminal 1961 work on chemical topology.^11^ This stereogenic unit is extremely unusual in that it is invariant when treated under the rules of mathematical topology; in contrast to simple covalent stereogenic units, the enantiomers of which can be interchanged by relaxing the Euclidean properties of molecular bonding (i.e., fixed bond lengths and angles) while maintaining atomic connectivity, topological stereoisomers cannot be exchanged without breaking and reforming atomic connections and are thus topologically invariant.^12^, ^13^

Over two decades passed between the identification of the potential for topological chirality in catenanes and the first isolation of the enantiomers of topologically chiral catenanes; Sauvage and Okamoto succeeded in separating the topological enantiomers of a catenane by using preparative chiral stationary phase high-performance liquid chromatography (PCSP-HPLC),^14^ a technique that allows the purification of small quantities of chiral molecules. Unfortunately, the techniques used to selectively produce chiral molecules based on covalent stereogenic units in a scalable manner have not been applied successfully to topologically chiral catenanes. As a result, the examples of enantiopure catenanes in which the mechanical bond provides the only fixed stereogenic unit all make use of PCSP-HPLC to separate the enantiomeric products.^14^, ^15^ This has prevented their investigation in enantioselective catalysis and sensing and materials science, even as examples of chiral interlocked molecules based on covalent stereogenic units^15^, ^16^, ^17^, ^18^, ^19^ and other chirotopic mechanical stereogenic elements^20^, ^21^ have begun to show promise in these areas.^8^

Building on our previous approach to mechanically planar chiral rotaxanes,^22^, ^23^ we propose a new approach to enantiopure topologically chiral catenanes (Figure 1). Our proposed methodology makes use of the properties of molecules that contain two stereogenic units, one of which is a classical covalent stereogenic center and the other of which is the mechanical topological element that arises from the enchained rings. Our proposed chiral auxiliary approach,^24^ including a covalent stereogenic center of fixed configuration in one of the rings in an active template coupling, gives rise to two possible interlocked products that differ only in the configuration of the mechanical bond. These diastereomers can be separated, at least in principle, by simple chemical means (e.g., silica gel chromatography) because they are no longer related as object and mirror image and thus have distinct physical properties. Once they are separated, “deleting” the covalent stereogenic unit from the catenanes would give rise to the separated mirror-image catenanes as single isomers, completely circumventing the need for enantiomer separation.

## Results and Discussion

We recently reported an improved^25^, ^26^ active-template^27^ Cu-mediated azide-alkyne cycloaddition^28^, ^29^ (AT-CuAAC)^30^ methodology for the synthesis of sterically crowded catenanes in excellent yield.^31^ We selected this methodology to demonstrate our proposed chiral auxiliary approach to topologically chiral catenanes because diastereomeric small crowded molecules, in which the topological and covalent elements of stereochemistry are held in close proximity and thus interact strongly, are *a priori* more likely to be separable. The required precursors, azide or alkyne pre-macrocycle (*R*)-**1** (which contains a fixed stereogenic center derived from an enantiopure amino acid) and macrocycles **2**^32^ were synthesized in a straightforward manner from readily available building blocks (see Supplemental Information).

**Scheme 1:**
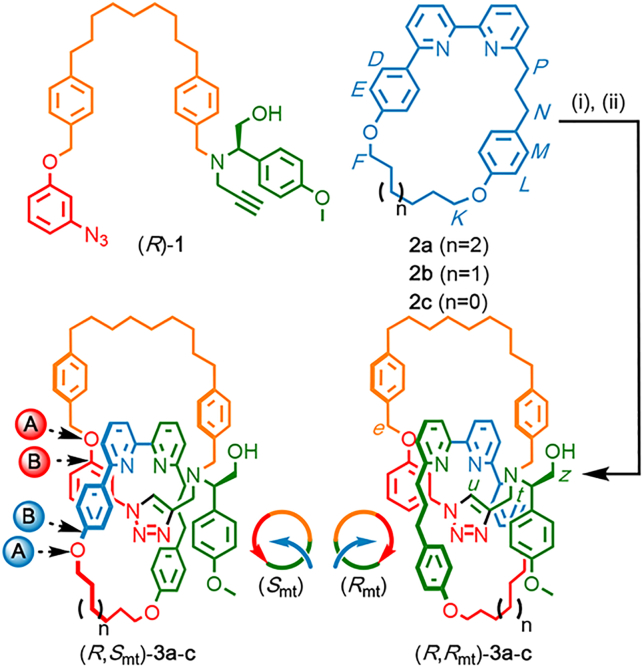
Synthesis of Diastereomeric Catenanes **3** Reagents and conditions: (i) slow addition (4 h) of (*R*)-**1** to macrocycle **2**, [Cu(MeCN)_4_]PF_6_, N^*i*^Pr_2_Et, CHCl_3_-EtOH (1:1) at 60°C; (ii) KCN and CH_2_Cl_2_-MeOH (1:1). (*R*,*R*/*S*_mt_)-**3a**: n = 2, 1:1 inseparable mixture, 72% combined isolated yield; (*R*,*R*/*S*_mt_)-**3b**: n = 1, 2:1 separable mixture favoring (*R*,*S*_mt_)-**3b**, 89% combined isolated yield; (*R*,*R*/*S*_mt_)-**3c**: n = 0, 1:1 inseparable mixture: ∼23% conversion of **2c** by ^1^H NMR analysis of the unpurified reaction mixture.

When pre-macrocycle (*R*)-**1** was added slowly to a solution of macrocycle **2a** and a copper catalyst (Scheme 1), catenanes **3a** were formed in high isolated yield (72%) as a 50:50 mixture of two interlocked products, the analytical data (nuclear magnetic resonance [NMR] and liquid chromatography-mass spectrometry [LC-MS]) of which were consistent with diastereomers. Disappointingly, we were unable to separate the stereoisomers of catenane **3a** by using simple chemical techniques; although diastereomers can theoretically be separated, this is not always practically true. Pleasingly, replacing macrocycle **2a** with smaller macrocycle **2b** gave rise to catenane **3b**, and in this case, the isomers were separable in good yield (57% major, 32% minor, and 89% combined yield). Moreover, the two possible products were formed in unequal amounts in a ratio of ∼2:1 as judged by ^1^H NMR analysis of the unpurified reaction mixture, selectivity that increases the overall yield of the major isomer. It is important to note that selectivity and separability are not related in a simple manner to the size of the bipyridine-containing ring; when smaller macrocycle **2c** was used, catenane **3c** was formed with no selectivity as an inseparable mixture.^33^, ^34^

**Figure 2:**
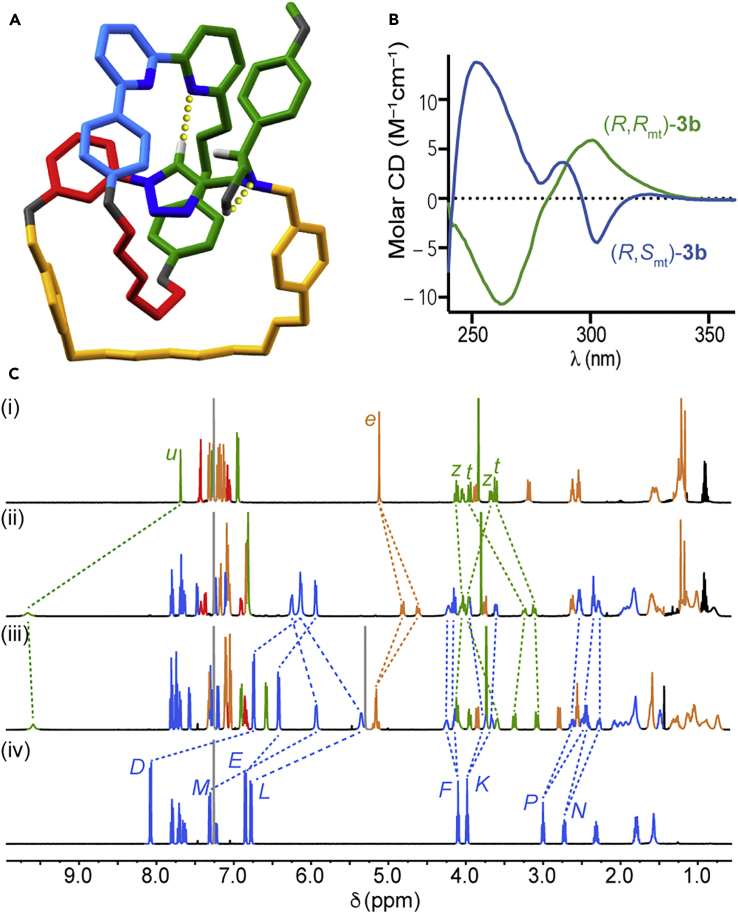
Characterization of Catenanes **3b** (A) Solid-state structure of major diastereomer (*R*,*S*_mt_)-**3b**^35^ with selected intercomponent interactions highlighted (selected distances [Å]: H_*u*_···N = 2.35, OH···N = 2.28). (B) CD spectra of (35 μM in CHCl_3_) (*R*,*S*_mt_)-**3b** and (*R*,*R*_mt_)-**3b**. (C) Partial stacked ^1^H NMR spectra (500 MHz, 298 K, CDCl_3_) of (i) the corresponding non-interlocked triazole macrocycle derived from (*S*)-**1**, (ii) catenane (*R*,*R*_mt_)-**3b**, (iii) catenane (*R*,*S*_mt_)-**3b**, and (iv) macrocycle **2b**. Selected signals are assigned and color coded (see Scheme 1). Signals corresponding to macrocycle **2b** are all shown in blue for clarity.

In order to assign the absolute stereochemistry of the interlocked products, we grew single crystals of a racemic^35^ sample of the major isomer of **3b** and subjected them to X-ray diffraction analysis (Figure 2A). This allowed us to determine the relative orientation of the interlocked rings. We assigned absolute stereochemical labels by considering the relative orientation of each macrocycle’s polar vectors, which followed a path from the highest-priority atom (A, assigned by the Cahn-Ingold-Prelog method) to the highest-priority ligand (B) of that atom. Once we assigned these vectors, we determined the absolute stereochemistry by orienting the assembly with the polar vector of one ring passing away from the observer through the cavity of the other and observing the orientation of the second polar vector; clockwise was assigned *R*_mt_, and anticlockwise was assigned *S*_mt_, and we propose that the “mt” suffix be used to highlight the mechanical topological origin of the stereochemistry.^7^ Using this approach, we determined the absolute stereochemistry of the major isomer to be (*R*,*S*_mt_)-**3b** and, by a process of elimination, the minor isomer determined to be (*R*,*R*_mt_).

The ^1^H NMR spectra of separated diastereomeric catenanes **3b** were clearly different from those of the corresponding non-interlocked components (Figure 2C); in both interlocked products, triazole resonance H_*u*_ appeared at higher ppm than the corresponding non-interlocked macrocycle, consistent with a H bond between this polarized C–H and a bipyridine N, as observed in the solid-state structure of racemic (*R*,*S*_mt_)-**3b**,^36^ and many other signals (e.g., flanking aromatic ring protons H_*D*_, H_*E*_, H_*L*_, and H_*M*_) shifted to lower ppm, consistent with the crowded environment of the mechanical bond. Mechanical bond formation also rendered several geminal methylene signals of the bipyridine macrocycle diastereotopic; protons H_*F*_, H_*K*_, H_*P*_, and H_*N*_, which are single environments in macrocycle **2b**, split apart into diastereotopic sets in catenanes **3b**.

Despite these gross similarities, the separated isomers of **3b** were clearly chemically distinct by ^1^H NMR; benzylic protons H_*e*_ appeared as an AB quartet in catenane (*R*,*S*_mt_)-**3b** and as separated doublets in (*R*,*R*_mt_)-**3b**. Similarly, H_*D*_, H_*E*_, H_*L*_, and H_*M*_ appeared close to one another in (*R*,*R*_mt_)-**3b** but were more widely dispersed in diastereomer (*R*,*S*_mt_)-**3b**. The circular dichroism (CD) spectra of the diastereomers were also clearly distinct: compared with the minor diastereomer (*R*,*R*_mt_)-**3b**, the major (*R*,*S*_mt_)-**3b** diastereomer displayed an additional peak (Figure 2B). Perhaps surprisingly, aside from the additional peak and a slight shift in the lower-wavelength signal, the CD traces of (*R*,*S*_mt_)-**3b** and (*R*,*R*_mt_)-**3b** were roughly mirror images of one another, suggesting that the topological element of stereochemistry dominates the appearance of the CD spectra.

**Scheme 2:**
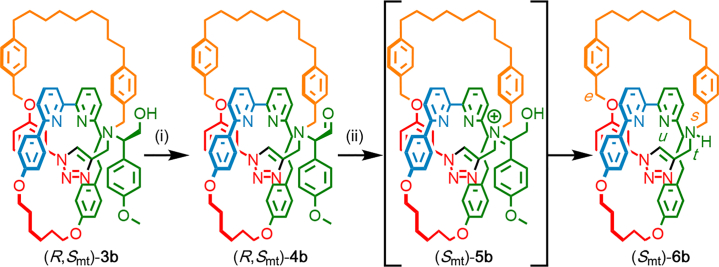
Cleavage of the Chiral Auxiliary from Catenane (*R*,*S*_mt_)-3b to Give Catenane (*S*_mt_)-**6b** Reagents and conditions: (i) (COCl)_2_, DMSO, NEt_3_, and CH_2_Cl_2_ at room temperature (RT); (ii) AcOH and CHCl_3_ at RT. **6b** was isolated in 68% yield over two steps.

Having demonstrated the synthesis and separation of topologically epimeric catenanes **3b**, we turned our attention to removing the covalent stereogenic element in order to produce enantiomeric catenanes **6b** (Scheme 2). The chiral auxiliary unit bore a striking resemblance to the achiral para-methoxybenzene (PMB) protecting group, and our original intention was to remove it in an analogous manner to a PMB group either by treatment with acid or by oxidation.^37^ However, treatment of (*R*,*S*_mt_)-**3b** with trifluoroacetic acid or oxidation with Ce(IV) led to extensive decomposition, and LC-MS showed cleavage of the triazole-containing macrocycle. Ultimately, the covalent stereogenic unit was cleaved from (*R*,*S*_mt_)-**3b** by a stepwise process of oxidation and hydrolysis inspired by the Amadori rearrangement of iminosugars;^38^, ^39^ treatment of (*R*,*S*_mt_)-**3b** under Swern^40^ conditions gave aldehyde **4b**, which was not isolated but immediately treated with acetic acid. Acetic acid catalyzed the removal of the auxiliary to give catenane **6b** presumably by isomerization of α-amino aldehyde catenane **4b** to the α-hydroxy iminium tautomer **5b** with subsequent hydrolysis.

**Figure 3:**
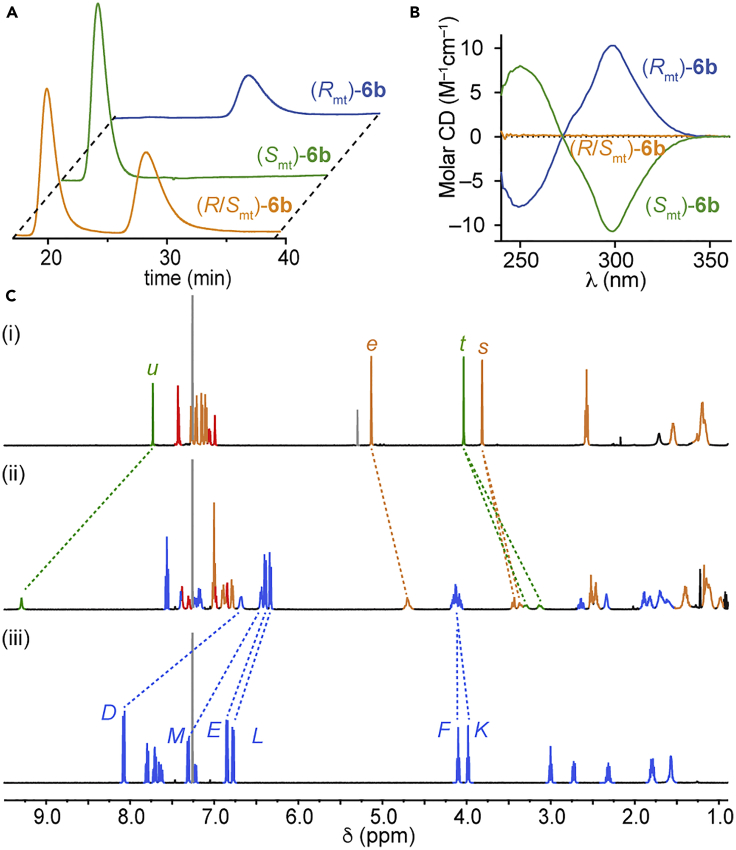
Characterization of Catenanes **6b** (A) Analytical chiral stationary phase HPLC chromatograms (RegisCell, 98:2 hexane-^*i*^PrOH, 0.5 mL/min) of (*R*_mt_)-**6b** (blue), (*S*_mt_)-**6b** (green), and racemic **6b** (orange). (B) CD spectra (35.0 μM in CHCl_3_, 293 K) of (*R*_mt_)-**6b** (blue), (*S*_mt_)-**6b** (green), and racemic **6b** (orange). (C) Partial stacked ^1^H NMR spectra (500 MHz, 298 K, CDCl_3_) of (i) the corresponding non-interlocked triazole macrocycle of catenane **6b**, (ii) catenane (*S*_mt_)-**6b**, and (iii) macrocycle **2b**. Selected signals are assigned and color coded (see Schemes 1 and 2). Signals corresponding to macrocycle **2b** are all shown in blue for clarity.

Catenane **6b** no longer contained a covalent stereogenic unit, and thus the topological stereogenic unit was the only remaining fixed stereochemical feature.^41^ The stereochemical purity of the products was confirmed by analytical CSP-HPLC (Figure 3A). A racemic sample of **6b** displayed two clear peaks in the chromatogram, whereas single peaks (<1:99 purity) were observed for the separated enantiomers. To assign the topological stereogenic unit, we considered that the relative orientation of the two rings remained unchanged during the cleavage of the auxiliary and applied the same approach discussed above for catenanes **3**; thus, (*R*,*S*_mt_)-**3b** gave rise to (*S*_mt_)-**6b**. The mirror-image isomer (*R*_mt_)-**6b** was synthesized starting from (*S*)-**1**. Analysis of catenanes (*R*_mt_)-**6b** (Figure 3C) and (S_mt_)-**6b** by ^1^H NMR confirmed that they were chemically identical with the exception of the topological element of stereochemistry; the spectra of the isomers were identical and, compared with their non-interlocked components, exhibited the expected changes in chemical shift. H_*u*_ shifted to higher ppm; H_*D*_, H_*E*_, H_*L*_, and H_*M*_ shifted to lower ppm; and protons H_*s*_ and H_*t*_, which are singlets in the non-interlocked macrocycle, were split into diastereotopic signals in the interlocked structure. CD spectroscopy (Figure 3B) confirmed the enantiomeric nature of the structures by revealing identical but mirror-image spectra for the two enantiomers.

### Conclusions

We have demonstrated that by combining a covalent stereogenic unit with a topological element of stereochemistry, it is possible to stereoselectively produce separable topological catenane epimers. Subsequent cleavage of the covalent stereogenic element from the separated products gave enantiopure topologically chiral catenane products. Although Sanders,^42^ Gagne,^43^ and Trabolsi^44^ have previously reported isolated examples of the serendipitous stereoselective synthesis of topological homo[2]catenane diastereomers under thermodynamic control, the multiple elements of covalent stereochemistry used to direct the stereochemical outcome of the reaction remained in the final product, whereas our chiral auxiliary approach gives access to molecules in which the mechanical bond provides the sole fixed stereogenic unit.^41^ Our chiral auxiliary concept is technically simple and, given the generality of the AT-CuAAC catenane-forming reaction,^31^ makes functionalized topologically chiral catenanes in which the mechanical bond provides the only stereogenic unit available for the first time without the need for CSP-HPLC separation.

## Data and Software Availability

The accession number for the solid-state structure of (*R*,*S*_mt_)**-3b** reported in this paper is CCDC: 1885204. Processed compound characterization data (NMR, circular dichroism, HPLC, and MS) are available freely from the University of Southampton repository (https://doi.org/10.5258/SOTON/D0828).

## Supplemental Information

Document S1. Figures S1–S110, Schemes S1 and S2, Table S1, and Supplemental Experimental Procedures

Data S1. CIF for Catenane (R,Smt)-**3b**

Data S2. ZIP File Containing Modelled Structures in MOL Format

Document S2. Article plus Supplemental Information
