# Supplementary material for: An Auxiliary Approach for the Stereoselective Synthesis of Topologically Chiral Catenanes
Source: Chem. 2019 Jun 13;5(6):1512–20. doi: 10.1016/j.chempr.2019.03.008 (PMC6588264; doi:10.1016/j.chempr.2019.03.008)
Supplement: Document S2. Article plus Supplemental Information [file mmc4.pdf]

Chem

CellPress

Article

An Auxiliary Approach for the Stereoselective Synthesis of Topologically Chiral Catenanes

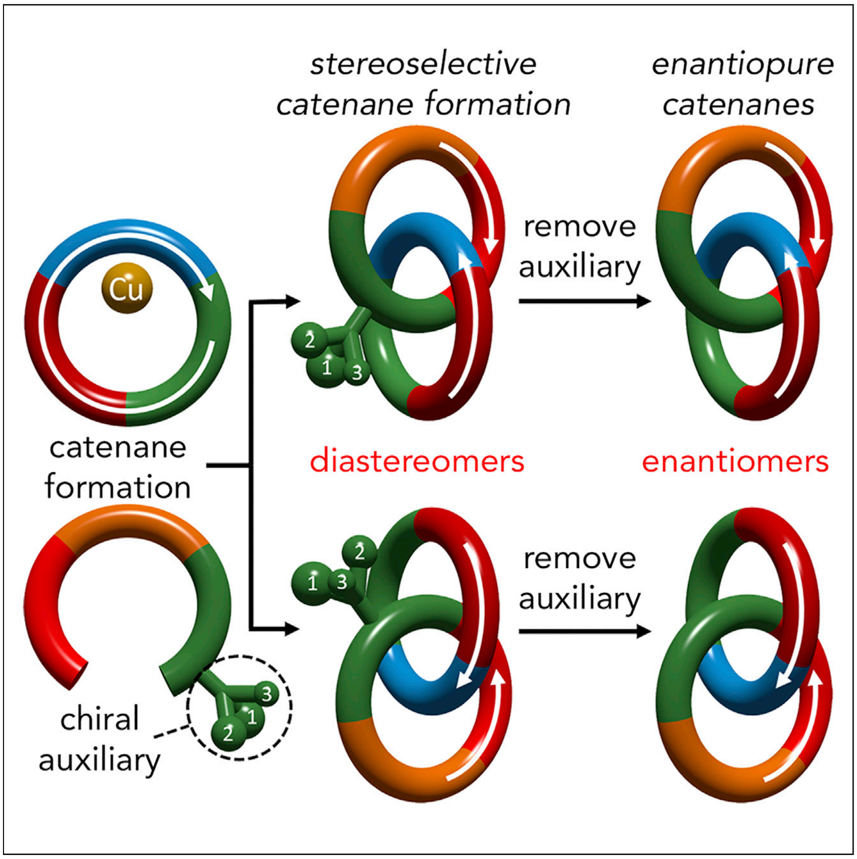

Catenanes, molecules comprising two rings held together like links in a chain, can exist as two mirror-image forms if the rings lack bilateral symmetry. These "topological enantiomers" are unusual because they cannot be interconverted by stretching or bending chemical bonds. To date, their synthesis has required the separation of mirror-image structures by specialist techniques. We present a simple method to allow the synthesis of topologically chiral catenanes, opening them up to investigation in catalysis, sensing, and materials science.

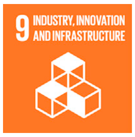

Mathieu Denis, James E.M. Lewis, Florian Modicom, Stephen M. Goldup

s.goldup@soton.ac.uk

HIGHLIGHTS

First stereoselective synthesis of a topologically chiral catenane

First absolute stereochemical assignment of a topologically chiral catenane

First example of an auxiliary approach to topologically chiral catenanes

Denis et al., Chem 5, 1512–1520
June 13, 2019 © 2019 The Author(s). Published by Elsevier Inc.
https://doi.org/10.1016/j.chempr.2019.03.008

Chem

CellPress

Article

An Auxiliary Approach for the Stereoselective Synthesis of Topologically Chiral Catenanes

Mathieu Denis,[1] James E.M. Lewis,[1,2] Florian Modicom,[1] and Stephen M. Goldup[1,3,*]

SUMMARY

Catenanes, molecules in which two rings are threaded through one another like links in a chain, can form as two structures related like an object and its mirror image but otherwise identical if the individual rings lack bilateral symmetry. These structures are described as "topologically chiral" because, unlike most chiral molecules, it is not possible to convert one mirror-image form to the other under the rules of mathematical topology. Although intriguing and discussed as early as 1961, to date all methods of accessing molecules containing only this topological stereogenic element require the separation of the mirror-image forms via chiral stationary phase high-performance liquid chromatography, which has limited their investigation to date. Here, we present a simple method that uses a readily available source of chiral information to allow the stereoselective synthesis of topologically chiral catenanes.

The Bigger Picture

Chiral molecules have occupied a special place in chemistry since Pasteur reported the painstaking separation of mirror-image crystals of tartaric acid salts in 1848. In the 21st century, chiral molecules remain a major scientific focus because of their importance in biology and their emerging applications in materials science. However, topologically chiral molecules, such as the catenanes described here, have received little attention because they are hard to make; preparative chiral stationary phase high-performance liquid chromatography allows the separation of their mirror-image forms but only on a very small scale. Here, we demonstrate the synthesis of topologically chiral catenanes by using standard synthetic techniques, marking their transition from "inaccessible curiosities" to valid synthetic targets for investigation in catalysis, sensing, medicinal chemistry, and materials science. Furthermore, this work will inspire efforts to access other neglected classes of chiral interlocked molecules.

INTRODUCTION

Chiral molecules occupy a special place in synthetic chemistry because of their ubiquity in biological systems and emerging applications in materials science.[1] A tetrahedral carbon atom bearing four different substituents is the archetypal unit that can give rise to molecular chirality.[2–4] However, chirality in organic molecules can arise because of a number of different covalent structural features in addition to such stereogenic centers, the most common examples of which are in molecules where atoms are arranged suitably around a fixed axis (commonly referred to as "axially chiral") such that they facially desymmetrize an oriented plane ("planar chiral") or are displayed in a helical arrangement ("helically chiral").[5,6] Regardless of the structural origin of molecular chirality, the key challenge in the synthesis of chiral molecules is the production of pure samples of one mirror-image form (enantiomer) of the product; because the different enantiomers of a chiral molecule by definition have identical properties under most circumstances, they must either be produced selectively or separated by specialist techniques. Thus, a significant amount of effort has been devoted to achieving these goals efficiently over the past century of synthetic chemistry research.

Much less widely known, and even less well explored, are the stereogenic elements that can arise in systems where two or more covalent subcomponents with suitable symmetry properties are permanently held together in a defined orientation by threading through one another to create a mechanical bond.[7–10] The first of these to be identified, the "topologically chiral" catenanes (Figure 1), were discussed by Wasserman and Frisch in their seminal 1961 work on chemical topology.[11] This stereogenic unit is extremely unusual in that it is invariant when treated under the rules of

 © 2019 The Author(s). Published by Elsevier Inc.
This is an open access article under the CC BY license (http://creativecommons.org/licenses/by/4.0/).

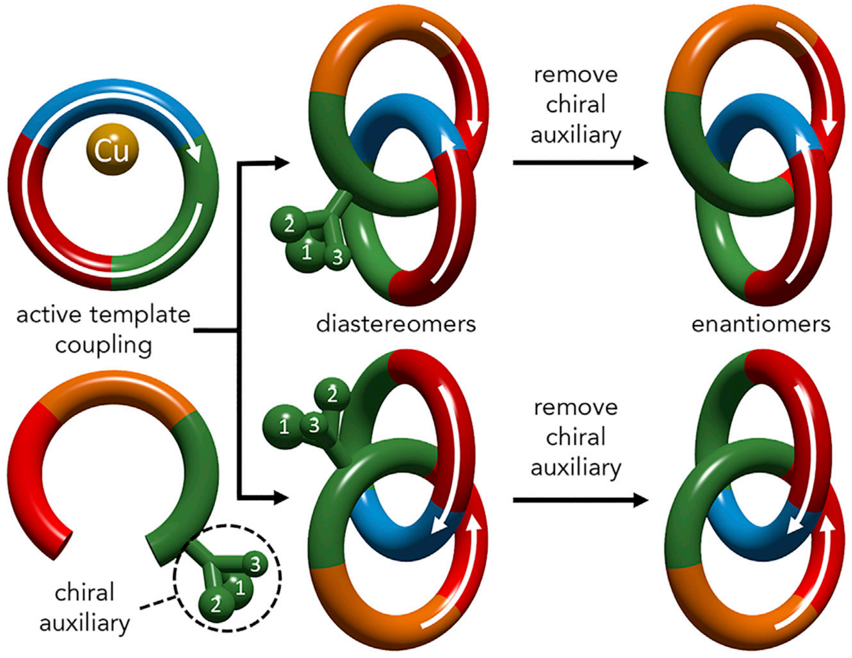

Figure 1. Schematic of Our Proposed Approach to Topologically Chiral Catenanes

mathematical topology; in contrast to simple covalent stereogenic units, the enantiomers of which can be interchanged by relaxing the Euclidean properties of molecular bonding (i.e., fixed bond lengths and angles) while maintaining atomic connectivity, topological stereoisomers cannot be exchanged without breaking and reforming atomic connections and are thus topologically invariant.[12,13]

Over two decades passed between the identification of the potential for topological chirality in catenanes and the first isolation of the enantiomers of topologically chiral catenanes; Sauvage and Okamoto succeeded in separating the topological enantiomers of a catenane by using preparative chiral stationary phase high-performance liquid chromatography (PCSP-HPLC),[14] a technique that allows the purification of small quantities of chiral molecules. Unfortunately, the techniques used to selectively produce chiral molecules based on covalent stereogenic units in a scalable manner have not been applied successfully to topologically chiral catenanes. As a result, the examples of enantiopure catenanes in which the mechanical bond provides the only fixed stereogenic unit all make use of PCSP-HPLC to separate the enantiomeric products.[14,15] This has prevented their investigation in enantioselective catalysis and sensing and materials science, even as examples of chiral interlocked molecules based on covalent stereogenic units[15–19] and other chirotopic mechanical stereogenic elements[20,21] have begun to show promise in these areas.[8]

Building on our previous approach to mechanically planar chiral rotaxanes,[22,23] we propose a new approach to enantiopure topologically chiral catenanes (Figure 1). Our proposed methodology makes use of the properties of molecules that contain two stereogenic units, one of which is a classical covalent stereogenic center and the other of which is the mechanical topological element that arises from the enchained rings. Our proposed chiral auxiliary approach,[24] including a covalent stereogenic center of fixed configuration in one of the rings in an active template coupling, gives

[1]School of Chemistry, University of Southampton, Highfield, Southampton SO17 1BJ, UK

[2]Department of Chemistry, Imperial College London, Molecular Sciences Research Hub, 80 Wood Lane, London W12 0BZ, UK

[3]Lead Contact

*Correspondence: s.goldup@soton.ac.uk

https://doi.org/10.1016/j.chempr.2019.03.008

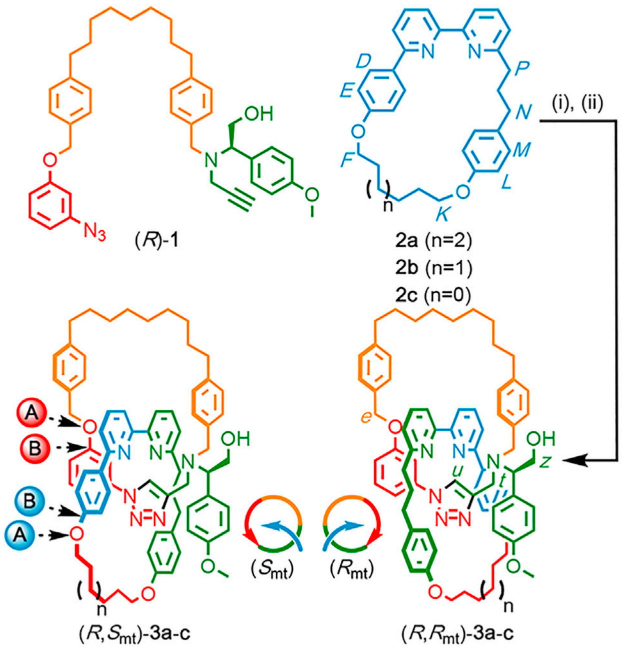

Scheme 1. Synthesis of Diastereomeric Catenanes 3

Reagents and conditions: (i) slow addition (4 h) of (*R*)-**1** to macrocycle **2**, $[Cu(MeCN)_4]PF_6$, N^iPr_2Et, $CHCl_3$-EtOH (1:1) at 60°C; (ii) KCN and CH_2Cl_2-MeOH (1:1). ($R,R/S_{mt}$)-**3a**: n = 2, 1:1 inseparable mixture, 72% combined isolated yield; ($R,R/S_{mt}$)-**3b**: n = 1, 2:1 separable mixture favoring (R,S_{mt})-**3b**, 89% combined isolated yield; ($R,R/S_{mt}$)-**3c**: n = 0, 1:1 inseparable mixture: ~23% conversion of **2c** by ^{1}H NMR analysis of the unpurified reaction mixture.

rise to two possible interlocked products that differ only in the configuration of the mechanical bond. These diastereomers can be separated, at least in principle, by simple chemical means (e.g., silica gel chromatography) because they are no longer related as object and mirror image and thus have distinct physical properties. Once they are separated, "deleting" the covalent stereogenic unit from the catenanes would give rise to the separated mirror-image catenanes as single isomers, completely circumventing the need for enantiomer separation.

RESULTS AND DISCUSSION

We recently reported an improved[25,26] active-template[27] Cu-mediated azide-alkyne cycloaddition[28,29] (AT-CuAAC)[30] methodology for the synthesis of sterically crowded catenanes in excellent yield.[31] We selected this methodology to demonstrate our proposed chiral auxiliary approach to topologically chiral catenanes because diastereomeric small crowded molecules, in which the topological and covalent elements of stereochemistry are held in close proximity and thus interact strongly, are *a priori* more likely to be separable. The required precursors, azide or alkyne pre-macrocycle (*R*)-**1** (which contains a fixed stereogenic center derived from an enantiopure amino acid) and macrocycles **2**[32] were synthesized in a straightforward manner from readily available building blocks (see Supplemental Information).

When pre-macrocycle (*R*)-**1** was added slowly to a solution of macrocycle **2a** and a copper catalyst (Scheme 1), catenanes **3a** were formed in high isolated yield (72%) as a 50:50 mixture of two interlocked products, the analytical data (nuclear magnetic resonance [NMR] and liquid chromatography-mass spectrometry [LC-MS]) of which were consistent with diastereomers. Disappointingly, we were unable to

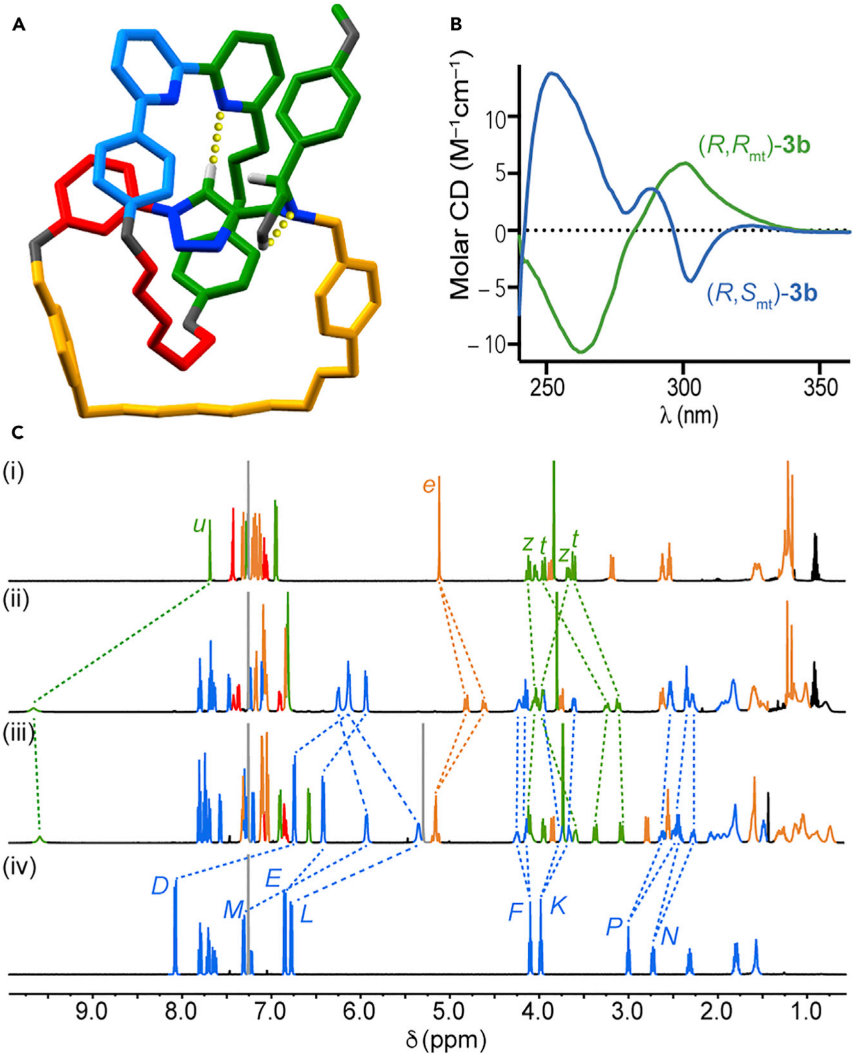

Figure 2. Characterization of Catenanes 3b

(A) Solid-state structure of major diastereomer (R,S_{mt})-**3b**[35] with selected intercomponent interactions highlighted (selected distances [Å]: $H_u\cdots N$ = 2.35, $OH\cdots N$ = 2.28).

(B) CD spectra of (35 μM in $CHCl_3$) (R,S_{mt})-**3b** and (R,R_{mt})-**3b**.

(C) Partial stacked ^1H NMR spectra (500 MHz, 298 K, $CDCl_3$) of (i) the corresponding non-interlocked triazole macrocycle derived from (S)-**1**, (ii) catenane (R,R_{mt})-**3b**, (iii) catenane (R,S_{mt})-**3b**, and (iv) macrocycle **2b**. Selected signals are assigned and color coded (see Scheme 1). Signals corresponding to macrocycle **2b** are all shown in blue for clarity.

separate the stereoisomers of catenane **3a** by using simple chemical techniques; although diastereomers can theoretically be separated, this is not always practically true. Pleasingly, replacing macrocycle **2a** with smaller macrocycle **2b** gave rise to catenane **3b**, and in this case, the isomers were separable in good yield (57% major, 32% minor, and 89% combined yield). Moreover, the two possible products were formed in unequal amounts in a ratio of ~2:1 as judged by ^1H NMR analysis of the unpurified reaction mixture, selectivity that increases the overall yield of the major isomer. It is important to note that selectivity and separability are not related in a simple manner to the size of the bipyridine-containing ring; when smaller macrocycle **2c** was used, catenane **3c** was formed with no selectivity as an inseparable mixture.[33,34]

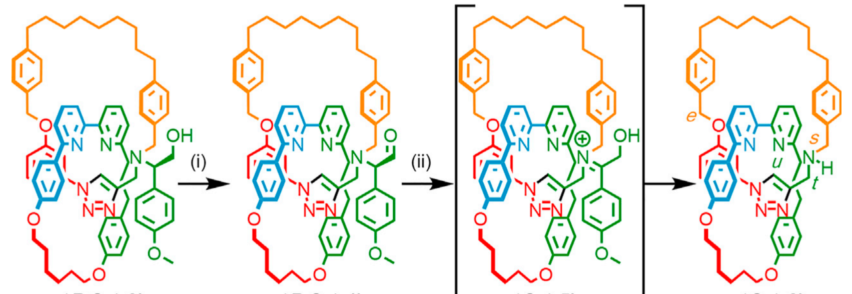

Scheme 2. Cleavage of the Chiral Auxiliary from Catenane (*R*,S_{mt})-3b to Give Catenane (S_{mt})-6b
Reagents and conditions: (i) $(COCl)_2$, DMSO, NEt_3, and CH_2Cl_2 at room temperature (RT); (ii) AcOH and $CHCl_3$ at RT. **6b** was isolated in 68% yield over two steps.

In order to assign the absolute stereochemistry of the interlocked products, we grew single crystals of a racemic[35] sample of the major isomer of **3b** and subjected them to X-ray diffraction analysis (Figure 2A). This allowed us to determine the relative orientation of the interlocked rings. We assigned absolute stereochemical labels by considering the relative orientation of each macrocycle's polar vectors, which followed a path from the highest-priority atom (A, assigned by the Cahn-Ingold-Prelog method) to the highest-priority ligand (B) of that atom. Once we assigned these vectors, we determined the absolute stereochemistry by orienting the assembly with the polar vector of one ring passing away from the observer through the cavity of the other and observing the orientation of the second polar vector; clockwise was assigned R_{mt}, and anticlockwise was assigned S_{mt}, and we propose that the "mt" suffix be used to highlight the mechanical topological origin of the stereochemistry.[7] Using this approach, we determined the absolute stereochemistry of the major isomer to be (*R*,S_{mt})-**3b** and, by a process of elimination, the minor isomer determined to be (*R*,R_{mt}).

The ^{1}H NMR spectra of separated diastereomeric catenanes **3b** were clearly different from those of the corresponding non-interlocked components (Figure 2C); in both interlocked products, triazole resonance H_u appeared at higher ppm than the corresponding non-interlocked macrocycle, consistent with a H bond between this polarized C–H and a bipyridine N, as observed in the solid-state structure of racemic (*R*,S_{mt})-**3b**,[36] and many other signals (e.g., flanking aromatic ring protons H_D, H_E, H_L, and H_M) shifted to lower ppm, consistent with the crowded environment of the mechanical bond. Mechanical bond formation also rendered several geminal methylene signals of the bipyridine macrocycle diastereotopic; protons H_F, H_K, H_P, and H_N, which are single environments in macrocycle **2b**, split apart into diastereotopic sets in catenanes **3b**.

Despite these gross similarities, the separated isomers of **3b** were clearly chemically distinct by ^{1}H NMR; benzylic protons H_e appeared as an AB quartet in catenane (*R*,S_{mt})-**3b** and as separated doublets in (*R*,R_{mt})-**3b**. Similarly, H_D, H_E, H_L, and H_M appeared close to one another in (*R*,R_{mt})-**3b** but were more widely dispersed in diastereomer (*R*,S_{mt})-**3b**. The circular dichroism (CD) spectra of the diastereomers were also clearly distinct: compared with the minor diastereomer (*R*,R_{mt})-**3b**, the major (*R*,S_{mt})-**3b** diastereomer displayed an additional peak (Figure 2B). Perhaps surprisingly, aside from the additional peak and a slight shift in the lower-wavelength signal, the CD traces of (*R*,S_{mt})-**3b** and (*R*,R_{mt})-**3b** were roughly mirror images of one another, suggesting that the topological element of stereochemistry dominates the appearance of the CD spectra.

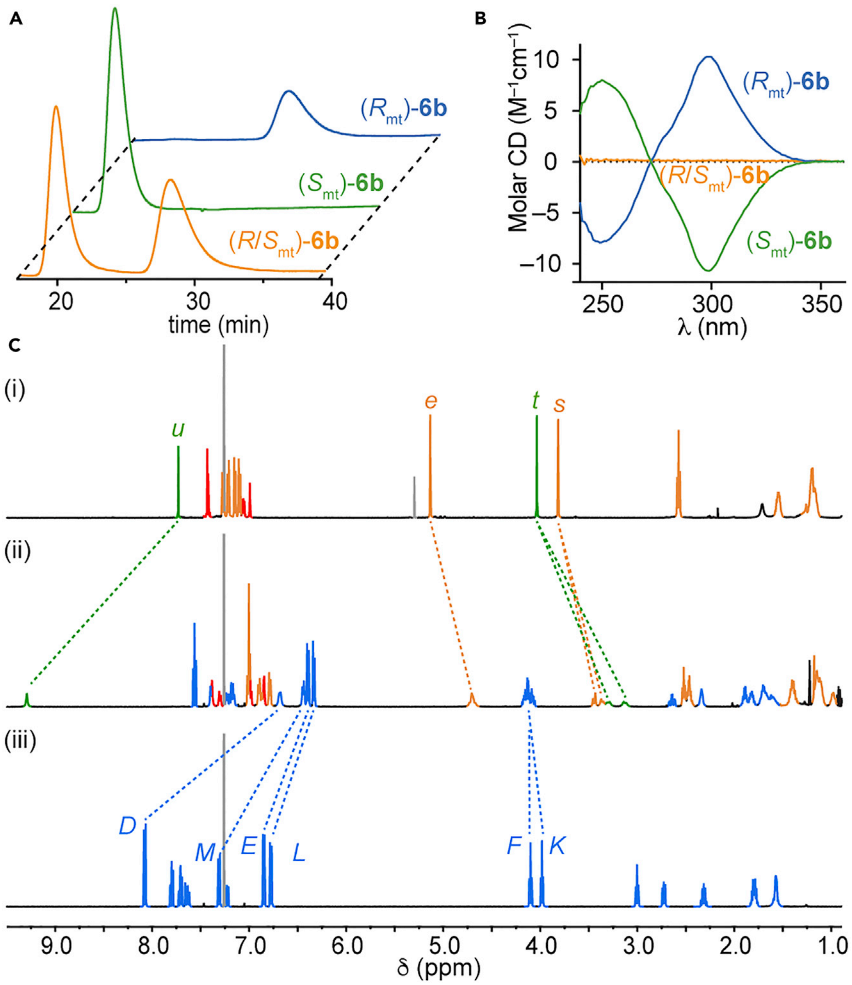

Figure 3. Characterization of Catenanes 6b

(A) Analytical chiral stationary phase HPLC chromatograms (RegisCell, 98:2 hexane-^{i}PrOH, 0.5 mL/min) of (R_{mt})-**6b** (blue), (S_{mt})-**6b** (green), and racemic **6b** (orange).

(B) CD spectra (35.0 μM in $CHCl_3$, 293 K) of (R_{mt})-**6b** (blue), (S_{mt})-**6b** (green), and racemic **6b** (orange).

(C) Partial stacked ^{1}H NMR spectra (500 MHz, 298 K, $CDCl_3$) of (i) the corresponding non-interlocked triazole macrocycle of catenane **6b**, (ii) catenane (S_{mt})-**6b**, and (iii) macrocycle **2b**. Selected signals are assigned and color coded (see Schemes 1 and 2). Signals corresponding to macrocycle **2b** are all shown in blue for clarity.

Having demonstrated the synthesis and separation of topologically epimeric catenanes **3b**, we turned our attention to removing the covalent stereogenic element in order to produce enantiomeric catenanes **6b** (Scheme 2). The chiral auxiliary unit bore a striking resemblance to the achiral para-methoxybenzene (PMB) protecting group, and our original intention was to remove it in an analogous manner to a PMB group either by treatment with acid or by oxidation.[37] However, treatment of (R,S_{mt})-**3b** with trifluoroacetic acid or oxidation with Ce(IV) led to extensive decomposition, and LC-MS showed cleavage of the triazole-containing macrocycle. Ultimately, the covalent stereogenic unit was cleaved from (R,S_{mt})-**3b** by a stepwise process of oxidation and hydrolysis inspired by the Amadori rearrangement of iminosugars;[38,39] treatment of (R,S_{mt})-**3b** under Swern[40] conditions gave aldehyde **4b**, which was not isolated but immediately treated with acetic acid. Acetic acid catalyzed the removal of the auxiliary to give catenane **6b** presumably by isomerization

of α-amino aldehyde catenane **4b** to the α-hydroxy iminium tautomer **5b** with subsequent hydrolysis.

Catenane **6b** no longer contained a covalent stereogenic unit, and thus the topological stereogenic unit was the only remaining fixed stereochemical feature.[41] The stereochemical purity of the products was confirmed by analytical CSP-HPLC (Figure 3A). A racemic sample of **6b** displayed two clear peaks in the chromatogram, whereas single peaks (<1:99 purity) were observed for the separated enantiomers. To assign the topological stereogenic unit, we considered that the relative orientation of the two rings remained unchanged during the cleavage of the auxiliary and applied the same approach discussed above for catenanes **3**; thus, (R,S_{mt})-**3b** gave rise to (S_{mt})-**6b**. The mirror-image isomer (R_{mt})-**6b** was synthesized starting from (S)-**1**. Analysis of catenanes (R_{mt})-**6b** (Figure 3C) and (S_{mt})-**6b** by ^{1}H NMR confirmed that they were chemically identical with the exception of the topological element of stereochemistry; the spectra of the isomers were identical and, compared with their non-interlocked components, exhibited the expected changes in chemical shift. H_u shifted to higher ppm; H_D, H_E, H_L, and H_M shifted to lower ppm; and protons H_s and H_t, which are singlets in the non-interlocked macrocycle, were split into diastereotopic signals in the interlocked structure. CD spectroscopy (Figure 3B) confirmed the enantiomeric nature of the structures by revealing identical but mirror-image spectra for the two enantiomers.

Conclusions

We have demonstrated that by combining a covalent stereogenic unit with a topological element of stereochemistry, it is possible to stereoselectively produce separable topological catenane epimers. Subsequent cleavage of the covalent stereogenic element from the separated products gave enantiopure topologically chiral catenane products. Although Sanders,[42] Gagne,[43] and Trabolsi[44] have previously reported isolated examples of the serendipitous stereoselective synthesis of topological homo[2]catenane diastereomers under thermodynamic control, the multiple elements of covalent stereochemistry used to direct the stereochemical outcome of the reaction remained in the final product, whereas our chiral auxiliary approach gives access to molecules in which the mechanical bond provides the sole fixed stereogenic unit.[41] Our chiral auxiliary concept is technically simple and, given the generality of the AT-CuAAC catenane-forming reaction,[31] makes functionalized topologically chiral catenanes in which the mechanical bond provides the only stereogenic unit available for the first time without the need for CSP-HPLC separation.

DATA AND SOFTWARE AVAILABILITY

The accession number for the solid-state structure of (R,S_{mt})-**3b** reported in this paper is CCDC: 1885204. Processed compound characterization data (NMR, circular dichroism, HPLC, and MS) are available freely from the University of Southampton repository (https://doi.org/10.5258/SOTON/D0828).

SUPPLEMENTAL INFORMATION

Supplemental Information can be found online at https://doi.org/10.1016/j.chempr.2019.03.008.

ACKNOWLEDGMENTS

S.M.G thanks the European Research Council (Consolidator Grant agreement no. 724987) and Leverhulme Trust (ORPG-2733) for funding. M.D. and F.M. thank the

Engineering and Physical Sciences Research Council for doctoral prize funding (EP/N509747/1 and EP/R513325/1, respectively). J.E.M.L. thanks the European Union's Horizon 2020 Research and Innovation Programme for a Marie Skłodowska-Curie Fellowship (grant agreement no. 660731). The authors thank Reach Separations for assistance with analytical CSP-HPLC.

AUTHOR CONTRIBUTIONS

S.M.G. conceived the project and secured project funding. M.D., J.E.M.L., and F.M. contributed equally to the design of experiments and methodology and their execution. S.M.G. wrote the manuscript with input from all authors. M.D., J.E.M.L., and F.M. contributed equally to the reviewing and editing of the manuscript.

DECLARATION OF INTERESTS

The authors declare no competing interests.

Received: December 15, 2018
Revised: January 14, 2019
Accepted: March 15, 2019
Published: April 11, 2019

REFERENCES AND NOTES

1. 1,126 of 11,712 articles published in the *Journal of the American Chemical Society, Angewandte Chemie, Chemical Science, Chemical Communications, and Chemistry: A European Journal* in 2017 referred to "chiral" or "enantio" in the title, abstract, or keywords (source: Scopus).

2. Pasteur, L. (1848). Mémoire sur la relation qui peut exister entre la forme cristalline et la composition chimique, et sur la cause de la polarisation rotatoire. C. R. Séances Acad. Sci. *26*, 535–538.

3. LeBel, J.A. (1874). Sur les relations qui existent entre les formules atomiques des corps organiques et le pouvoir rotatoire de leurs dissolutions. Bull. Soc. Chim. Fr. *22*, 337–347.

4. van 't Hoff, J.H. (1874). Sur les formules de structure dans l'espace. Arch. Neerl. 1–10.

5. It should be noted that the presence of a stereogenic unit is a necessary but not sufficient condition because the appearance of chirality as molecular asymmetry is a whole molecule property: Mislow, K., and Siegel, J. (1984). Stereoisomerism and local chirality J. Am. Chem. Soc. *106*, 3319–3328.

6. Eliel, E., Wilen, S., and Mander, L. (1994). Stereochemistry of Organic Compounds (John Wiley and Sons, Inc.).

7. Stoddard, J.F. (2017). Mechanically interlocked molecules (MIMs)—molecular shuttles, switches, and machines (Nobel lecture). Angew. Chem. Int. Ed. *56*, 11094–11125.

8. Sauvage, J.P. (2017). From chemical topology to molecular machines (Nobel lecture). Angew. Chem. Int. Ed. *56*, 11080–11093.

9. Erbas-Cakmak, S., Leigh, D.A., McTernan, C.T., and Nussbaumer, A.L. (2015). Artificial molecular machines. Chem. Rev. *115*, 10081–10206.

10. Jamieson, E.M.G., Modicom, F., and Goldup, S.M. (2018). Chirality in rotaxanes and catenanes. Chem. Soc. Rev. *47*, 5266–5311.

11. Frisch, H.L., and Wasserman, E. (1961). Chemical topology 1. J. Am. Chem. Soc. *83*, 3789–3795.

12. For a recent review, see: Fielden, S.D.P., Leigh, D.A., and Woltering, S.L. (2017). Molecular knots Angew. Chem. Int. Ed. *56*, 11166–11194.

13. For a recent example see: Zang, H., Miras, H.N., Yan, J., Long, D.L., and Cronin, L. (2012). Assembly and autochirogenesis of a chiral inorganic polythioanion Möbius strip via symmetry breaking J. Am. Chem. Soc. *134*, 11376–11379.

14. Kaida, Y., Okamoto, Y., Chambron, J.-C., Mitchell, D.K., and Sauvage, J.-P. (1993). The separation of optically active copper (I) catenates. Tetrahedron Lett. *34*, 1019–1022.

15. Yamamoto, C., Okamoto, Y., Schmidt, T., Jäger, R., and Vögtle, F. (1997). Enantiomeric resolution of cycloenantiomeric rotaxane, topologically chiral catenane, and pretzel-shaped molecules: observation of pronounced circular dichroism. J. Am. Chem. Soc. *119*, 10547–10548.

16. Blanco, V., Leigh, D.A., Marcos, V., Morales-Serna, J.A., and Nussbaumer, A.L. (2014). A switchable [2]rotaxane asymmetric organocatalyst that utilizes an acyclic chiral secondary amine. J. Am. Chem. Soc. *136*, 4905–4908.

17. Mitra, R., Zhu, H., Grimme, S., and Niemeyer, J. (2017). Functional mechanically interlocked molecules: asymmetric organocatalysis with a catenated bifunctional Brønsted acid. Angew. Chem. Int. Ed. *56*, 11456–11459.

18. Lim, J.Y.C.C., Marques, I., Félix, V., and Beer, P.D. (2017). A chiral halogen bonding [3] rotaxane for recognition and sensing of biologically relevant dicarboxylate anions. Angew. Chem. Int. Ed. *57*, 584–588.

19. Inouye, M., Hayashi, K., Yonenaga, Y., Itou, T., Fujimoto, K., Uchida, T., Iwamura, M., and Nozaki, K. (2014). A doubly alkynylpyrene-threaded [4]rotaxane that exhibits strong circularly polarized luminescence from the spatially restricted excimer. Angew. Chem. Int. Ed. *53*, 14392–14396.

20. Ishiwari, F., Nakazono, K., Koyama, Y., and Takata, T. (2017). Induction of single-handed helicity of polyacetylenes using mechanically chiral rotaxanes as chiral sources. Angew. Chem. Int. Ed. *56*, 14858–14862.

21. Cakmak, Y., Erbas-Cakmak, S., and Leigh, D.A. (2016). Asymmetric catalysis with a mechanically point-chiral rotaxane. J. Am. Chem. Soc. *138*, 1749–1751.

22. Bordoli, R.J., and Goldup, S.M. (2014). An efficient approach to mechanically planar chiral rotaxanes. J. Am. Chem. Soc. *136*, 4817–4820.

23. Jinks, M.A., de Juan, A., Denis, M., Fletcher, C.J., Galli, M., Jamieson, E.M.G., Modicom, F., Zhang, Z., and Goldup, S.M. (2018). Stereoselective synthesis of mechanically planar chiral rotaxanes. Angew. Chem. Int. Ed. *57*, 14806–14810.

24. This is directly analogous to the same approach in covalent systems: Gnas, Y., and Glorius, F. (2006). Chiral auxiliaries - principles and recent applications Synthesis (Stuttg), 1899–1930.

25. Goldup, S.M., Leigh, D.A., Long, T., McGonigal, P.R., Symes, M.D., and Wu, J. (2009). Active metal template synthesis of [2] catenanes. J. Am. Chem. Soc. *131*, 15924–15929.

26. Sato, Y., Yamasaki, R., and Saito, S. (2009). Synthesis of [2]catenanes by oxidative intramolecular diyne coupling mediated by

macrocyclic copper(I) complexes. Angew. Chem. Int. Ed. *48*, 504–507.

27. Denis, M., and Goldup, S.M. (2017). The active template approach to interlocked molecules. Nat. Rev. Chem. *1*.

28. Tornøe, C.W., Christensen, C., and Meldal, M. (2002). Peptidotriazoles on solid phase: [1,2,3]-triazoles by regiospecific copper(I)-catalyzed 1,3-dipolar cycloadditions of terminal alkynes to azides. J. Org. Chem. *67*, 3057–3064.

29. Rostovtsev, V.V., Green, L.G., Fokin, V.V., and Sharpless, K.B. (2002). A stepwise huisgen cycloaddition process: copper(I)-catalyzed regioselective 'ligation' of azides and terminal alkynes. Angew. Chem. Int. Ed. *41*, 2596–2599.

30. Aucagne, V., Hänni, K.D., Leigh, D.A., Lusby, P.J., and Walker, D.B. (2006). Catalytic "click" rotaxanes: a substoichiometric metal-template pathway to mechanically interlocked architectures. J. Am. Chem. Soc. *128*, 2186–2187.

31. Lewis, J.E.M., Modicom, F., and Goldup, S.M. (2018). Efficient multicomponent active template synthesis of catenanes. J. Am. Chem. Soc. *140*, 4787–4791.

32. Lewis, J.E.M., Bordoli, R.J., Denis, M., Fletcher, C.J., Galli, M., Neal, E.A., Rochette, E.M., and Goldup, S.M. (2016). High yielding synthesis of 2,2′-bipyridine macrocycles, versatile intermediates in the synthesis of rotaxanes. Chem. Sci. *7*, 3154–3161.

33. The conversion of macrocycle **2c** to catenanes **3c** was extremely low, and as a result, it was not possible to isolate the interlocked product. However, ^{1}H NMR analysis of the crude reaction mixture revealed signals consistent with the two diastereomers of catenanes 3c in equal proportions (Figure S80).

34. The origin of the observed stereoselectivity in the case of 3b is unclear at this stage, although preliminary molecular modeling (see Supplemental Information for further details) suggests that, as previously observed,[23] selectivity arises as a result of a biased pre-equilibrium, which in this case is perhaps opposed by the kinetic selectivity of the key bond-forming step. However, given the size of the molecules and the relatively small stereochemical bias observed, significantly more detailed modeling, including the identification of a suitable transition state and the inclusion of explicit solvent, alongside extensive corroborating experimental data, would be required in order to fully elucidate and hopefully optimize the stereoselectivity of the reaction.

35. Attempts to grow single crystals of enantiopure (R,Smt)-**3b** failed. Both enantiomers were observed in the solid-state structure of racemic (R*,S*mt)-**3b** (see Supplemental Information for details; asterisks indicate that the assigned stereochemistry is relative rather than absolute). The 1H NMR spectrum of (R*,S*mt)-**3b** was identical to that of (R,Smt)-**3b**.

36. Lahlali, H., Jobe, K., Watkinson, M., and Goldup, S.M. (2011). Macrocycle size matters: "small" functionalized rotaxanes in excellent yield using the CuAAC active template approach. Angew. Chem. Int. Ed. *50*, 4151–4155.

37. Wuts, P.G.M., and Greene, T.W. (2006). Greene's Protective Groups in Organic Synthesis (John Wiley & Sons, Inc.).

38. Mehmandoust, M., Marazano, C., and Das, B.C. (1989). A stereoselective route to enantiomeric 2-alkyl-1,2,3,6-tetrahydropyridines. J. Chem. Soc. Chem. Commun. 1185.

39. Hodge, J.E. (1955). The Amadori rearrangement. Adv. Carbohydr. Chem. *10*, 169–205.

40. Omura, K., and Swern, D. (1978). Oxidation of alcohols by "activated" dimethyl sulfoxide. A preparative, steric and mechanistic study. Tetrahedron *34*, 1651–1660.

41. Although the mechanical stereogenic element is the only remaining fixed source of stereochemistry in **6b**, as with most molecules, multiple sources of dynamic stereochemistry, including the stereogenic sp^3 hybridized N atom, are present, and these can undergo inversion between R and S configurations and conformational stereoisomerism as a result of rotation of C–C bonds, for example, between the pyridine rings and in the alkyl chains of the two macrocycles.

42. Lam, R.T.S., Belenguer, A., Roberts, S.L., Naumann, C., Jarrosson, T., Otto, S., and Sanders, J.K. (2005). Amplification of acetylcholine-binding catenanes from dynamic combinatorial libraries. Science *308*, 667–669.

43. Chung, M.K., White, P.S., Lee, S.J., and Gagné, M.R. (2009). Synthesis of interlocked 56-membered rings by dynamic self-templating. Angew. Chem. Int. Ed. *48*, 8683–8686.

44. Prakasam, T., Lusi, M., Nauha, E., Olsen, J.C., Sy, M., Platas-Iglesias, C., Charbonnière, L.J., and Trabolsi, A. (2015). Dynamic stereoisomerization in inherently chiral bimetallic [2] Catenanes. Chem. Commun. *51*, 5840–5843.

Chem, Volume *5*

Supplemental Information

An Auxiliary Approach for the Stereoselective Synthesis of Topologically Chiral Catenanes

Mathieu Denis, James E.M. Lewis, Florian Modicom, and Stephen M. Goldup

Supplemental Experimental Procedures

1. General Experimental 2
2. Syntheses of macrocycles 2b and 2c 3
S2 3
S4 7
2b 10
S5 13
S6 16
2c 19
3. Syntheses of U-shapes 1 and S11. 23
(*R*)-1 32
(*S*)-1 36
S11 37
4. Syntheses of catenanes 3 and 6 40
(*R*,S_{mt})-3b and (*R*,R_{mt})-3b 44
(*S*,S_{mt})-3b and (*S*,R_{mt})-3b 53
(*R*,*R*/S_{mt})-3c 54
rac-6b 55
(S_{mt})-6b 59
(R_{mt})-6b 60
5. Syntheses of triazole-functionalised macrocycles S12 and S13 61
S12 61
S13 65
6. Chiral Stationary Phase HPLC analysis of catenane 6b, S8 and S9 68
7. Single Crystal X-ray Analysis of Catenane (*S**,R^*_{mt})-3b 71
8. Preliminary Molecular Modelling of the AT-CuAAC Reaction Between (*R*)-1 and 2b ... 74
9. Supplemental References 78

1. General Experimental

Synthesis: Unless otherwise stated, all reagents, including anhydrous solvents, were purchased from commercial sources and used without further purification. All reactions were carried out under an atmosphere of N_2 using anhydrous solvents unless otherwise stated. Petrol refers to the fraction of petroleum ether boiling in the range 40-60 °C. Flash column chromatography was performed using Biotage Isolera-4 automated chromatography system, employing Biotage SNAP or ZIP cartridges. Analytical TLC was performed on precoated silica gel plates (0.25 mm thick, 60F254, Merck, Germany) and observed under UV light.

Analysis: NMR spectra were recorded on Bruker AV400, AV3-400 or AV500, at a constant temperature of 298 K. Chemical shifts are reported in parts per million from low to high field and referenced to residual solvent. ^{13}C data were typically recorded as phased JMOD experiments. Coupling constants (*J*) are reported in Hertz (Hz). Standard abbreviations indicating multiplicity were used as follows: m = multiplet, quint = quintet, q = quartet, t = triplet, d = doublet, s = singlet, app. = apparent, br = broad. Signal assignment was carried out using 2D NMR methods (HSQC, HMBC, COSY, NOESY) where necessary. In the case of some complex multiplets with contributions from more than one signal absolute assignment was not possible. Here indicative either/or assignments are provided. All melting points were determined using a Griffin apparatus. Low resolution mass spectrometry was carried out by the mass spectrometry services at the University of Southampton (Waters TQD mass spectrometer equipped with a triple quadrupole analyser with UHPLC injection [BEH C18 column; MeCN-hexane gradient {0.2% formic acid}]). High resolution mass spectrometry was carried out by the mass spectrometry services at the University of Southampton (MaXis, Bruker Daltonics, with a Time of Flight (TOF) analyser; samples were introduced to the mass spectrometer via a Dionex Ultimate 3000 autosampler and uHPLC pump in a gradient of 20% acetonitrile in hexane to 100% acetonitrile (0.2% formic acid) over 5 min at 0.6 mL min; column: Acquity UPLC BEH C18 (Waters) 1.7 micron 50 × 2.1mm). As accurate mass measurements are of limited value for compounds with Mw >1000 Da, in these cases a graphical comparison of the observed isotope pattern and the predicted isotopic distribution is provided.

The following compounds were synthesized according to literature procedures: S1, S3, macrocycle **2a**,[1] **S7**,[2] **S10**.[3]

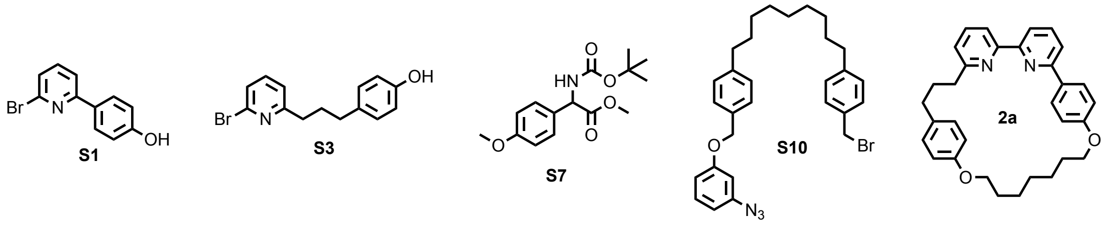

2. Syntheses of macrocycles 2b and 2c

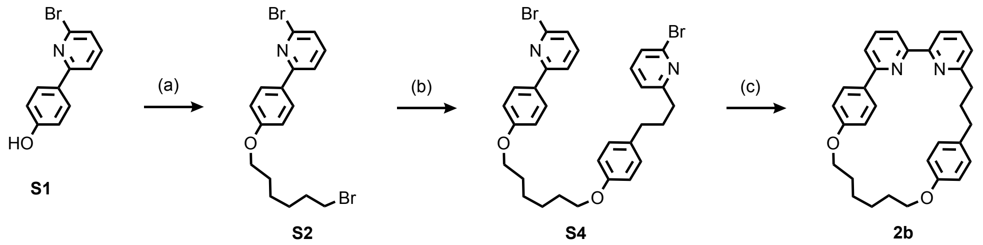

Scheme S1 Synthesis of macrocycle **2b**. Conditions: (a) 1,6-dibromohexane, K_2CO_3, MeCN, reflux, 18 h, 68%; (b) **S3**, K_2CO_3, MeCN, reflux, 86%; (c) $Ni(PPh_3)_2Br_2$, PPh_3, Mn, Et_4NI, DMF/THF, 50 °C, 6 h, 71%.

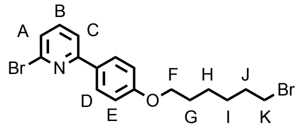

S2

To a solution of **S1** (1 g, 4 mmol, 1 eq.) in MeCN (20 mL) was added K_2CO_3 (2.2 g, 16 mmol, 4 eq.) as a solid. After stirring for 30 minutes, 1,6-dibromohexane (1.53 mL, 2.5 mmol, 2.5 eq.) was added and the reaction was stirred at reflux for 18 h. The cooled reaction mixture was filtered through celite. The solvent was removed *in vacuo*. The residue was purified by column chromatography (Petrol with a gradient from 0 to 50% CH_2Cl_2) to give **S2** as a white solid (1.1 g, 68%). m.p. 94-96 °C.^{1}H NMR (400 MHz, $CDCl_3$) **δ**: 7.94 (d, J = 8.9, 2H, H_D), 7.61 (dd, J = 7.7, 0.9, 1H, H_C), 7.54 (t, J = 7.7, 1H, H_B), 7.34 (dd, J = 7.7, 0.9, 1H, H_A), 6.96 (d, J = 8.9, 2H, H_E), 4.02 (t, J = 6.4, 2H, H_F), 3.43 (t, J = 6.7, 2H, H_K), 1.97-1.87 (m, 2H, H_J), 1.87-1.77 (m, 2H, H_G), 1.60-1.45 (m, 4H, H_H, H_I). ^{13}C NMR (101 MHz, $CDCl_3$) **δ**: 160.6, 158.4, 142.2, 139.0, 130.3, 128.5, 125.6, 118.2, 114.8, 68.0, 33.9, 32.8, 29.2, 28.1, 25.4. HR-ESI-MS m/z = 411.9910 $[M+H]^+$ (calc. for $C_{17}H_{20}Br_2NO$ 411.9906).

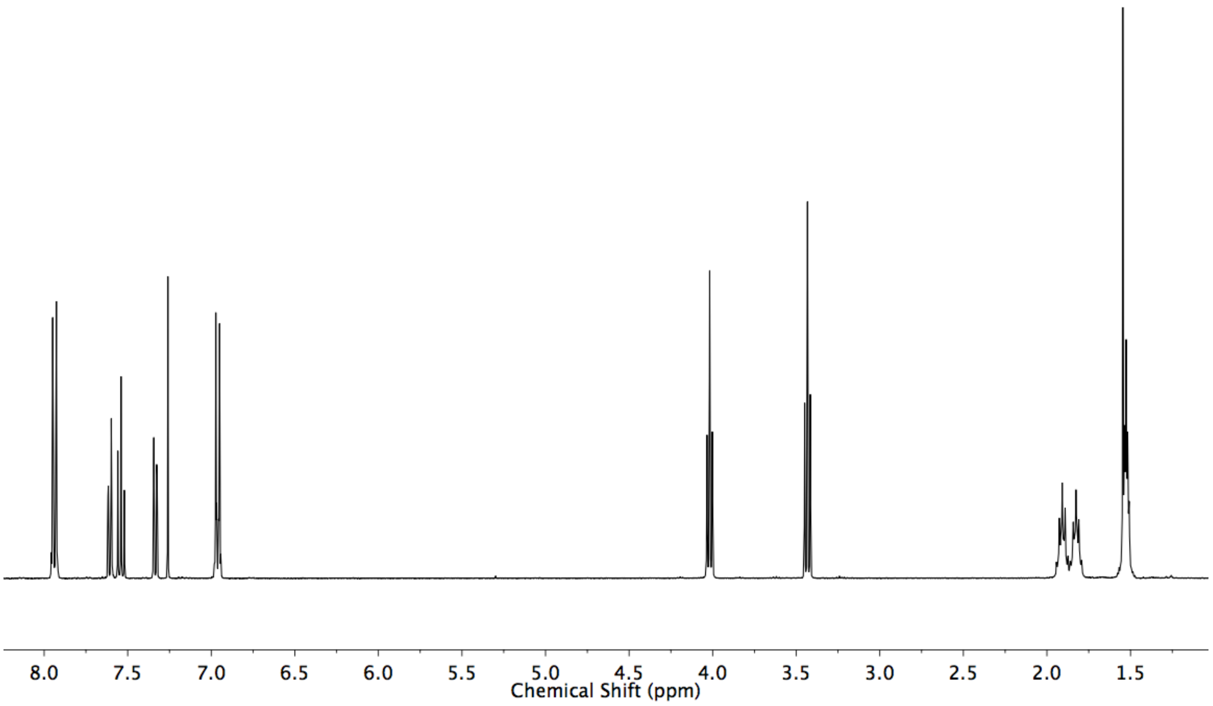

Figure S1 ^{1}H NMR (400 MHz, $CDCl_3$) of **S2**.

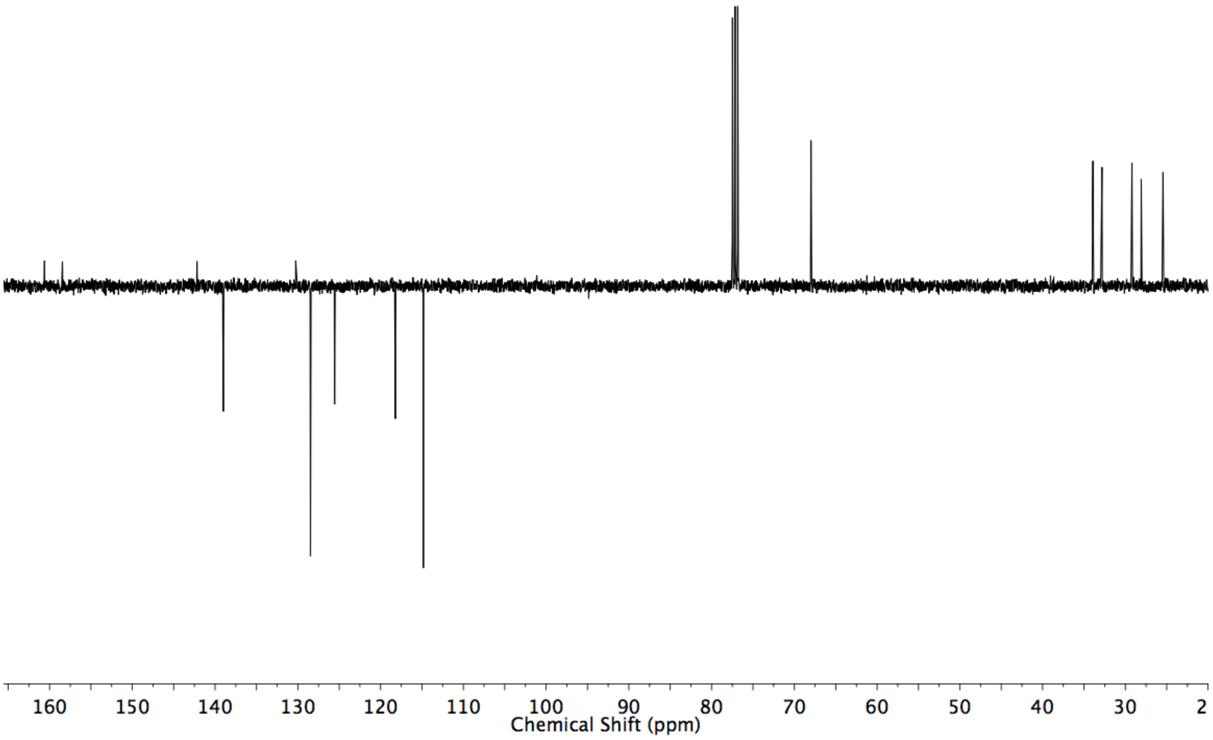

Figure S2 JMOD NMR (101 MHz, $CDCl_3$) of **S2**.

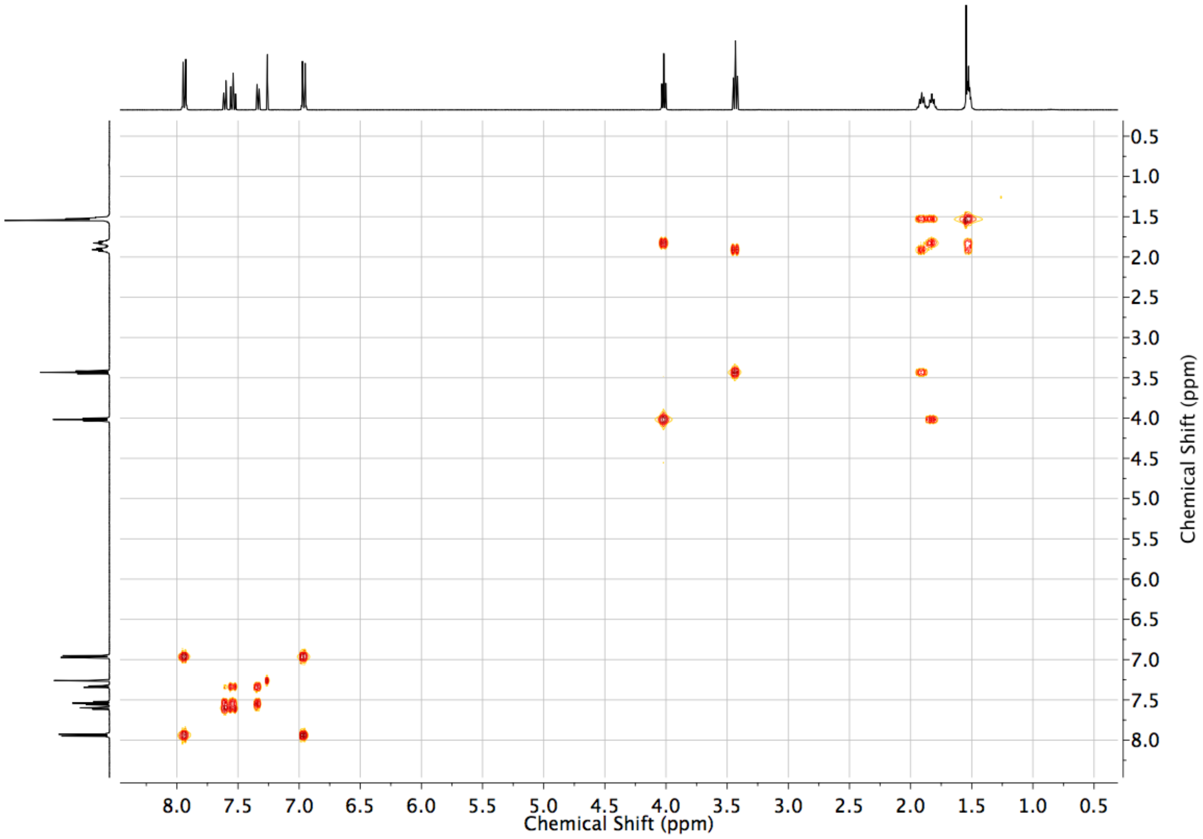

Figure S3 COSY NMR ($CDCl_3$) of **S2**.

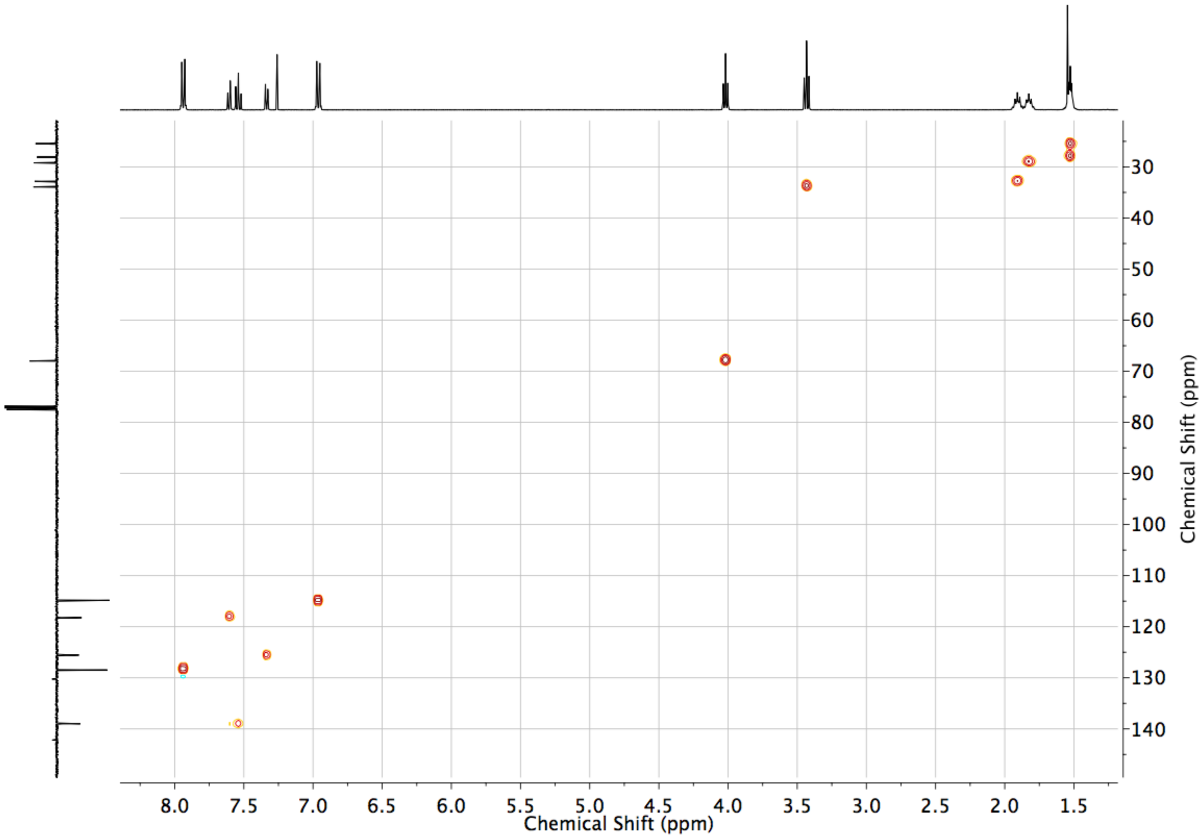

Figure S4 HSQC NMR ($CDCl_3$) of **S2**.

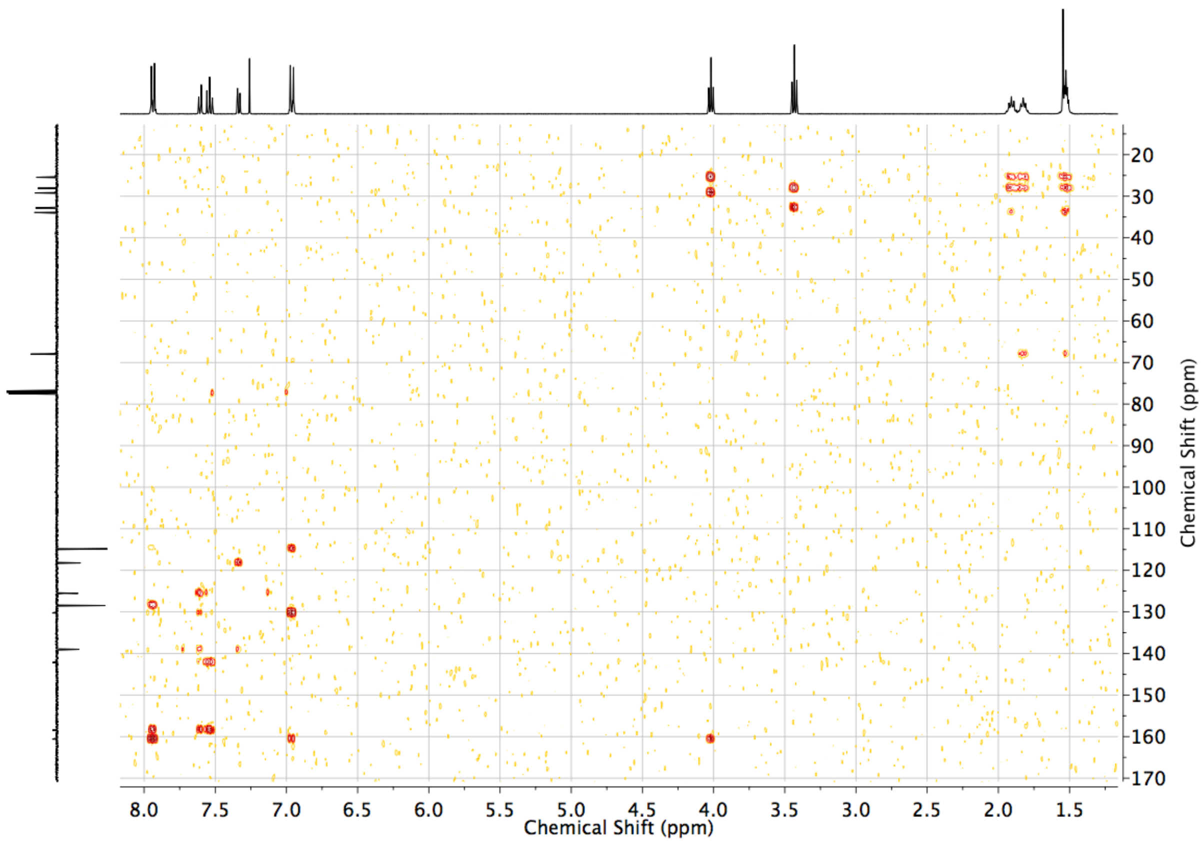

Figure S5 HMBC NMR ($CDCl_3$) of **S2**.

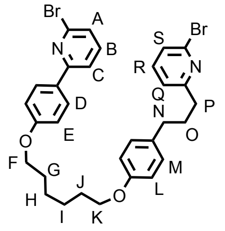

S4

To a solution of **S3** (4.5 g, 15.3 mmol, 1.2 eq.) in MeCN (70 mL) was added K_2CO_3 (2.76 g, 20.0 mmol, 1.7 eq.) as a solid. After stirring for 30 minutes, **S2** (5.0 g, 12.1 mmol, 1.0 eq.) was added as a solid and the reaction stirred at reflux for 18 h. The solvent was removed *in vacuo* and the resultant solid dissolved in CH_2Cl_2 (500 mL), washed with H_2O (100 mL) and brine (100 mL), dried ($MgSO_4$), filtered and the solvent removed *in vacuo*. After purification by column chromatography (Petrol/CH_2Cl_2 1/1 with a gradient to 80% CH_2Cl_2), **S4** (6.5 g, 86%) was obtained as a white solid. m.p. 96-98 °C. ^{1}H NMR (400 MHz, $CDCl_3$) **δ**: 7.94 (d, J = 8.8, 2H, H_D), 7.60 (dd, J = 7.7, 0.9, 1H, H_C), 7.54 (t, J = 7.7, 1H, H_B), 7.43 (t, J = 7.7, 1H, H_R), 7.33 (dd, J = 7.7, 0.9, 1H, H_A), 7.29 (dd, J = 7.7, 0.9, 1H, H_S), 7.13-7.04 (m, 4H, H_M, H_Q), 6.96 (d, J = 8.9, 2H, H_E), 6.82 (d, J = 8.5, 2H, H_L), 4.02 (t, J = 6.5, 2H, H_F), 3.95 (t, J = 6.4, 2H, H_K), 2.78 (t app, J = 7.8, 2H, H_P), 2.61 (t app, J = 7.6, 2H, H_N), 2.07-1.95 (m, 2H, H_O), 1.90-1.76 (m, 4H, H_G, H_J), 1.61-1.50 (m, 4H, H_H, H_I). ^{13}C NMR (101 MHz, $CDCl_3$) **δ**: 164.0, 160.7, 158.5, 157.5, 142.2, 141.7, 139.0, 138.7, 134.0, 130.2, 129.5, 128.5, 125.5, 125.4, 121.6, 118.2, 114.8, 114.6, 68.1, 68.0, 37.6, 34.7, 31.7, 29.4, 29.3, 26.1. HR-ESI-MS m/z = 623.0907 $[M+H]^+$ (calc. for $C_{31}H_{33}Br_2N_2O_2$ 623.0903).

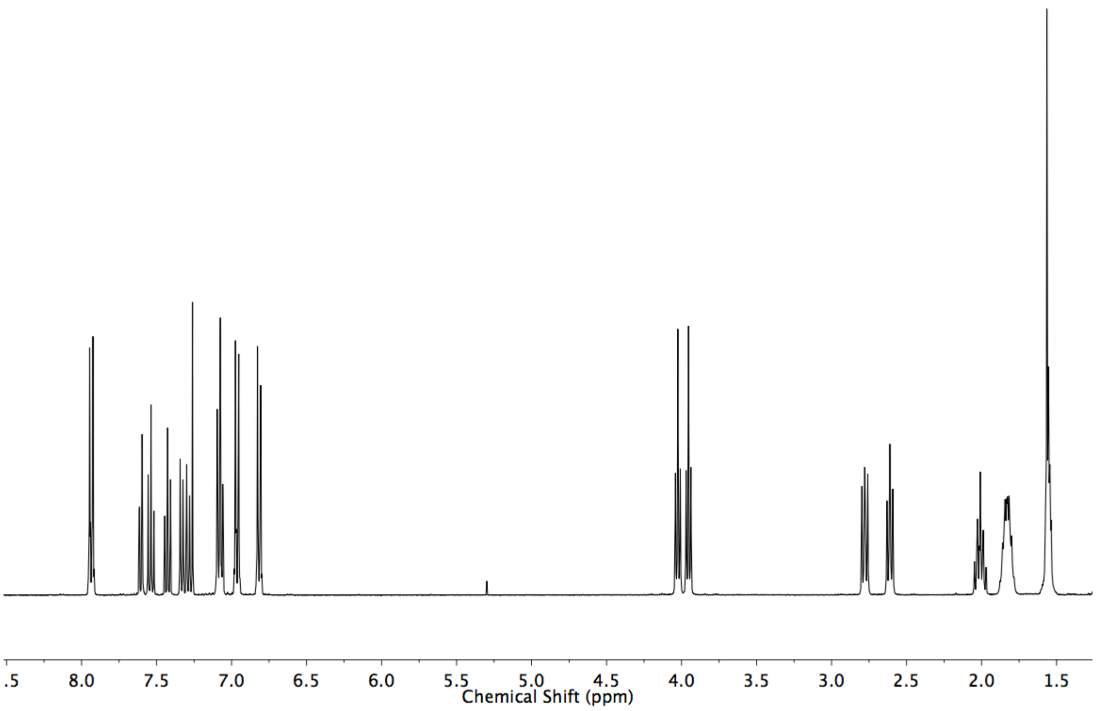

Figure S6 ^{1}H NMR (400 MHz, $CDCl_3$) of **S4**.

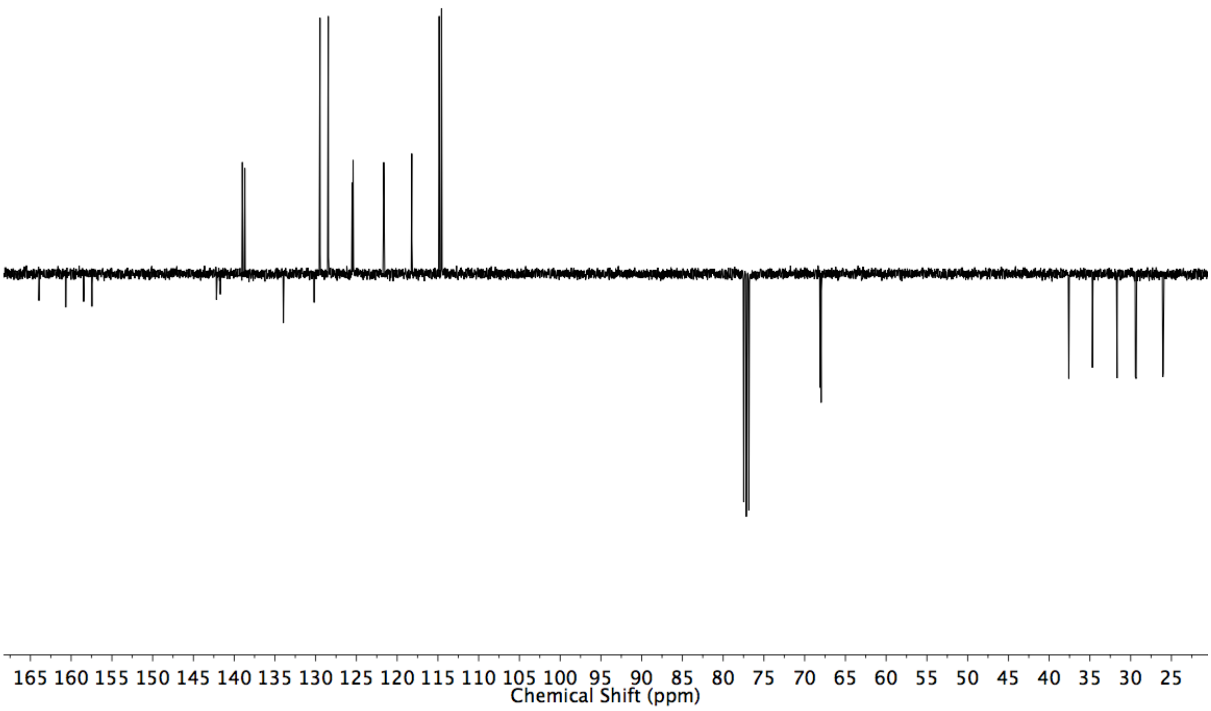

Figure S7 JMOD NMR (101 MHz, $CDCl_3$) of **S4**.

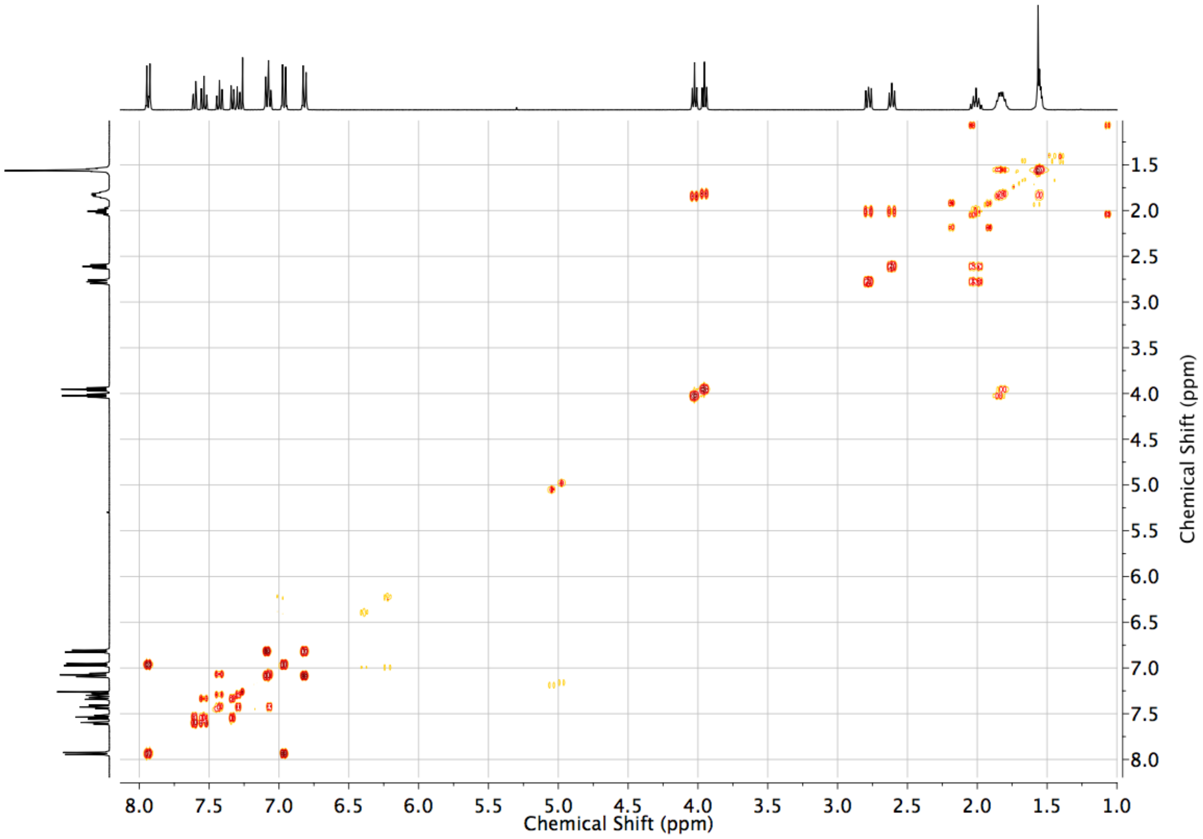

Figure S8 COSY NMR ($CDCl_3$) of **S4**.

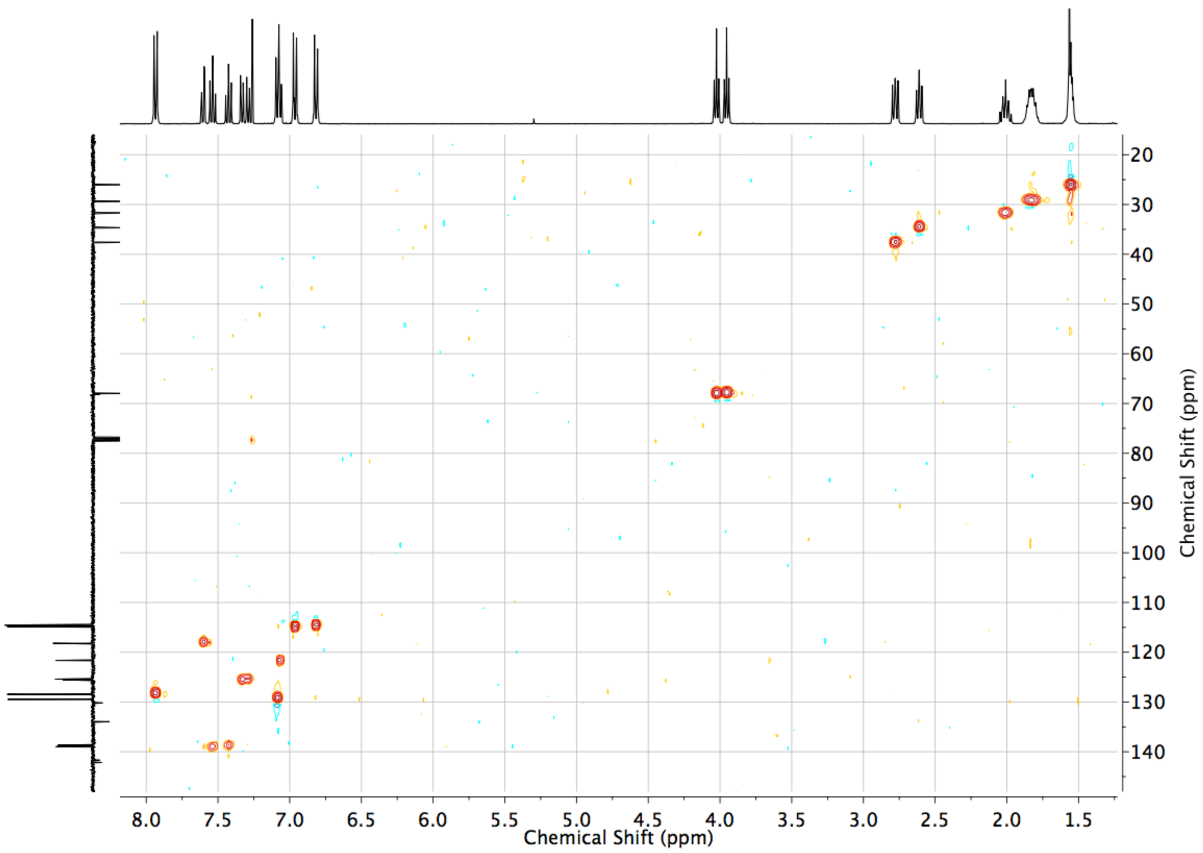

Figure S9 HSQC NMR ($CDCl_3$) of **S4**.

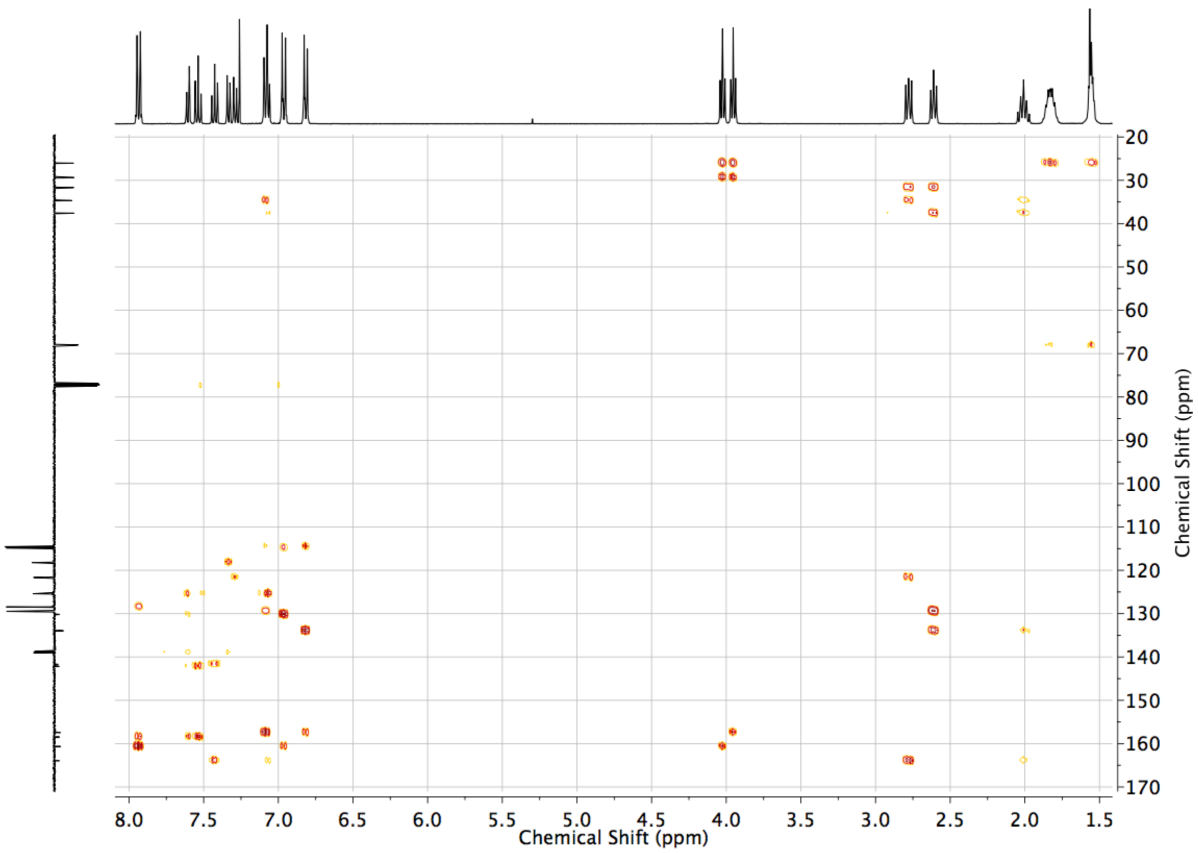

Figure S10 HMBC NMR ($CDCl_3$) of **S4**.

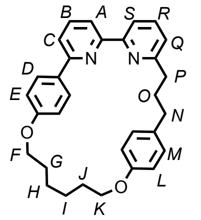

2b

[Ni(PPh_3)$_2$$Br_2$] (1.49 g, 2.00 mmol, 1 eq.), PPh_3 (1.05 g, 4.00 mmol, 2 eq.), Mn (1.10 g, 20.0 mmol, 10 eq.) and NEt_4I (0.514 g, 2.00 mmol, 1 eq.) in DMF (20 mL) were sonicated for 10 min, followed by stirring at 50 °C for 1 h. To this catalyst mixture was added **S4** (1.25 g, 2.00 mmol, 1 eq.) in DMF (20 mL) via syringe over 4 h, followed by additional stirring of the reaction for 1 h. To the cooled reaction was added CH_2Cl_2 (100 mL) and EDTA-NH_3 solution (100 mL). After filtering through a pad of Celite the organic phase was washed with water (2 × 100 mL) and brine (100 mL), and the combined aqueous phases extracted with CH_2Cl_2 (50 mL). The combined organic phases were dried ($MgSO_4$), filtered and the solvent removed *in vacuo*. The crude product was purified by column chromatography (Petrol with a gradient of 0 to 60% $CHCl_3$ + 0.2% EtOH) yielded **2b** as white solid (0.653 g, 71%). m.p. 130-132 °C. ^{1}H NMR (500 MHz, $CDCl_3$) **δ**: 8.08 (d, *J* = 9.0, 2H, H_D), 7.80 (t, *J* = 7.8, 1H, H_B), 7.71 (t, *J* = 7.8, 1H, H_R), 7.70 (dd, *J* = 7.7, 0.9, 1H, H_C), 7.65 (dd, *J* = 7.7, 0.9, 1H, H_A), 7.62 (dd, *J* = 7.9, 0.9, 1H, H_S), 7.31 (d, *J* = 8.7, 2H, H_M), 7.22 (dd, *J* = 7.6, 0.9, 1H, H_Q), 6.85 (d, *J* = 9.0, 2H, H_E), 6.78 (d, *J* = 8.7, 2H, H_L), 4.10 (t, *J* = 6.7, 2H, H_F), 3.98 (t, *J* = 6.1, 2H, H_K), 3.00 (t, *J* = 7.2, 2H, H_P), 2.73 (dd, *J* = 8.8, 6.6, 2H, H_N), 2.41-2.20 (m, 2H, H_O), 1.79 (m, 4H, H_G, H_J), 1.66-1.51 (m, 4H, H_H, H_I). ^{13}C NMR (126 MHz, $CDCl_3$) **δ**: 162.5, 160.0, 157.5, 157.4, 156.7, 156.4, 137.5, 136.9, 135.2, 132.2, 130.1, 128.8, 122.8, 119.5, 119.4, 118.7, 115.3, 114.9, 68.1, 67.7, 37.0, 34.3, 31.4, 28.6, 28.2, 24.8, 24.5. HR-ESI-MS *m/z* = 465.2535 [M+H]$^+$ (calc. for $C_{31}H_{33}N_2O_2$ 465.2537).

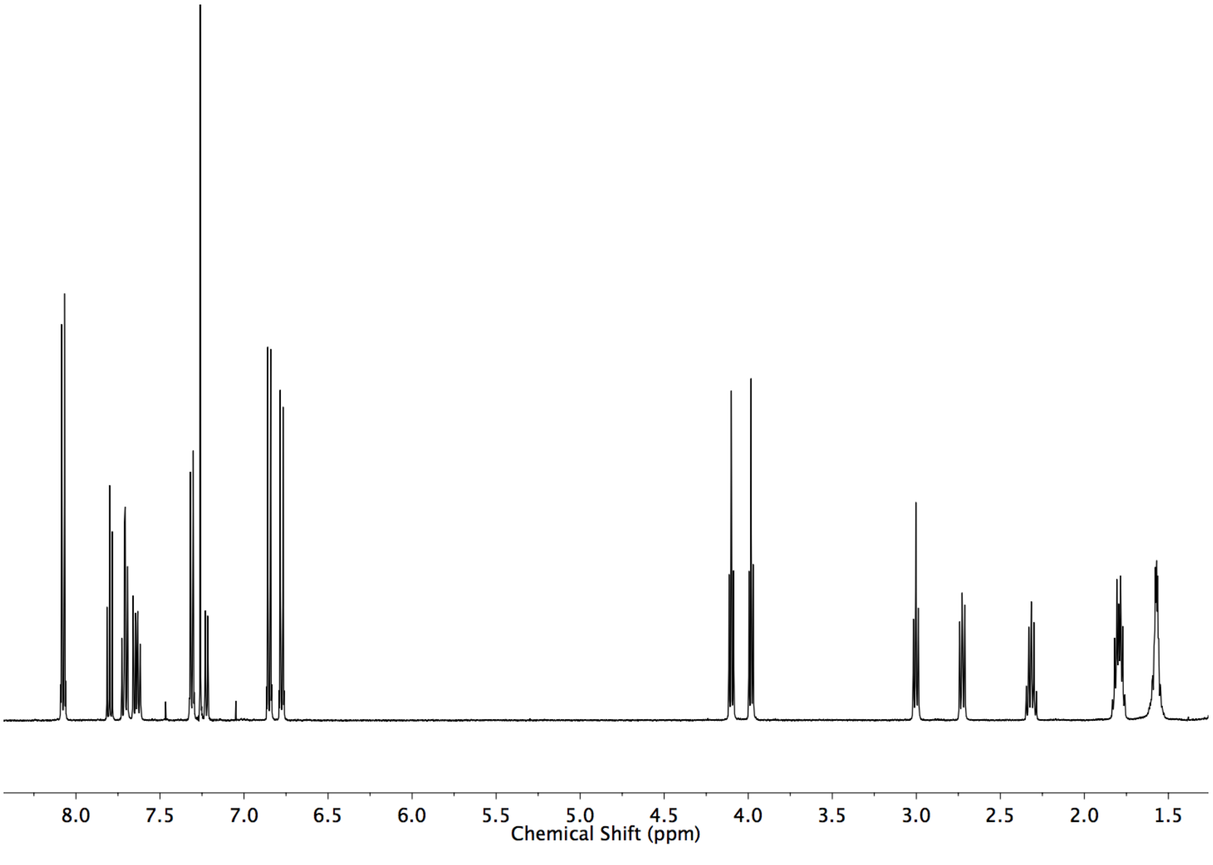

Figure S11 ^{1}H NMR (500 MHz, $CDCl_3$) of **2b**.

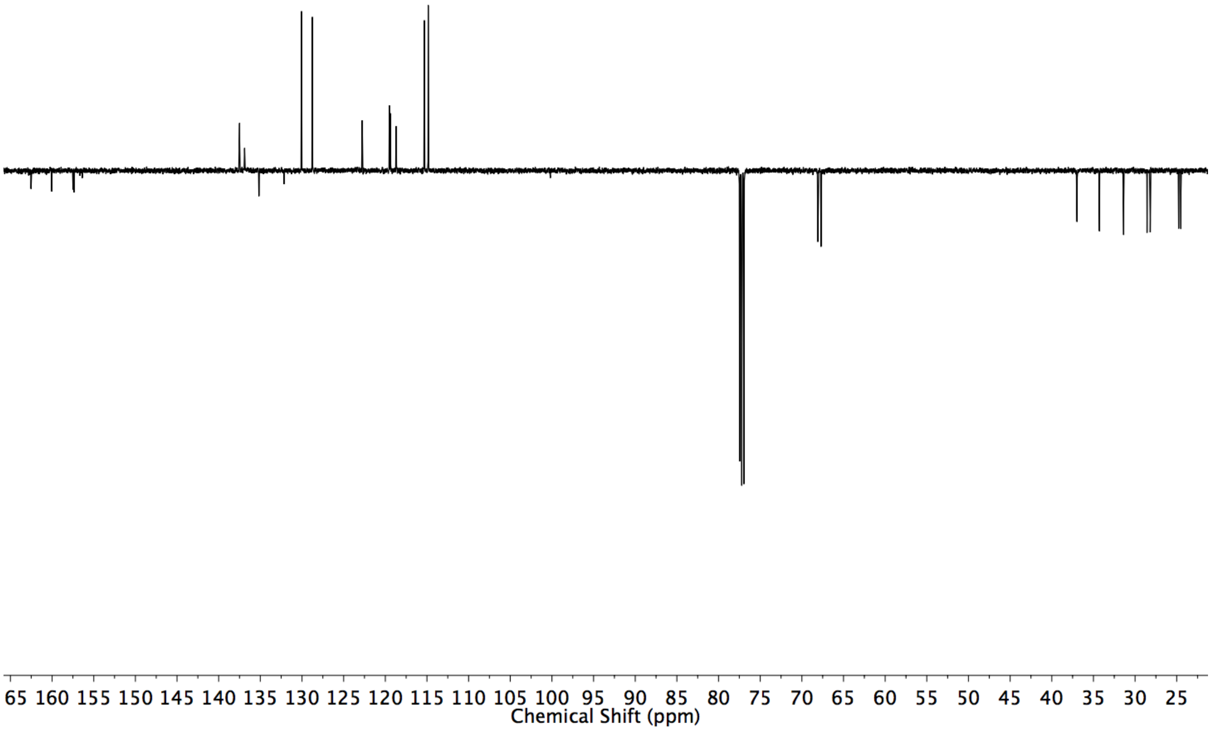

Figure S12 JMOD NMR (126 MHz, $CDCl_3$) of **2b**.

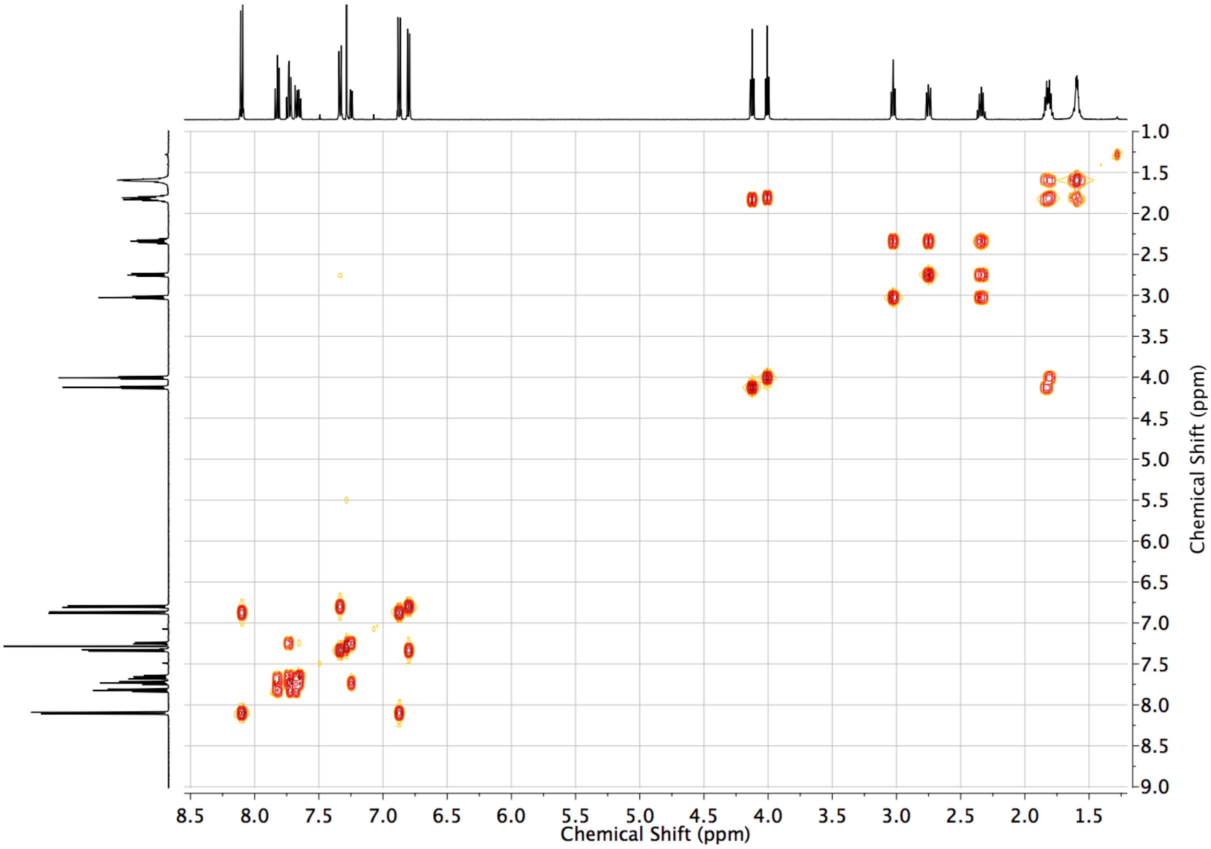

Figure S13 COSY NMR ($CDCl_3$) of **2b**.

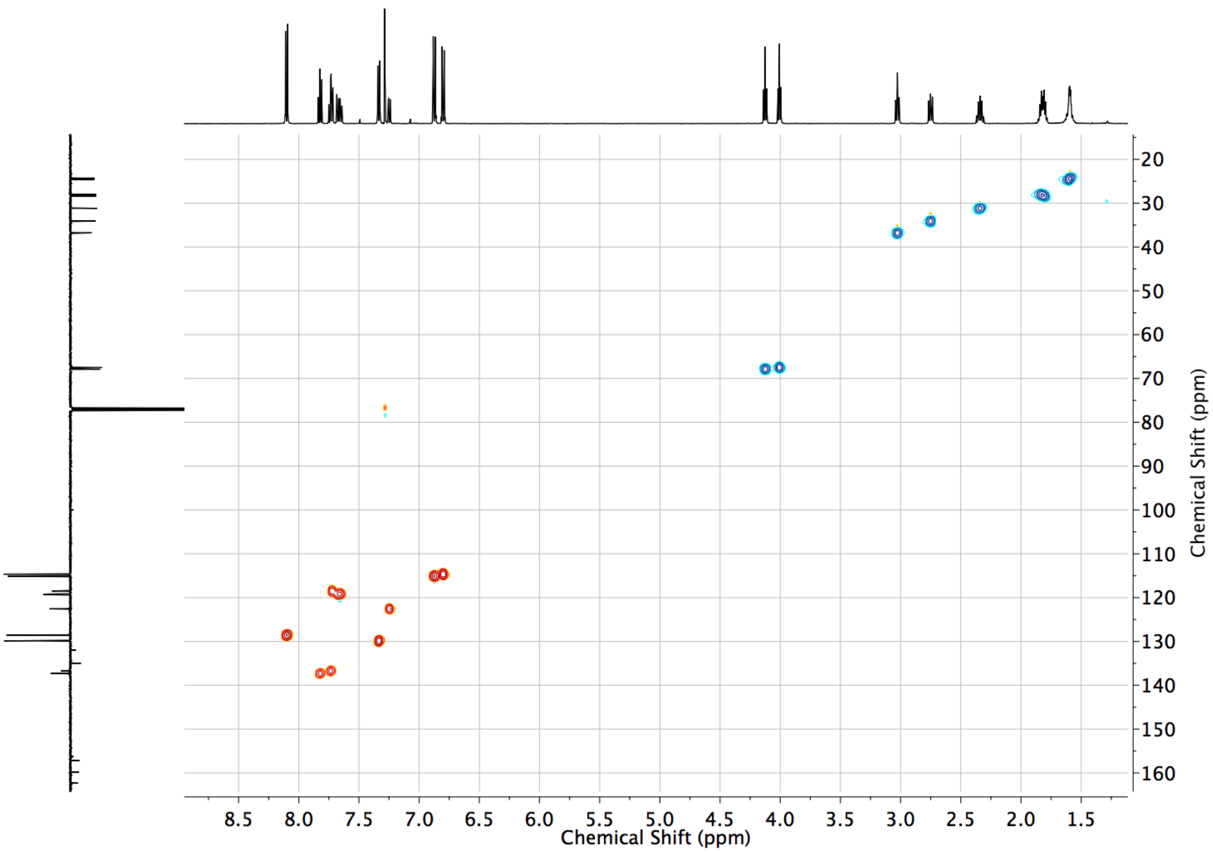

Figure S14 HSQC NMR ($CDCl_3$) of **2b**.

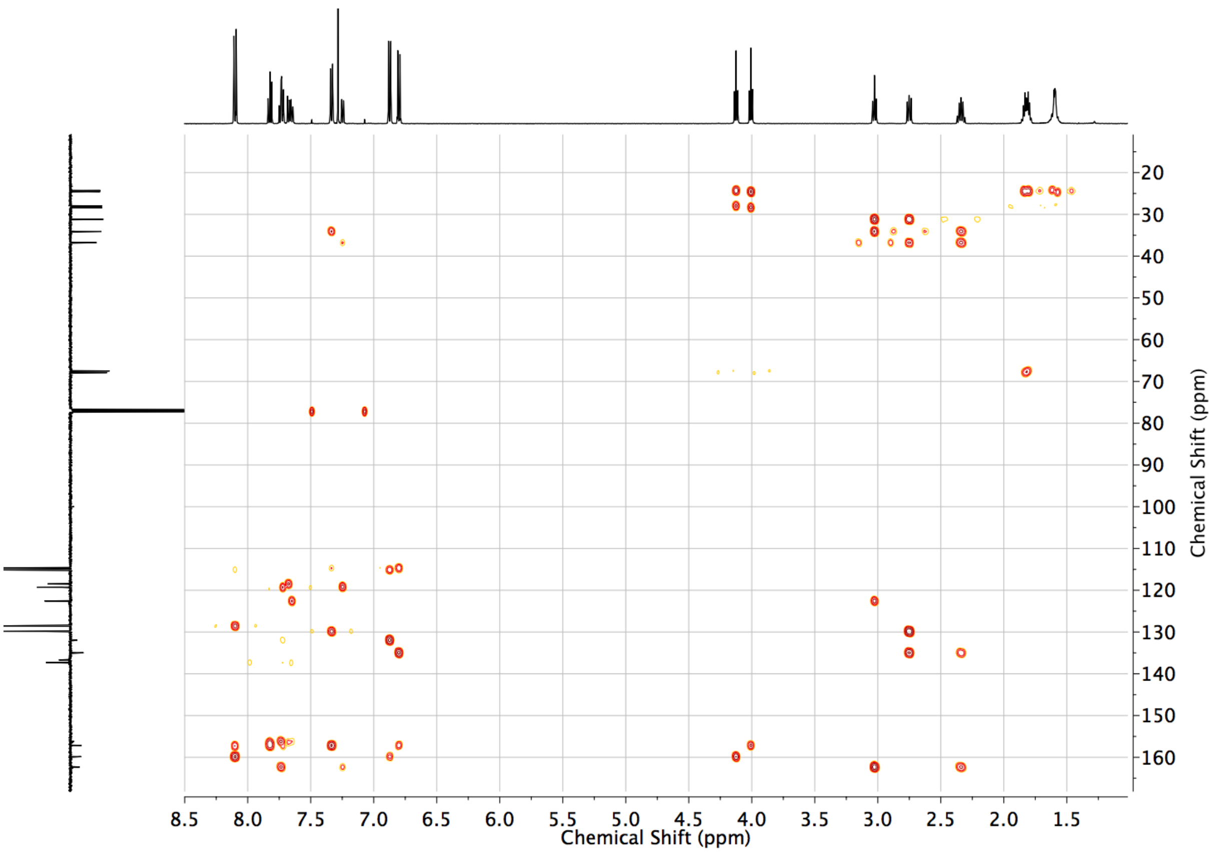

Figure S15 HMBC NMR ($CDCl_3$) of **2b**.

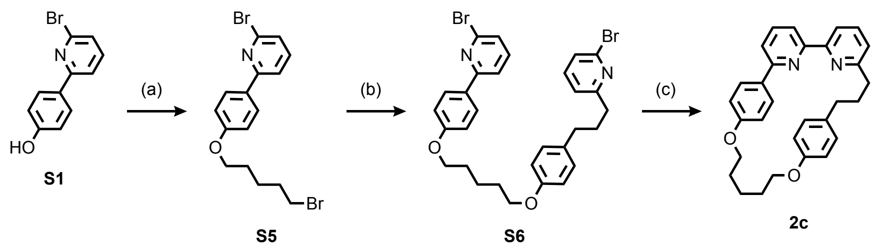

Scheme S2 Synthesis of macrocycle **2c**. Conditions: (a) 1,5-dibromoheptane, K_2CO_3, MeCN, reflux, 18 h, 69%; (b) **S3**, K_2CO_3, MeCN, reflux, 80%; (c) $Ni(PPh_3)_2Br_2$, PPh_3, Mn, Et_4NI, DMF/THF, 50 °C, 6 h, 38%.

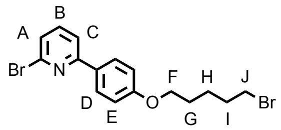

S5

To a solution of **S1** (1 g, 4 mmol, 1 eq.) in MeCN (20 mL) was added K_2CO_3 (2.2 g, 16 mmol, 4 eq.) as a solid. After stirring for 30 minutes, 1,6-dibromopentane (1.35 mL, 2.5 mmol, 2.5 eq.) was added and the reaction was stirred at reflux for 18 h. The cooled reaction mixture was filtered through celite. The solvent was removed *in vacuo*. The residue was purified by column chromatography (Petrol with a gradient from 0 to 50% CH_2Cl_2) to give **S5** as a white solid (1.1 g, 69%). m.p. 70-72 °C ^{1}H NMR (400 MHz, $CDCl_3$) **δ**: 7.94 (d, *J* = 8.9, 2H, H_D), 7.60 (dd, *J* = 7.7, 0.9, 1H, H_C), 7.54 (t, *J* = 7.7, 1H, H_B), 7.33 (dd, *J* = 7.7, 0.9, 1H, H_A), 6.96 (d, *J* = 8.9, 2H, H_E), 4.02 (t, *J* = 6.3, 2H, H_F), 3.45 (t, *J* = 6.7, 2H, H_K), 2.01-1.90 (m, 2H, H_I), 1.89-1.78 (m, 2H, H_G), 1.70-1.60 (m, 2H, H_H). ^{13}C NMR (101 MHz, $CDCl_3$) **δ**: 160.5, 158.4, 142.2, 139.0, 130.3, 128.5, 125.6, 118.2, 114.8, 67.8, 33.7, 32.6, 28.5, 25.0. HR-ESI-MS *m/z* = 397.9752 $[M+H]^+$ (calc. for $C_{16}H_{18}Br_2NO$ 397.9750).

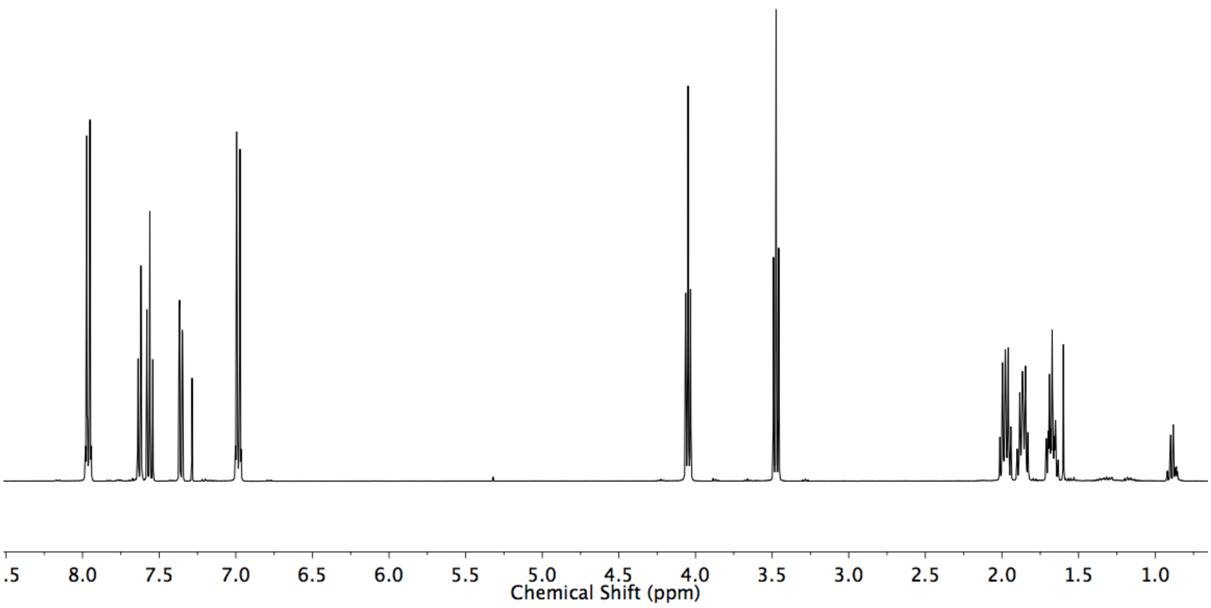

Figure S16 ^{1}H NMR (400 MHz, $CDCl_3$) of **S5**.

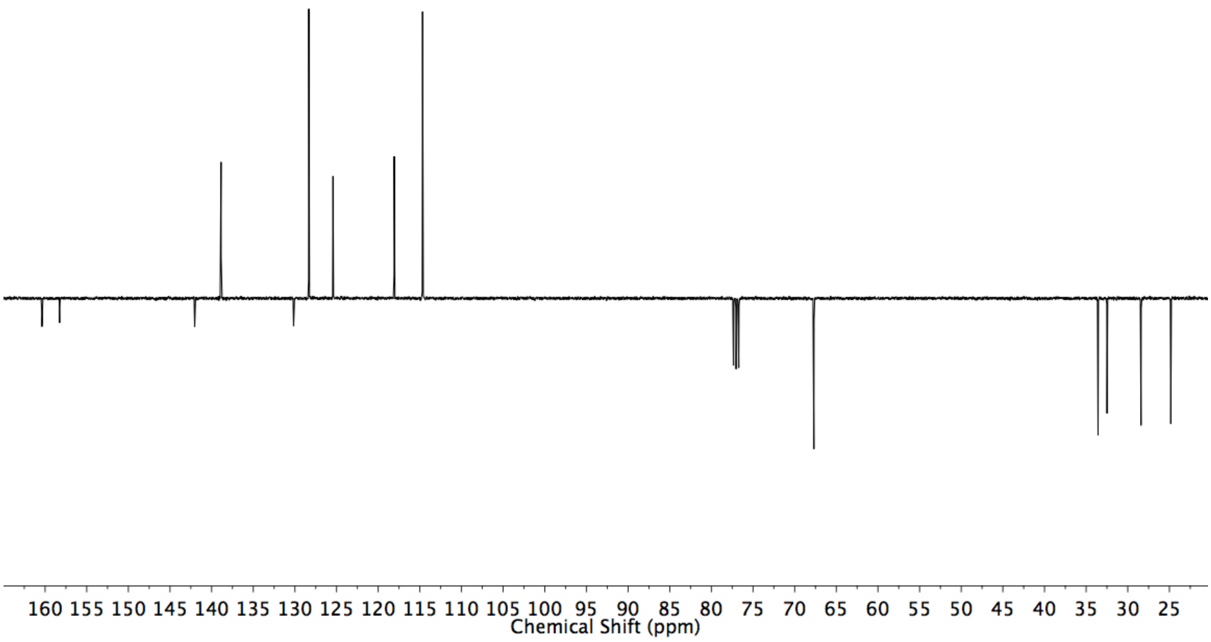

Figure S17 JMOD NMR (101 MHz, $CDCl_3$) of **S5**.

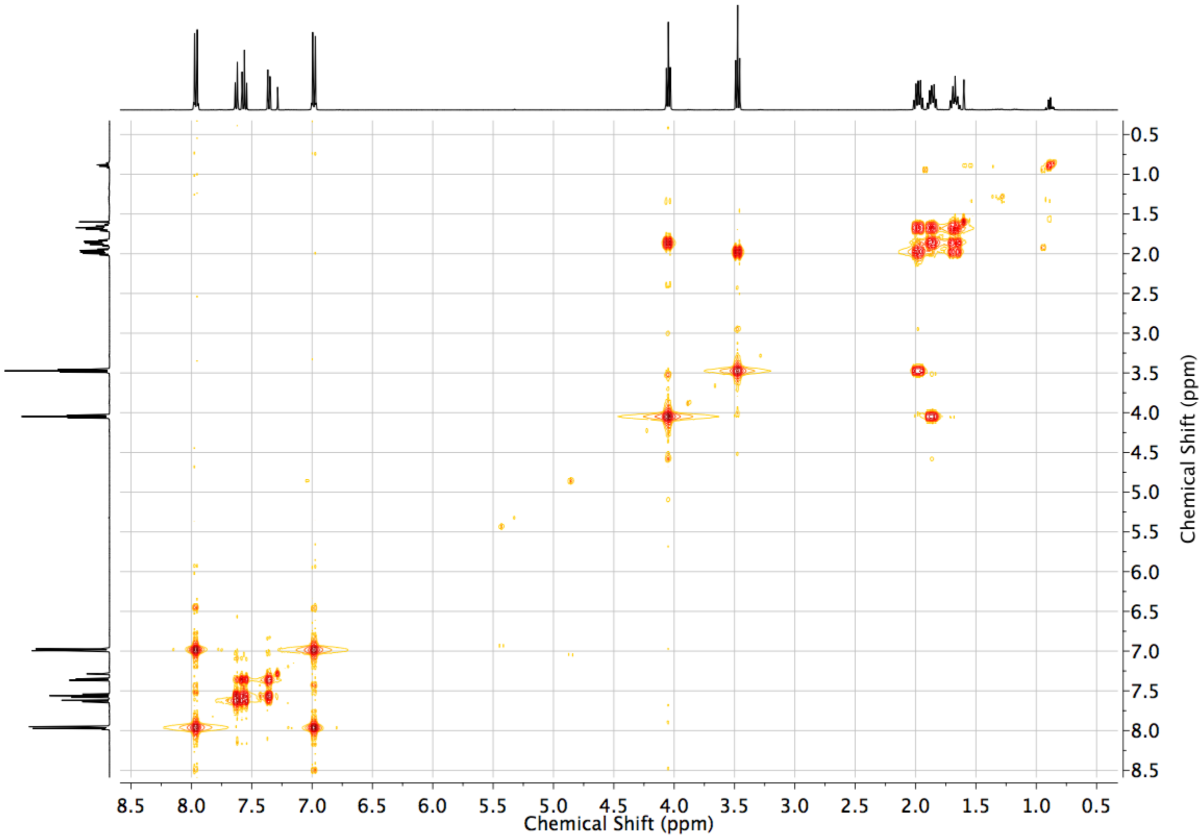

Figure S18 COSY NMR ($CDCl_3$) of **S5**.

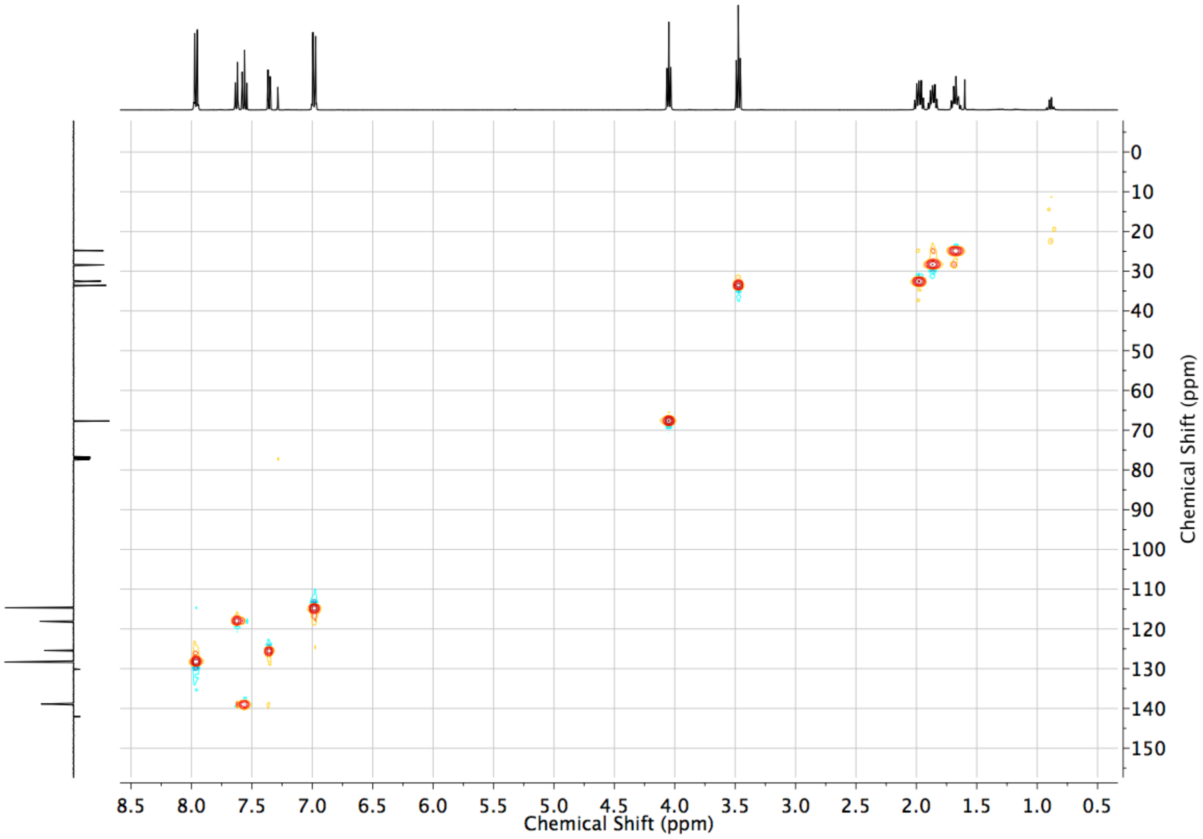

Figure S19 HSQC NMR ($CDCl_3$) of **S5**.

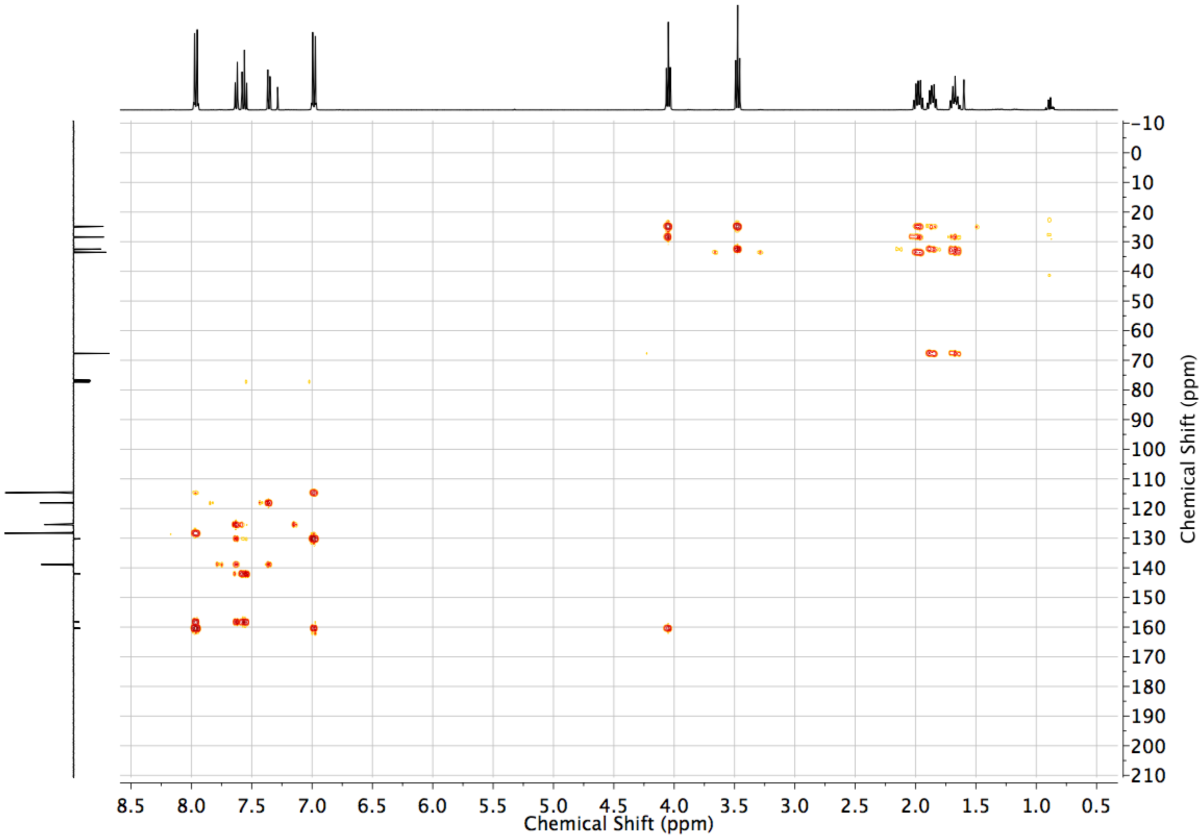

Figure S20 HMBC NMR ($CDCl_3$) of **S5**.

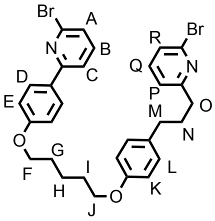

S6

To a solution of **S3** (0.88 g, 3 mmol, 1.2 eq.) in MeCN (10 mL) was added K_2CO_3 (1.38 g, 10 mmol, 4 eq.) as a solid. After stirring for 30 minutes, **S5** (1.0 g, 2.5 mmol, 1.0 eq.) was added as a solid and the reaction stirred at reflux for 18 h. The solvent was removed *in vacuo* and the resultant solid dissolved in CH_2Cl_2 (75 mL), washed with H_2O (20 mL) and brine (20 mL), dried ($MgSO_4$), filtered and the solvent removed *in vacuo*. After purification by column chromatography (Petrol/CH_2Cl_2 1/1 with a gradient to 80% CH_2Cl_2), **S6** (1.4 g, 90%) was obtained as a white solid. m.p. 82-84 °C. ^{1}H NMR (400 MHz, $CDCl_3$) **δ**: 7.93 (d, J = 8.8, 2H, H_D), 7.60 (dd, J = 7.8, 0.9, 1H, H_C), 7.54 (t, J = 7.7, 1H, H_B), 7.43 (t, J = 7.7, 1H, H_Q), 7.33 (dd, J = 7.7, 0.9, 1H, H_A), 7.29 (dd, J = 7.7, 0.9, 1H, H_R), 7.12-7.03 (m, 4H, H_L, H_P), 6.96 (d, J = 8.9, 2H, H_E), 6.82 (d, J = 8.6, 2H, H_K), 4.02 (t, J = 6.4, 2H, H_F), 3.95 (t, J = 6.4, 2H, H_J), 2.78 (t app, J = 7.8, 2H, H_O), 2.61 (t app, J = 7.6, 2H, H_M), 2.07-1.95 (m, 2H, H_N), 1.93-1.81 (m, 4H, H_G, H_I), 1.73-1.62 (m, 2H, H_H). ^{13}C NMR (101 MHz, $CDCl_3$) **δ**: 163.9, 160.6, 158.5, 157.4, 142.2, 141.7, 139.0, 138.7, 134.0, 130.2, 129.5, 128.5, 125.5, 125.4, 121.6, 118.2, 114.8, 114.6, 68.0, 67.9, 37.6, 34.7, 31.7, 29.2, 29.1, 22.9. HR-ESI-MS m/z = 609.0736 $[M+H]^+$ (calc. for $C_{30}H_{31}Br_2N_2O_2$ 609.0747).

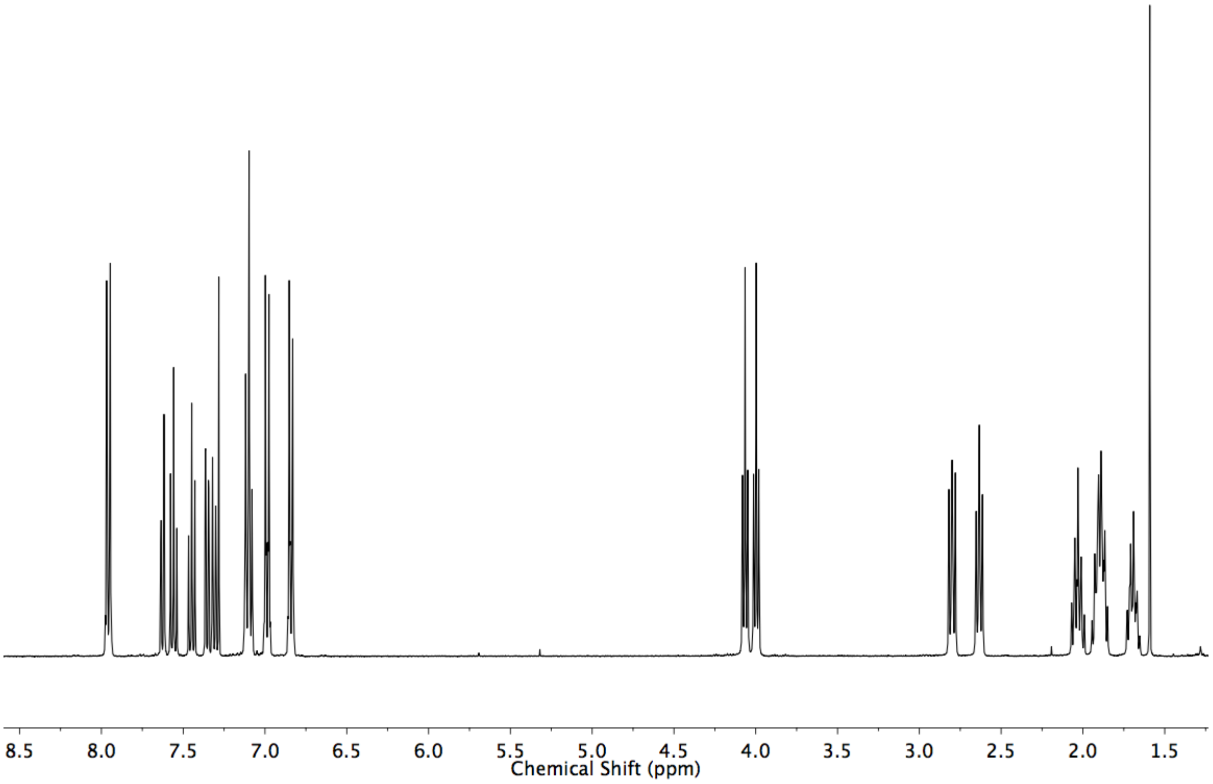

Figure S21 ^{1}H NMR (400 MHz, $CDCl_3$) of **S6**.

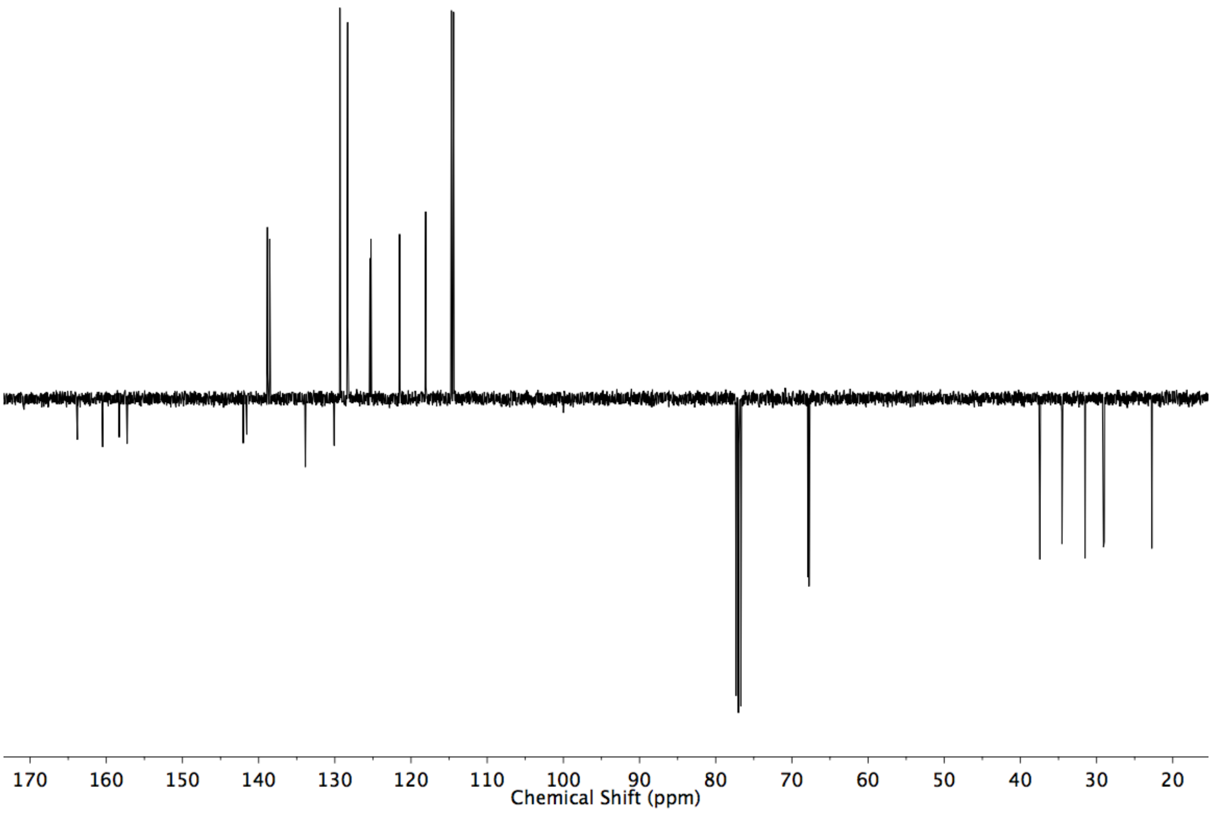

Figure S22 JMOD NMR (101 MHz, $CDCl_3$) of **S6**.

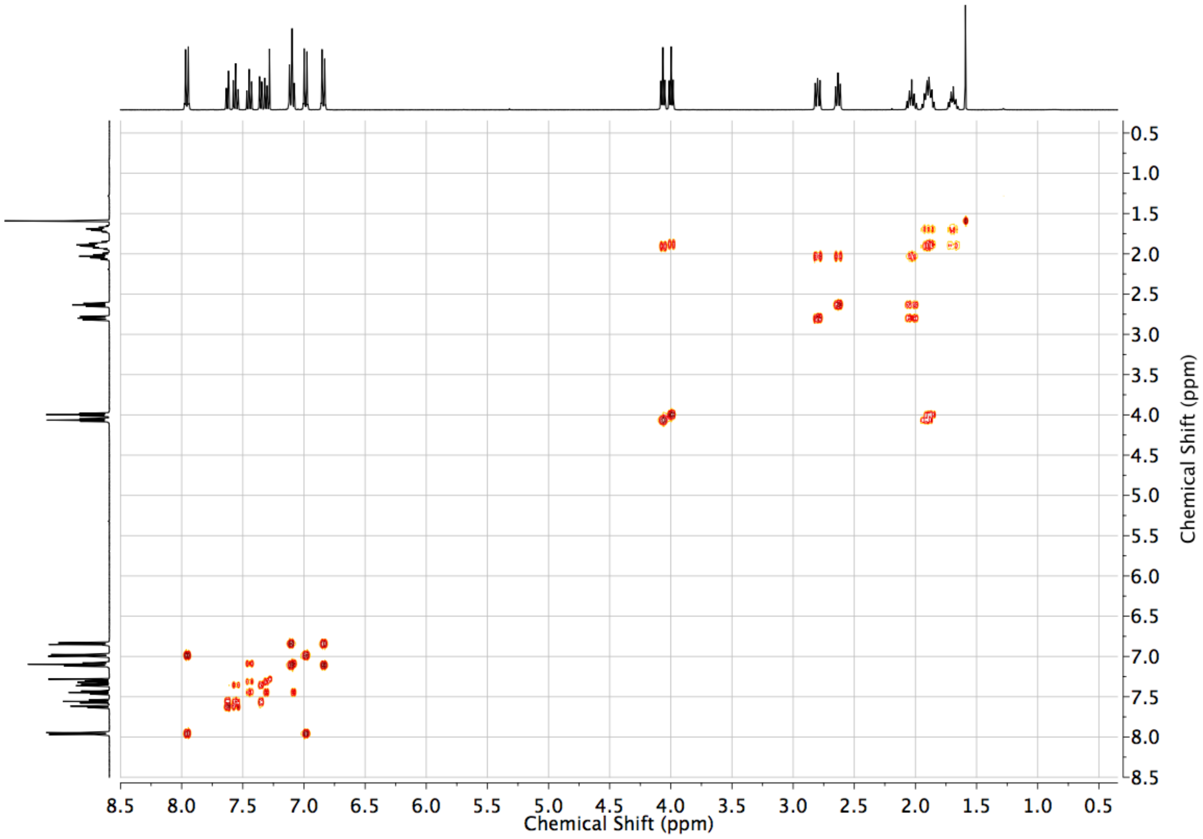

Figure S23 COSY NMR ($CDCl_3$) of **S6**.

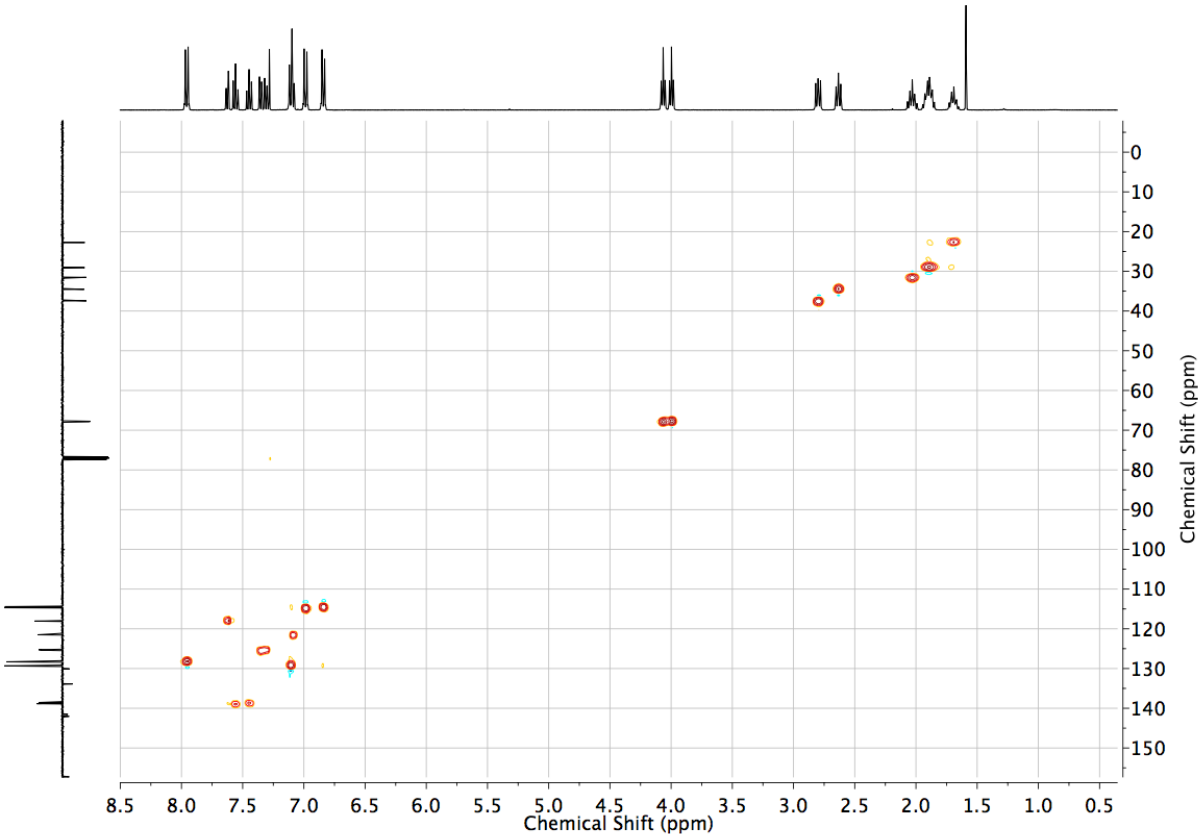

Figure S24 HSQC NMR ($CDCl_3$) of **S6**.

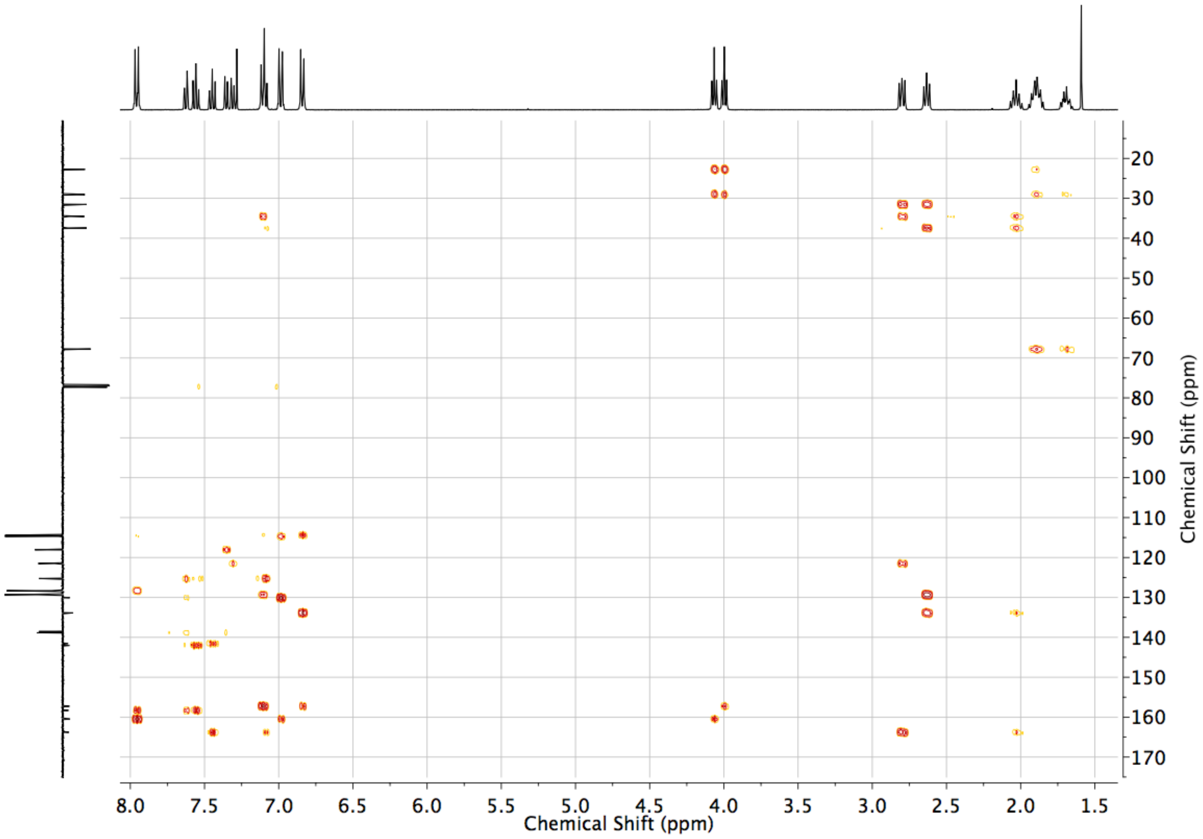

Figure S25 HMBC NMR ($CDCl_3$) of **S6**.

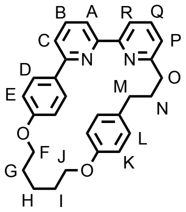

2c

[$Ni(PPh_3)_2Br_2$] (1.49 g, 2.00 mmol, 1 eq.), PPh_3 (1.05 g, 4.00 mmol, 2 eq.), Mn (1.10 g, 20.0 mmol, 10 eq.) and NEt_4I (0.514 g, 2.00 mmol, 1 eq.) in DMF (20 mL) were sonicated for 10 min, followed by stirring at 50 °C for 1 h. To this catalyst mixture was added **S6** (1.22 g, 2.00 mmol, 1 eq.) in DMF (20 mL) via syringe over 4 h, followed by additional stirring of the reaction for 1 h. To the cooled reaction was added CH_2Cl_2 (100 mL) and EDTA-NH_3 solution (100 mL). After filtering through a pad of Celite the organic phase was washed with water (2 × 100 mL) and brine (100 mL), and the combined aqueous phases extracted with CH_2Cl_2 (50 mL). The combined organic phases were dried ($MgSO_4$), filtered and the solvent removed *in vacuo*. The crude product was purified by column chromatography (CH_2Cl_2 with 1% acetone) yielded **2c** as white solid (0.340 g, 38%). m.p. 138-140 °C. ^1H NMR (400 MHz, $CDCl_3$) **δ**: 8.04 (d, J = 8.8, 2H, H_D), 7.79 (t, J = 7.8, 1H, H_B), 7.75-7.67 (m, 2H, H_c, H_Q), 7.70 (dd, J = 7.7, 0.9, 1H, H_S), 7.66 (dd, J = 7.7, 0.9, 1H, H_A), 7.63 (dd, J = 7.8, 1.0, 1H, H_R), 7.30 (d, J = 8.7, 2H, H_L), 7.22 (dd, J = 7.6, 1.0, 1H, H_P), 6.93-6.70 (m, 4H, H_E, H_K), 4.12 (t, J = 7.3, 2H, H_F), 4.03 (t, J = 6.1, 2H, H_J), 3.01 (t, J = 7.3, 2H, H_O), 2.70 (dd, J = 8.8, 6.6, 2H, H_M), 2.39-2.27 (m, 2H, H_N), 1.91-1.78 (m, 4H, H_G, H_I), 1.72-1.62 (m, 2H, H_H). ^{13}C NMR (126 MHz, $CDCl_3$) **δ**: 162.3, 159.5, 157.3, 156.9, 156.3, 156.0, 137.3, 136.7, 135.3, 132.0, 129.9, 128.6, 122.6, 119.2, 119.1, 118.5, 115.3, 114.9, 68.4, 67.3, 36.8, 34.2, 31.2, 27.9, 27.2, 21.3. HR-ESI-MS m/z = 451.2386 $[M+H]^+$ (calc. for $C_{30}H_{31}N_2O_2$ 451.2380).

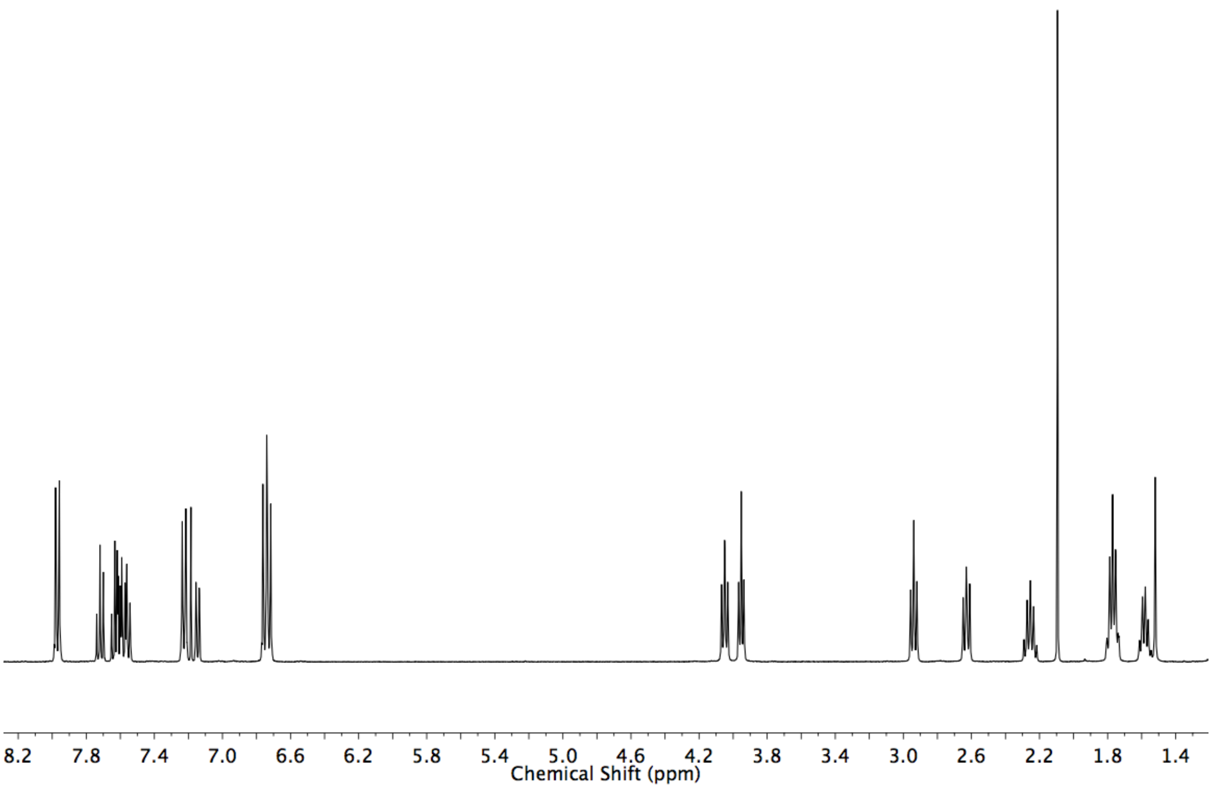

Figure S26 ^{1}H NMR (400 MHz, $CDCl_3$) of **2c**.

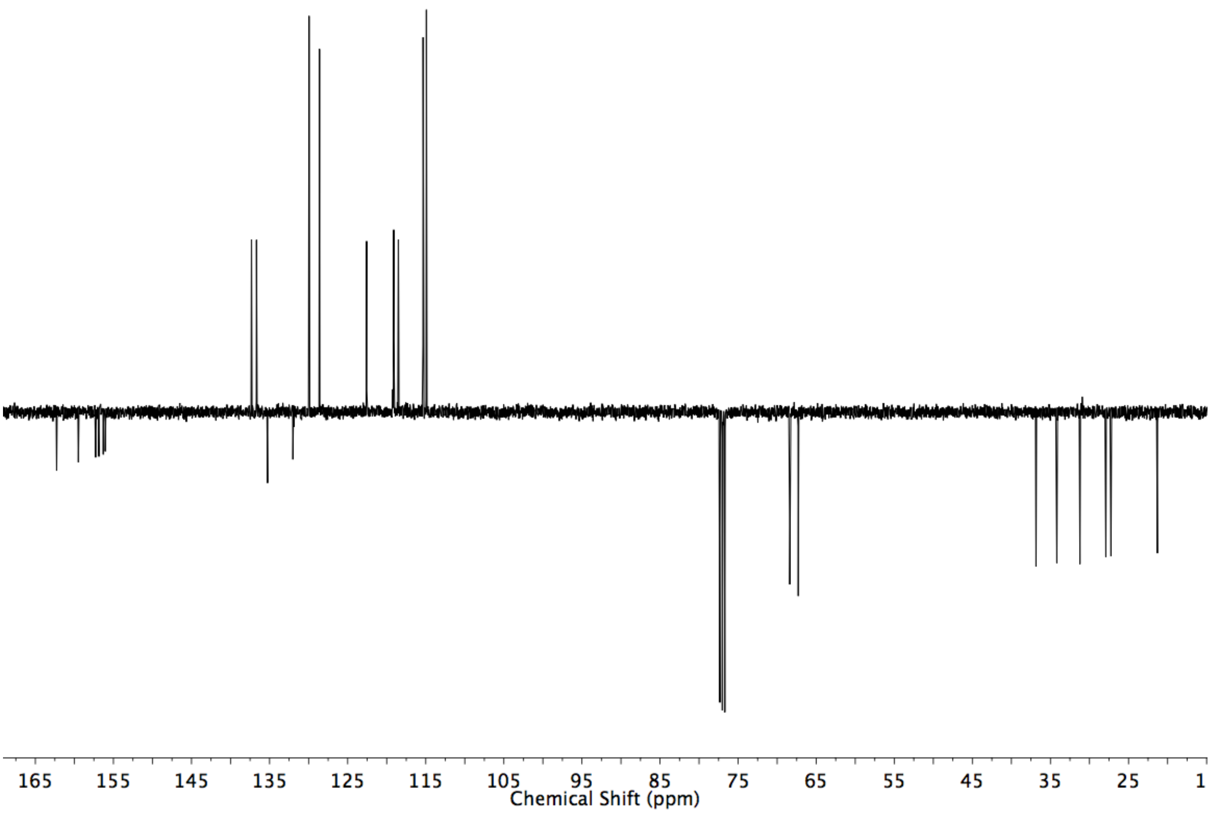

Figure S27 JMOD NMR (101 MHz, $CDCl_3$) of **2c**.

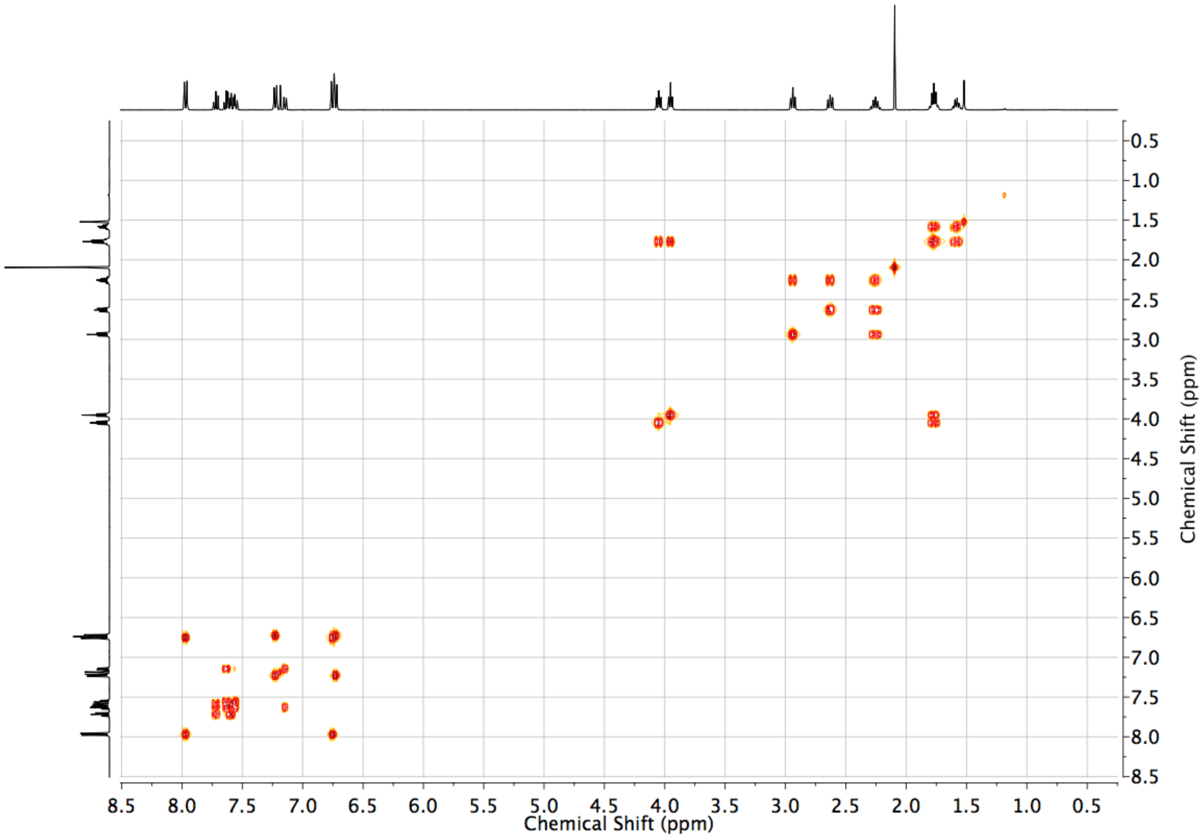

Figure S28 COSY NMR ($CDCl_3$) of **2c**.

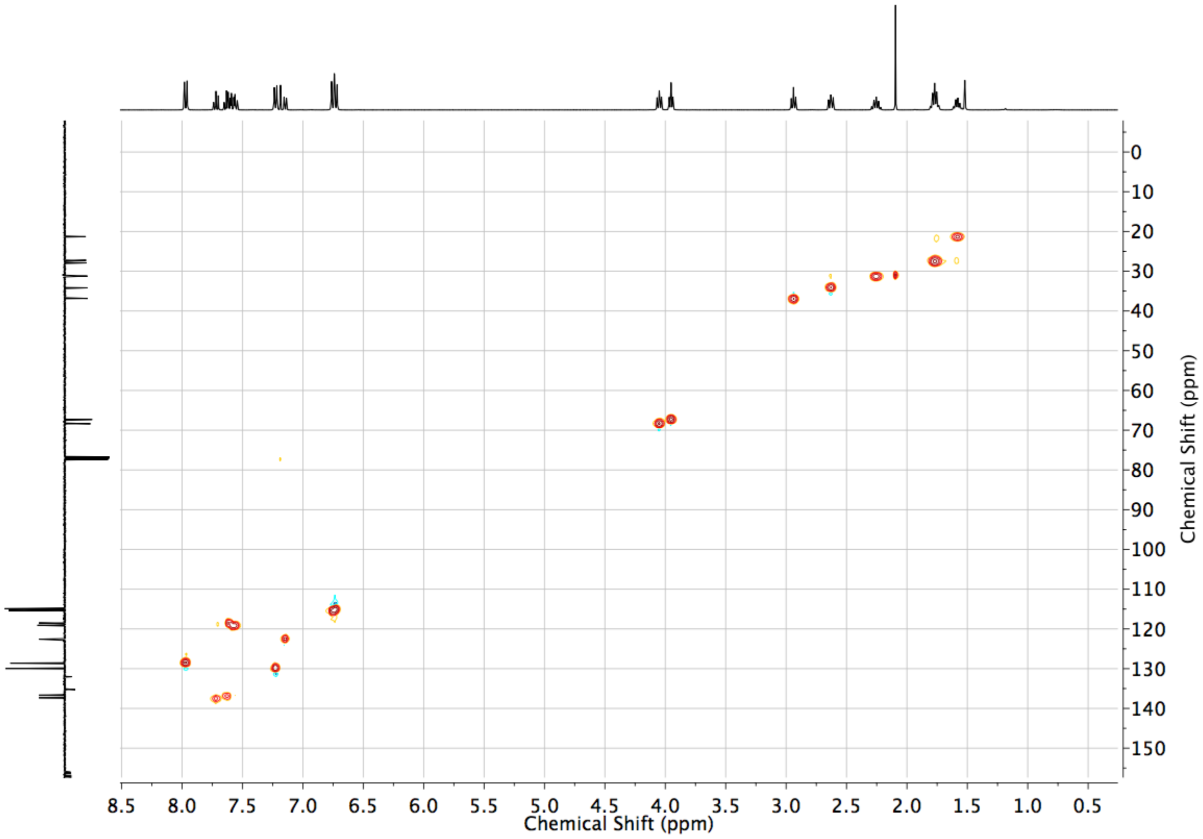

Figure S29 HSQC NMR ($CDCl_3$) of **2c**.

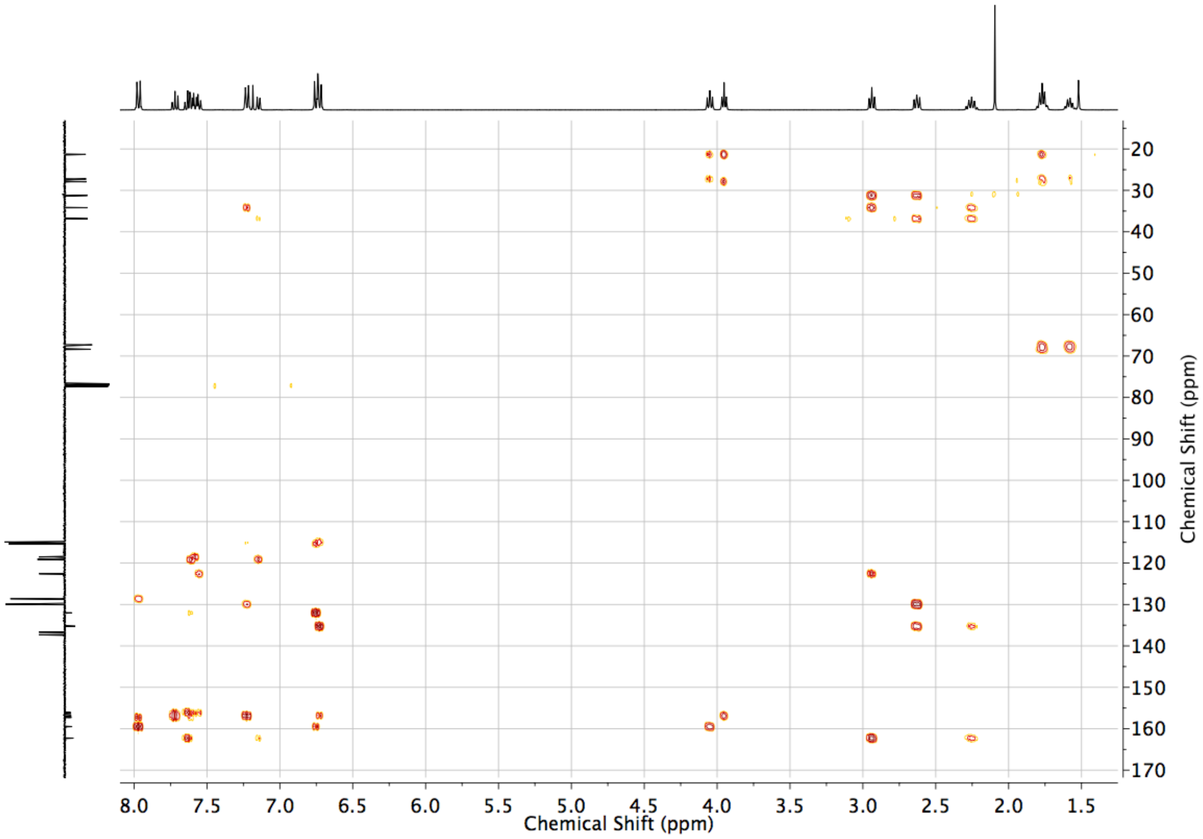

Figure S30 HMBC NMR ($CDCl_3$) of **2c**.

3. Syntheses of U-shapes 1 and S11.

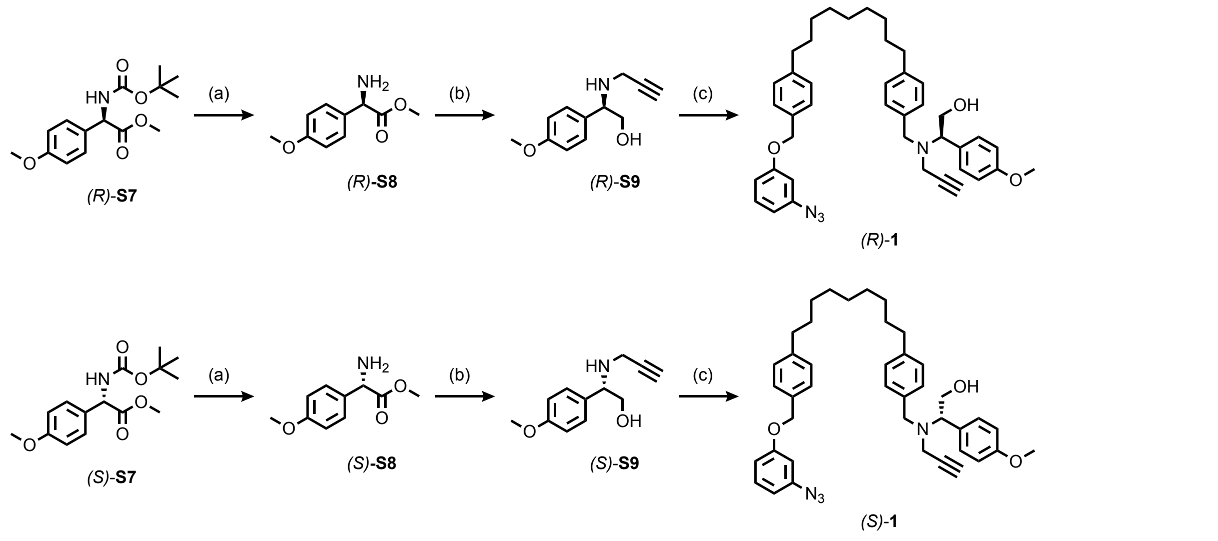

Scheme S3 Syntheses of U-shapes (*R*)-**1** and (*S*)-**1**. Conditions: (a) HCl, dioxane, r.t., 16 h, 26% (*R*) and 60% (*S*) after recrystalisation; (b) $LiAlH_4$, THF, 0 °C to r.t., 2 h, then propargylbromide, K_2CO_3, MeCN, r.t., 16 h, 43% (*R*) and 40% (*S*) over two steps; (c) **S11**, K_2CO_3, MeCN, reflux, 20 h, 99% (*R*) and 90% (*S*).

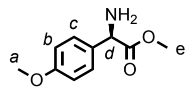

(*R*)-**S8**

Boc-protected amine (*R*)-**S7** (31.9 g, 108 mmol, 1 eq.) was stirred in 4N HCl in dioxane (162 mL, 648 mmol, 6 eq) for 16 h. Et_2O (160 mL) was added and the precipitate was recovered *via* vacuum filtration, washed with more Et_2O and dried in a desiccator. The ammonium salt (22.8 g, 98.4 mmol) was recrystallized from a refluxing solution of EtOAc/MeOH, affording 6.5 g (26%) of salt with an ee > 99%. The latter was treated with saturated $NaHCO_3$ (50 mL) and the aqueous layer was extracted with CH_2Cl_2 (2 × 50 mL). The combined organics were dried ($MgSO_4$), filtered and the solvent evaporated *in vacuo* to give amine (*R*)-**S8** as a colourless oil (5.37 g, 98%). ^{1}H NMR (400 MHz, $CDCl_3$) **δ**: 7.29 (d, J = 8.8, 2H, H_c), 6.88 (d, J = 8.8, 2H, H_b), 4.58 (s, 1H, H_d), 3.80 (s, 3H, H_a), 3.70 (s, 3H, H_e), 1.88 (br s, 2H, -*NH*-). ^{13}C NMR (101 MHz, $CDCl_3$) **δ**: 174.8, 159.5, 132.5, 128.1, 114.3, 58.2, 55.4, 52.5. HPLC: Whelk-O 1 (hexane/*i*-PrOH, 90:10), flow rate 2.0 mL.min^{-1}, λ = 275 nm, t_{major} = 19.6, ee > 99%).

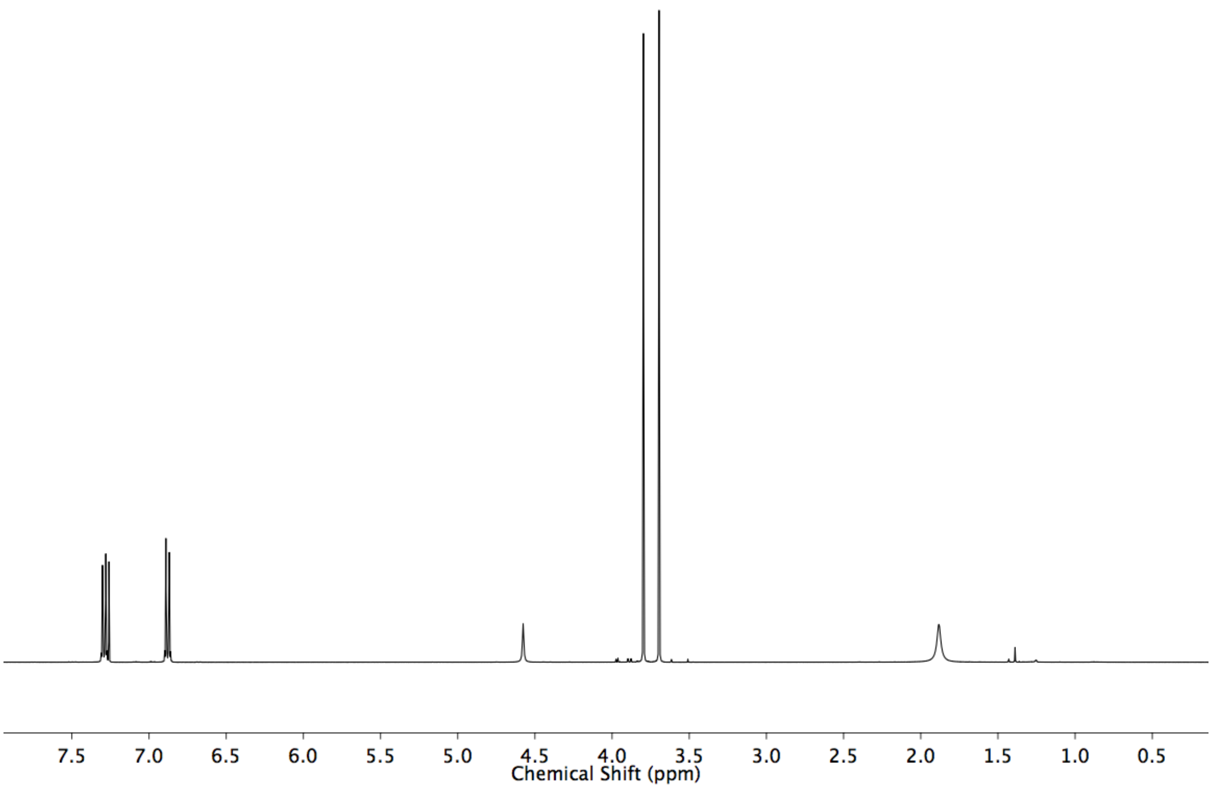

Figure S31 ^{1}H NMR (400 MHz, $CDCl_3$) of (*R*)-**S8**.

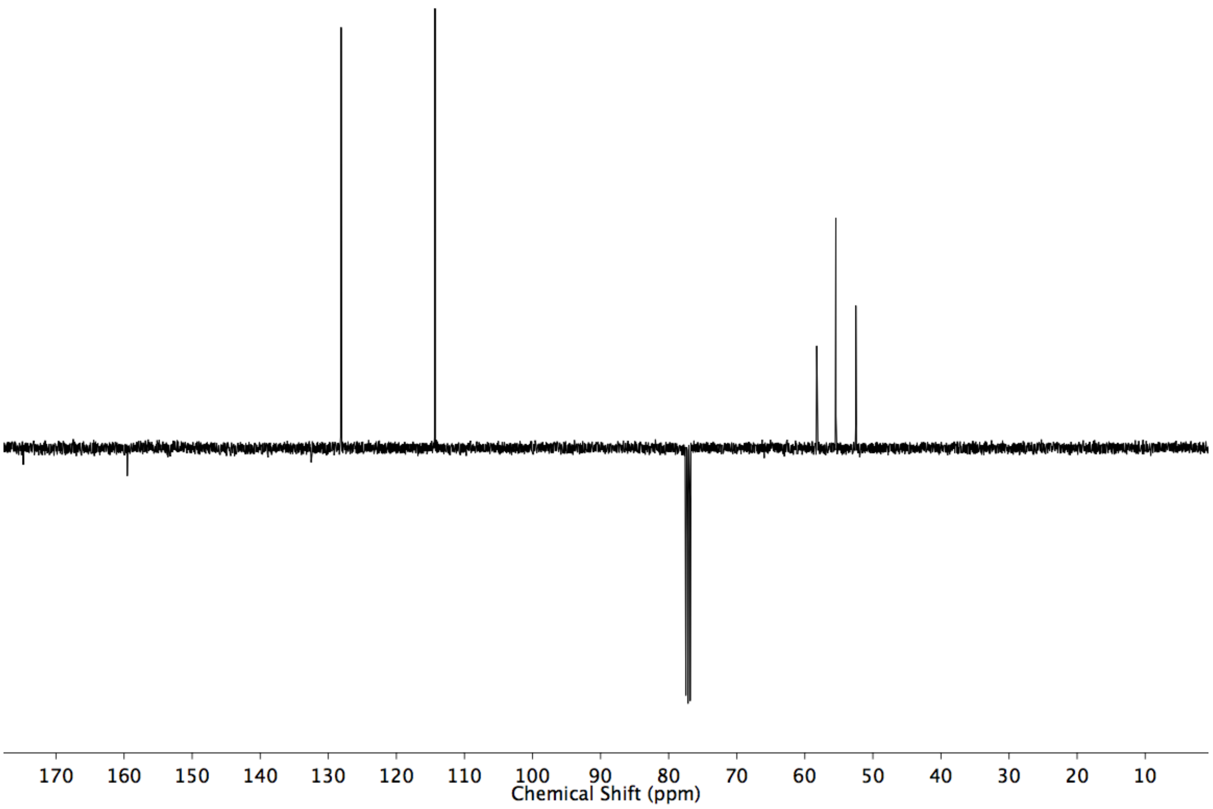

Figure S32 JMOD NMR (101 MHz, $CDCl_3$) of (*R*)-**S8**.

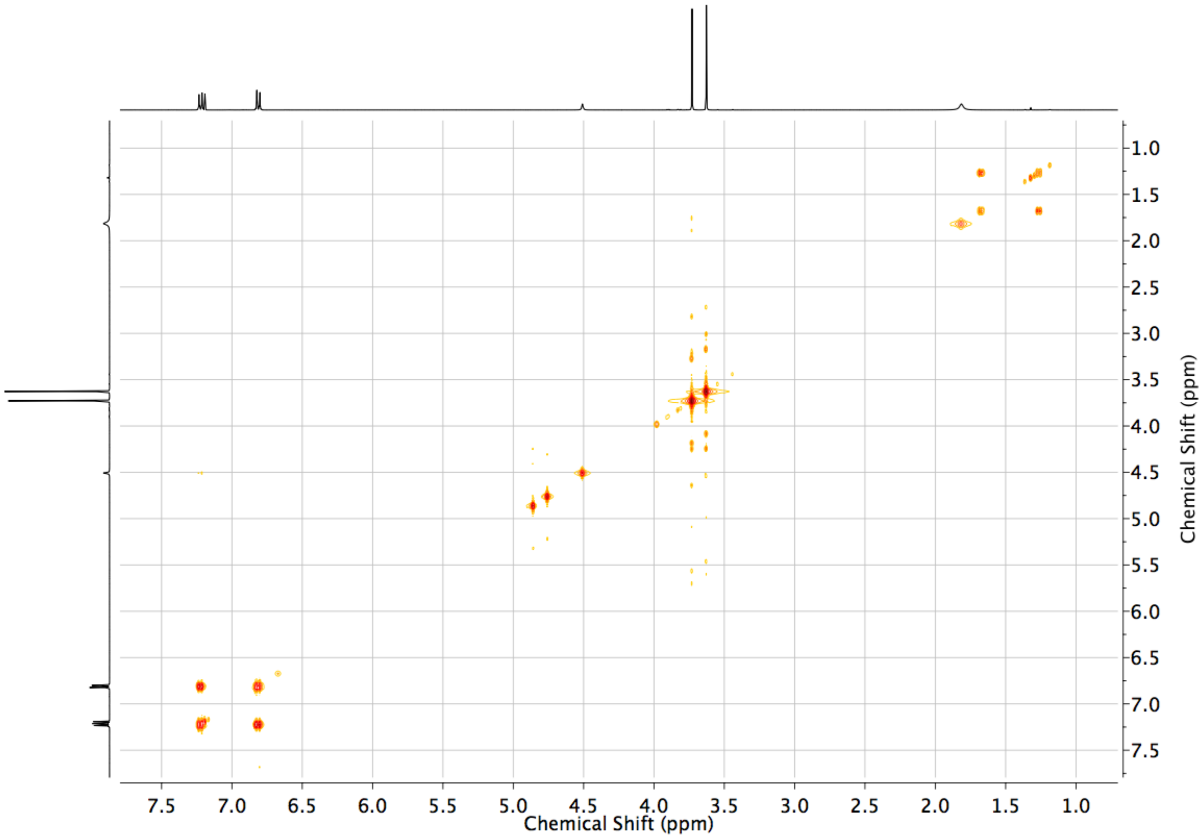

Figure S33 COSY NMR ($CDCl_3$) of (*R*)-**S8**.

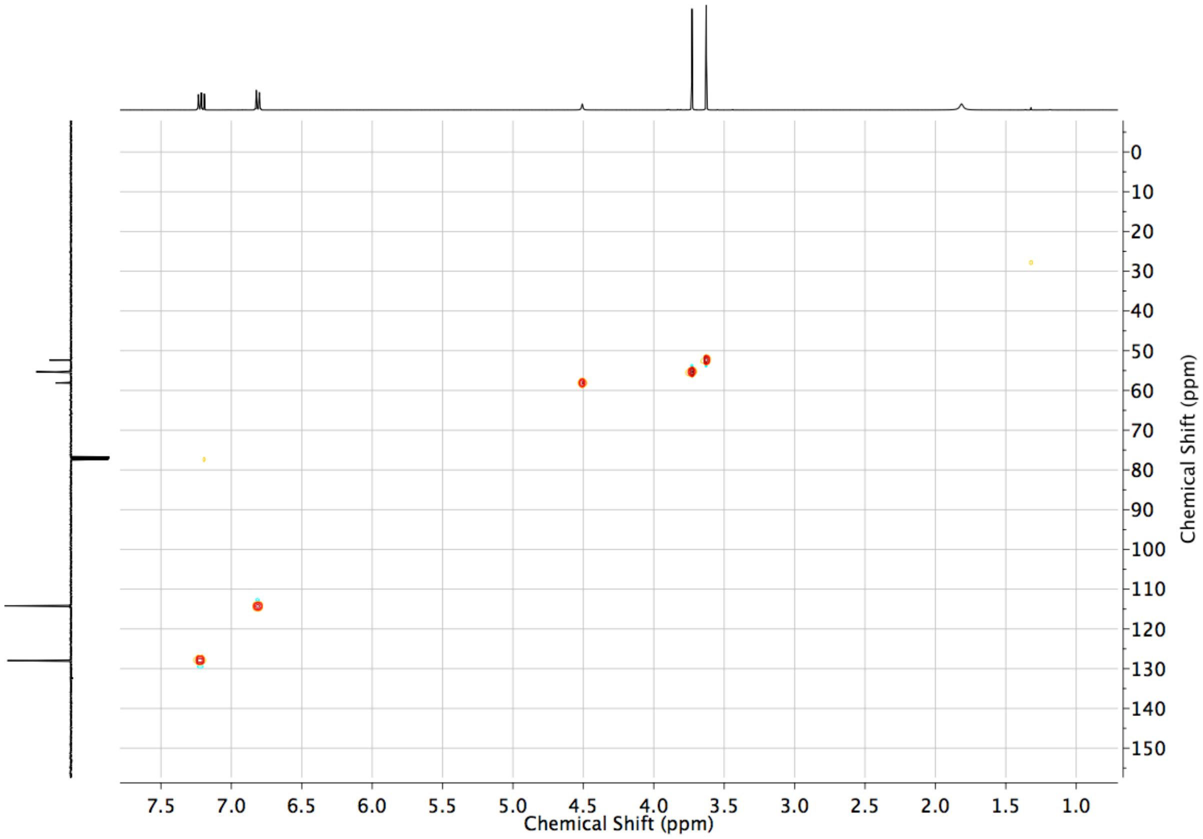

Figure S34 HSQC NMR ($CDCl_3$) of (*R*)-**S8**.

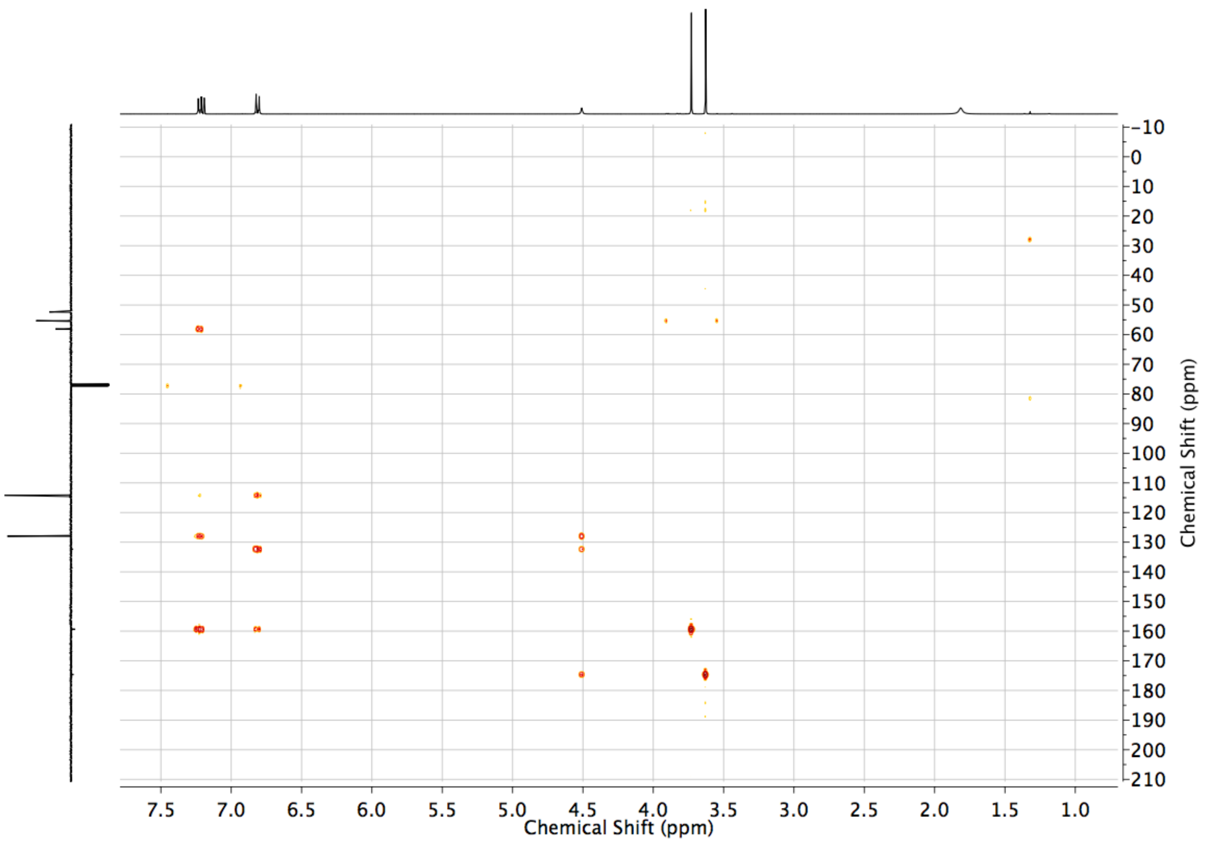

Figure S35 HMBC NMR ($CDCl_3$) of (*R*)-**S8**.

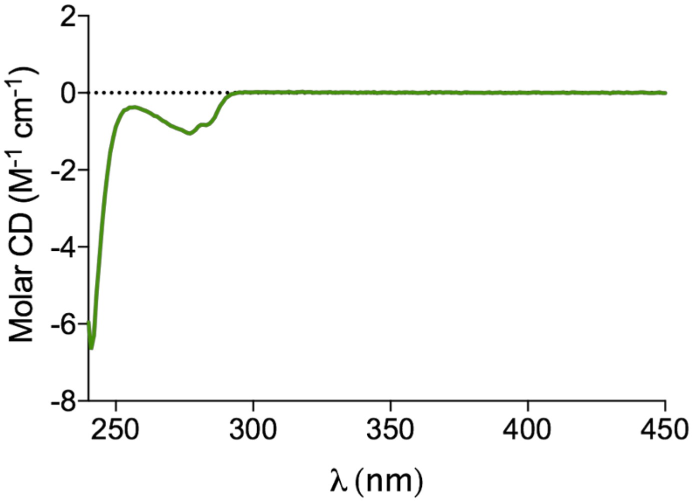

Figure S36 Circular dichroism spectrum of (*R*)-**S8** (200.0 μM in $CHCl_3$, 293 K).

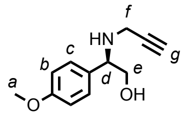

(*R*)-**S9**

To a suspension of LAH (0.751 g, 19.0 mmol, 1.5 eq.) in THF (100 mL) at 0 °C was added dropwise (*R*)-**S8** (2.470 g, 12.7 mmol, 1 eq.) as a solution in THF (10 mL) after which the reaction mixture was allowed to stir at r.t. for 2 h. Water (3 mL) was carefully added to the mixture at 0 °C followed by 10 M aq NaOH (2 mL) and the crude stirred for an additional 30 min. The suspension was then filtered through $MgSO_4$ with THF and the filtrate concentrated. The crude pale yellow solid (1.78 g, 10.7 mmol, 1 eq.) was dissolved in MeCN (50 mL) at r.t. and to this solution was added K_2CO_3 (2.222 g, 16.1 mmol, 1.5 eq.) followed after 15 min by propargyl bromide (80 wt. % in toluene, 1.19 mL, 10.7 mmol, 1 eq.). The resulting mixture was stirred for 16 h then filtered through celite and concentrated *in vacuo*. The crude residue was purified *via* flash column chromatography on silica gel with a step-wise gradient of EtOAc in petrol 0 - 15 - 40%, providing the pure product (*R*)-**S9** as pale yellow oil (1.120 g, 43% over 2 steps). ^{1}H NMR (400 MHz, $CDCl_3$) **δ**: 7.26 (d, *J* = 8.7, 2H, H_c), 6.89 (d, *J* = 8.7, 2H, H_b), 3.96 (dd, *J* = 8.3, 4.5, 1H, H_d), 3.80 (s, 3H, H_a), 3.73 (dd, *J* = 10.8, 4.5, 1H, H_e), 3.60 (dd, *J* = 10.7, 8.3, 1H, H_e), 3.42 (dd, *J* = 17.0, 2.4, 1H, one of H_f), 3.20 (dd, *J* = 17.0, 2.4, 1H, one of H_f), 2.21 (t, *J* = 2.4, 1H, H_g), 1.79 (s, 1H, -*NH*-). ^{13}C NMR (400 MHz, $CDCl_3$) **δ**: 159.4, 131.6, 128.8, 114.2, 82.1, 71.6, 67.0, 62.4, 55.4, 35.8. HR-ESI-MS *m/z* = 206.1173 $[M+H]^+$ (calc. for $C_{12}H_{16}NO_2$ 206.1173). HPLC: RegisPack (hexane/EtOH, 97:3), flow rate 1.5 mL.min^{-1}, λ = 275 nm, t_{major} = 15.3, ee > 99%).

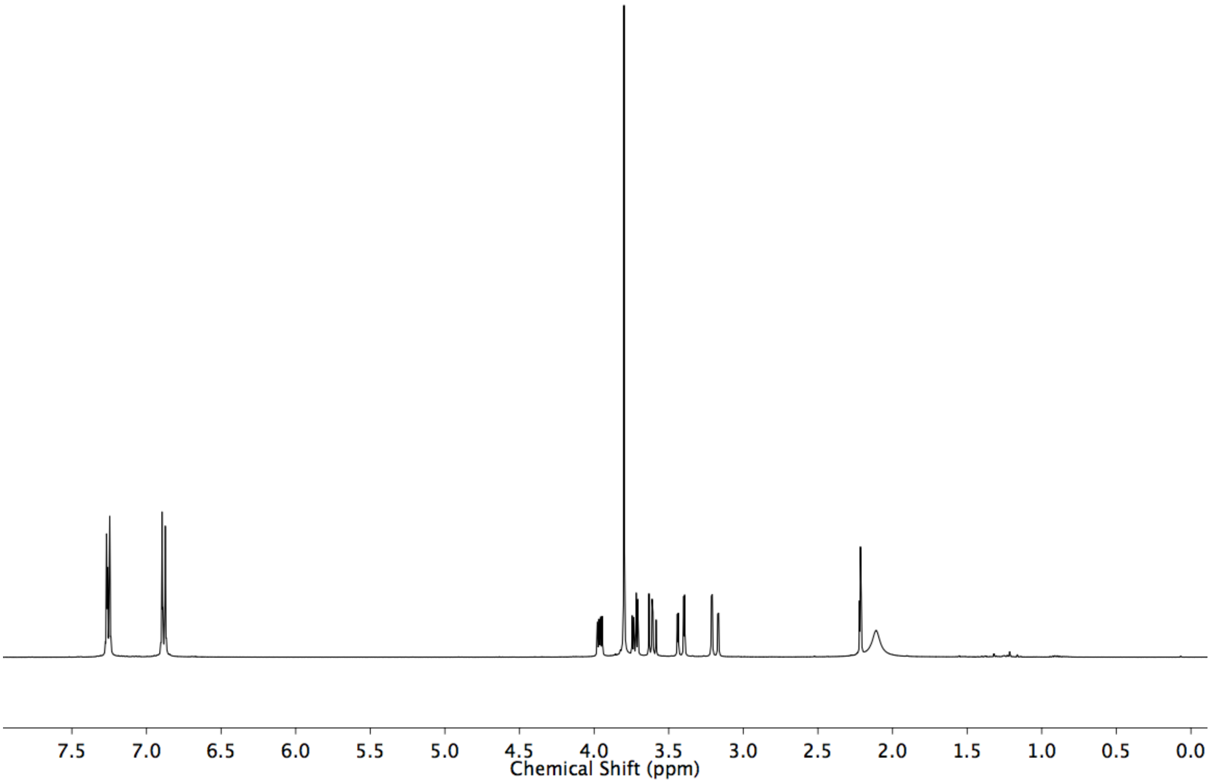

Figure S37 ^{1}H NMR (400 MHz, $CDCl_3$) of (*R*)-**S9**.

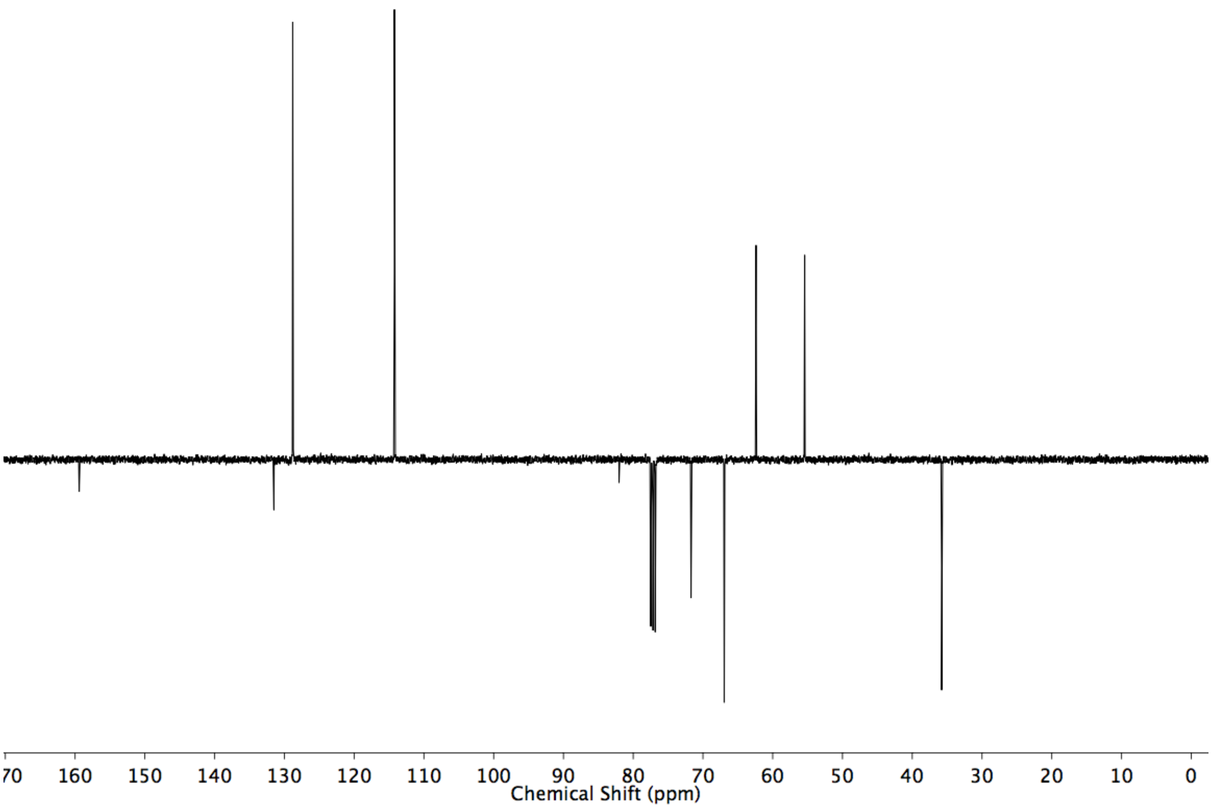

Figure S38 JMOD NMR (101 MHz, $CDCl_3$) of (*R*)-**S9**.

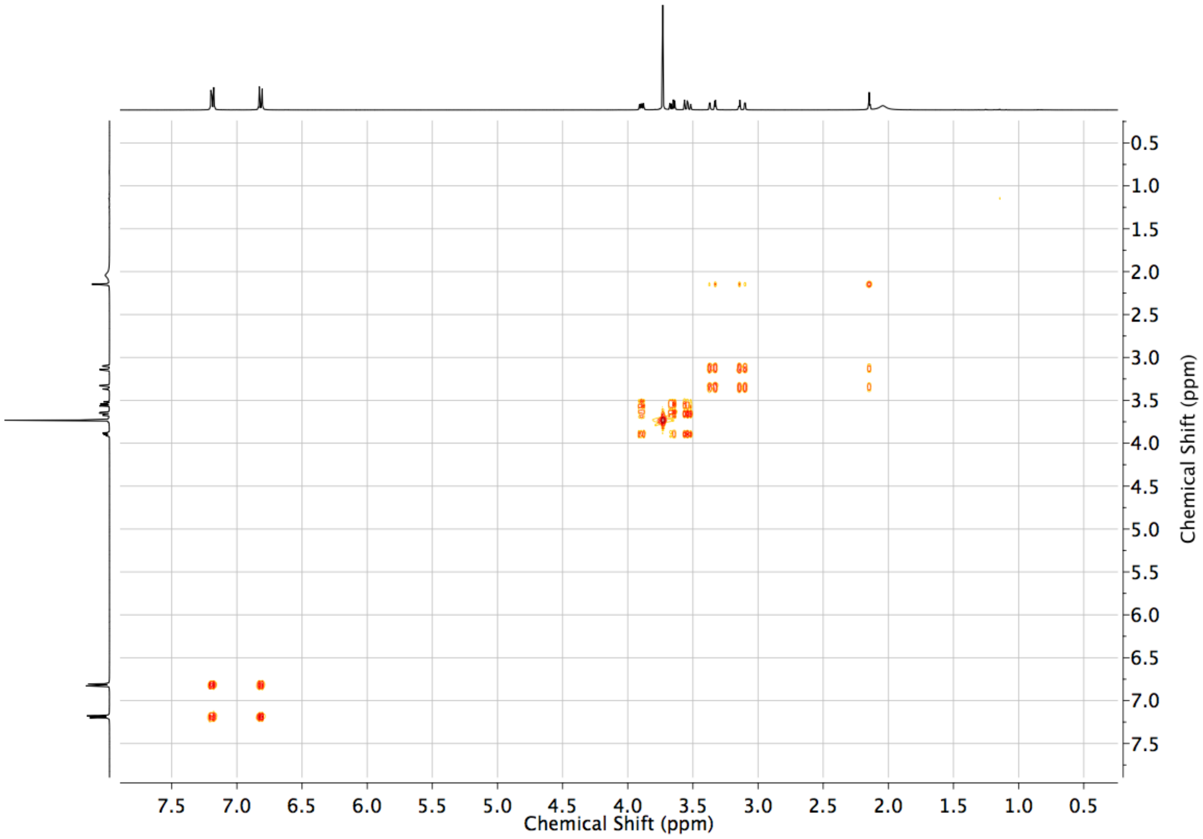

Figure S39 COSY NMR ($CDCl_3$) of (*R*)-**S9**.

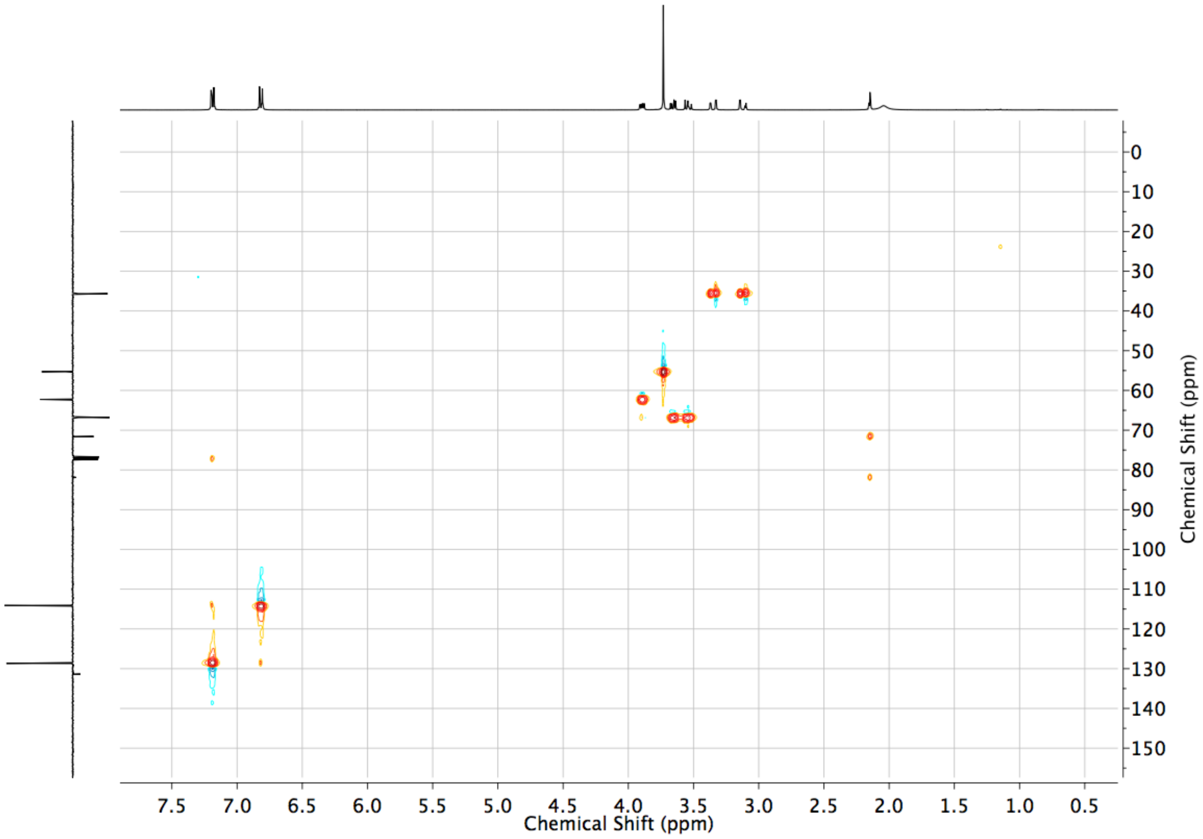

Figure S40 HSQC NMR ($CDCl_3$) of (*R*)-**S9**.

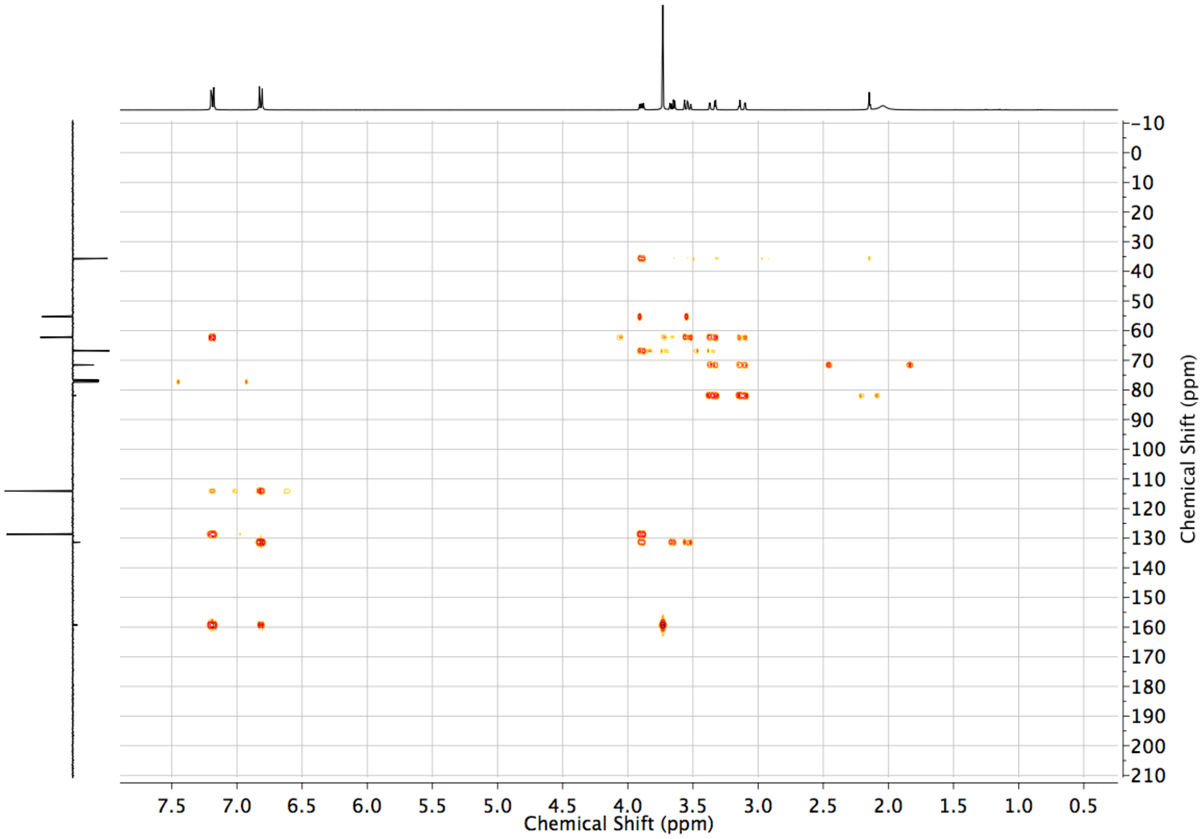

Figure S41 HMBC NMR ($CDCl_3$) of (*R*)-**S9**.

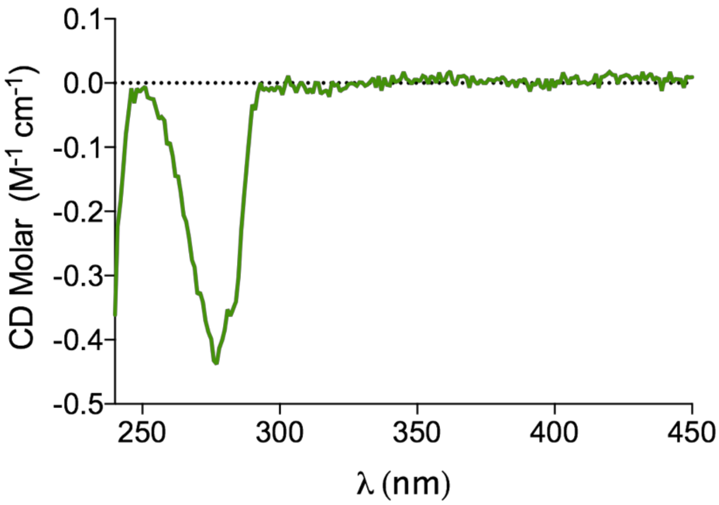

Figure S42 Circular dichroism spectrum of (*R*)-**S9** (200.0 μM in $CHCl_3$, 293 K).

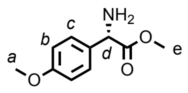

(*S*)-**S8**

Boc-protected amine (*S*)-**S7** (30.1 g, 102 mmol, 1 eq.) was stirred in 4N HCl in dioxane (152 mL, 611 mmol, 6 eq) for 16 h. Et_2O (150 mL) was added and the precipitate was recovered *via* vacuum filtration, washed with more Et_2O and dried in a desiccator. The ammonium salt (22.8 g, 98.4 mmol) was recrystallized from a refluxing solution of EtOAc/MeOH. The process was repeated twice using the filtrate of the previous recrystallization, affording a combined mass of 14.9 g (60%) of salt with an ee > 99%. The latter was treated with saturated $NaHCO_3$ (150 mL) and the aqueous layer was extracted with CH_2Cl_2 (2 × 100 mL). The combined organics were dried ($MgSO_4$), filtered and the solvent evaporated *in vacuo* to give amine (*S*)-**S8** as a colourless oil (12.2 g, 98%). HPLC: Whelk-O 1 (hexane/*i*-PrOH, 90:10), flow rate 2.0 mL.min^{-1}, λ = 275 nm, t_{major} = 15.5, ee > 99%). Spectroscopic data were identical to those reported for (*R*)-**S8** with the exception of the circular dichroism spectra.

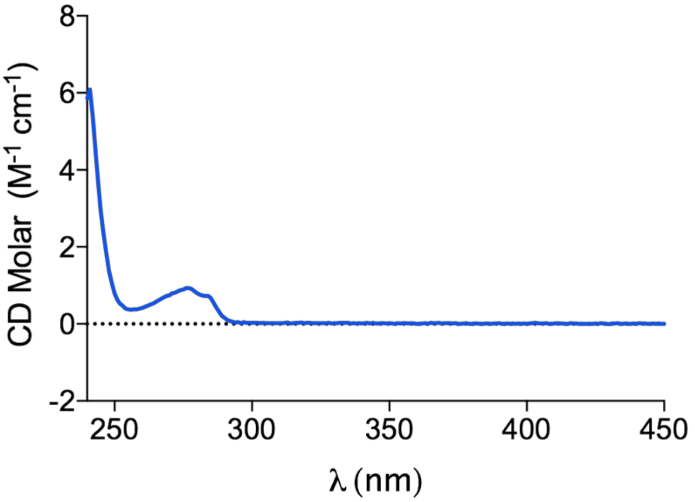

Figure S43 Circular dichroism spectrum of (*S*)-**S8** (200.0 μM in $CHCl_3$, 293 K).

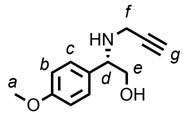

(*S*)-**S9**

To a suspension of LAH (87 mg, 2.20 mmol, 1.5 eq.) in THF (11 mL) at 0 °C was added dropwise (*S*)-**S8** (285 mg, 1.46 mmol, 1 eq.) as a solution in THF (1 mL) after which the reaction mixture was allowed to stir at r.t. for 2 h. Water (0.5 mL) was carefully added to the mixture at 0 °C followed by 10 M aq NaOH (0.5 mL) and the crude stirred for an additional 30 min. The suspension was then filtered through $MgSO_4$ with THF and the filtrate concentrated. The crude pale yellow solid (230 mg, 1.40 mmol, 1 eq.) was dissolved in MeCN (5 mL) at r.t. and to this solution was added K_2CO_3 (291 mg, 2.10 mmol, 1.5 eq.) followed after 15 min by propargyl bromide (80 wt. % in toluene, 0.16 mL, 1.40 mmol, 1 eq.). The resulting mixture was stirred for 16 h then filtered through celite and concentrated *in vacuo*. The crude residue was purified *via* flash column chromatography on silica gel with a step-wise gradient of EtOAc in petrol 0 - 15 - 40%, providing the pure product (*S*)-**S9** as pale yellow oil (181 mg, 40% over 2 steps). HPLC: RegisPack (hexane/EtOH, 97:3), flow rate 1.5 $mL.min^{-1}$, λ = 275 nm, t_{major} = 13.3, ee > 99%). Spectroscopic data were identical to those reported for (*R*)-**S9** with the exception of the circular dichroism spectra.

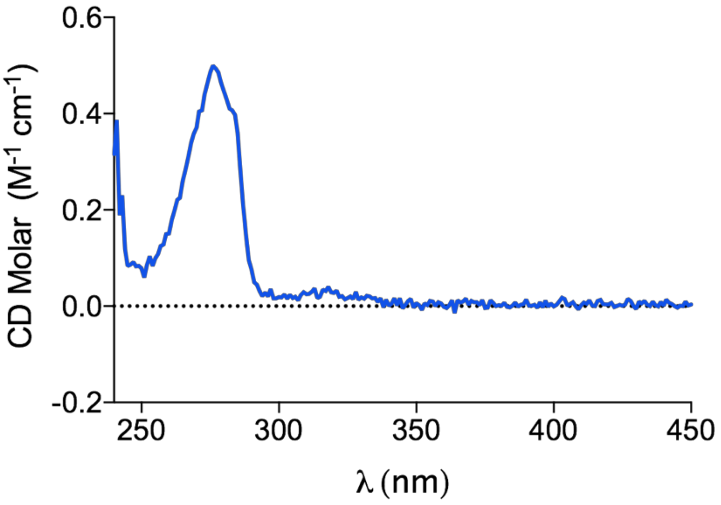

Figure S44 Circular dichroism spectrum of (*S*)-**S9** (200.0 μM in $CHCl_3$, 293 K).

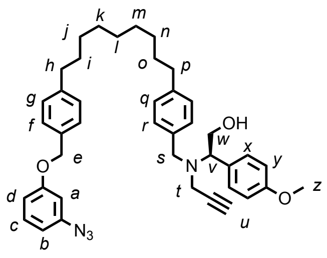

(*R*)-**1**

S10 (0.296 g, 0.568 mmol, 1 eq.), (*R*)-**S9** (0.140 g, 0.682 mmol, 1.2 eq.) and K_2CO_3 (0.785 g, 5.68 mmol, 10 eq.) were stirred at 80 °C in MeCN (25 mL) under air in a sealed vial for 20 h. The cooled reaction mixture was filtered through celite and purified by column chromatography (Petrol/CH_2Cl_2 1/1 with a gradient from 0 to 10% EtOAc) to give the product (*R*)-**1** as a colorless oil (0.362 g, 99%). ^{1}H NMR (500 MHz, $CDCl_3$) **δ**: 7.33-7.30 (m, 4H, H_f, H_x), 7.26-7.19 (m, 5H, H_c, H_g, H_r), 7.12 (d, *J* = 8.1, 2H, H_q), 6.91 (d, *J* = 8.8, 2H, H_y), 6.76 (ddd, *J* = 8.3, 2.3, 0.9, 1H, H_b or H_d), 6.66-6.63 (m, 2H, H_a, H_b or H_d), 5.01 (s, 2H, H_e), 4.01-3.97 (m, H_v, H_w), 3.84-3.77 (m, 5H, H_s, $H_{w'}$, H_z), 3.41-3.37 (m, 1H, H_t), 3.31 (d, *J* = 13.3, 1H, $H_{s'}$), 3.13 (dd, *J* = 17.2, 2.4, 1H, $H_{t'}$), 2.62-2.56 (m, 4H, H_h, H_l), 2.23 (app. t, *J* = 2.4, 1H, H_u), 1.63-1.55 (m, 4H, H_i, H_o), 1.30 (br. m, 10H, H_j, H_k, H_l, H_m, H_n). ^{13}C NMR (126 MHz, $CDCl_3$) **δ**: 160.2, 159.5, 143.2, 142.1, 141.4, 135.8, 133.8, 130.6, 130.0, 129.8, 129.0, 128.8, 128.6, 127.8, 114.1, 111.6 (×2) 106.1, 79.6, 73.3, 70.3, 65.4, 62.8, 55.4, 53.9, 39.0, 35.9, 35.8, 31.7, 31.6, 29.6 (×3), 29.5, 29.4. HR-ESI-MS *m/z* = 645.3799 $[M+H]^+$ (calc. for $C_{41}H_{49}N_4O_3$ 645.3799).

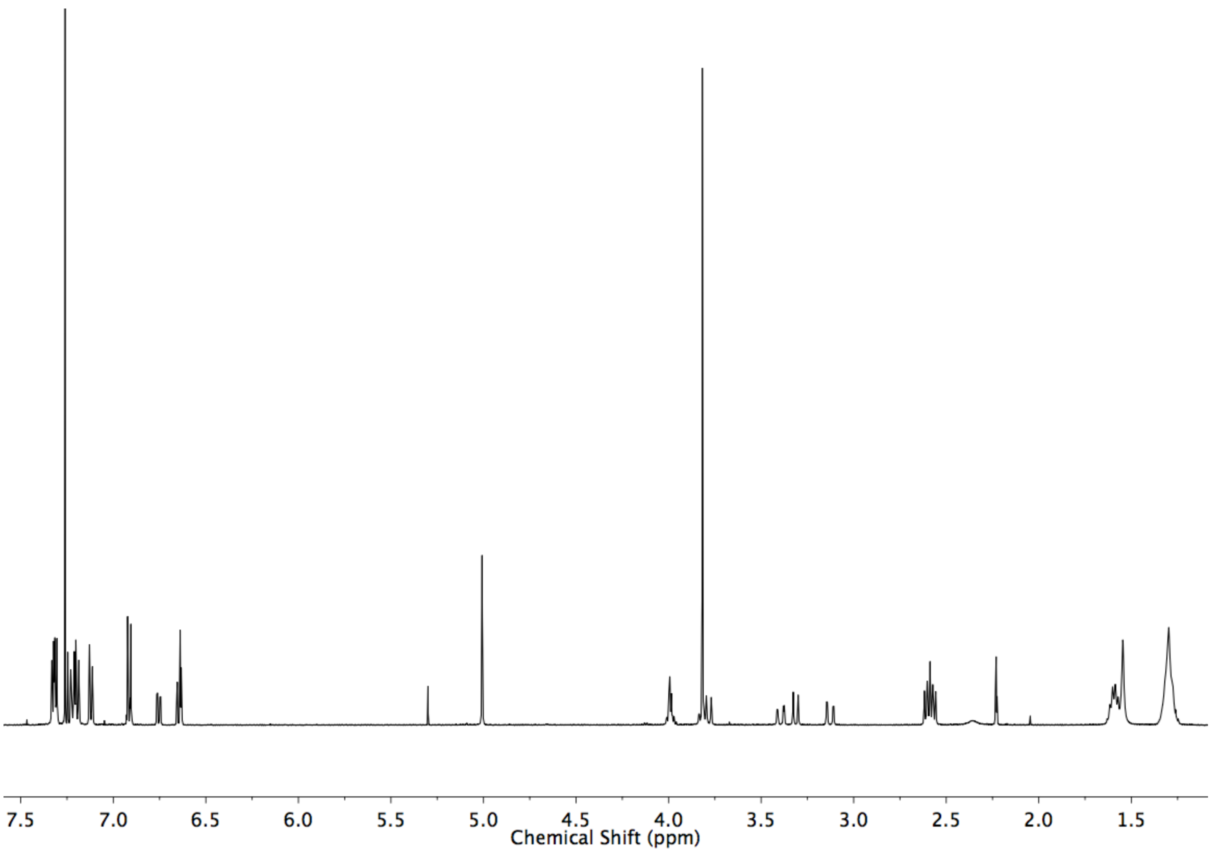

Figure S45 ^{1}H NMR (500 MHz, $CDCl_3$) of (*R*)-**1**.

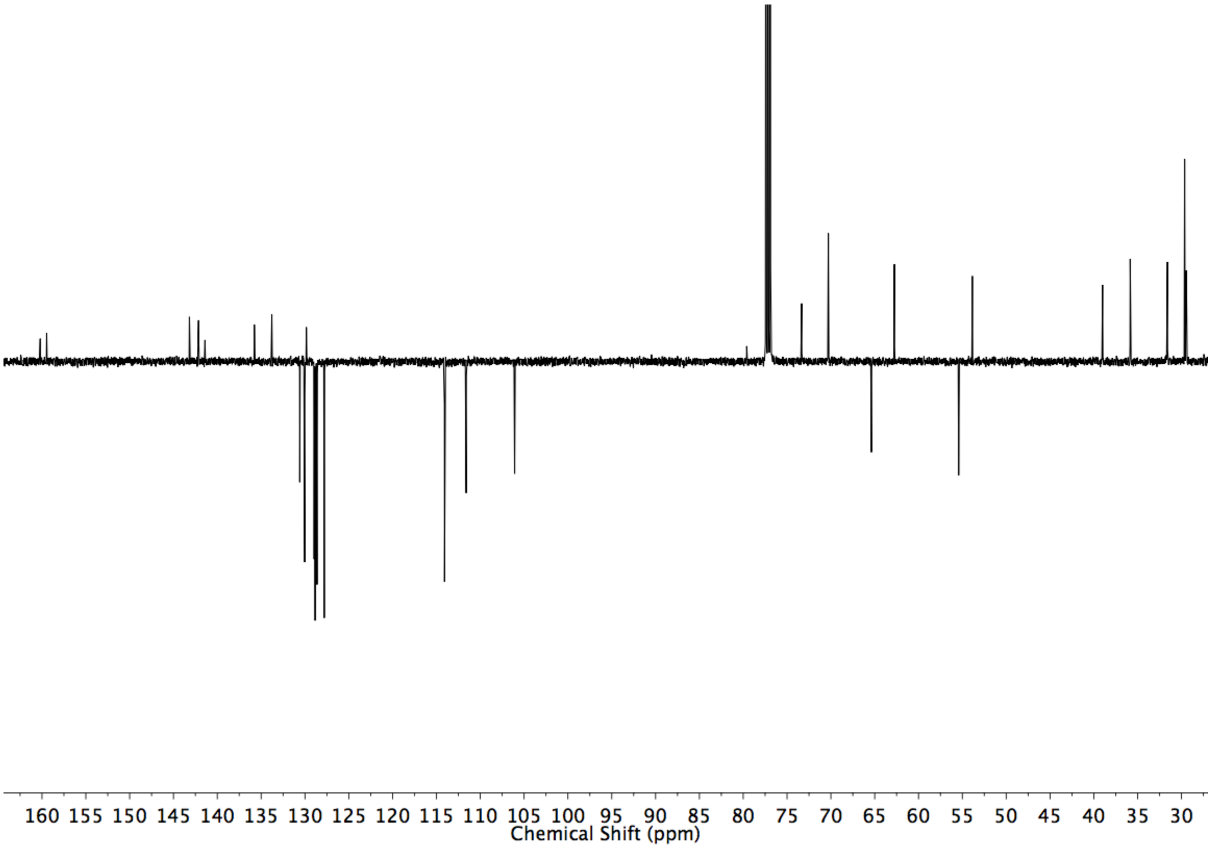

Figure S46 JMOD NMR (126 MHz, $CDCl_3$) of (*R*)-**1**.

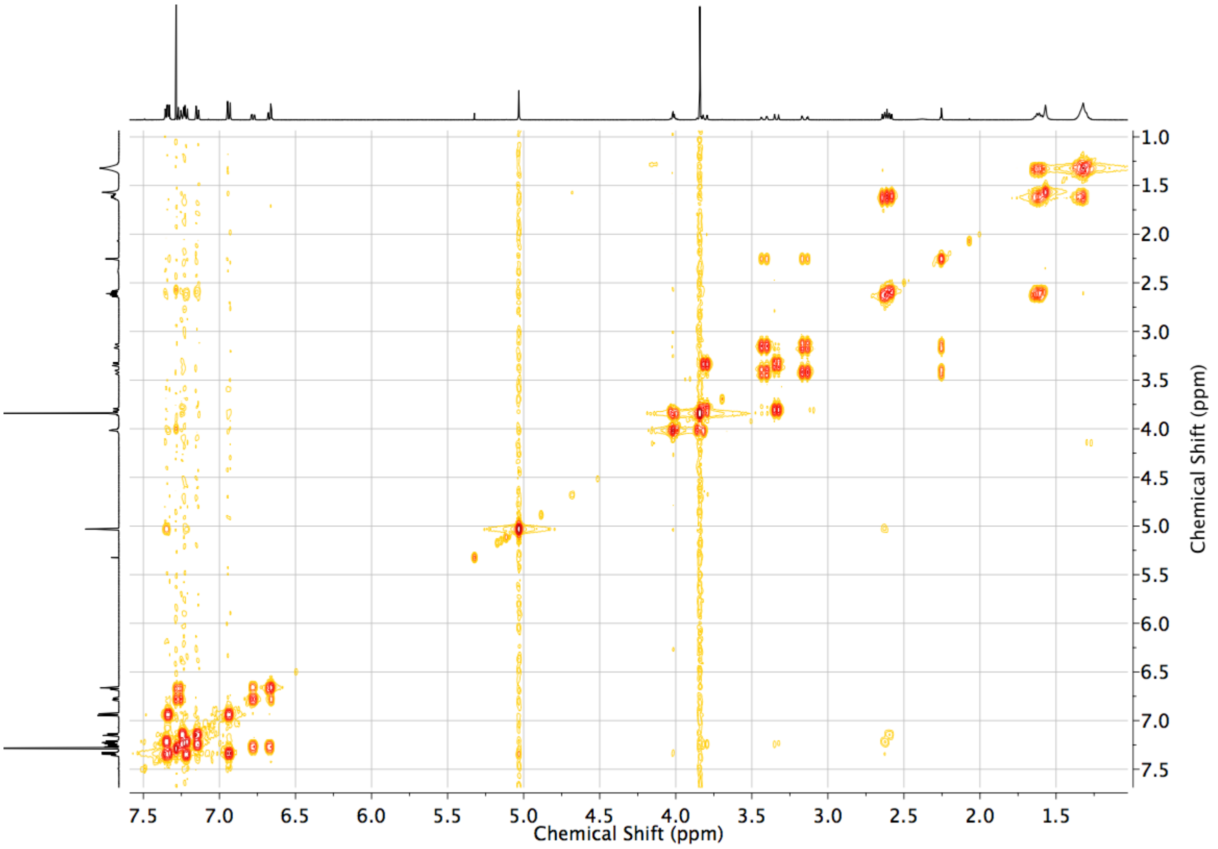

Figure S47 COSY NMR ($CDCl_3$) of (*R*)-**1**.

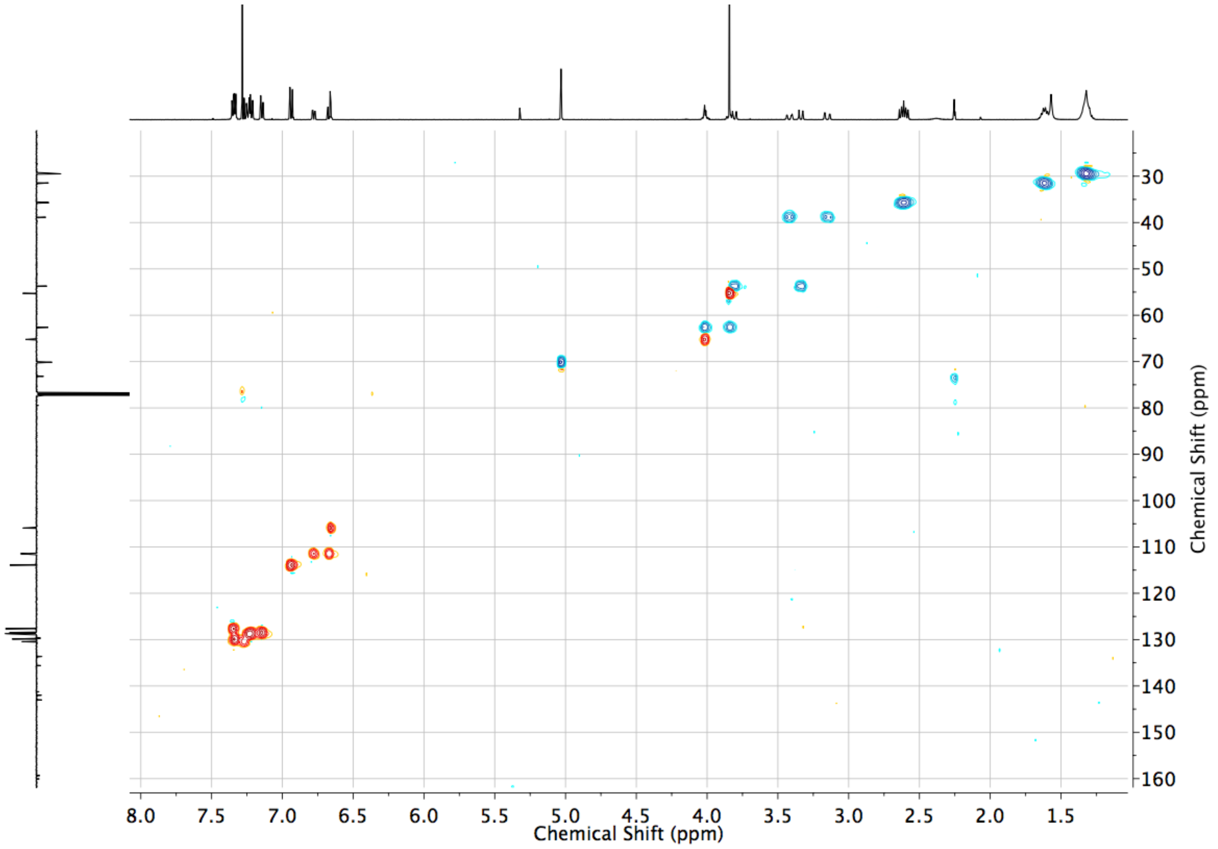

Figure S48 HSQC NMR ($CDCl_3$) of (*R*)-**1**.

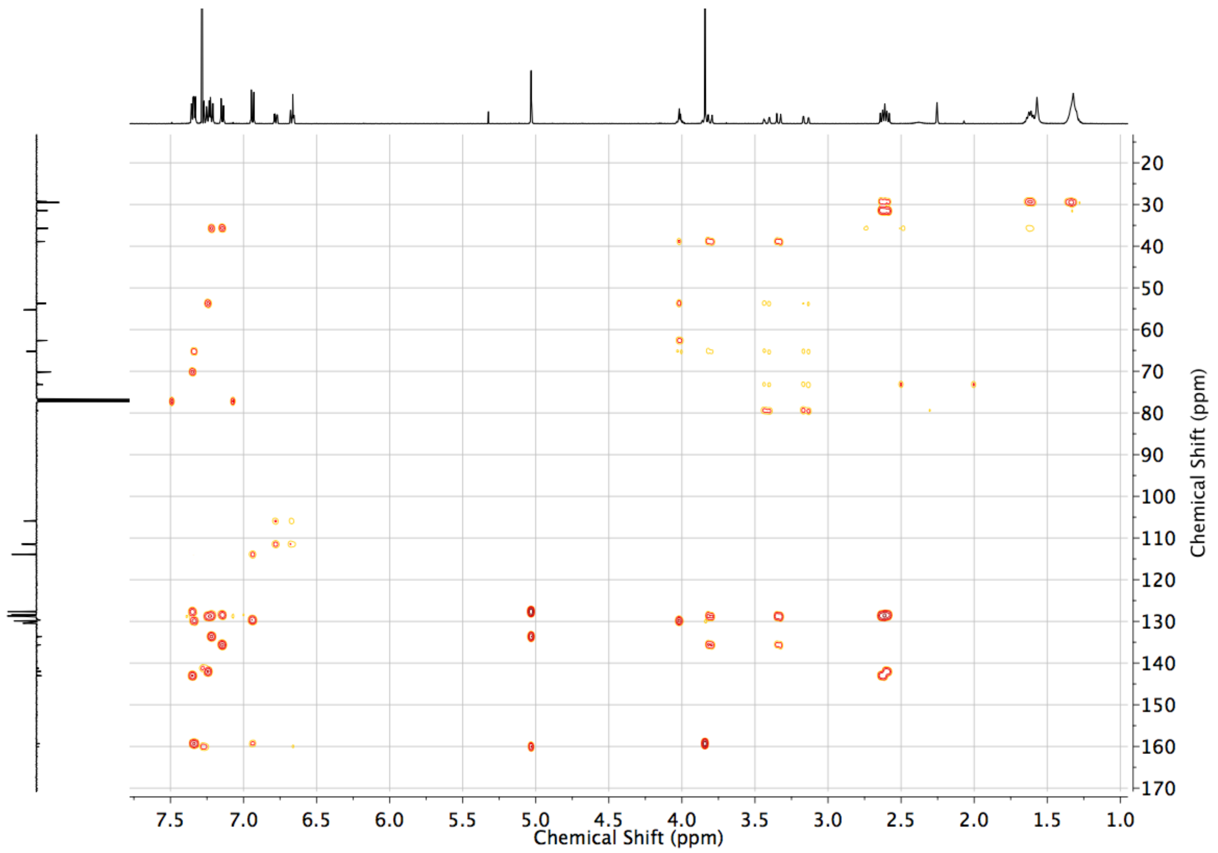

Figure S49 HMBC NMR ($CDCl_3$) of (*R*)-**1**.

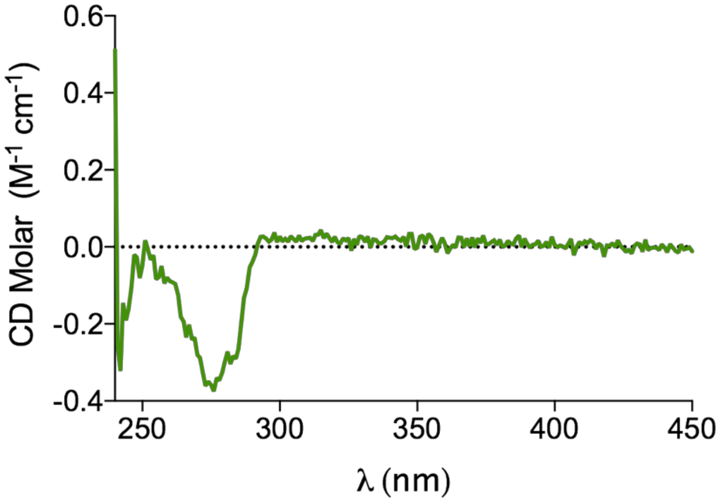

Figure S50 Circular dichroism spectrum of (*R*)-**1** (100.0 μM in $CHCl_3$, 293 K).

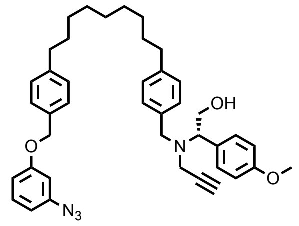

(*S*)-**1**

S10 (630 mg, 1.21 mmol, 1 eq.), (*S*)-**S9** (308 mg, 1.50 mmol, 1.2 eq.) and K_2CO_3 (829 mg, 6.00 mmol, 5 eq.) were stirred at 80 °C in MeCN (50 mL) under air in a sealed vial for 20 h. The cooled reaction mixture was filtered through celite and purified by column chromatography (Petrol/CH_2Cl_2 1/1 with a gradient from 0 to 10% EtOAc) to give the product as a colorless oil (700 mg, 90%). Spectroscopic data were identical to those reported for (*R*)-**1**.

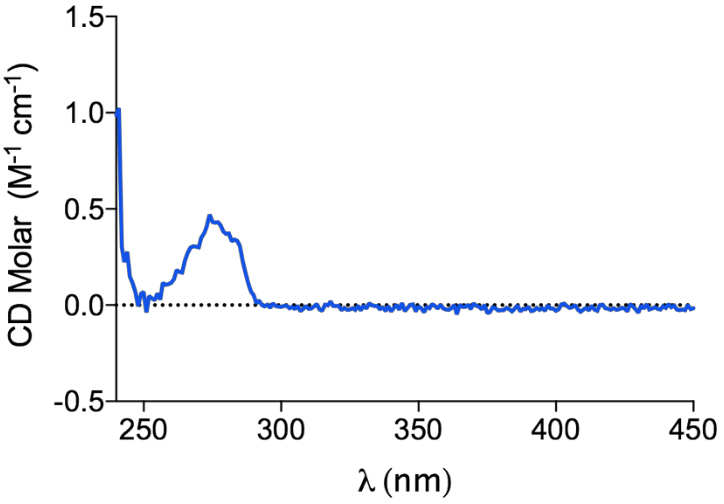

Figure S51 Circular dichroism spectrum of (*S*)-**1** (100.0 μM in $CHCl_3$, 293 K).

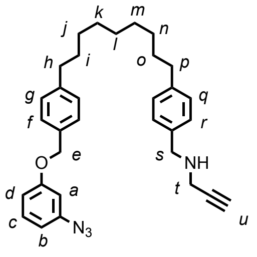

S11

S10 (253 mg, 0.50 mmol, 1 eq.), propargylamine (83 mg, 1.5 mmol, 3.0 eq.) and K_2CO_3 (83 mg, 0.60 mmol, 1.2 eq.) were stirred at 80 °C in MeCN (3 mL) under air in a sealed vial for 24 h. The cooled reaction mixture was filtered through celite and purified by column chromatography (CH_2Cl_2 with a gradient from 0 to 5% EtOAc) to give the product as a colorless oil (40 mg, 17%). ^{1}H NMR (400 MHz, $CDCl_3$) **δ**: 7.34 (d, J = 8.2, 2H, H_f), 7.28-7.23 (m, 3H, H_r, H_c), 7.21 (d, J = 8.2, 2H, H_g), 7.15 (d, J = 8.2, 2H, H_q), 6.79-6.75 (m, 1H, H_b or H_d), 6.68-6.63 (m, 2H, H_a, H_b or H_d), 5.02 (s, 2H, H_e), 3.86 (s, 2H, H_s), 3.44 (d, J = 2.4, 2H, H_t), 2.61 (td, J = 9.5, 7.5, 4H, H_h, H_p), 2.26 (t, J = 2.4, 1H, H_u), 1.69-1.54 (m, 4H, H_i, H_o), 1.39-1.23 (m, 10H, H_j, H_k, H_l, H_m, H_n). ^{13}C NMR (101 MHz, $CDCl_3$) **δ**: 160.2, 143.2, 142.0, 141.4, 136.7, 133.8, 130.6, 128.8, 128.6, 128.5, 127.8, 111.7 (×2), 106.1, 82.3, 71.6, 70.3, 52.2, 37.5, 35.8, 35.8, 31.6 (×2), 29.6 (×3), 29.4 (×2). HR-ESI-MS *m/z* 495.3121 $[M+H]^+$ (calc. for $C_{32}H_{39}N_4O$ 495.3118).

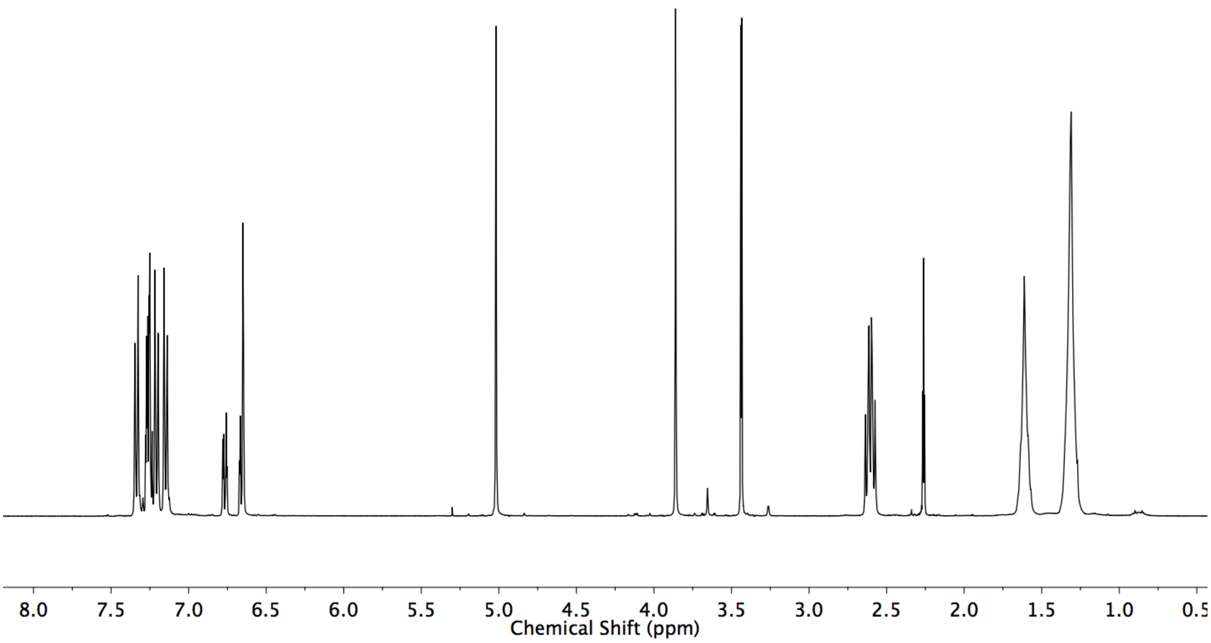

Figure S52 ^{1}H NMR (400 MHz, $CDCl_3$) of **S11**.

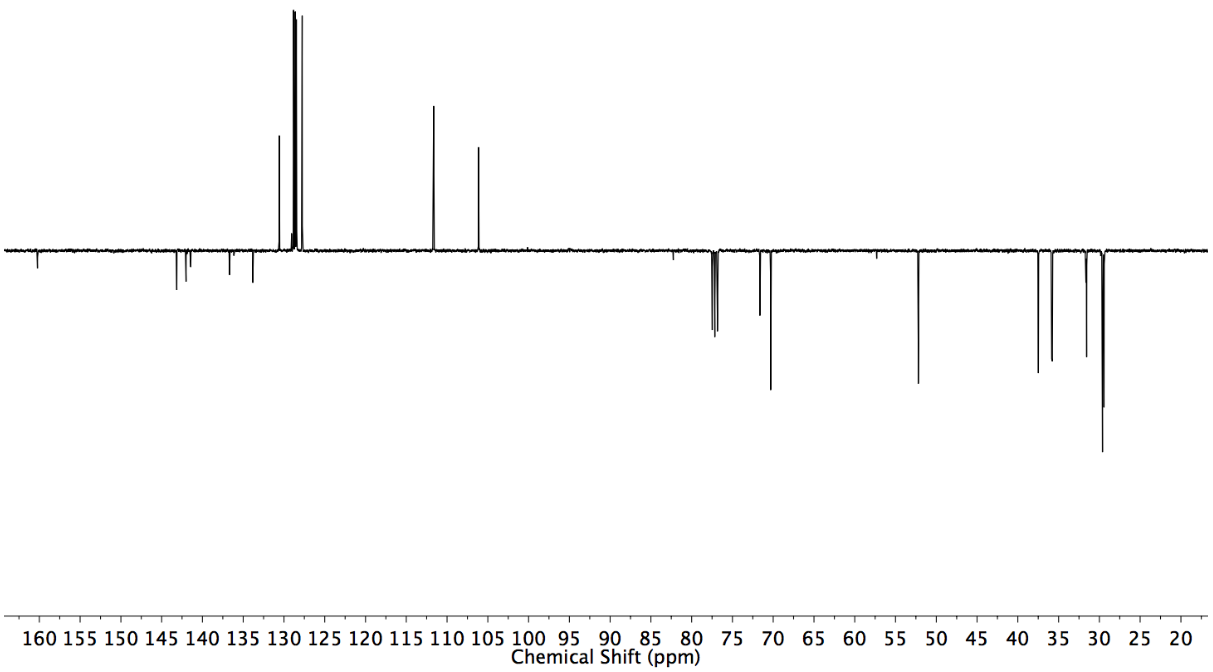

Figure S53 JMOD NMR (101 MHz, $CDCl_3$) of **S11**.

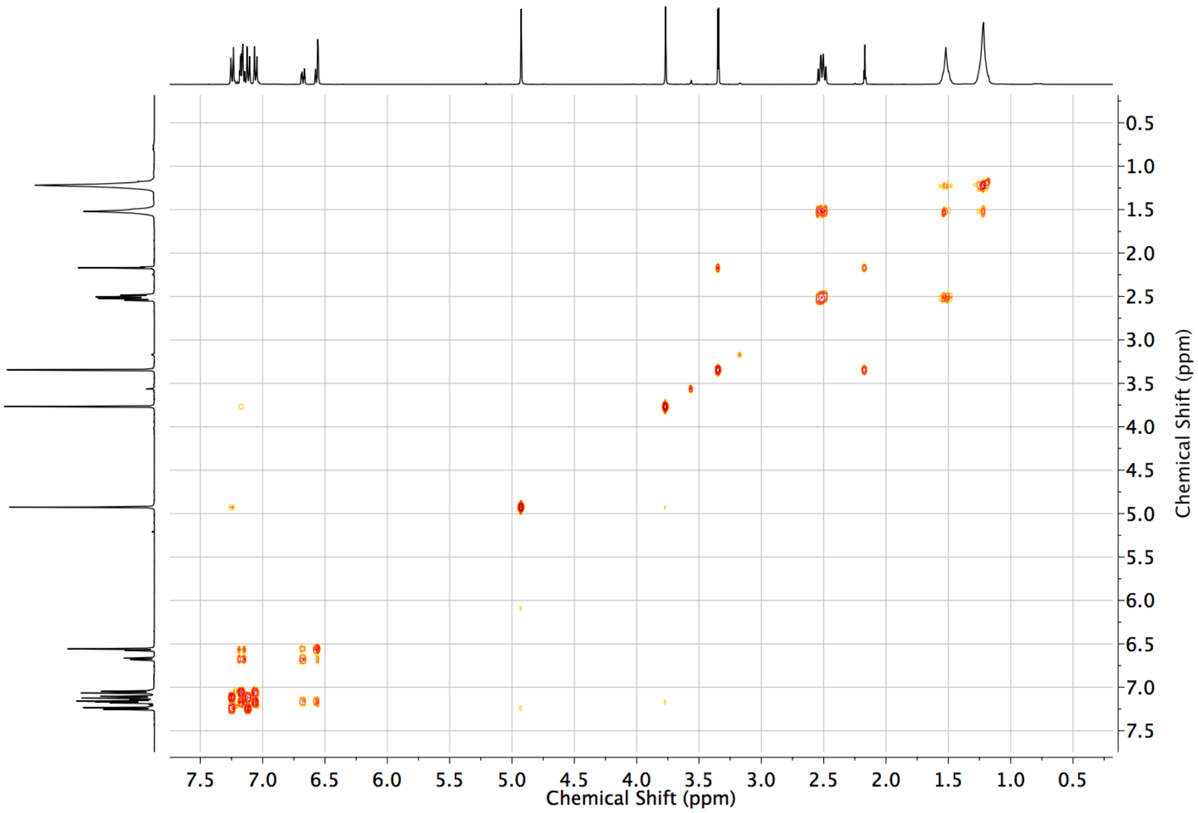

Figure S54 COSY NMR ($CDCl_3$) of **S11**.

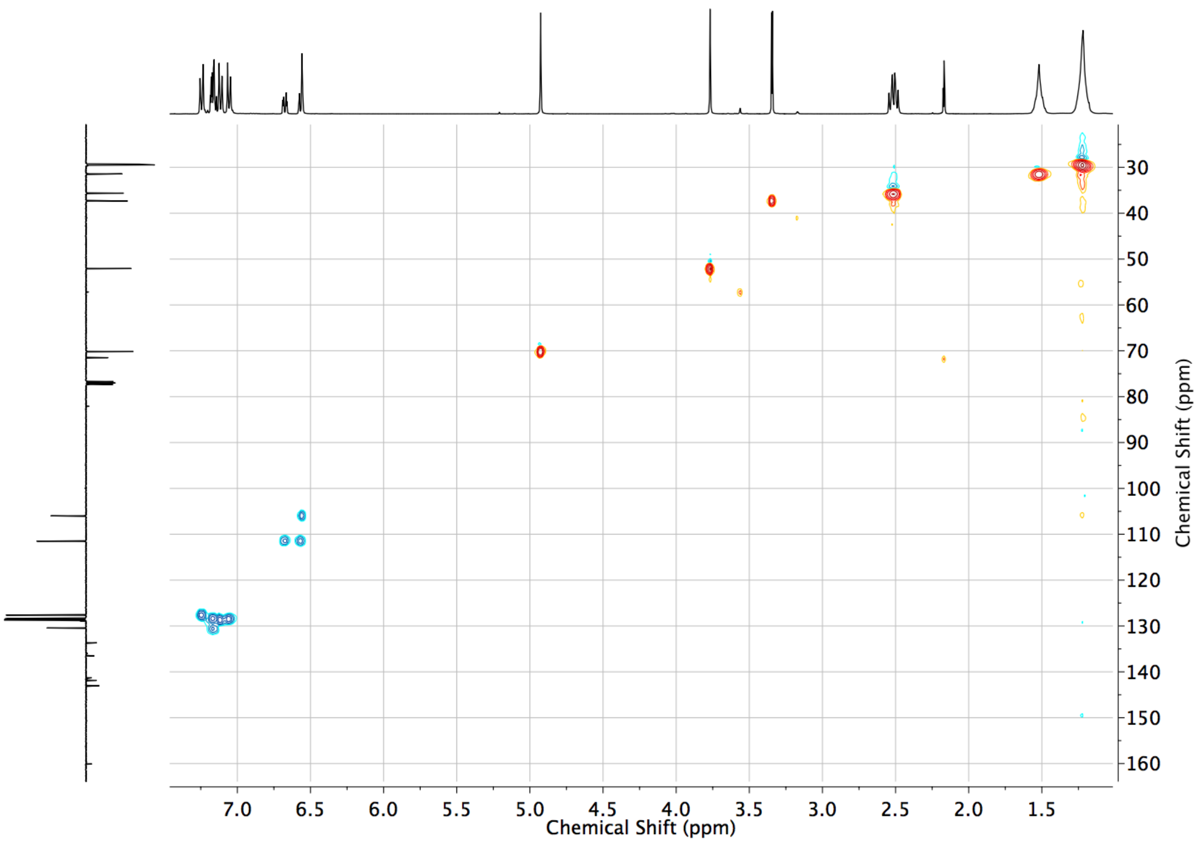

Figure S55 HSQC NMR ($CDCl_3$) of **S11**.

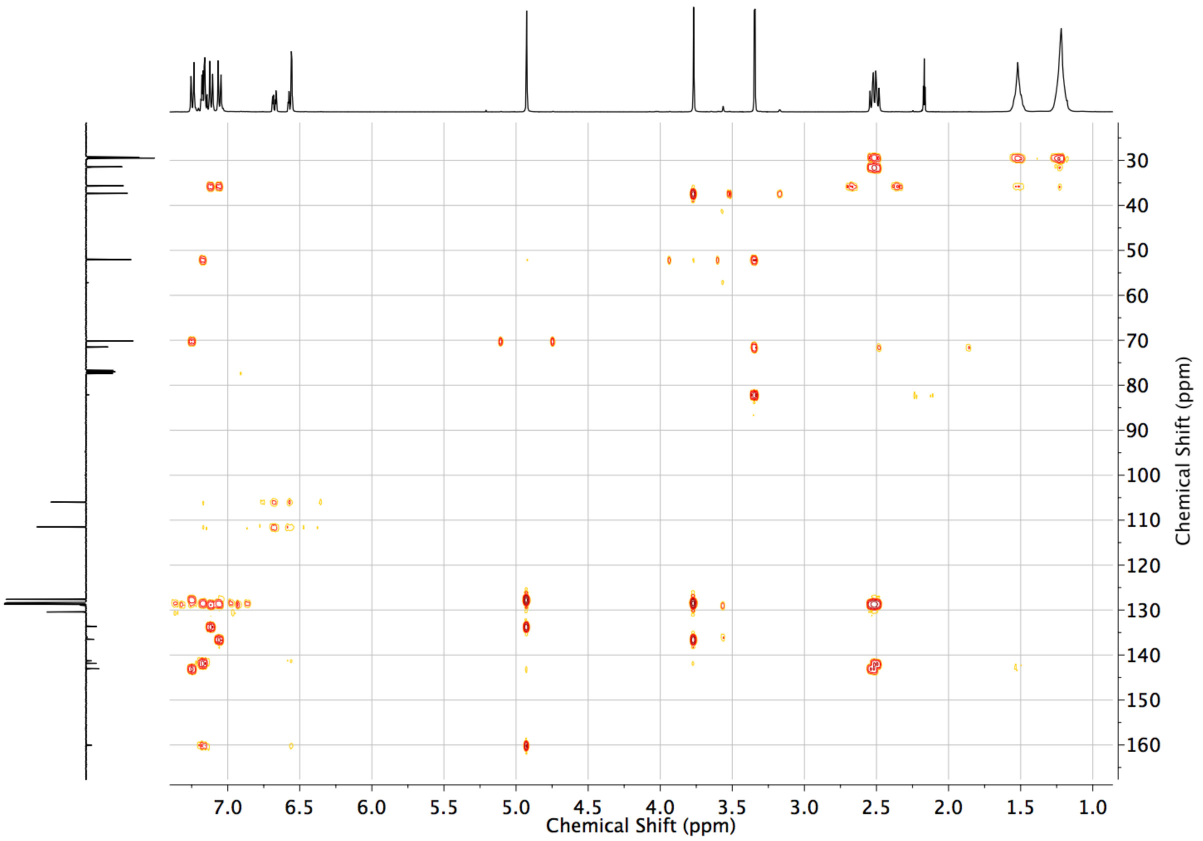

Figure S56 HMBC NMR ($CDCl_3$) of **S11**.

4. Syntheses of catenanes 3 and 6

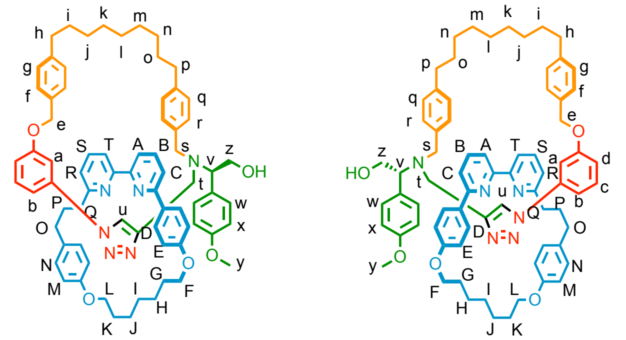

(R,S_{mt})-**3a** (R,R_{mt})-**3a**

(R,R/S_{mt})-3a

To a solution of **2a** (24.1 mg, 0.050 mmol, 1 eq.), $[Cu(CH_3CN)_4]PF_6$ (18.5 mg, 0.0495 mmol, 0.99 eq.), $^iPr_2NEt$ (18 µL, 0.10 mmol, 2 eq.) in 1:1 $CHCl_3$/EtOH (5.0 mL) at 60 °C was added (R)-**1** (40.0 mg, 0.060 mmol, 1.2 eq.) in 1:1 $CHCl_3$/EtOH (2.4 mL) over 4 h. After removal of the solvent *in vacuo*, the residue was dissolved in 1:1 CH_2Cl_2/MeOH (2.5 mL) and KCN (16 mg, 0.25 mmol, 5 eq.) added as a solid. After stirring at r.t. for 30 minutes the solvent was removed under a flow of air. The residue was dissolved in CH_2Cl_2 (5 mL) and washed with H_2O (4 × 5 mL), dried ($MgSO_4$) and the solvent removed *in vacuo*. After purification by column chromatography on silica (Petrol with a gradient of 0 to 100% Et_2O over 10 CVs) (R,R/S_{mt})-**3a** was obtained as a white foam (40.1 mg, 72%, 1 : 1 diastereoisomeric ratio determined by ^1H NMR). ^1H NMR (400 MHz, $CDCl_3$) **δ**: 9.24 (s, 1H H_u), 9.19 (s, 1H , $H_{u'}$), 7.82 (t, J = 7.8, 1H, H_S), 7.79 (t, J = 7.8, 1H, $H_{S'}$), 7.74 (t, J = 7.8, 1H, H_B), 7.71-7.63 (m, 3H, $H_{B'}$, H_T, $H_{T'}$), 7.57 (dd, J = 7.8, 0.9, 1H, H_A), 7.49 (dd, J = 7.8, 0.9, 1H, $H_{A'}$), 7.40-7.32 (m, 3H, H_a or $H_{a'}$ or H_b or $H_{b'}$ or H_c or $H_{c'}$ or H_d or $H_{d'}$), 7.28-7.02 (m, 24H, H_C, $H_{C'}$, H_R, $H_{R'}$, H_r, $H_{r'}$, H_w, H_f, H_q, $H_{q'}$, H_a or $H_{a'}$ or H_b or $H_{b'}$ or H_c or $H_{c'}$ or H_d or $H_{d'}$), 7.01-6.95 (m, 3H, H_g, H_a or $H_{a'}$ or H_b or $H_{b'}$ or H_c or $H_{c'}$ or H_d or $H_{d'}$), 6.95-6.87 (m, 3H, $H_{w'}$, H_a or $H_{a'}$ or H_b or $H_{b'}$ or H_c or $H_{c'}$ or H_d or $H_{d'}$), 6.82 (d, J = 8.7, 2H, H_x), 6.76 (s, 4H, $H_{f'}$, $H_{g'}$), 6.71 (d, J = 8.7, 2H, H_D or $H_{D'}$ or H_N or $H_{N'}$), 6.71 (d, J = 8.7, 2H, $H_{x'}$), 6.28 (d, J = 8.5, 2H, H_D or $H_{D'}$ or H_N or $H_{N'}$), 6.21 (d, J = 8.7, 2H, H_E or $H_{E'}$ or H_M or $H_{M'}$), 6.17 (d, J = 8.3 Hz, 2H, H_q or $H_{q'}$ or H_r or $H_{r'}$), 6.02 (d, J = 8.7, 4H, H_E or $H_{E'}$ or H_M or $H_{M'}$), 5.93 (d, J = 8.3 Hz, 2H, H_q or $H_{q'}$ or H_r or $H_{r'}$), 5.53 (d, J = 8.7, 4H, H_E or $H_{E'}$ or H_M or $H_{M'}$), 5.12-4.96 (m, 2H, H_e), 4.80 (d, J = 14.4, 1H, $H_{e'}$), 4.57 (d, J = 14.4, 1H, $H_{e'}$), 4.21-3.82 (m, 8H, H_v, $H_{v'}$, H_z, $H_{z'}$, H_F or $H_{F'}$ or H_L or $H_{L'}$), 3.79 (s, 3H, H_y), 3.76 (s, 3H, $H_{y'}$), 3.75-3.50 (m, 6H, 1 of H_s, 1 of $H_{s'}$, H_F or $H_{F'}$ or H_L or $H_{L'}$), 3.41 (d, J = 14.4, 1H, 1 of $H_{t'}$), 3.18 (d, J = 14.4, 1H, 1 of H_t), 3.01 (d, J = 14.4, 1H, 1 of H_t), 2.99 (d, J = 14.4, 1H, 1 of $H_{t'}$), 2.86 (d, J = 14.4, 1H, 1 of $H_{s'}$), 2.70-2.38 (m, 5H, H_h, $H_{h'}$, 1 of H_s), 1.96-1.78 (m, 8H, H_G, $H_{G'}$, H_K, $H_{K'}$), 1.76-1.48 (m, 12H, H_H, $H_{H'}$, H_J, $H_{J'}$, H_i, $H_{i'}$), 1.29-1.20 (m, 2H, $H_{i'}$), 1.13-0.95 (m, 8H, H_I, $H_{I'}$, Hj, $H_{j'}$), 0.78-0.68 (m, 4H, H_l, $H_{l'}$). ^{13}C NMR (101 MHz, $CDCl_3$) **δ** 163.6, 163.5, 159.5, 159.3, 159.1, 159.0, 158.6, 158.2, 157.9, 157.8, 157.6, 157.1, 156.7, 156.6, 144.0, 143.7, 142.3, 142.1, 141.6, 141.5, 138.4, 138.3, 137.3, 137.2, 137.0, 136.9, 136.8, 136.7, 134.5, 134.4, 131.6, 131.4, 131.3, 130.2, 130.1, 129.3, 129.1, 128.9, 128.8, 128.7, 128.6, 128.5, 128.4, 128.2, 128.1 (×2), 127.5, 126.7, 126.1, 122.7 (×2), 122.6, 122.4, 120.1, 119.9, 119.8 (×2), 119.7, 119.6, 115.1, 114.7, 114.5, 114.3, 113.8, 113.7, 113.6, 113.5, 112.7, 112.0, 106.1, 105.7, 68.8, 68.0, 67.6, 67.4, 65.2, 65.1, 62.9, 62.7, 61.4, 60.9, 55.4, 55.3, 53.5, 53.4, 44.9, 44.3, 41.0, 37.3, 36.1, 36.0, 35.7 (×2), 35.2, 35.1, 33.4, 33.2, 31.7 (×2), 31.6 (×2), 30.4, 30.3, 30.2, 29.4, 29.3, 29.2, 29.1, 29.0 (×2), 28.9, 28.7, 28.6, 28.2, 28.1, 26.1, 26.0 (×2), 25.9. LR-ESI-MS m/z = 1123.6 $[M+H]^+$ (calc. for $C_{73}H_{83}N_6O_5$ 1123.6).

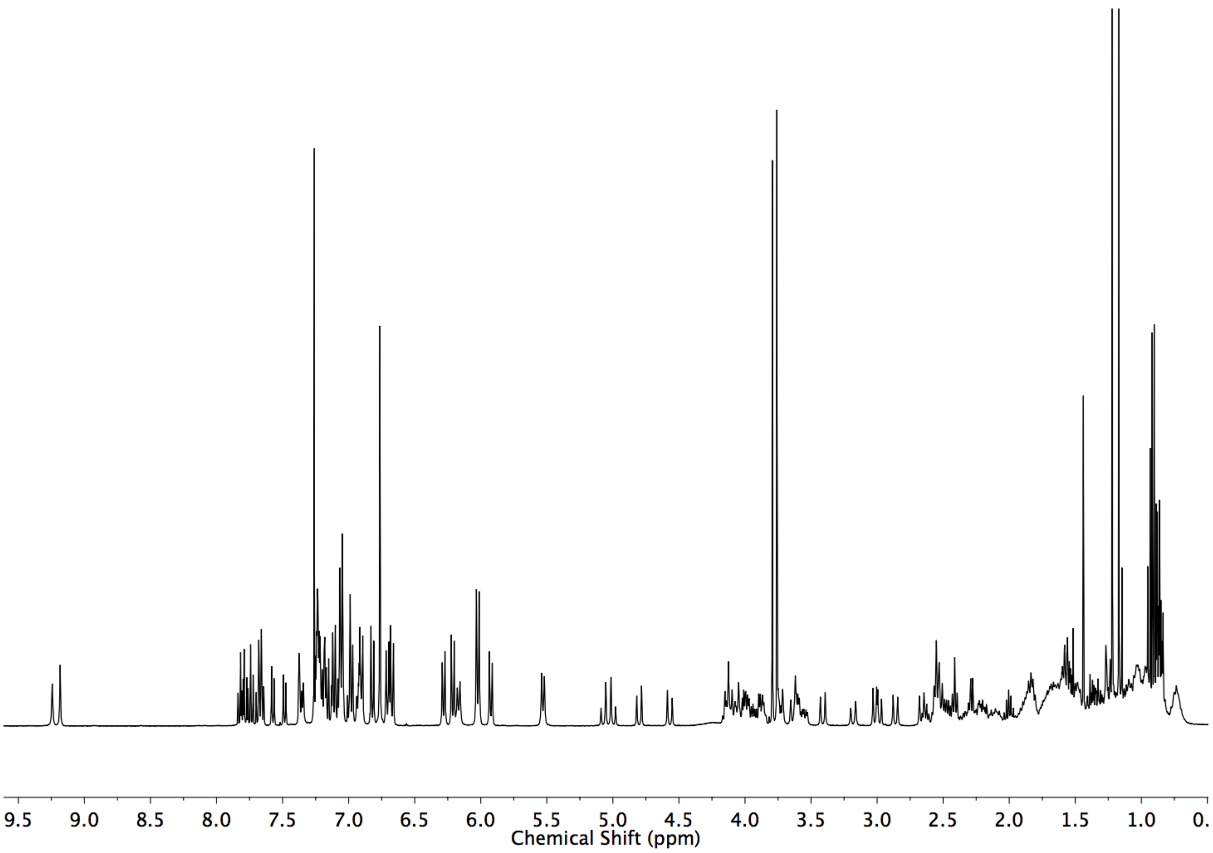

Figure S57 ^{1}H NMR (400 MHz, $CDCl_3$) of (R,S/R_{mt})-**3a**.

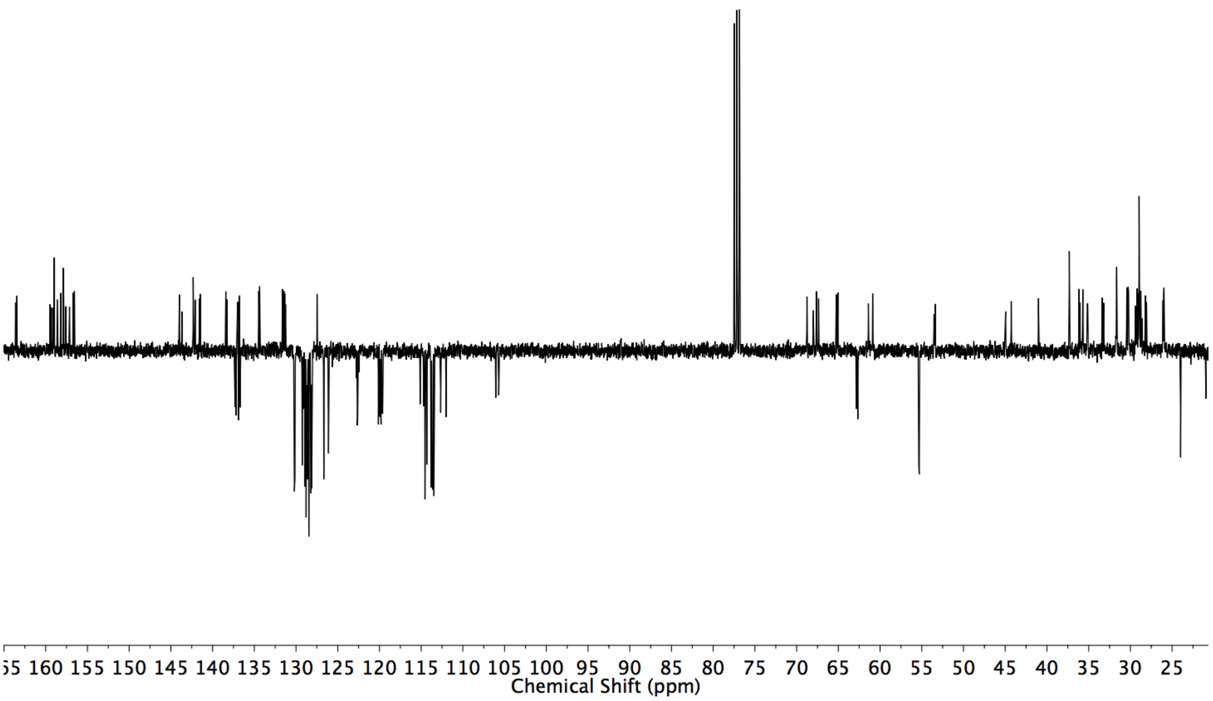

Figure S58 JMOD NMR (101 MHz, $CDCl_3$) of (R,S/R_{mt})-**3a**.

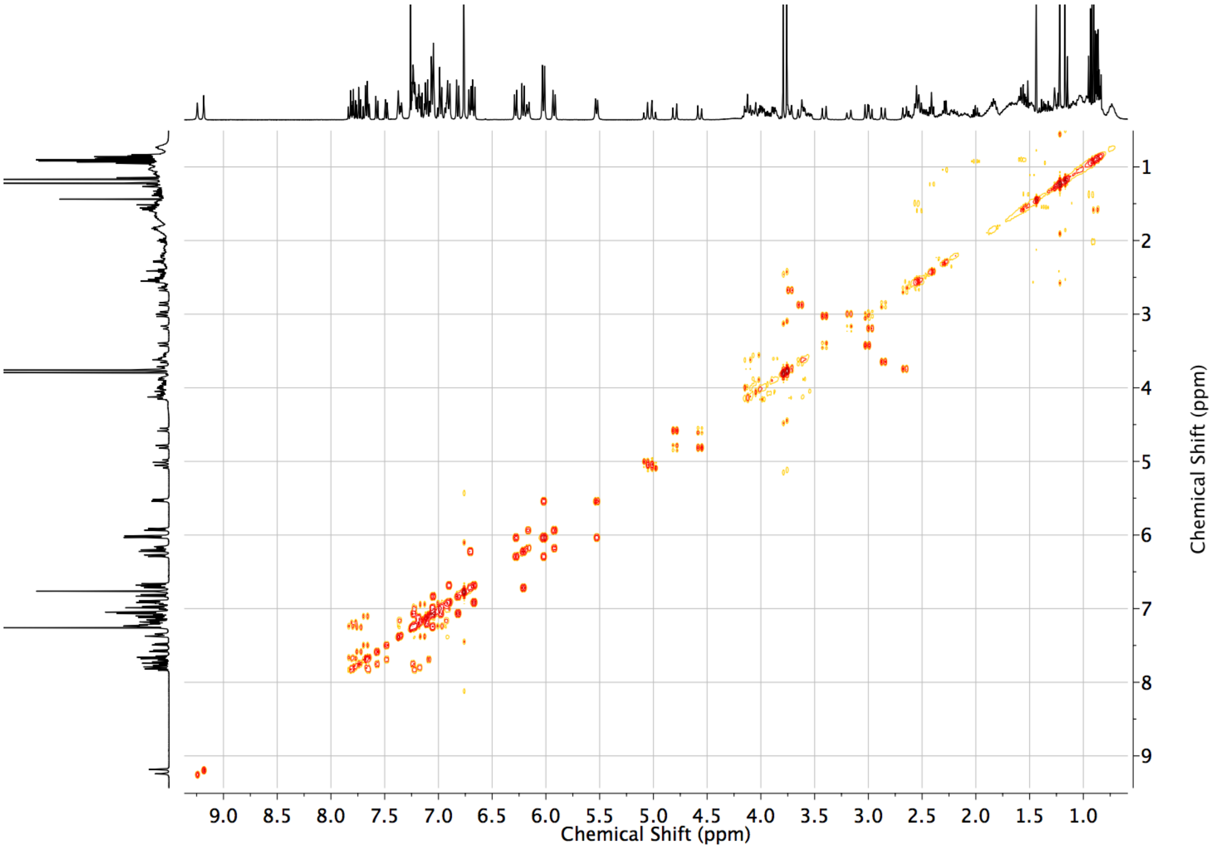

Figure S59 COSY NMR ($CDCl_3$) of (*R*,*S*/*R*$_{mt}$)-**3a**.

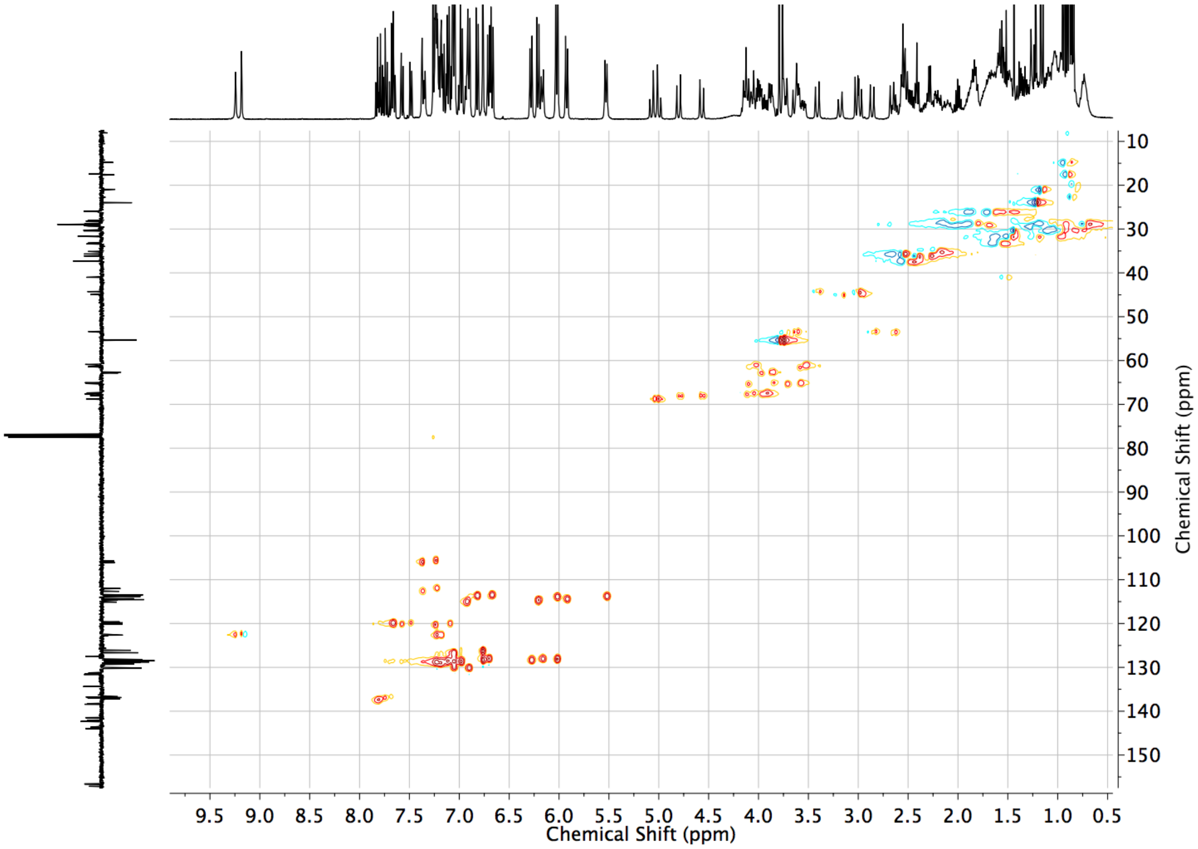

Figure S60 HSQC NMR ($CDCl_3$) of (*R*,*S*/*R*$_{mt}$)-**3a**.

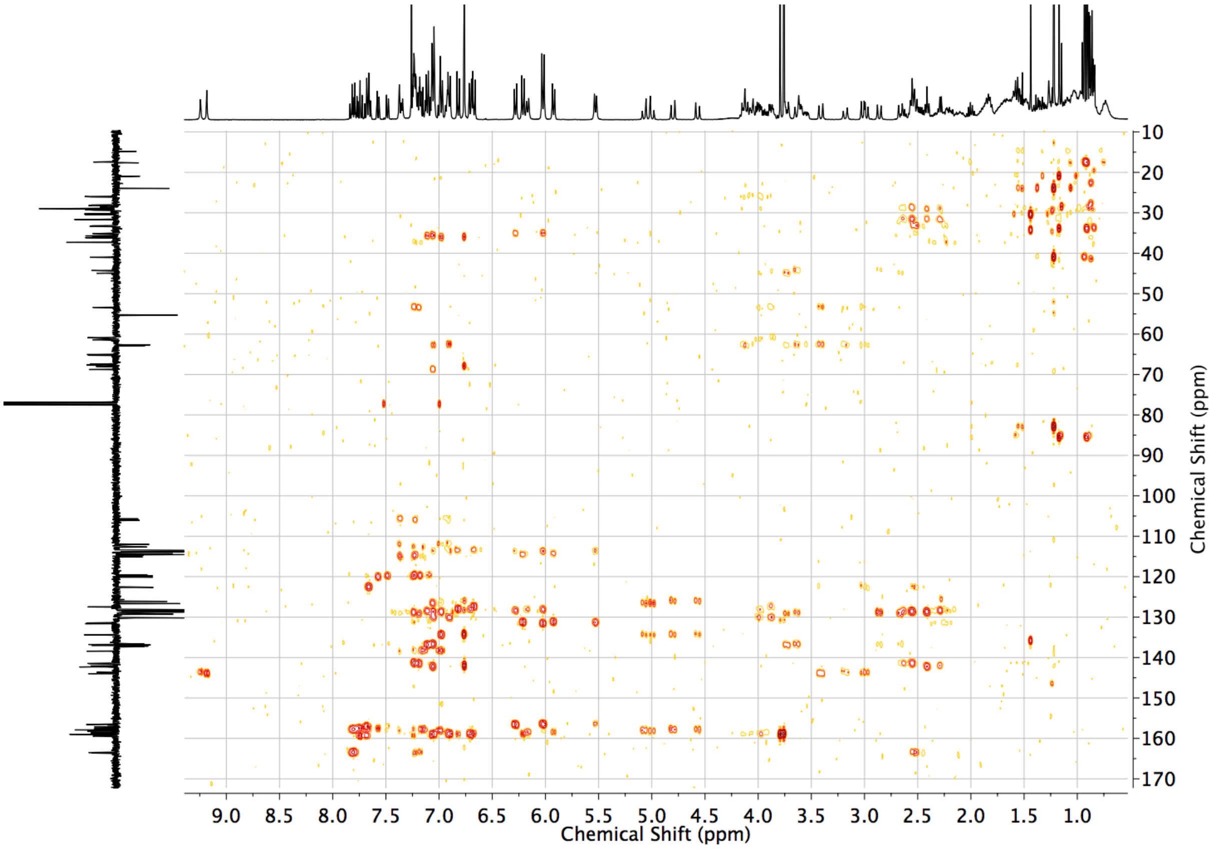

Figure S61 HMBC NMR ($CDCl_3$) of (*R*,*S*/*R*$_{mt}$)-**3a**.

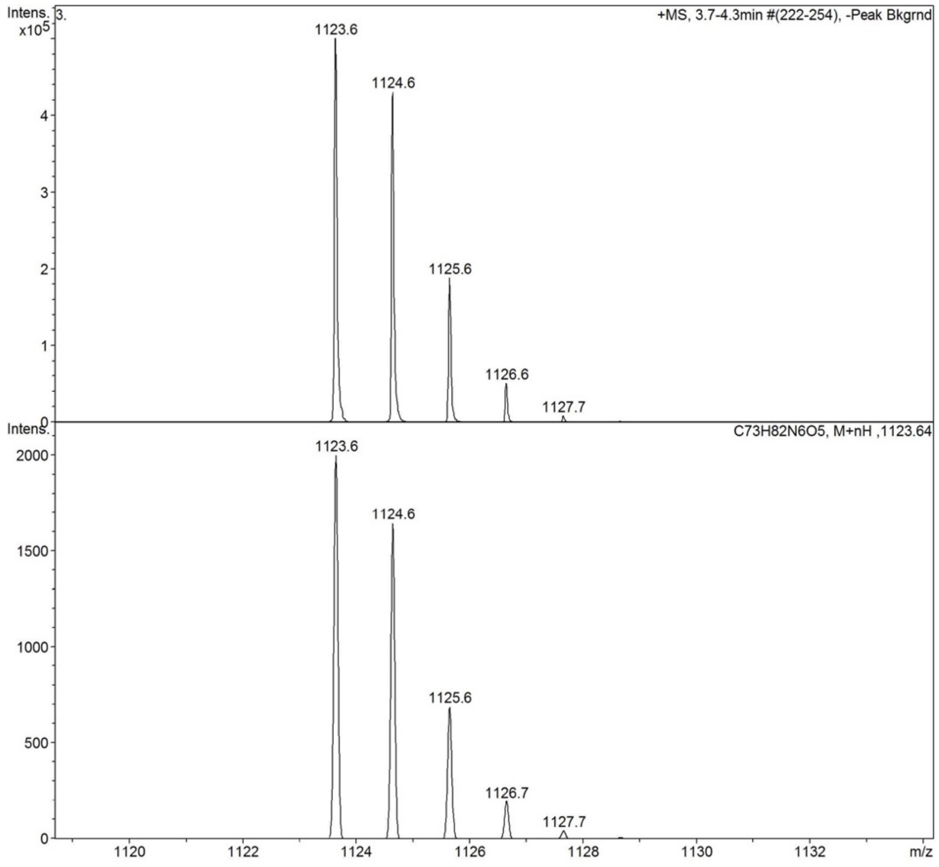

Figure S62 Observed (top) and calculated (bottom) isotopic patterns for (*R*,*S*/*R*$_{mt}$)-**3a**.

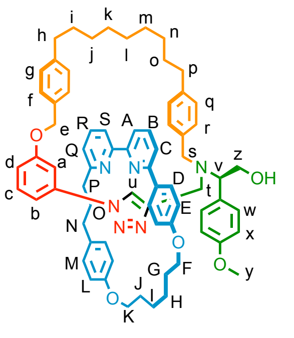

(*R*,S_{mt})-**3b**

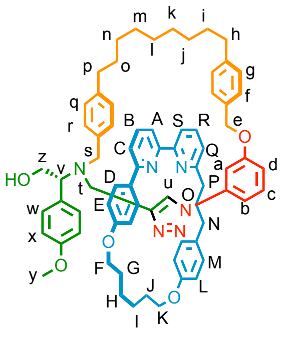

(*R*,R_{mt})-**3b**

(*R*,S_{mt})-**3b** and (*R*,R_{mt})-**3b**

To a solution of **2b** (116.2 mg, 0.25 mmol, 1.0 eq.), $[Cu(CH_3CN)_4]PF_6$ (92.8 mg, 0.249 mmol, 1.0 eq.), $^{i}Pr_2NEt$ (87 µL, 0.50 mmol, 2 eq.) in 1:1 $CHCl_3$/EtOH (10 mL) at 60 °C was added (*R*)-**1** (194.0 mg, 0.60 mmol, 1.2 eq.) in 1:1 $CHCl_3$/EtOH (15 mL) over 4 h. After removal of the solvent *in vacuo*, the residue was dissolved in 1:1 CH_2Cl_2/MeOH (10 mL) and KCN (160 mg, 2.5 mmol, 10 eq.) added as a solid. After stirring at r.t. for 30 minutes the solvent was removed under a flow of air. The residue was dissolved in CH_2Cl_2 (50 mL) and washed with H_2O (4 × 10 mL), dried ($MgSO_4$) and the solvent removed *in vacuo*. The residue containing catenane (*R*,*R*/S_{mt})-**3b** (in a 0.67:0.33 diastereoisomeric ratio, **Figure S41**) was purified by column chromatography on silica (Petrol/CH_2Cl_2/EtOAc/Et_2O 140/30/15/15), to yield (*R*,S_{mt})-**3b** (160 mg, 57%) and (*R*,R_{mt})-**3b** (90 mg, with 5% of (*R*,S_{mt})-**3b**, 32%) as white foams .

Catenane (*R*,S_{mt})-**3b**. ^{1}H NMR (500 MHz, $CDCl_3$) δ : 9.59 (br. s, 1H, H_u), 7.81 (t, J = 7.8, 1H, H_R), 7.74 (t, J = 7.8, 1H, H_B), 7.69 (dd, J = 7.8, 1.0, 1H, H_S), 7.57 (dd, J = 7.8, 1.0, 1H, H_A), 7.35-7.29 (m, 3H, Hc, H_r), 7.27 (dd, J = 7.8, 0.9, 1H, H_C), 7.21 (dd, J = 7.8, 0.9, 1H, H_Q), 7.13-7.07 (m, 5H, H_q, H_f, H_a), 7.04 (d, J = 8.4, 2H, H_g), 6.90 (d, J = 8.7, 2H, H_w), 6.88-6.81 (m, 2H, H_b, H_c), 6.74 (d, J = 8.7, 2H, H_D), 6.58 (d, J = 8.7, 2H, H_x), 6.42 (d, J = 8.7, 2H, H_E), 5.94 (d, J = 7.8, 2H, H_M), 5.35 (d, J = 7.5, 2H, H_L), 5.22-5.15 (m, 2H, H_e), 4.30-4.21 (m, 1H, one of H_F), 4.19-4.04 (m, 2H, one of H_z, one of H_F), 3.95 (dd, J = 11.2, 4.5, 1H, H_v), 3.85 (d, J = 13.6, 1H, one of H_s), 3.75-3.72 (m, 4H, 1 of H_K, H_y), 3.70-3.63 (m, 1H, one of H_K), 3.63-6.56 (m, 1H, 1 of H_z), 3.37 (d, J = 14.4, 1H, one of H_t), 3.08 (d, J = 14.4, 1H, one of H_t), 2.79 (d, J = 13.7, 1H, one of H_s), 2.67-2.59 (m, 1H, one of H_P), 2.56 (t, J = 6.9, 2H, H_h), 2.54- 2.38 (m, 1H, one of H_P), 2.32- 2.24 (m, 1H, one of H_N), 2.13- 2.04 (m, 1H, one of H_N), 2.04-1.97 (m, 1H, one of H_G), 1.97-1.90 (m, 1H, one of H_G), 1.90-1.73 (m, 6H, H_J, H_H, H_I), 1.67-1.55 (m, 4H, H_i, H_j), 1.49 (q, J = 6.9, 2H, H_O), 1.39-1.22 (m, 4H, H_n, H_o), 1.19-1.10 (m, 2H, H_k), 1.10-1.00 (m, 2H, H_m), 0.81-0.68 (m, 2H, H_l). ^{13}C NMR (126 MHz, $CDCl_3$) δ : 163.3, 159.9, 159.5, 159.0, 158.1, 157.9, 157.6, 156.8, 143.2, 142.2, 141.6, 138.5, 137.3, 136.9, 136.8, 134.5, 132.0, 131.4, 130.2, 129.1, 128.9, 128.8 (×2), 128.0, 127.9, 127.2, 126.1, 123.4, 122.8, 120.4, 120.1, 119.8, 116.2, 114.9, 114.2, 113.4, 111.8, 105.2, 69.9, 68.7, 65.3, 62.5, 60.6, 55.3, 52.7, 44.7, 37.2, 36.2, 35.8, 35.1, 33.2, 31.7, 31.6, 30.5, 30.4, 29.7, 29.3, 29.2, 28.8 (×2), 25.3, 24.8. LR-ESI-MS *m/z* = 1109.64 $[M+H]^+$ (calc. for $C_{72}H_{81}N_6O_5$ 1109.63).

Catenane (*R*,R_{mt})-**3b**. ^{1}H NMR (500 MHz, $CDCl_3$) δ : 9.66 (s, 1H, H_u), 7.80 (t, J = 7.7, 1H, H_R), 7.68 (t, J = 7.7, 1H, H_B), 7.64 (d, J = 7.8, 1H, H_S), 7.47 (dd, J = 7.8, 0.9, 1H, H_A), 7.42 (br. s, 1H, H_a), 7.37 (dd, J = 7.8, 0.9, 1H, H_d), 7.24 (d, J = 7.7, 0.9, 1H, H_Q), 7.17 (d, J = 7.7, 2H, H_r), 7.14-7.03 (m, 7H, H_C, H_c, H_g, H_q), 6.94-6.86 (m, 1H, H_b), 6.87-6.76 (m, 6H, H_f, H_w, H_x), 6.25 (d, J = 7.8, 2H, H_M), 6.13 (d, J = 8.2, 4H, H_D or H_E, H_L), 5.94 (d, J = 8.1, 2H, H_D or H_E), 4.82 (d, J = 14.4, 1H, one of H_e), 4.62 (d, J = 14.4, 1H, one of H_e), 4.28-4.18 (m, 1H, one of H_K), 4.15 (app. t, J = 10.8, 1H, H_F), 4.10-4.00 (m, 2H, one of H_z, H_v), 4.00-3.93 (m, 2H, 1 of H_K, one of H_z), 3.80 (s, 3H, H_y), 3.75 (d, J = 13.8, 1H, H_s), 3.61 (dd, J = 10.5, 4.3, 1H, H_F), 3.25 (d, J = 15.0, 1H, H_t), 3.12 (d, J = 15.0, 1H, H_t), 2.68-2.48 (m, 5H, H_P, H_h, one of H_s), 2.39-2.21 (m, 2H, H_N), 2.05-1.75 (m, 8H, H_G, H_H, H_I, H_J), 1.66-1.45 (m, 6H, H_O, H_i, H_o), 1.25-0.95 (m, 8H, H_j, H_k, H_m, H_n), 0.90-0.75 (m, 2H, H_l). ^{13}C NMR (126 MHz, $CDCl_3$) **δ** 163.3, 159.6, 159.2, 159.0, 157.8, 157.5, 156.9, 156.7, 143.3, 141.9, 141.5, 138.3, 137.3, 136.7, 136.6, 134.3, 131.8, 131.7, 130.1, 129.3, 128.7, 128.5, 128.3 (×2), 127.9 127.8, 125.8, 123.4, 122.7, 120.1, 119.7, 119.6, 115.9, 114.9, 114.3, 113.5, 112.8, 106.0, 69.9, 67.8, 66.0, 62.4, 61.1, 55.2, 53.3, 45.2, 37.2, 35.9, 35.7, 35.0, 33.0, 31.6 (×2), 30.2, 30.1, 29.4, 29.2, 29.0, 28.8 (×2), 25.2, 24.6. LR-ESI-MS *m/z* = 1109.64 $[M+H]^+$ (calc. for $C_{72}H_{81}N_6O_5$ 1109.63).

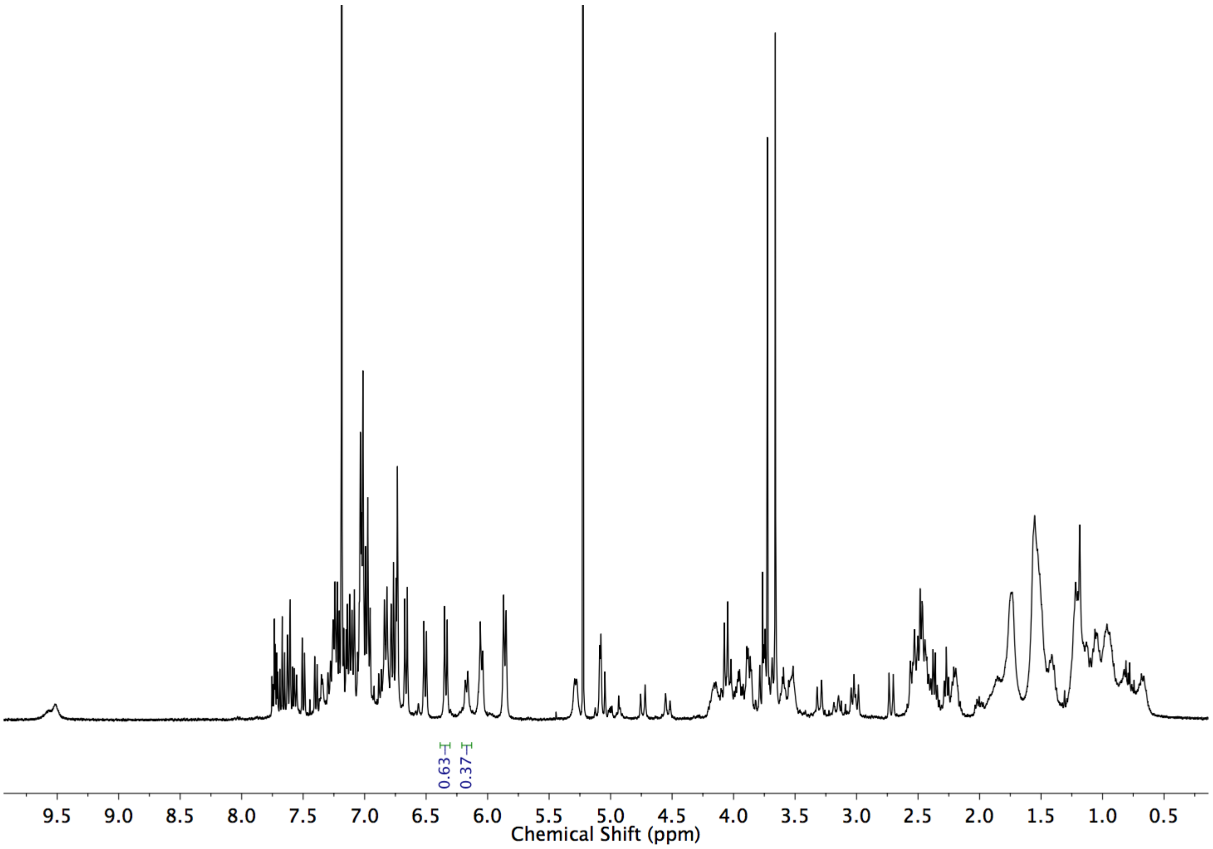

Figure S63 ^{1}H NMR (400 MHz, $CDCl_3$) of (*R*,*R*/S_{mt})-**3b** prior to purification by chormatography.

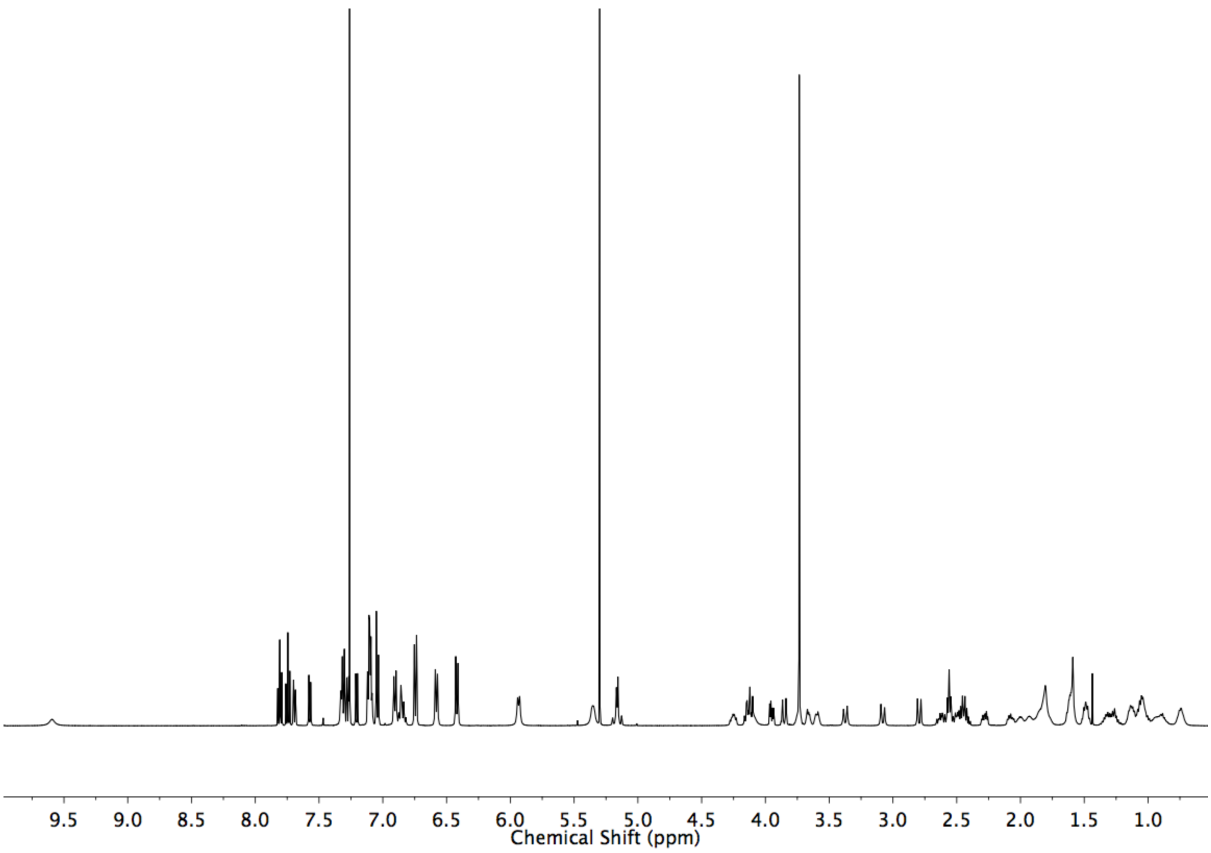

Figure S64 ^{1}H NMR (500 MHz, $CDCl_3$) of (R,S_{mt})-**3b**.

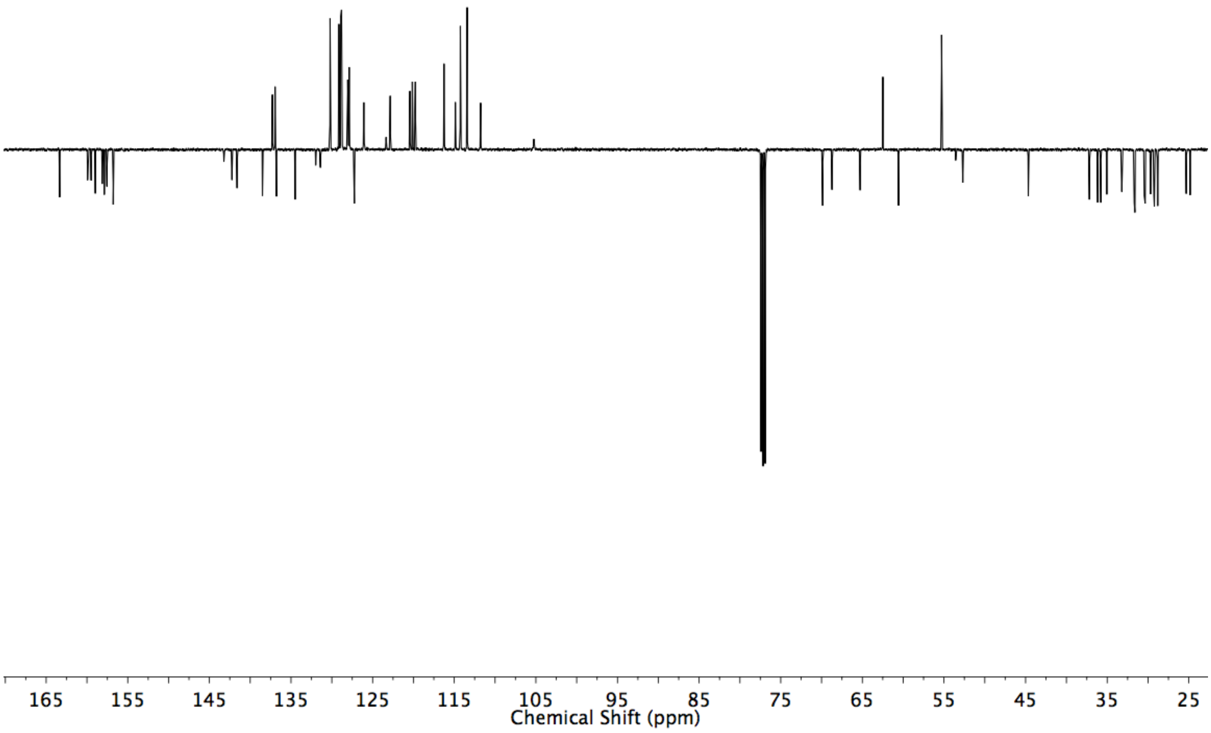

Figure S65 JMOD NMR (126 MHz, $CDCl_3$) of (R,S_{mt})-**3b**.

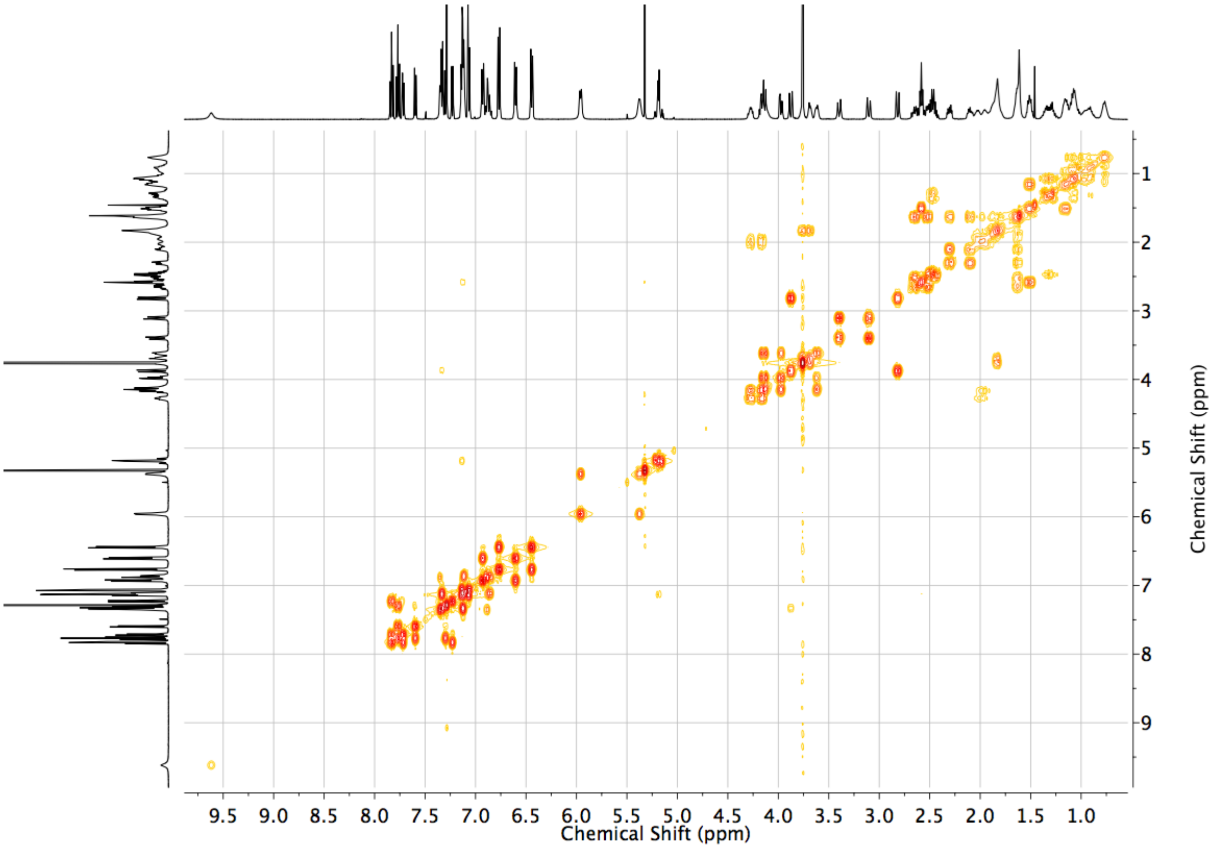

Figure S66 COSY NMR ($CDCl_3$) of (*R*,S_{mt})-**3b**.

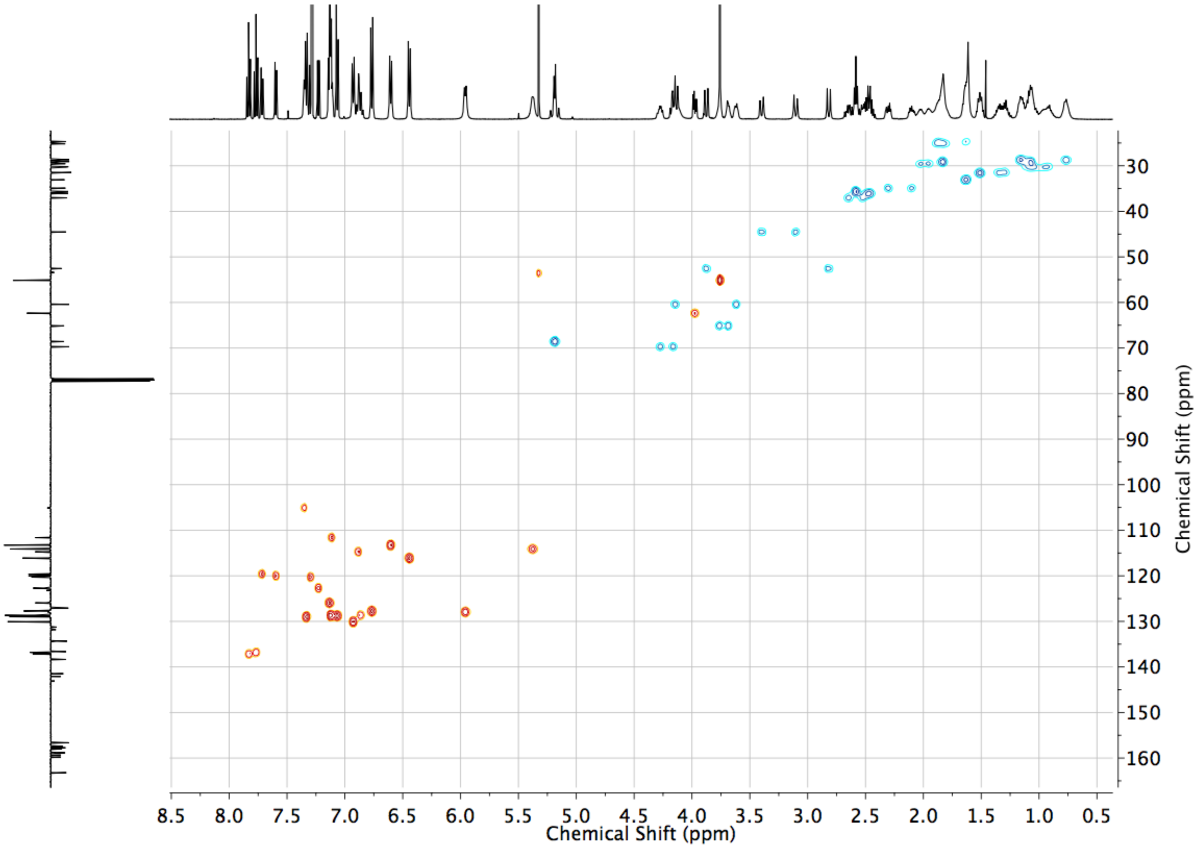

Figure S67 HSQC NMR ($CDCl_3$) of (*R*,S_{mt})-**3b**.

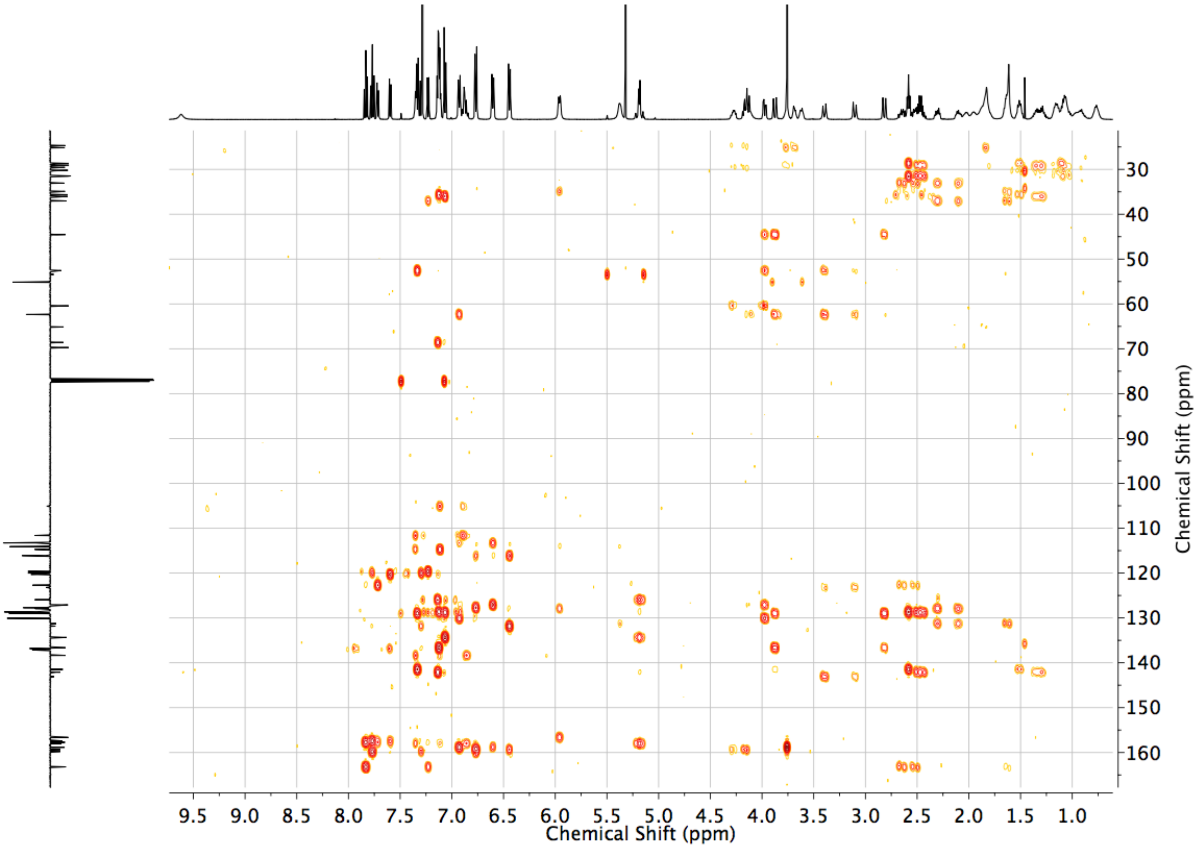

Figure S68 HMBC NMR ($CDCl_3$) of (*R*,S_{mt})-**3b**.

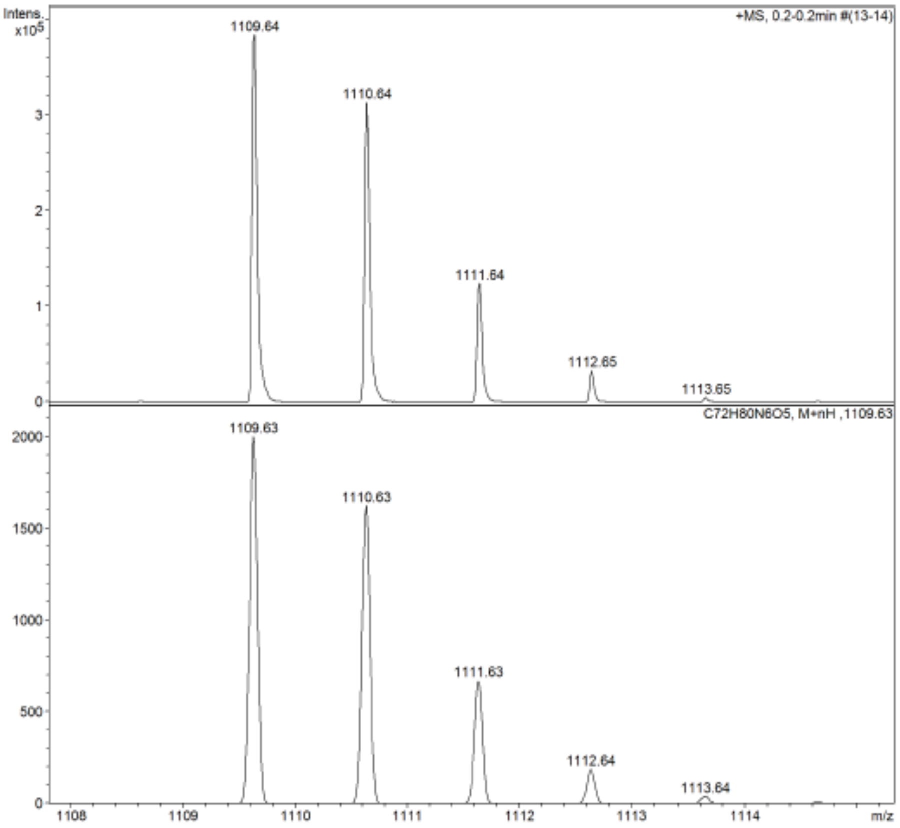

Figure S69 Observed (top) and calculated (bottom) isotopic patterns for (*R*,S_{mt})-**3b**.

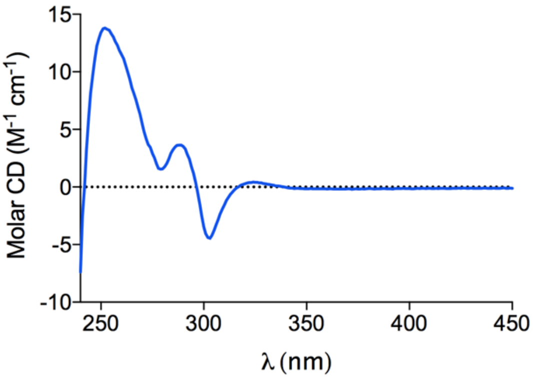

Figure S70 Circular dichroism spectrum of (*R*,S_{mt})-**3b** (35.0 μM in $CHCl_3$, 293 K).

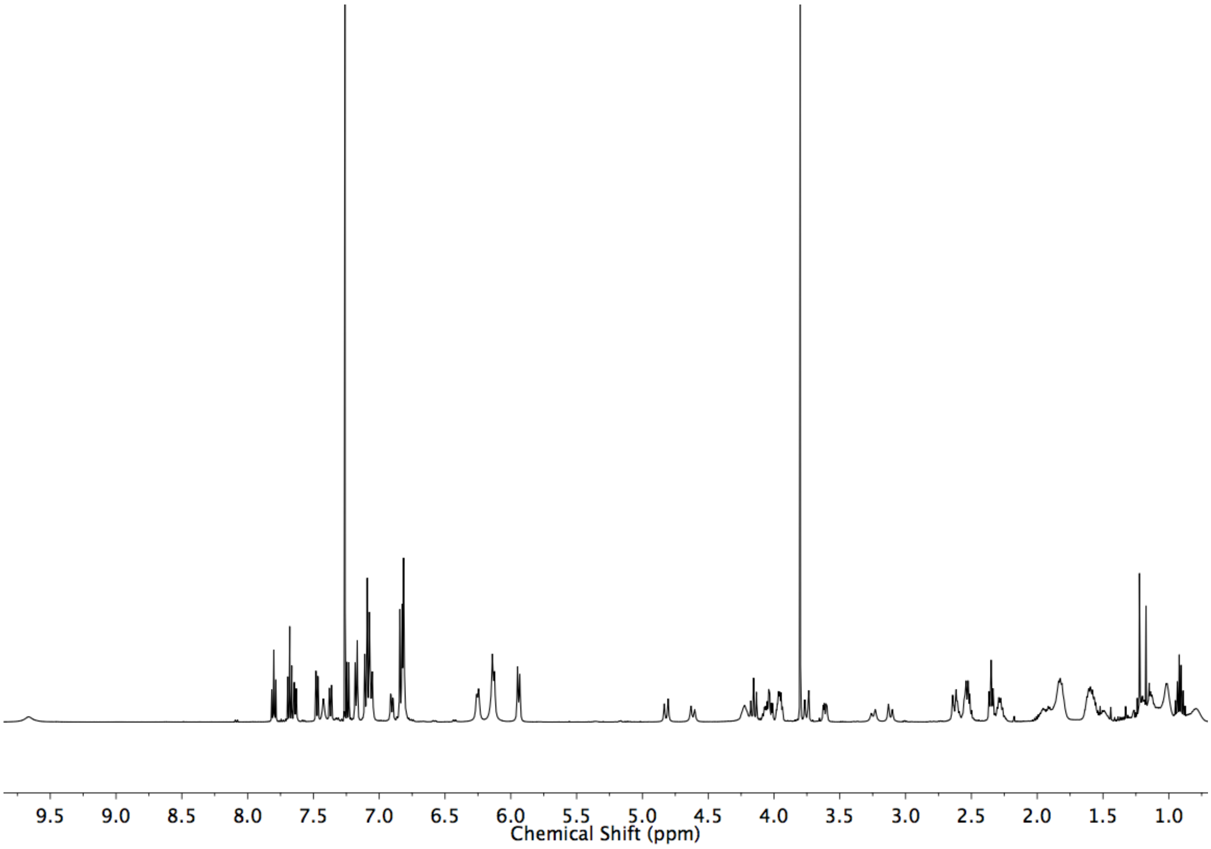

Figure S71 ^{1}H NMR (500 MHz, $CDCl_3$) of (*R*,R_{mt})-**3b**.

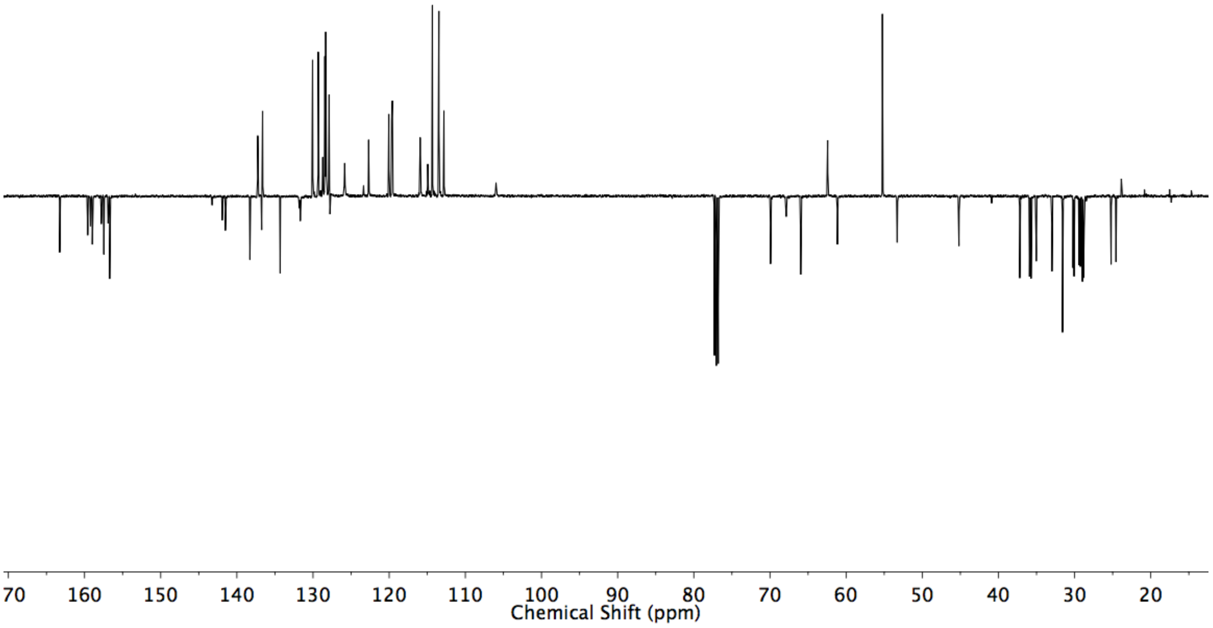

Figure 72 JMOD NMR (126 MHz, $CDCl_3$) of (*R*,R_{mt})-**3b**.

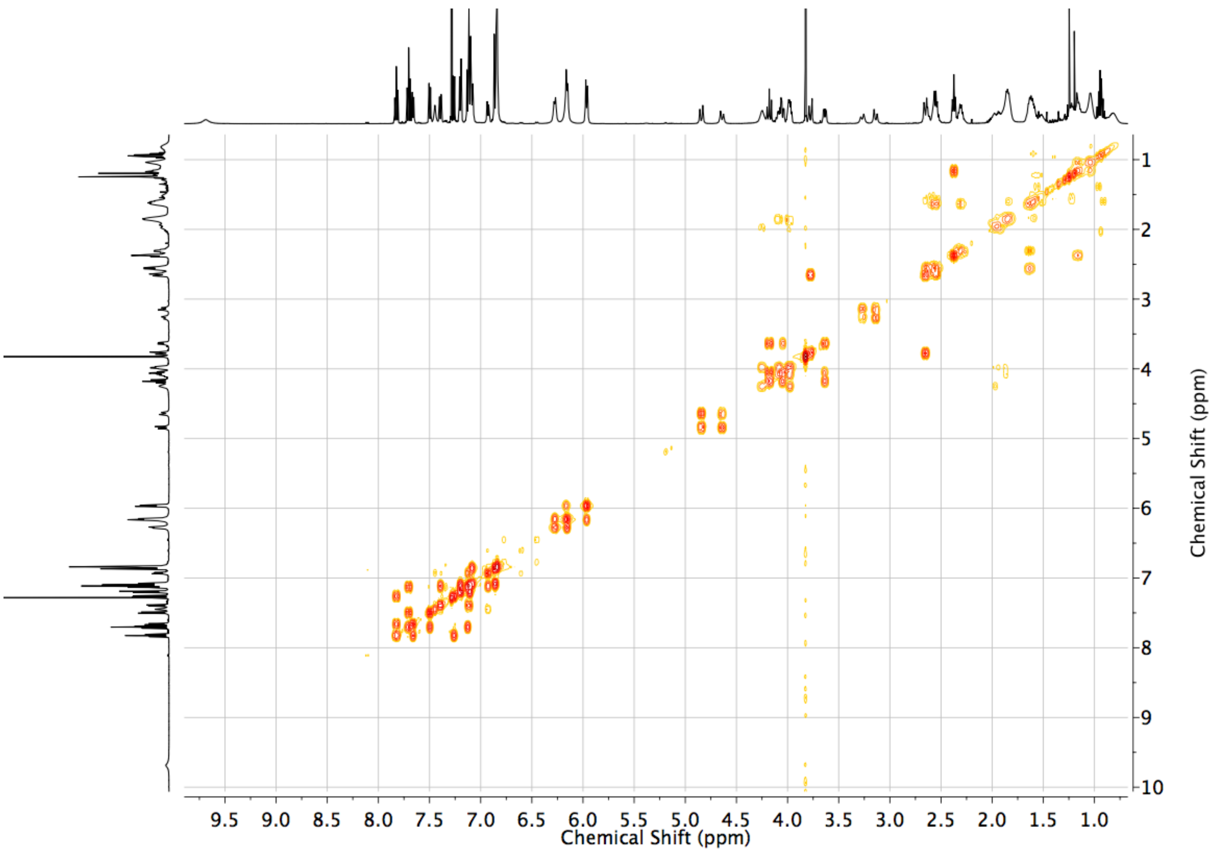

Figure S73 COSY NMR ($CDCl_3$) of (*R*,R_{mt})-**3b**.

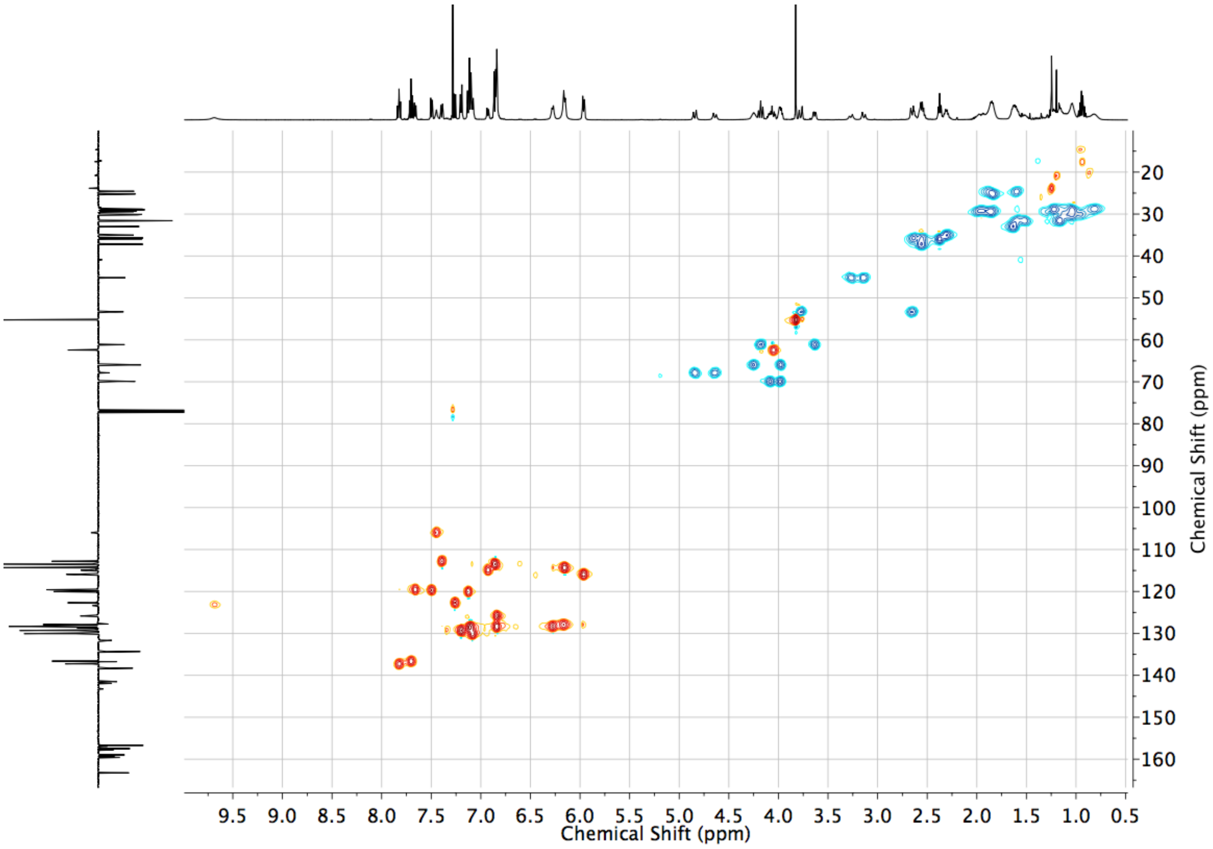

Figure S74 HSQC NMR ($CDCl_3$) of (R,R_{mt})-**3b**.

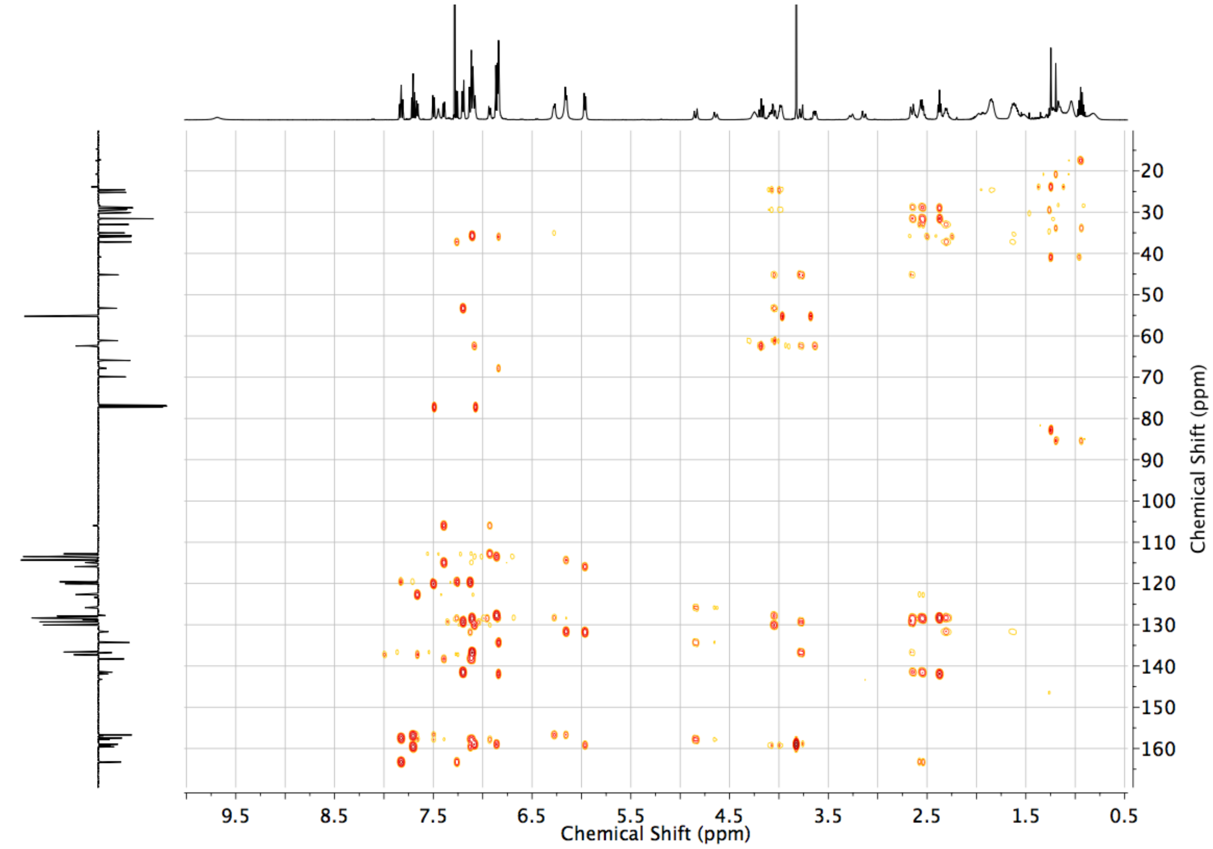

Figure S75 HMBC NMR ($CDCl_3$) of (R,R_{mt})-**3b**.

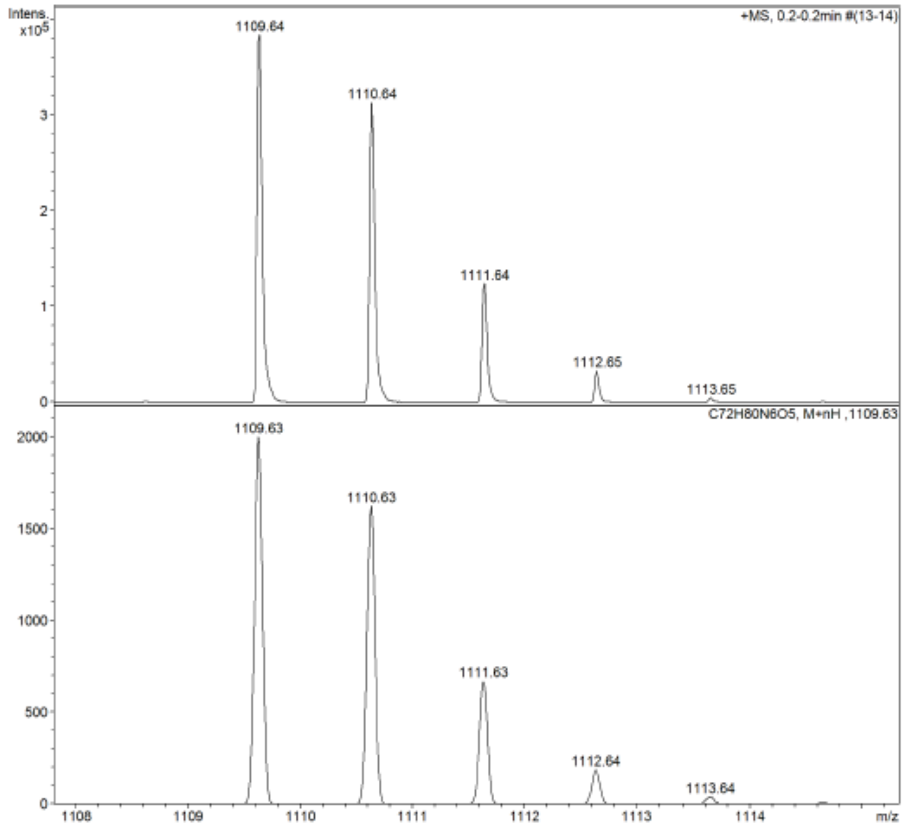

Figure S76 Observed (top) and calculated (bottom) isotopic patterns for (R,R_{mt})-**3b**.

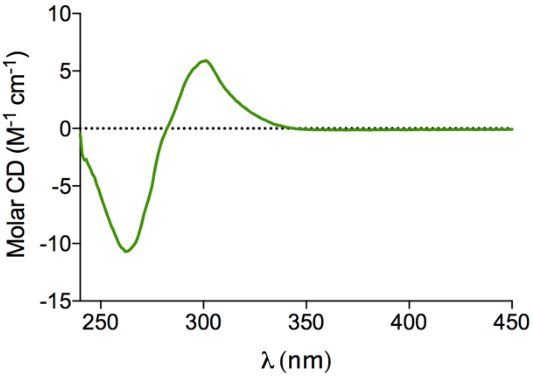

Figure S77 Circular dichroism spectrum of (R,R_{mt})-**3b** (35.0 μM in $CHCl_3$, 293 K).

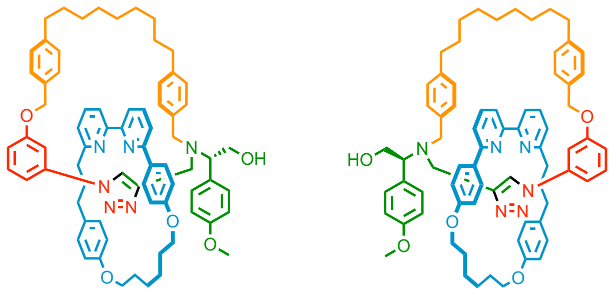

(S,S_{mt})-**3b** and (S,R_{mt})-**3b**

To a solution of **2b** (288 mg, 0.62 mmol, 1.0 eq.), [Cu(CH_3CN)$_4$]PF_6 (231 mg, 0.62 mmol, 1.0 eq.), ^{i}Pr$_2$NEt (218 µL, 1.24 mmol, 2 eq.) in 1:1 $CHCl_3$/EtOH (25 mL) at 60 °C was added (S)-**1** (400 mg, 0.62 mmol, 1.2 eq.) in 1:1 $CHCl_3$/EtOH (24 mL) over 4 h. After removal of the solvent *in vacuo*, the residue was dissolved in 1:1 CH_2Cl_2/MeOH (20 mL) and KCN (320 mg, 5 mmol, 8 eq.) added as a solid. After stirring at r.t. for 30 minutes the solvent was removed under a flow of air. The residue was dissolved in CH_2Cl_2 (100 mL) and washed with H_2O (4 × 20 mL), dried ($MgSO_4$) and the solvent removed *in vacuo*. The residue containing catenane (S,R/S_{mt})-**3b** (in a 0.67:0.33 diastereoisomeric ratio) was purified by column chromatography on silica (Petrol/CH_2Cl_2/EtOAc/Et_2O 140/30/15/15), to yield (S,R_{mt})-**3b** (300 mg, 44%) and (S,S_{mt})-**3b** (200 mg, with 3% of (S,R_{mt})-**3b**, 30%) as a white foams. Spectroscopic data were identical to those reported for (R,S_{mt})-**3b** and (R,R_{mt})-**3b** respectively with the exception of the circular dichroism spectra.

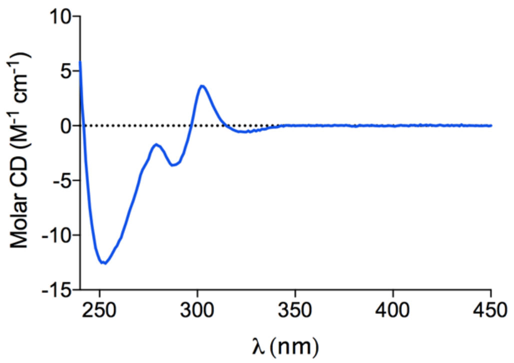

Figure S78 Circular dichroism spectrum of (S,R_{mt})-**3b** (35.0 µM in $CHCl_3$, 293K).

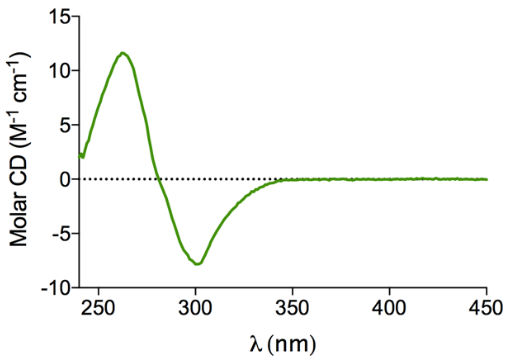

Figure S79 Circular dichroism spectrum of (S,S_{mt})-**3b** (35.0 µM in $CHCl_3$, 293 K).

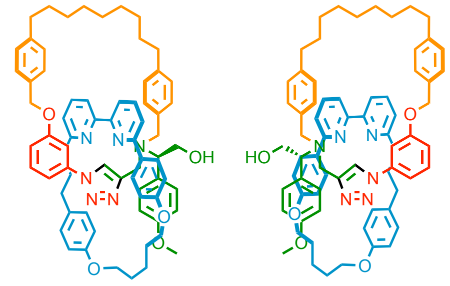

(R,S_{mt})-**3c** (R,R_{mt})-**3c**

(R,R/S_{mt})-**3c**

To a solution of **2c** (11.3 mg, 0.025 mmol, 1 eq.), $[Cu(CH_3CN)_4]PF_6$ (8.9 mg, 0.024 mmol, 0.96 eq.), $^{i}Pr_2NEt$ (9 μL, 0.050 mmol, 2 eq.) in 1:1 $CHCl_3$/EtOH (2.5 mL) at 60 °C was added (R)-**1** (16.1 mg, 0.025 mmol, 1 eq.) in 1:1 $CHCl_3$/EtOH (1.0 mL) over 4 h. After removal of the solvent *in vacuo*, the residue was dissolved in 1:1 CH_2Cl_2/MeOH (2.5 mL) and KCN (16 mg, 0.25 mmol, 10 eq.) added as a solid. After stirring at r.t. for 30 minutes the solvent was removed under a flow of air. The residue was dissolved in CH_2Cl_2 (5 mL) and washed with H_2O (4 × 5 mL), dried ($MgSO_4$) and the solvent removed *in vacuo*. Low conversion of **2c** was observed (~23%). The residue containing catenane (R,R/S_{mt})-**3c** in a 1:1 diastereoisomeric ratio (**Figure S80**) was not purified.

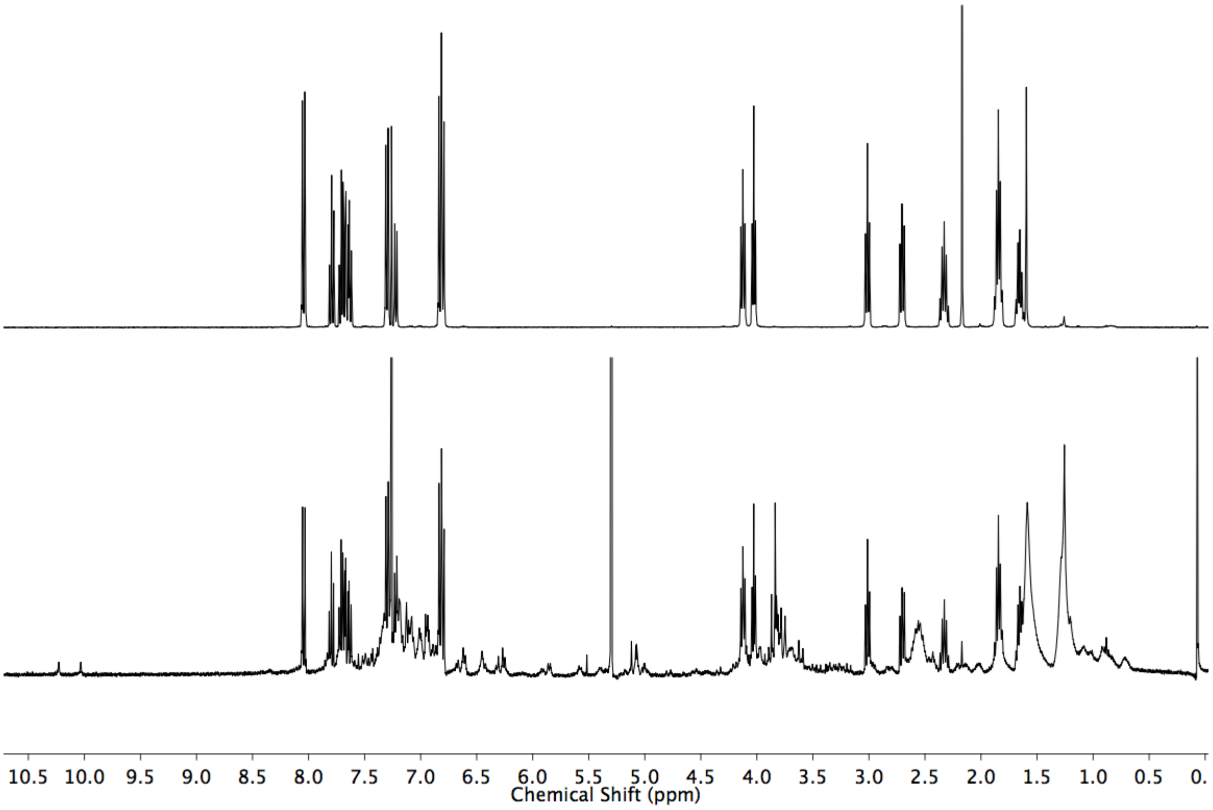

Figure S80 Stacked partial ^{1}H NMR (400 MHz, $CDCl_3$) spectra of **2c** (top) and crude **3c** reaction mixture.

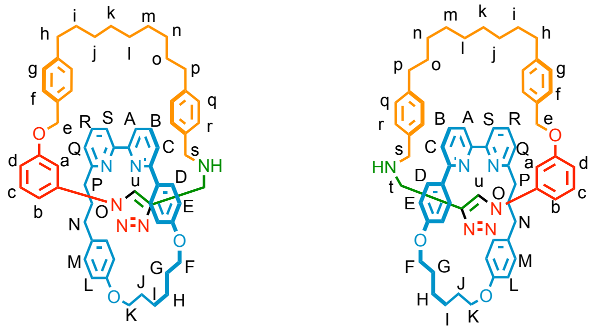

(S_{mt})-**6b** (R_{mt})-**6b**

rac-**6b**

To a solution of **2b** (23.2 mg, 0.050 mmol, 1 eq.), [Cu(CH_3CN)$_4$]PF_6 (18.5 mg, 0.0495 mmol, 0.99 eq.), $^{i}Pr_2NEt$ (18 µL, 0.10 mmol, 2 eq.) in 1:1 $CHCl_3$/EtOH (5.0 mL) at 60 °C was added **S11** (24.0 mg, 0.060 mmol, 1.2 eq.) in 1:1 $CHCl_3$/EtOH (2.4 mL) over 4 h. After removal of the solvent *in vacuo*, the residue was dissolved in 1:1 CH_2Cl_2/MeOH (2.5 mL) and KCN (16 mg, 0.25 mmol, 10 eq.) added as a solid. After stirring at r.t. for 30 minutes the solvent was removed under a flow of air. The residue was dissolved in CH_2Cl_2 (5 mL) and washed with H_2O (4 × 5 mL), dried ($MgSO_4$) and the solvent removed *in vacuo*. After purification by column chromatography on silica (CH_2Cl_2 with a gradient of 0 to 50% EtOAc) *rac*-**6b** was obtained as a white foam (33.0 mg, 70%). ^{1}H NMR (500 MHz, $CDCl_3$) **δ** : 9.29 (s, 1H, H_u), 7.56 (t, J = 7.8 Hz, 1H, H_R), 7.43-7.36 (m, 2H, H_B, H_a or H_b or H_c or H_d), 7.30 (m, 1H, H_a or H_b or H_c or H_d), 7.23 (dd, J = 5.1, 3.5 Hz, 1H, H_A), 7.21-7.12 (m, 3H, H_C, H_Q, H_S), 7.03-6.96 (m, 5H, H_q, H_r, H_a or H_b or H_c or H_d), 6.90 (d, J = 7.6 Hz, 2H, H_f), 6.88-6.83 (m, 2H, H_a or H_b or H_c or H_d), 6.79 (d, J = 8.0 Hz, 2H, H_g), 6.68 (d, J = 6.6 Hz, 2H, H_D), 6.44 (d, J = 7.9 Hz, 2H, H_M), 6.40 (d, J = 8.7 Hz, 2H, H_E), 6.33 (d, J = 8.7 Hz, 2H, H_L), 4.71 (m, 2H, H_e), 4.21-4.02 (m, 4H, H_F, H_K), 3.45 (d, J = 11.6 Hz, 1H, 1 of H_s), 3.36 (d, J = 12.1 Hz, 1H, 1 of H_s), 3.30 (d, J = 11.3 Hz, 1H, 1 of H_t), 3.13 (d, J = 11.8 Hz, 1H, 1 of H_t), 2.64 (td, J = 13.6, 4.9 Hz, 1H, 1 of H_P or H_N), 2.52 (t, J = 7.1 Hz, 2H, H_h or H_p), 2.50-2.42 (m, 3H, H_h or H_p), 2.40-2.28 (m, 3H, H_P or H_N), 1.94-1.87 (m, 2H, H_G or H_J), 1.86-1.78 (m, 2H, H_G or H_J), 1.76-1.51 (m, 6H, H_H, H_I, H_O), 1.48-1.31 (m, 4H, H_i, H_o), 1.21-1.04 (m, 8H, H_j, H_k, H_m, H_n), 1.04-0.80 (m, 2H, H_l). ^{13}C NMR (126 MHz, $CDCl_3$) **δ** 163.0, 159.4, 158.8 (×2), 157.3, 156.8, 156.7, 142.3, 140.7 [assigned by HMBC analysis], 138.3, 137.0, 136.6, 134.2, 132.5 (×2), 129.5, 128.9, 128.7 [assigned by HMBC analysis] 128.5 (×2), 128.4 (×2), 127.9, 127.1, 122.2, 120.3, 120.0, 119.3, 116.1, 114.2, 113.4, 112.1, 106.7, 105.4, 69.4, 69.3, 66.1, 53.7, 44.9, 36.9, 35.7, 35.5, 35.1, 32.3, 31.7, 31.4, 30.3, 30.2, 29.4, 29.3, 29.2, 28.9, 28.7, 25.3, 24.9. HR-ESI-MS m/z = 959.5591 [M+H]$^+$ (calc. for $C_{63}H_{71}N_6O_3$ 959.5582).

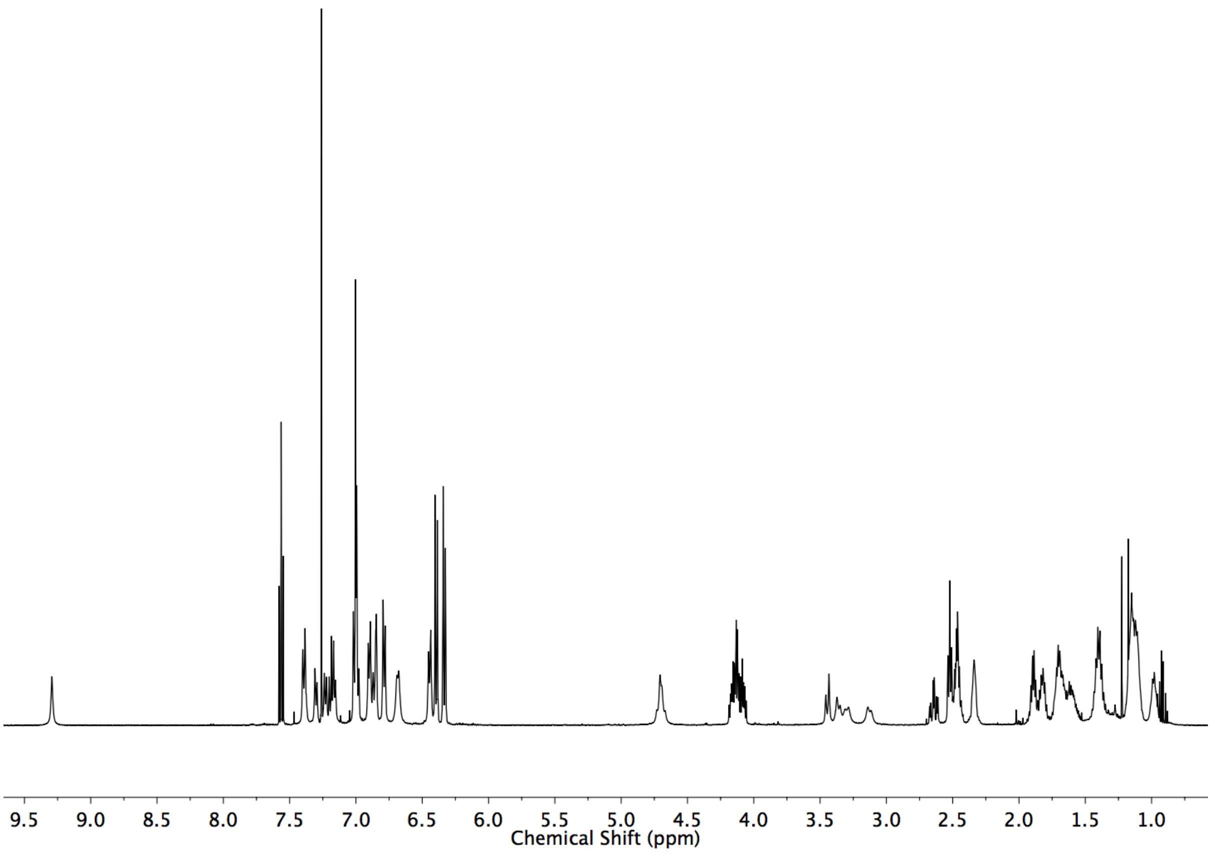

Figure S81 ^{1}H NMR (500 MHz, $CDCl_3$) of **6b**.

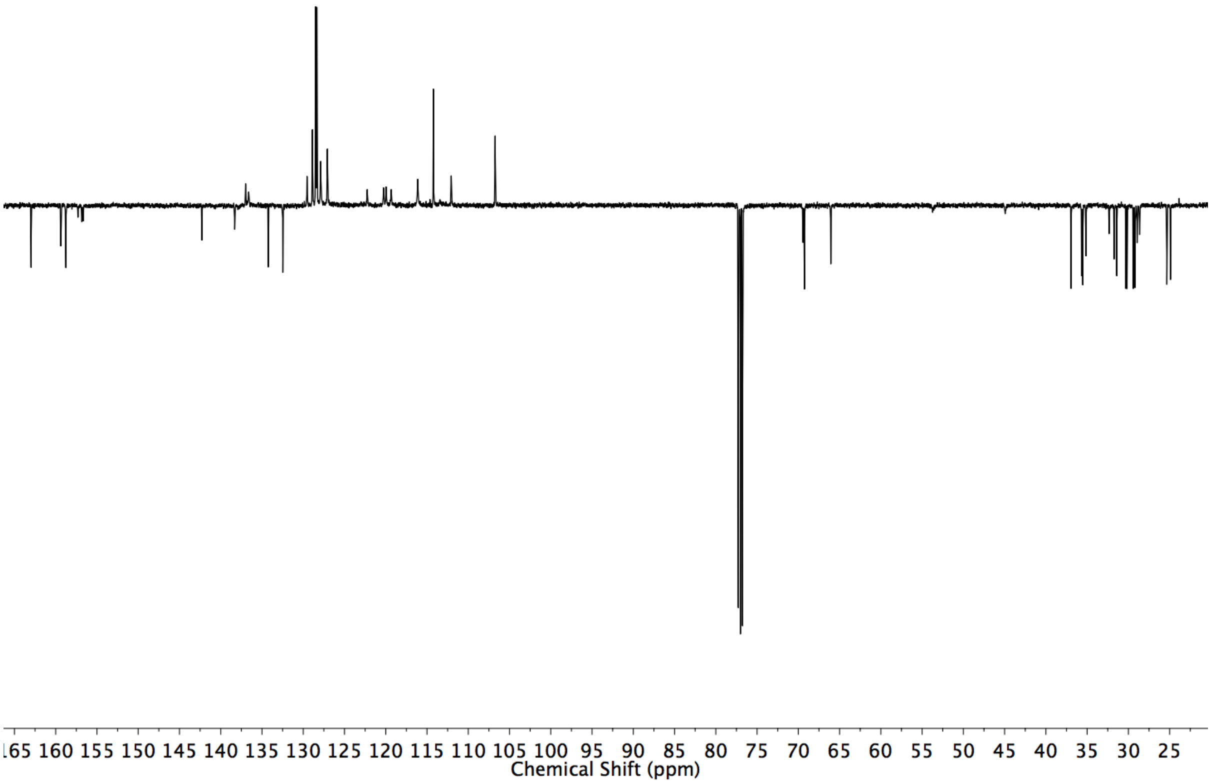

Figure S82 JMOD NMR (126 MHz, $CDCl_3$) of **6b**.

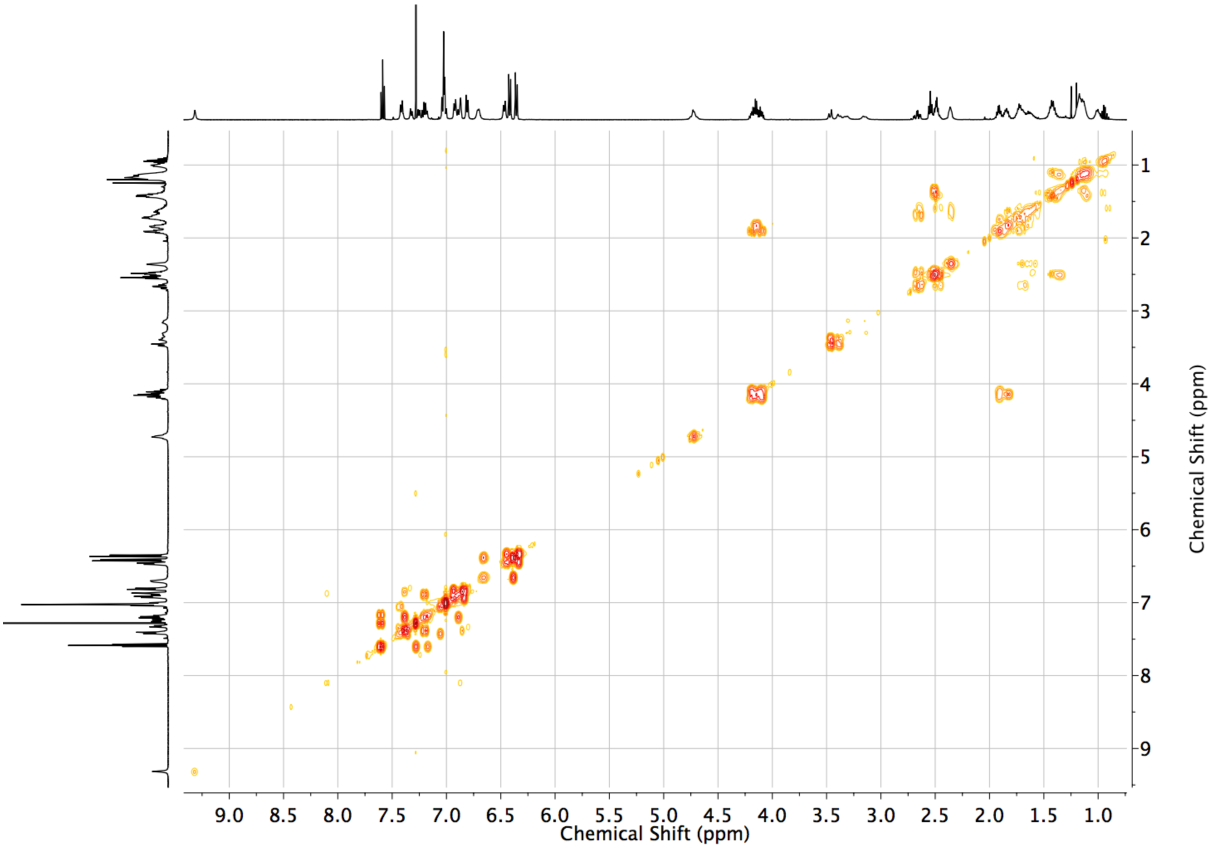

Figure S83 COSY NMR ($CDCl_3$) of **6b**.

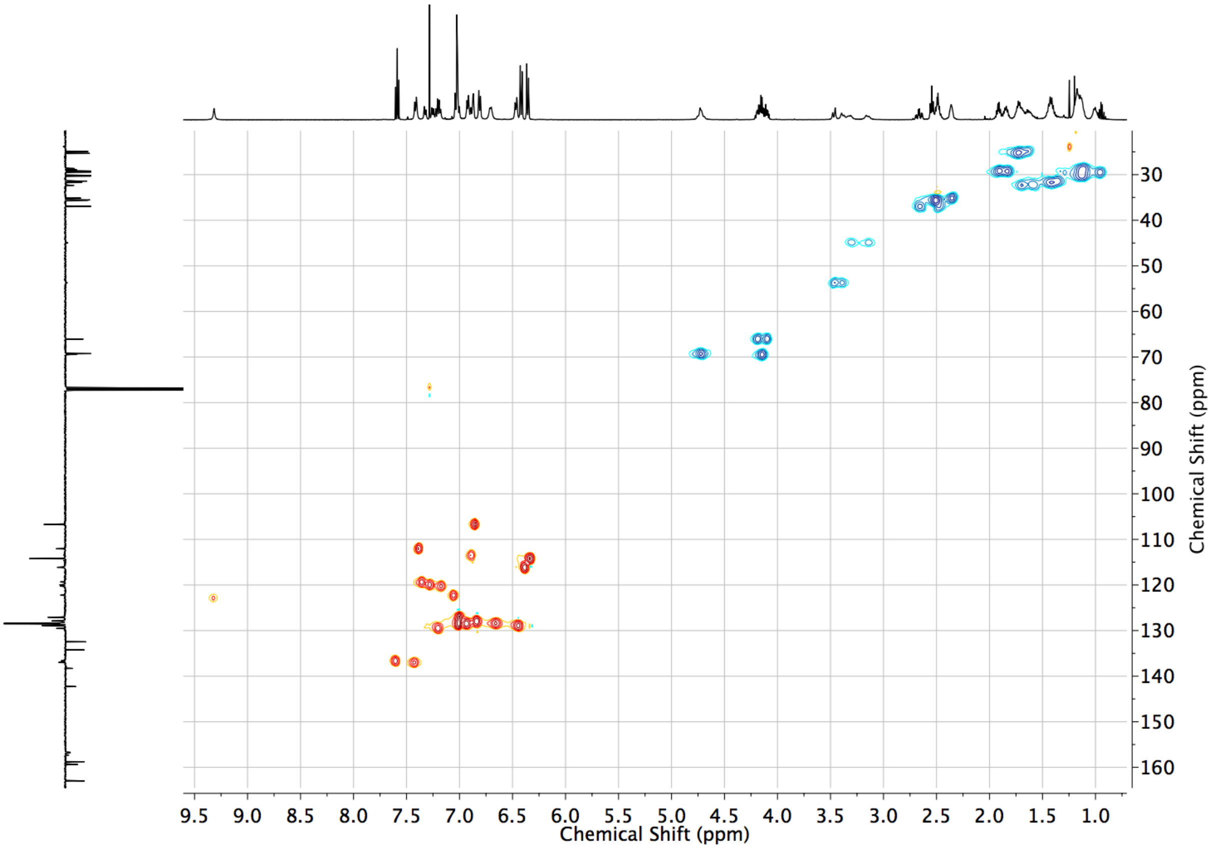

Figure S84 HSQC NMR ($CDCl_3$) of **6b**.

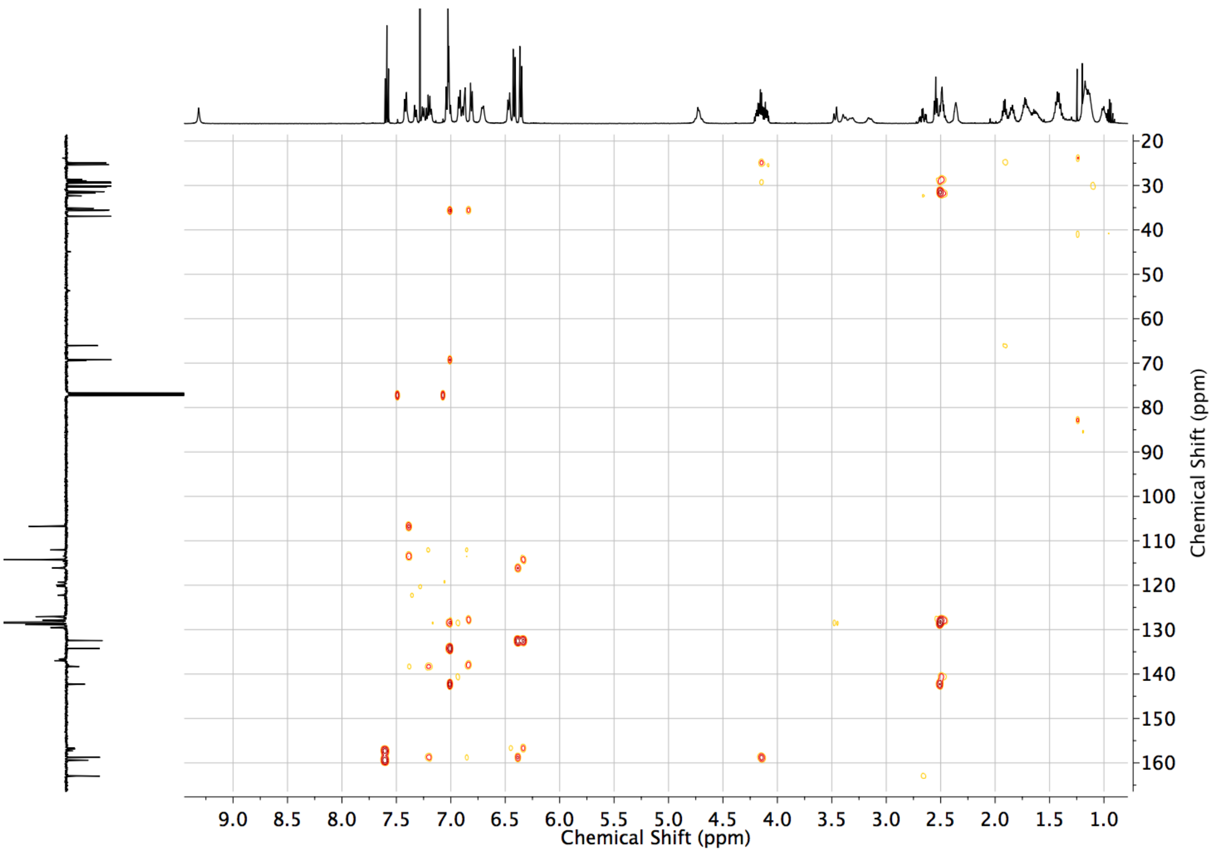

Figure S85 HMBC NMR ($CDCl_3$) of **6b**.

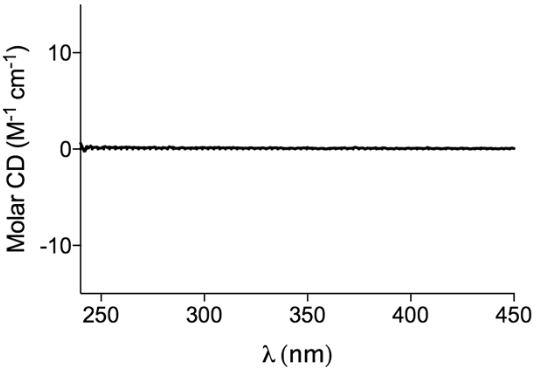

Figure S86 Circular dichroism spectrum of *rac*-**6b** (43.0 µM in $CHCl_3$, 293 K).

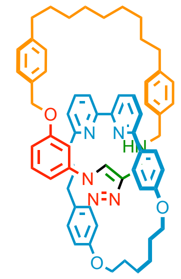

(S_{mt})-**6b**

To a solution of oxalyl chloride (140 µl, 1.60 mmol, 1.2 eq.) in dry CH_2Cl_2 (3 mL) at -78 °C was added DMSO (230 µL, 3.25 mmol, 2.4 eq.). The mixture was stirred for 10 minutes at -78 °C before 0.67 mL of the activated DMSO solution was added to (*R*,S_{mt})-**3b** (150 mg, 0.135 mmol, 1 eq.) in dry CH_2Cl_2 (5 mL) at -78 °C. NEt_3 (91 µL, 0.65 mmol, 4.8 eq.) was added at -78 °C. The mixture was warmed to r.t. and stirred at r.t. for 1 h. The reaction mixture was diluted with CH_2Cl_2 (50 mL) and washed with a saturated solution of $NaHCO_3$ $_{(aq)}$ (20 mL), dried ($MgSO_4$) and the solvent removed *in vacuo*. The residue was dissolved in $CHCl_3$ (5 mL), AcOH (1 mL) was added and the mixture was stirred for 3 h at r.t. The solvent was removed *in vacuo*. The residue was dissolved in CH_2Cl_2 (100 mL) and washed with $NaHCO_3$ (20 mL), dried ($MgSO_4$) and the solvent removed *in vacuo*. After purification by column chromatography on silica (CH_2Cl_2 with a gradient of 0 to 50% EtOAc) (S_{mt})-**6b** was obtained as a white foam (89.0 mg, 68% over 2 steps). HPLC: RegisCell (hexane/*i*-PrOH, 98:2), flow rate 0.5 mL.min^{-1}, t_{major} = 26.5, ee = 98.5%). Spectroscopic data were identical to those reported for *rac*-**6b** with the exception of the circular dichroism spectra.

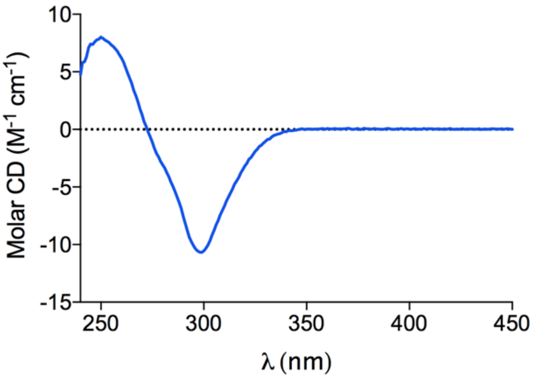

Figure S87 Circular dichroism spectrum of (S_{mt})-**6b** (43.0 µM in $CHCl_3$, 293 K).

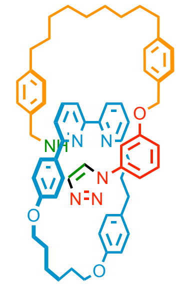

(R_{mt})-**6b**

An identical procedure to that for (S_{mt})-**6b** but employing (S,R_{mt})-**3b** (290 mg, 0.27 mmol), oxalyl chloride (50 µL, 0.60 mmol, 2.2 eq.), DMSO (85 µL, 1.20 mmol, 4.4 eq.) and NEt_3 (167 µL, 1.20 mmol, 4.4 eq.) afforded (R_{mt})-**6b** as a white foam (120 mg, 50% over 2 steps). HPLC: RegisCell (hexane/*i*-PrOH, 98:2), flow rate 0.5 $mL.min^{-1}$, t_{major} = 20.0, ee > 99%). Spectroscopic data were identical to those reported for *rac*-**6b** with the exception of the circular dichroism spectra.

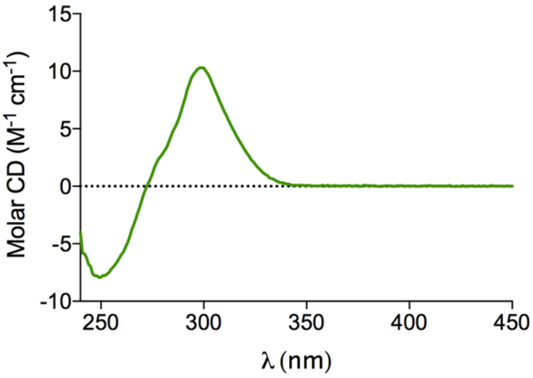

Figure S88 Circular dichroism spectrum of (R_{mt})-**6b** (43.0 µM in $CHCl_3$, 293 K).

5. Syntheses of triazole-functionalised macrocycles S12 and S13

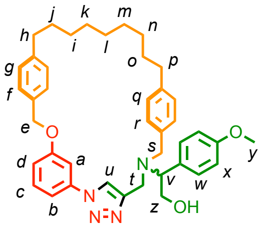

S12

To a solution of [Cu(CH_3CN)$_4$]PF_6 (23.1 mg, 0.062 mmol, 0.98 eq.), $^{i}Pr_2NEt$ (27 μL, 0.15 mmol, 2.4 eq.) in 1:1 $CHCl_3$/EtOH (6.2 mL) at 60 °C was added **1** (40.0 mg, 0.062 mmol, 1.0 eq.) in 1:1 $CHCl_3$/EtOH (2.4 mL) over 4 h. After removal of the solvent *in vacuo*, the residue was dissolved in CH_2Cl_2 (20 mL) and washed with EDTA-NH_3 (2 × 10 mL), dried ($MgSO_4$) and the solvent removed *in vacuo*. After purification by column chromatography on silica (Petrol/CH_2Cl_2 1/1 with a gradient from 0 to 20% EtOAc) **S12** was obtained as a white foam (30.0 mg, 75%). ^{1}H NMR (500 MHz, $CDCl_3$) **δ**: 7.69 (s, 1H, H_u), 7.54-7.41 (m, 2H, H_c, H_b or H_d), 7.32 (d, *J* = 8.2, 2H, H_f), 7.27 (d, *J* = 8.8, 2H, H_w), 7.21 (d, *J* = 8.0, 2H, H_r), 7.17 (d, *J* = 8.4, 2H, H_g), 7.12 (d, *J* = 8.2, 2H, H_q), 7.08 (dt, *J* = 2.4, 1.0, 1H, H_a), 7.07-7.04 (m, 1H, H_b or H_d), 6.95 (d, *J* = 8.8, 2H, H_x), 5.12 (s, 2H, H_e), 4.12 (t, *J* = 10.6, 1H, 1 of H_z), 4.04 (dd, *J* = 10.4, 4.8, 1H, H_v), 3.95 (d, *J* = 15.2, 1H, 1 of H_t), 3.88 (d, *J* = 13.4, 1H, 1 of H_s), 3.84 (s, 3H, H_y), 3.68 (dd, *J* = 10.8, 4.7, 1H, 1 of H_z), 3.61 (d, *J* = 14.6, 1H, 1 of H_t), 3.18 (d, *J* = 13.6, 1H, 1 of H_s), 2.62 (td, *J* = 6.7, 2.4, 2H, H_h or H_p), 2.54 (t, *J* = 7.7, 2H, , H_h or H_p), 1.64-1.50 (m, 4H, H_i, H_o), 1.22 (m, 10H, H_j, H_k, H_l, H_m, H_n). ^{13}C NMR (126 MHz, $CDCl_3$) **δ** 159.5 (×2), 147.5, 143.0, 142.1, 136.2, 133.5, 130.8, 130.5, 129.2, 128.8, 128.7, 128.1, 127.2, 120.4, 114.2, 113.9, 113.2, 108.3, 70.4, 63.6, 61.0, 55.4, 53.9, 44.7, 35.6, 35.5, 31.2, 30.9, 29.6, 29.2, 29.0, 28.9, 28.3. HR-ESI-MS *m/z* = 645.3813 $[M+H]^+$ (calc. for $C_{41}H_{49}N_4O_3$ 645.3799).

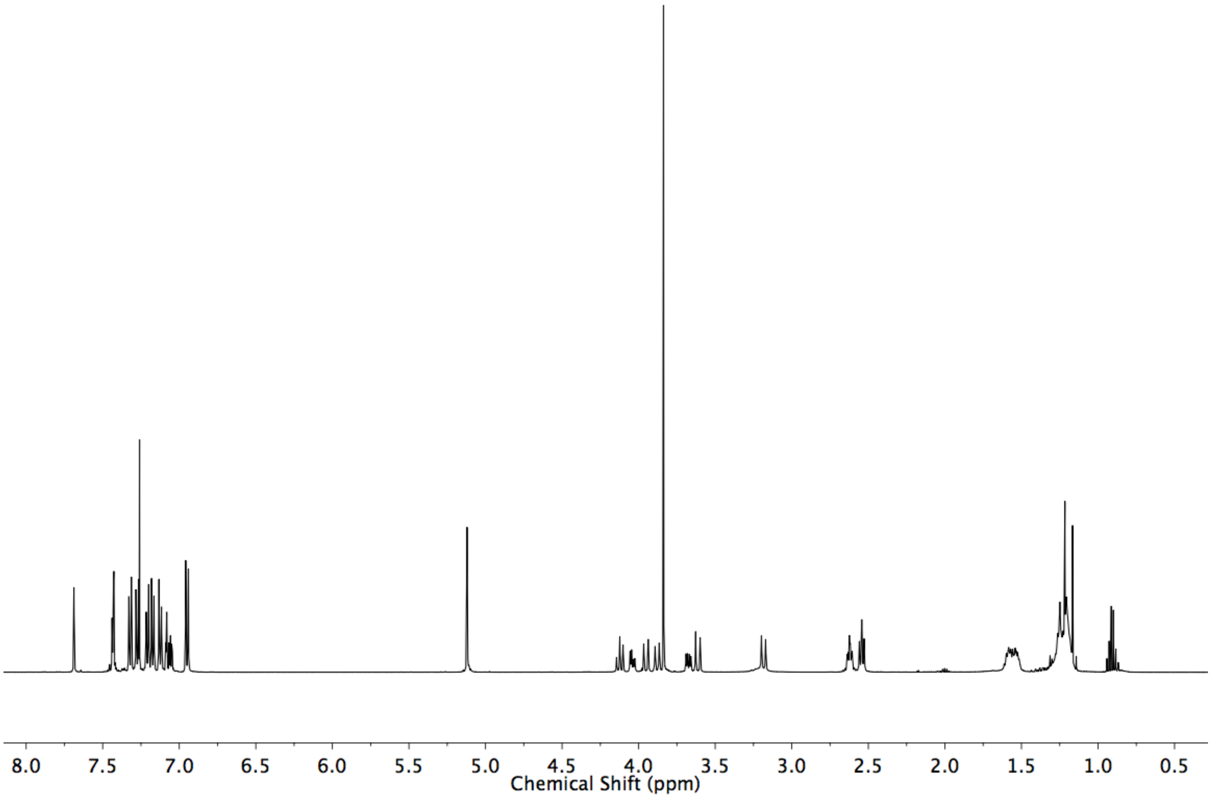

Figure S89 ^{1}H NMR (500 MHz, $CDCl_3$) of **S12**.

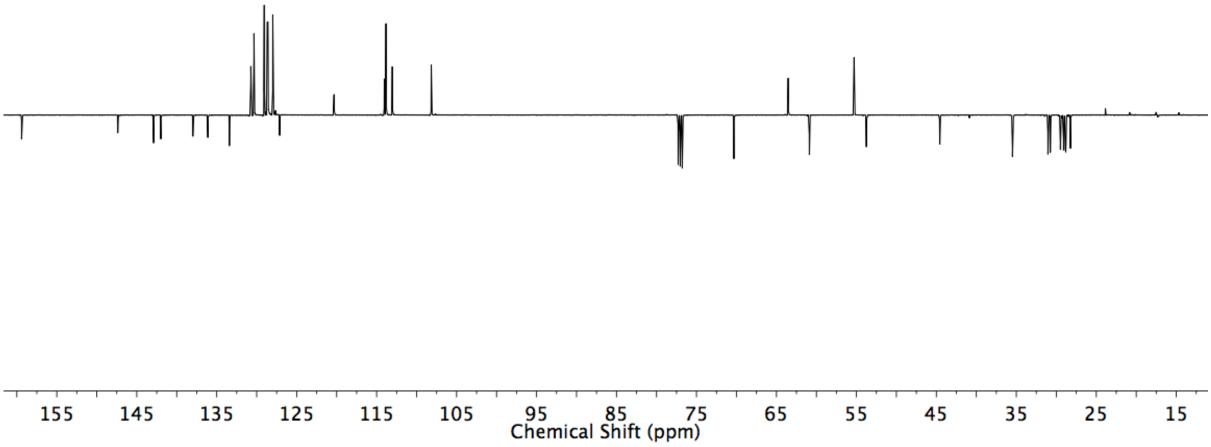

Figure S90 JMOD NMR (126 MHz, $CDCl_3$) of **S12**.

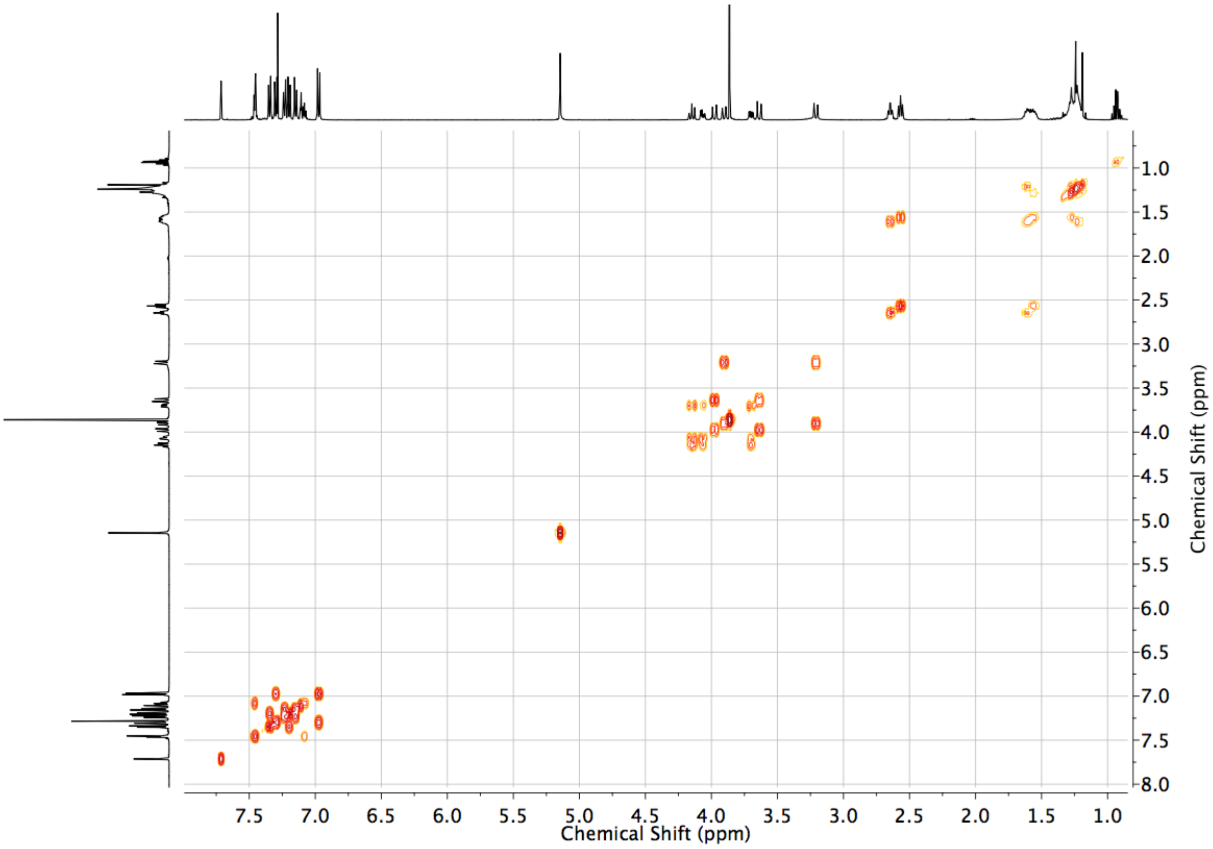

Figure S91 COSY NMR ($CDCl_3$) of **S12**.

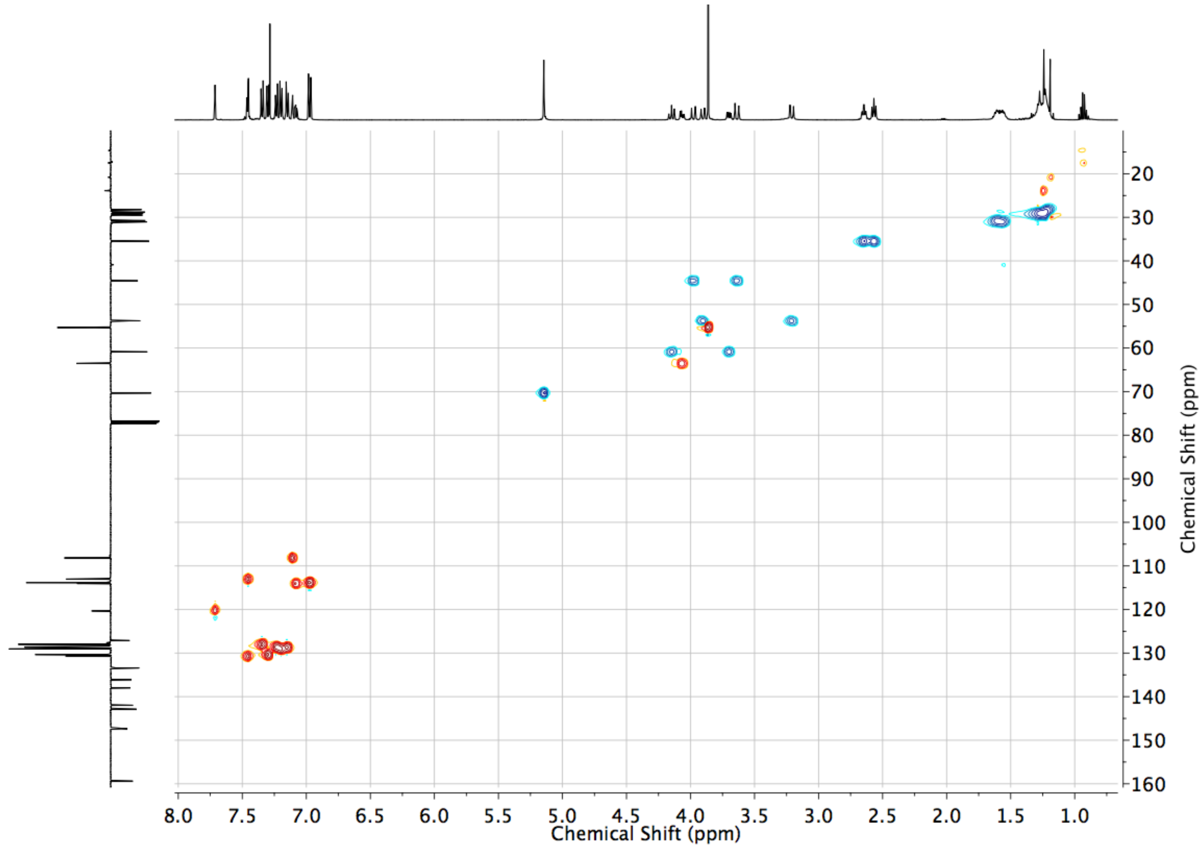

Figure S92 HSQC NMR ($CDCl_3$) of **S12**.

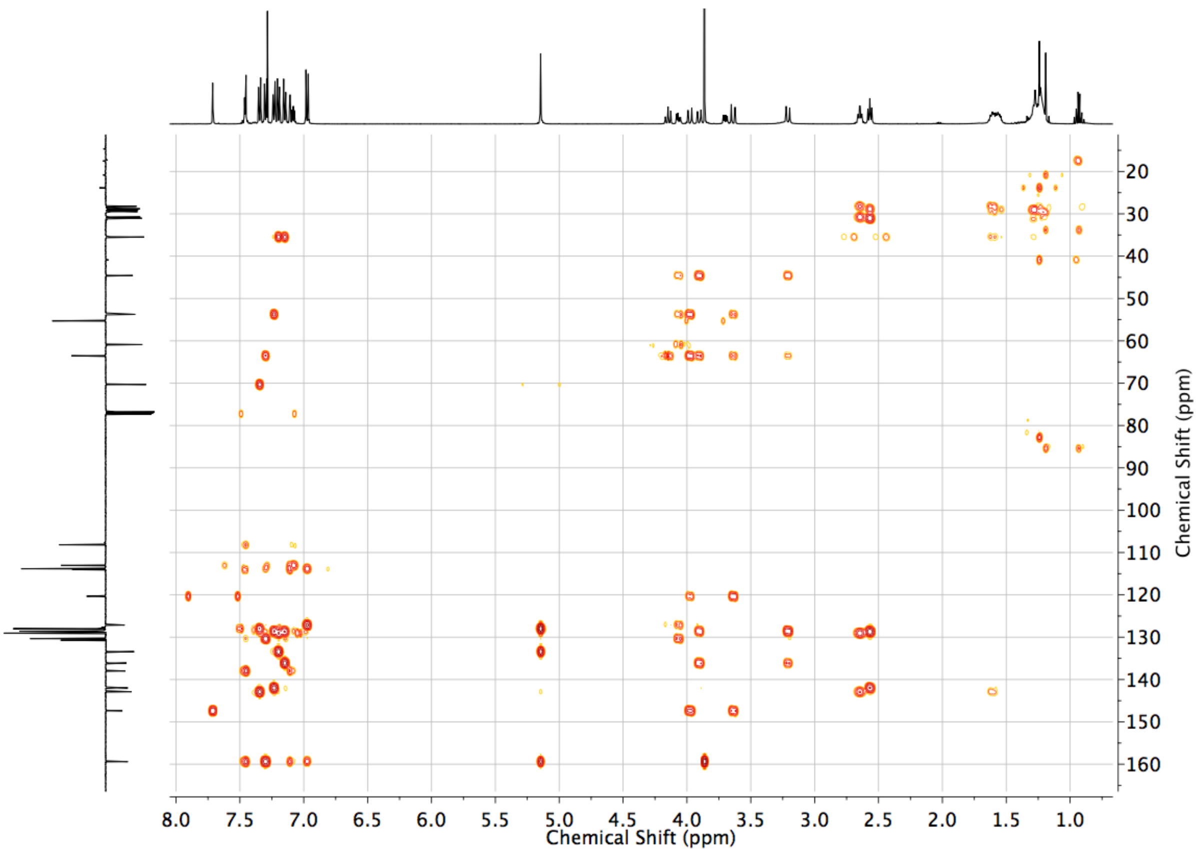

Figure S93 HMBC NMR ($CDCl_3$) of **S12**.

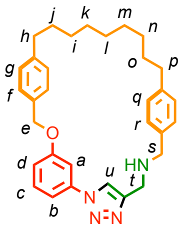

S13

To a solution of $[Cu(CH_3CN)_4]PF_6$ (27.9 mg, 0.075 mmol, 0.98 eq.), $^iPr_2NEt$ (27 μL, 0.15 mmol, 2.0 eq.) in 1:1 $CHCl_3$/EtOH (7.5 mL) at 60 °C was added **S11** (38.0 mg, 0.077 mmol, 1.0 eq.) in 1:1 $CHCl_3$/EtOH (3.0 mL) over 4 h. After removal of the solvent *in vacuo*, the residue was dissolved in CH_2Cl_2 (20 mL) and washed with EDTA-NH_3 (2 × 10 mL), dried ($MgSO_4$) and the solvent removed *in vacuo*. After purification by column chromatography on silica (Petrol/CH_2Cl_2 1/1 with a gradient from 0 to 50% EtOAc) **S13** was obtained as a white foam (12.0 mg, 32%). ^{1}H NMR (500 MHz, $CDCl_3$) **δ**: 7.73 (s, 1H, H_u), 7.44-7.41 (m, 2H, H_c, H_b or H_d), 7.27 (d, J = 8.4, 2H, H_f), 7.22 (d, J = 8.4, 2H, H_r), 7.14 (d, J = 8.4, 2H, H_g), 7.10 (d, J = 8.4, 2H, H_q), 7.08-7.04 (m, 1H, H_b or H_d), 7.00-6.97 (m, 1H, H_a), 5.13 (s, 2H, H_e), 4.04 (s, 2H, H_t), 3.82 (s, 2H, H_s), 2.57 (t, J = 7.3, 4H, H_h, H_p), 1.62-1.50 (m, 4H, H_i, H_o), 1.40-1.05 (m, 10H, H_j, H_k, H_l, H_m, H_n). ^{13}C NMR (126 MHz, $CDCl_3$) **δ** 159.7, 148.1, 143.0, 141.7, 138.2, 137.2, 133.7, 130.8, 129.1, 128.8, 128.3, 127.6, 120.3, 115.1, 113.2, 107.8, 70.6, 53.4, 44.6, 35.7, 35.4, 31.2 (×2), 29.6 (×2), 29.2, 28.9, 28.4. HR-ESI-MS m/z 495.3123 $[M+H]^+$ (calc. for $C_{32}H_{39}N_4O$ 495.3118).

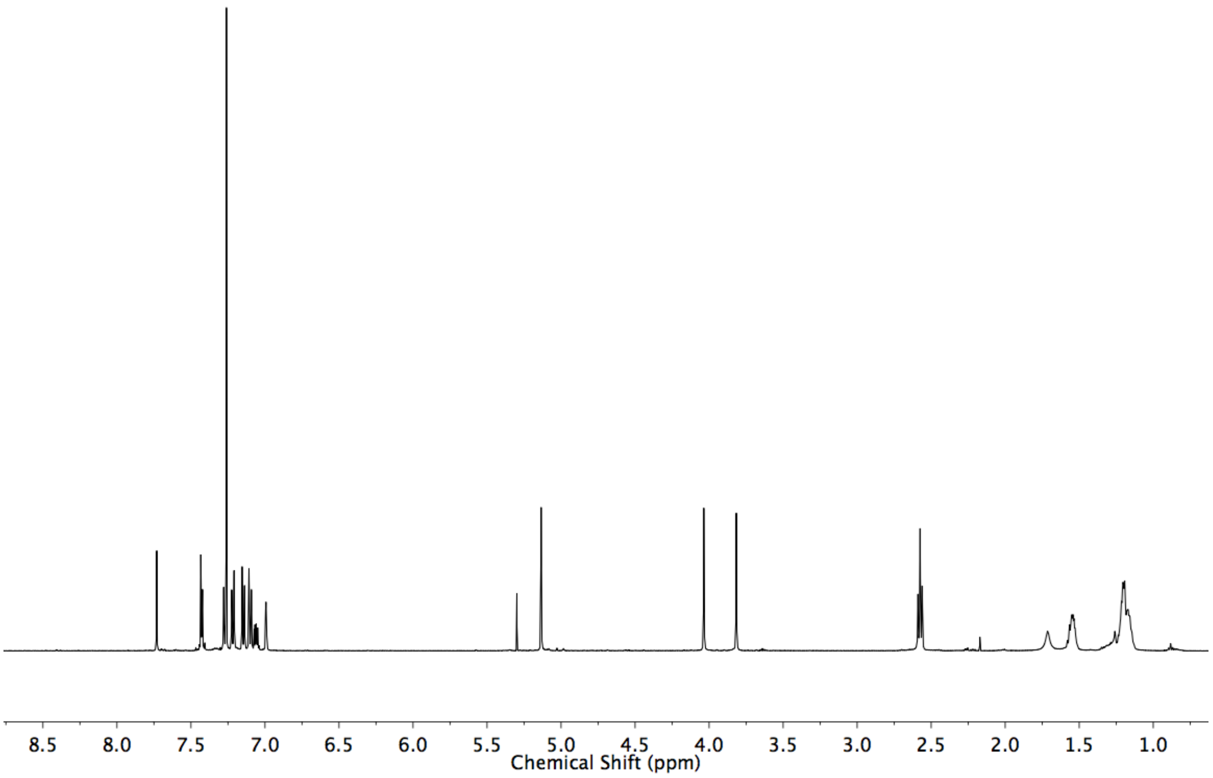

Figure S94 ^{1}H NMR (500 MHz, $CDCl_3$) of **S13**.

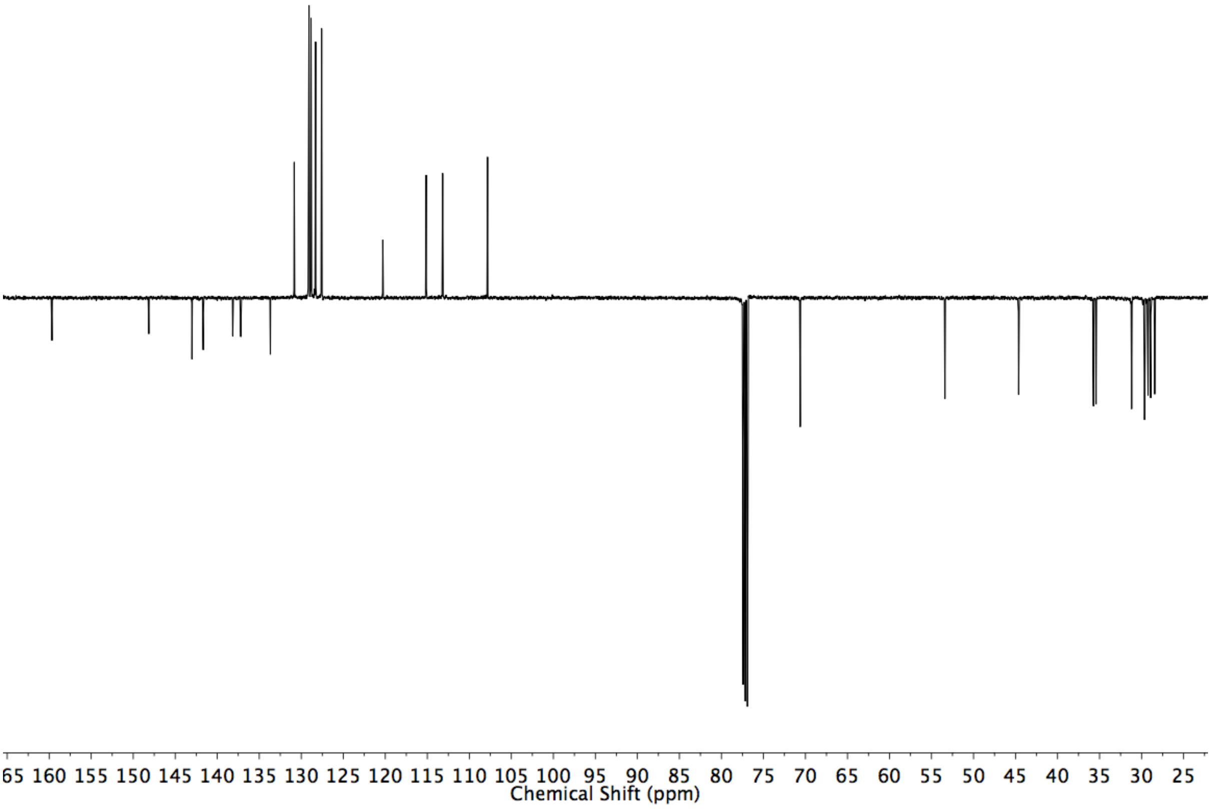

Figure S95 JMOD NMR (126 MHz, $CDCl_3$) of **S13**.

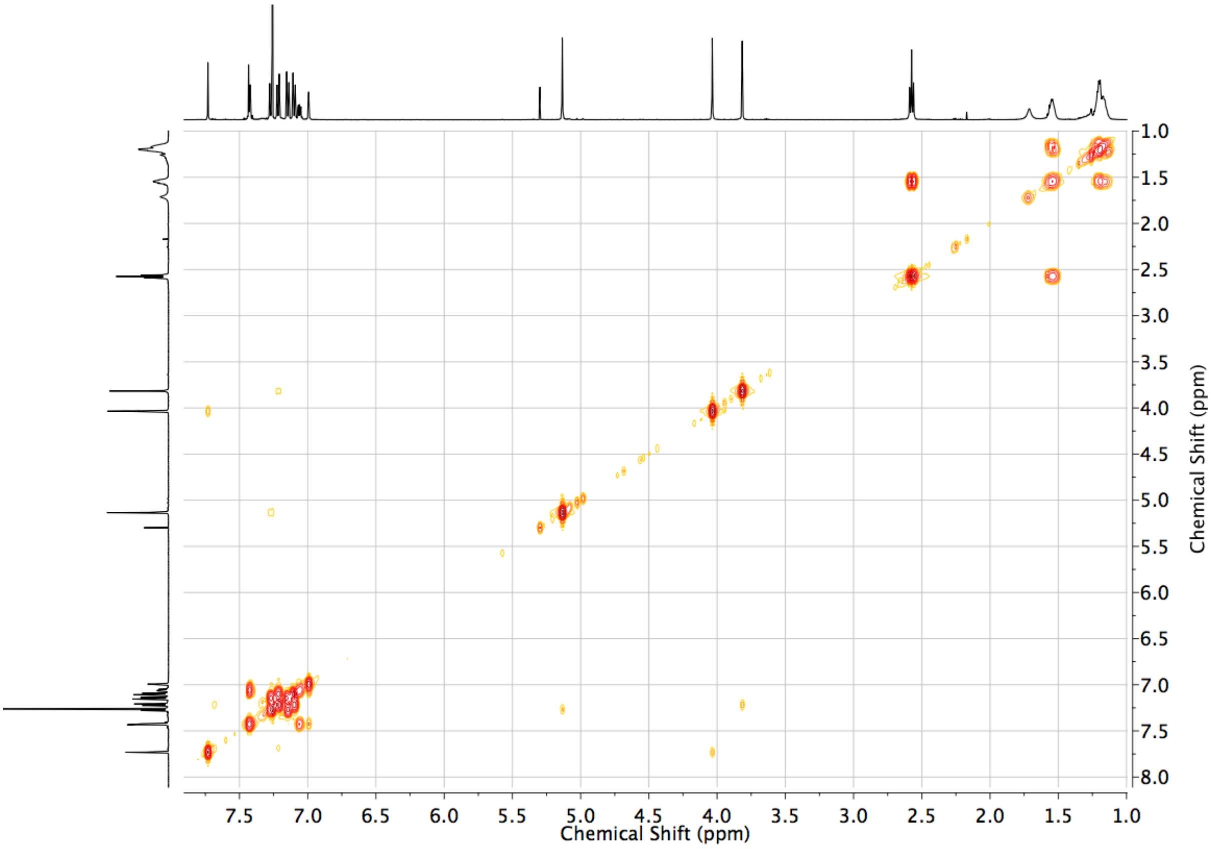

Figure S96 JMOD NMR ($CDCl_3$) of **S13**.

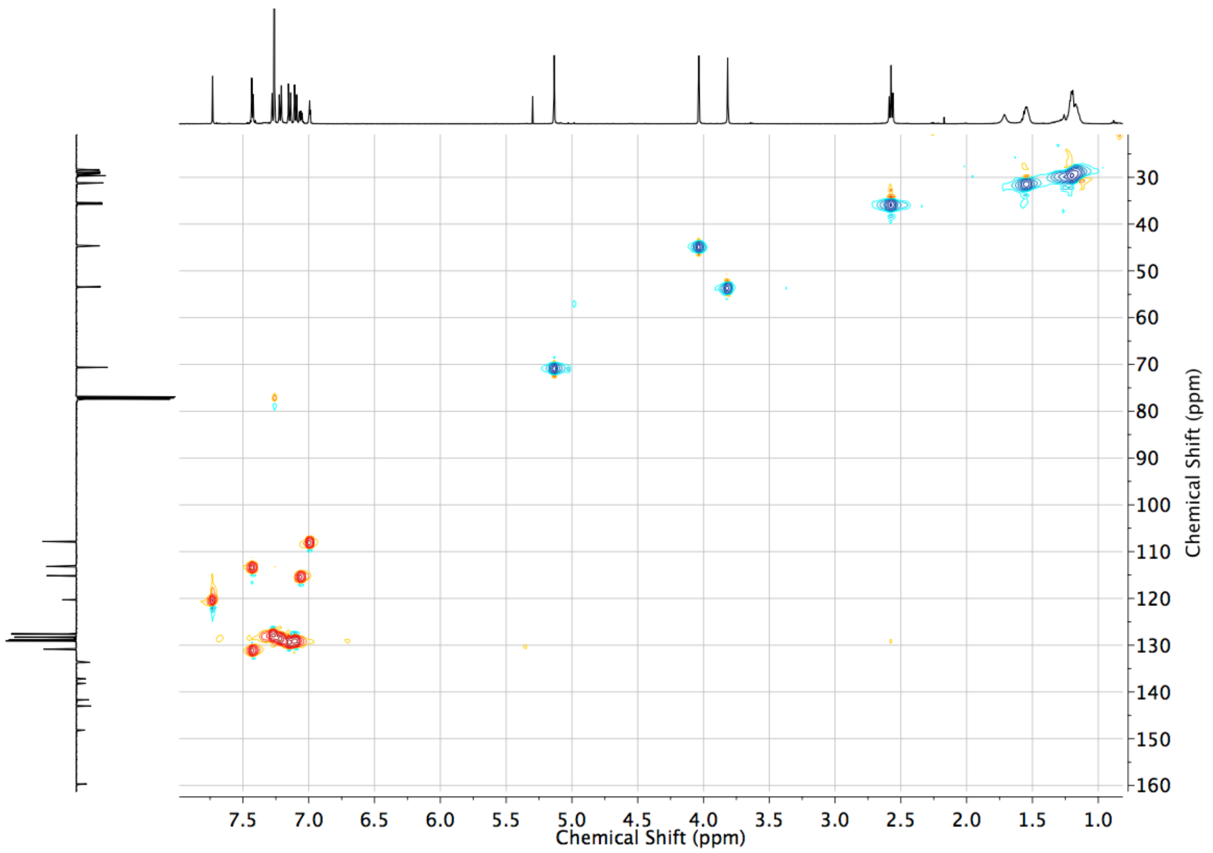

Figure S97 HSQC NMR ($CDCl_3$) of **S13**.

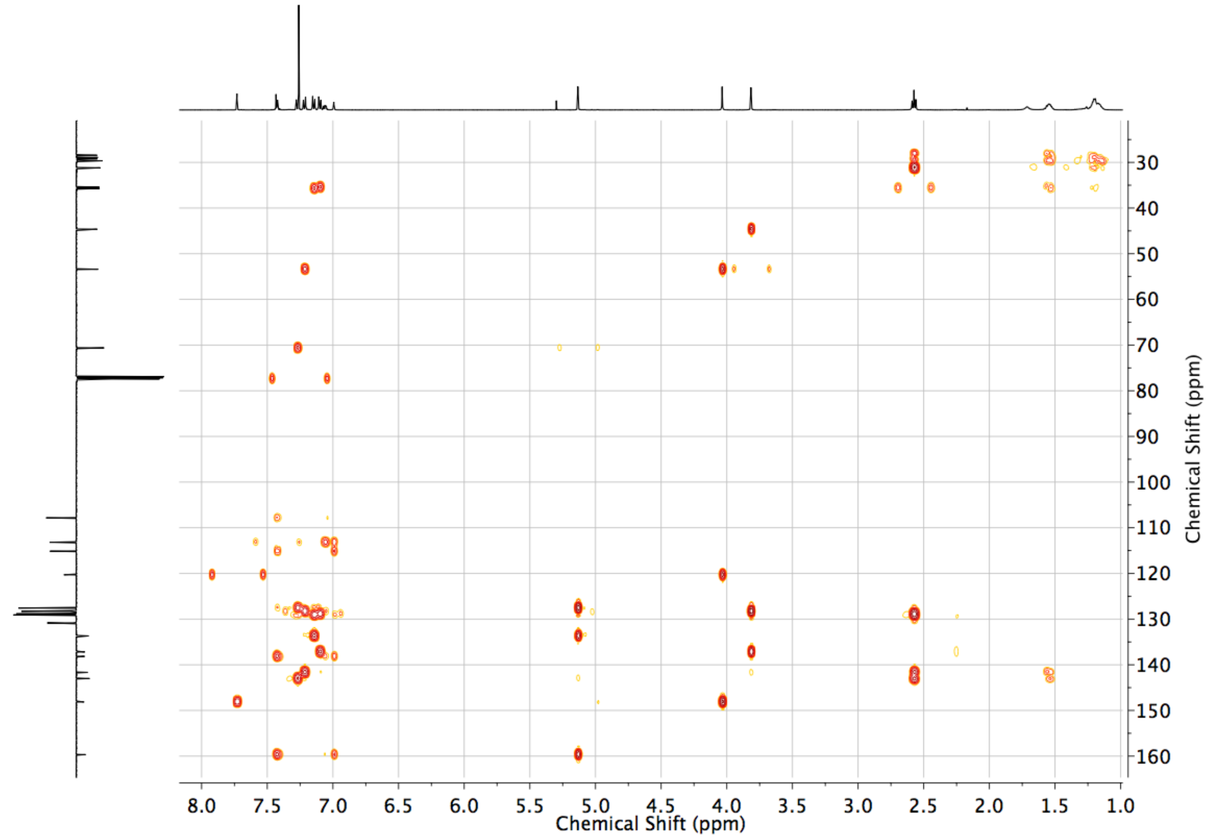

Figure S98 HMBC NMR ($CDCl_3$) of **S13**.

6. Chiral Stationary Phase HPLC analysis of catenane 6b, S8 and S9

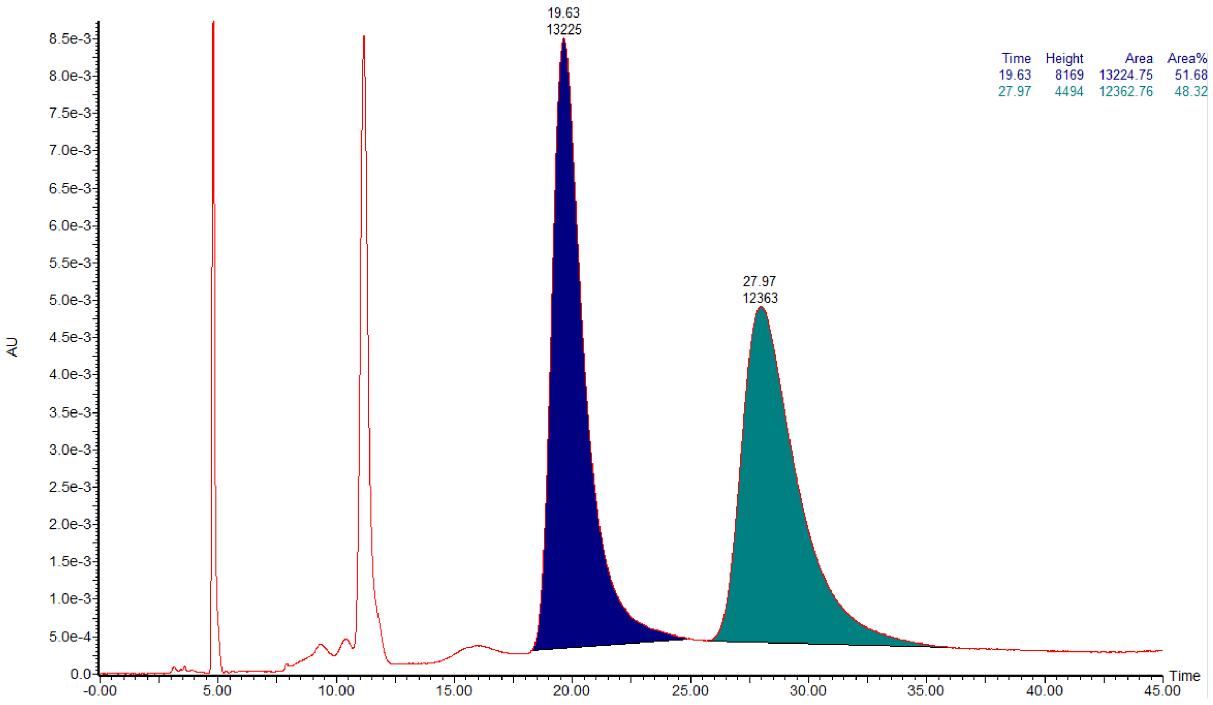

Figure S99 CSP-HPLC for the Racemic catenane **6b.** RegisCell (hexane/*i*-PrOH, 98:2), flow rate 0.5 mL.min^{-1}, diode array detection.

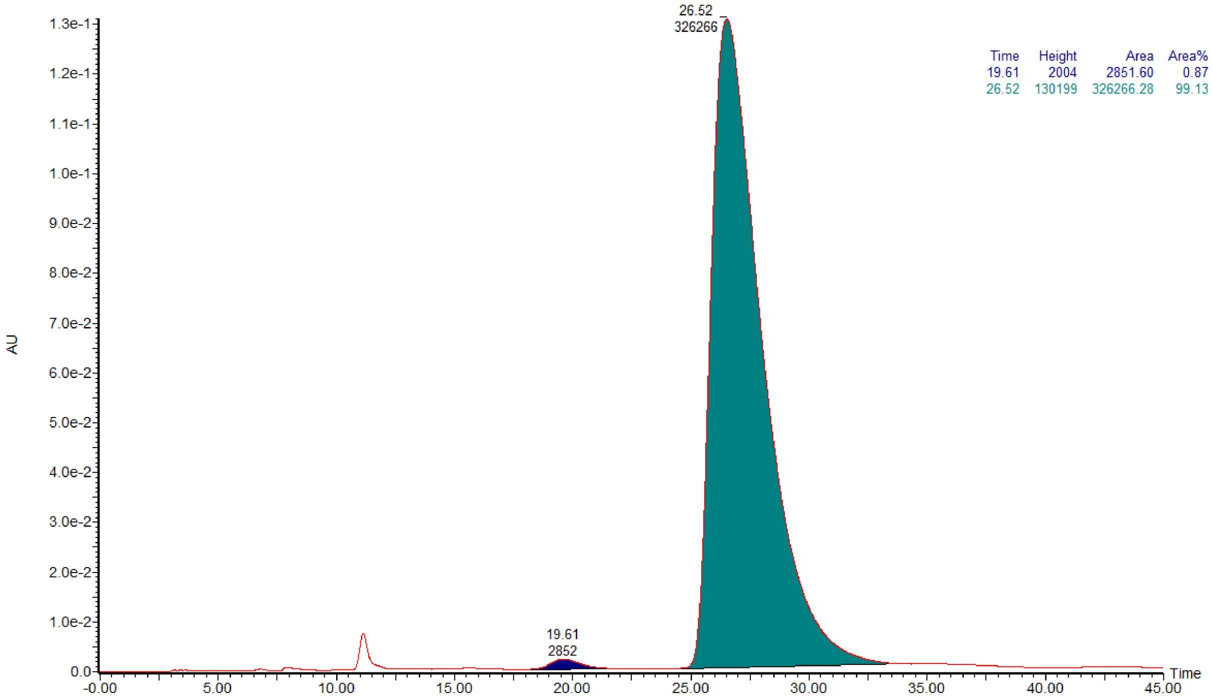

Figure S100 CSP-HPLC for the (R_{mt})-**6b.** RegisCell (hexane/*i*-PrOH, 98:2), flow rate 0.5 mL.min^{-1}, diode array detection.

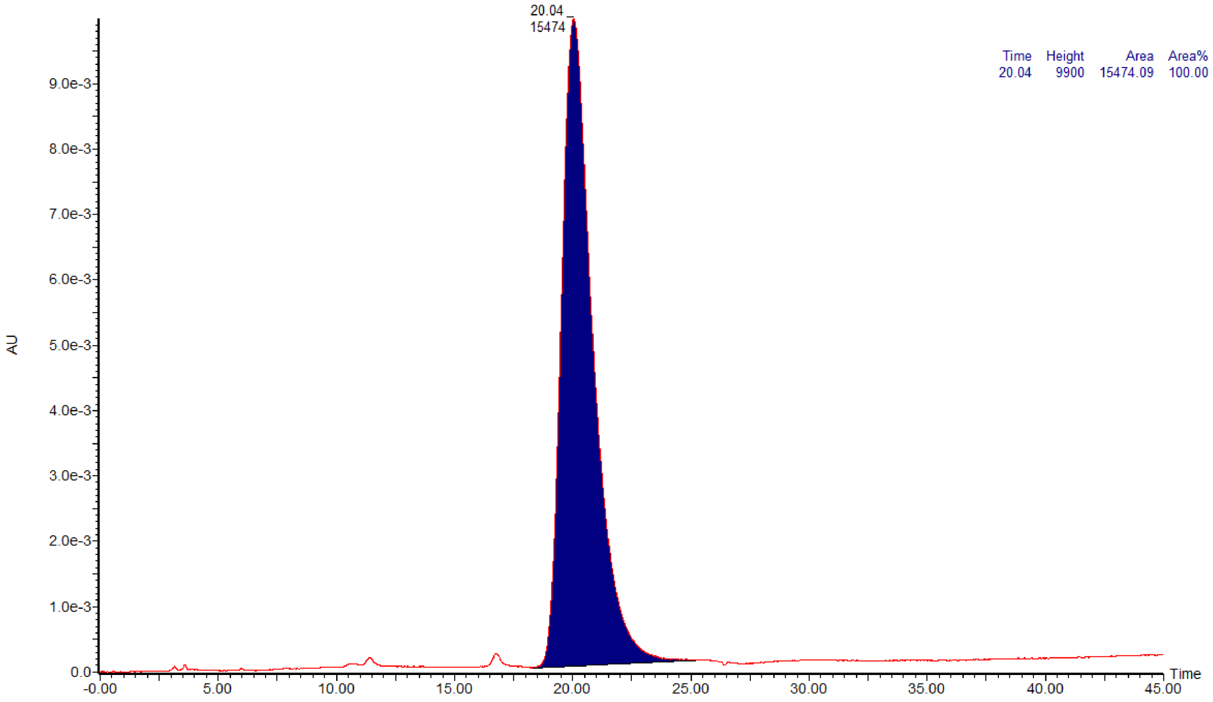

Figure S101 CSP-HPLC for the (S_{mt})-**6b.** RegisCell (hexane/*i*-PrOH, 98:2), flow rate 0.5 mL.min^{-1}, diode array detection.

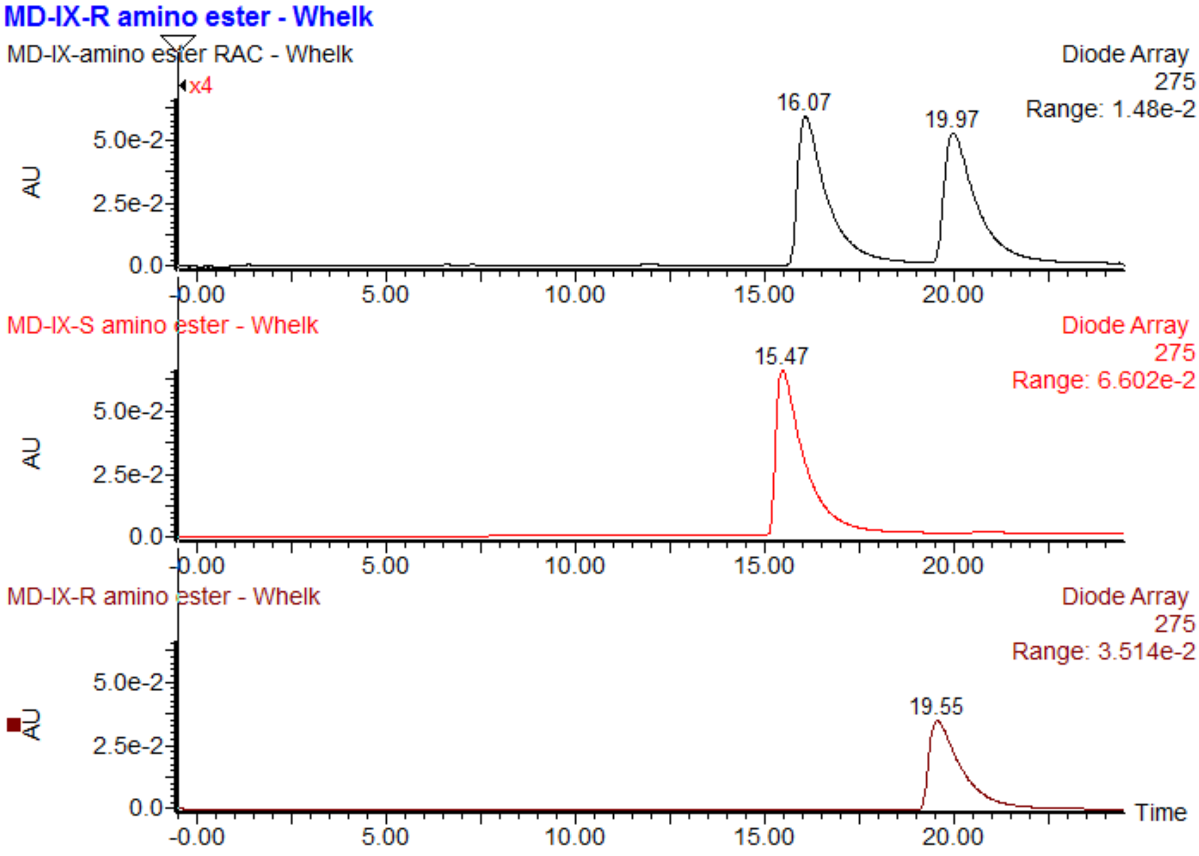

Figure S102 CSP-HPLC of (top) rac-**S8**, (middle) (*S*)-**S8**, and (bottom) (*R*)-**S8.** Whelk-O 1 (hexane/*i*-PrOH, 90:10), flow rate 2.0 mL.min^{-1}, λ = 275 nm.

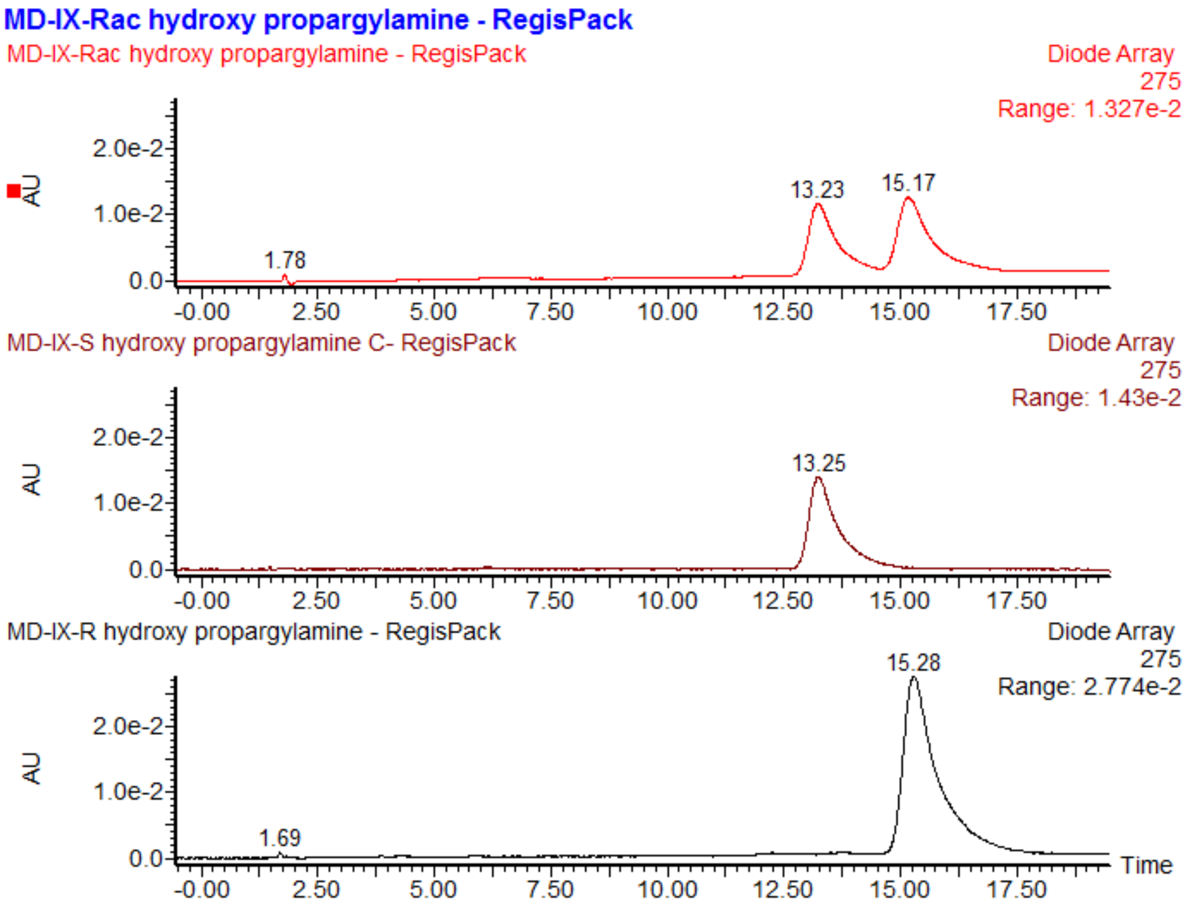

Figure S103 CSP-HPLC of (top) rac-**S9**, (middle) (*S*)-**S9**, and (bottom) (*R*)-**S9**. RegisPack (hexane/EtOH, 97:3), flow rate 1.5 mL.min^{-1}, λ = 275 nm.

7. Single Crystal X-ray Analysis of Catenane (S^*,R^*_{mt})-3b

Experimental: Single colourless crystals of catenane (S^*,R^*_{mt})-**3b** were obtained from vapor diffusion of pentane into a chloroform solution of the product. A suitable crystal was selected (0.10×0.09×0.08 mm^3) and data were collected at a steady T = 100(2) K using a FRE+ HF diffractometer equipped with a Saturn 724+ enhanced sensitivity detector. Cell determination, data collection, data reduction, cell refinement and absorption correction were performed with CrysalisPro. During the data processing, the lower resolution limit was set to 0.90 Å because of the low resolution of the material. The structure was solved with the SHelXT[6] structure solution program using the Intrinsic Phasing solution method and by using Olex2[7] (Dolomanov et al., 2009) as the graphical interface. The model was refined against F_2 using anisotropic thermal displacement parameters for all non-hydrogen atoms using with version 2018/3 of ShelXL[6] using Least Squares minimisation. Hydrogen atoms were placed in calculated positions and refined using a riding model. ShelX SADI, SIMU, ISOR, and RIGU restraints were used in the refinement strategy, as listed in the cif file, mostly for one of the aromatic rings and the alkyl chain connected to it in one of the two catenanes present within the unit cell.

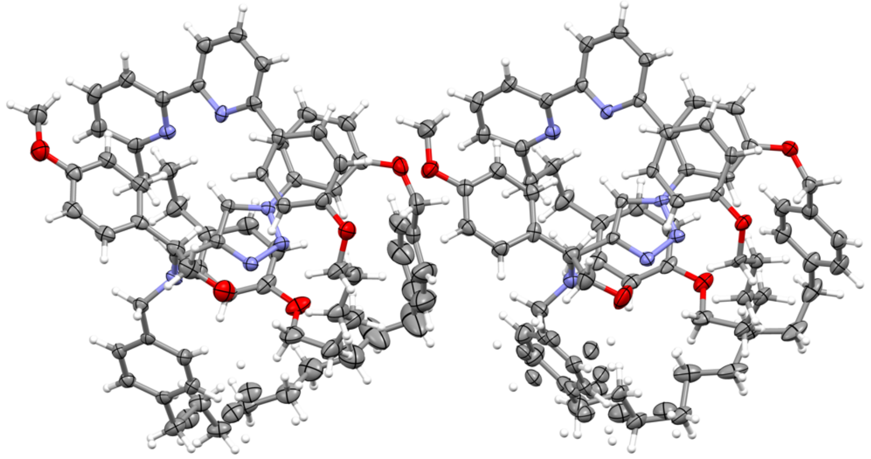

Figure S104 Ellipsoid plot of the asymmetric unit of a racemic mixture (R,S_{mt}) and (S,R_{mt}) of catenane **3b**. Ellipsoids are shown at the 50% probability level.

Compound	**C6MajorDia**	
CCDC No.	1885204	
Empirical formula	$C_{144}H_{158}N_{12}O_{10}$	
Formula weight	2216.81	
Temperature	100(2) K	
Wavelength	0.71073 Å	
Crystal system	Monoclinic	
Space group	$P2_1/n$	
Unit cell dimensions	a = 26.2879(9) Å	[illegible]= 90°
	b = 16.1275(5) Å	[illegible]= 106.741(4)°
	c = 29.7838(11) Å	[illegible] = 90°
Volume	12091.9(8) Å^3	
Z	4	
Density (calculated)	1.218 Mg/m^3	
Absorption coefficient	0.077 mm^{-1}	
F(000)	4744	
Crystal size	0.095 × 0.090 × 0.080 mm^3	
Theta range for data collection	1.500 to 23.256°.	

Index ranges	-29<=h<=29, -17<=k<=17, -33<=l<=33
Reflections collected	162942
Independent reflections	17362 [R(int) = 0.1998]
Completeness to theta = 23.256°	100.0 %
Absorption correction	Semi-empirical from equivalents
Max. and min. transmission	1.00000 and 0.72382
Refinement method	Full-matrix least-squares on F^2
Data / restraints / parameters	17362 / 0 / 1499
Goodness-of-fit on F^2	1.038
Final R indices [I>2sigma(I)]	R_1 = 0.0885, wR_2 = 0.2087
R indices (all data)	R_1 = 0.1668, wR_2 = 0.2649
Extinction coefficient	n/a
Largest diff. peak and hole	0.673 and -0.575 e.Å^{-3}

The solid state structure of the racemic (R,S_{mt})-**3b**/(S,R_{mt})-**3b** mixture contains two crystallographically independent conformers of the catenane in the asymmetric unit. These pack together to form one-dimensional chains composed of single enantiomers extending along the *a* axis (**Figure S105**). Adjacent to each of the chains pack, on one side, anti-parallel chains of the same enantiomer, and, on the other side, chains of the opposite enantiomer. This results in a two-dimensional lattice with a repeating pattern of (R,S_{mt}) (R,S_{mt}) (S,R_{mt}) (S,R_{mt}) one-dimensional chains (**Figure S106**), with adjacent chains of opposite enantiomers packing in an anti-parallel fashion. This packing of chains composed of opposite enantiomers adjacent to each other could help explain the relative ease with which the racemic mixture was able to be crystallised relative to the enantiopure compound.

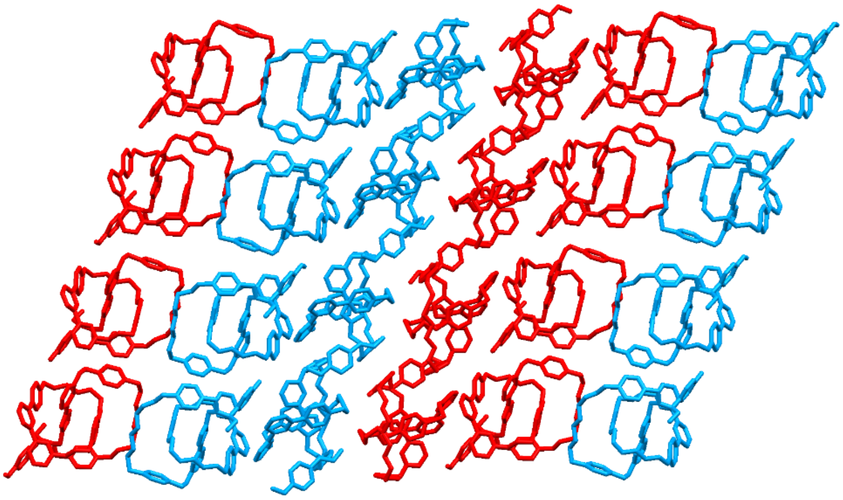

Figure S105 Packing diagram of the solid state structure of (R,S_{mt})-**3b**/(S,R_{mt})-**3b** showing the one-dimensional chains that run parallel to the *a* axis. (R,S_{mt})-**3b** is shown in red and (S,R_{mt})-**3b** in blue. Hydrogen atoms have been omitted flor clarity

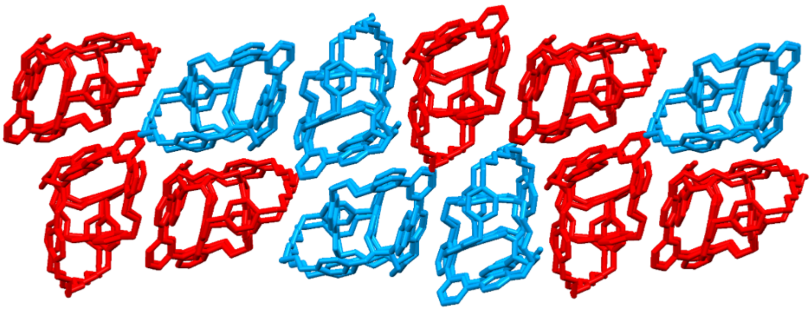

Figure S106 Packing diagram of the solid state structure of (R,S_{mt})-**3b**/(S,R_{mt})-**3b** viewed down the *a* axis. (R,S_{mt})-**3b** is shown in red and (S,R_{mt})-**3b** in blue. Hydrogen atoms have been omitted flor clarity.

8. Preliminary Molecular Modelling of the AT-CuAAC Reaction Between (*R*)-1 and 2b

Based on previous work,[4] the AT-CuAAC reaction of macrocycle **2b** with (*R*)-**1** is thought to proceed *via* Cu^I-acetylides **Ib** which are irreversibly converted to Cu^I-triazolides **IIb** and then, after protolytic work-up, to catenanes (R,S_{mt})-**3b** (major) and (R,R_{mt})-**3b** (**Scheme S4**). Based on this proposed mechanism, two obvious sources of diastereoselectivity can be identified; i) an energy difference between (*R*,*S*)-**Ib** and (*R*,*R*)-**Ib** which results in a biased pre-equilibrium (Cu^I acetylide formation can be expected to be reversible in the presence of N^iPr_2Et) prior to irreversible covalent bond formation; ii) a difference in reaction rate for the conversion of (*R*,*S*)-**Ib** → (R,S_{mt})-**IIb** and (*R*,*R*)-**Ib** → (R,R_{mt})-**IIb**.

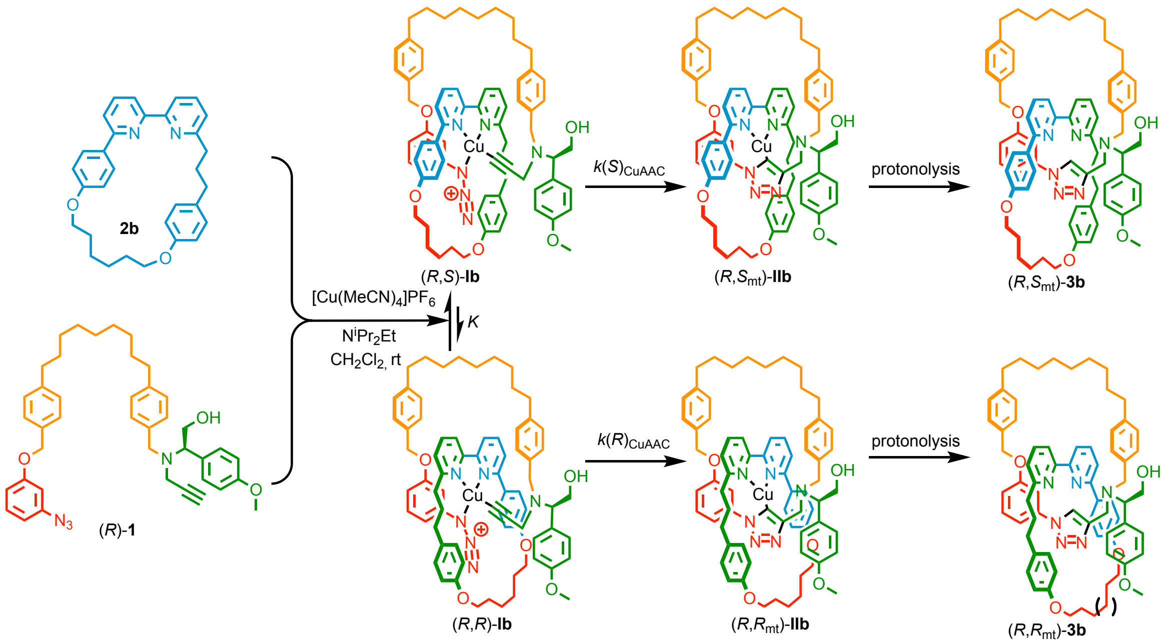

Scheme S4 Schematic mechanism of the AT-CuAAC reaction *via* acetylides (**Ib**) and triazolides (**IIb**)

To probe the origin of stereoselectivity in this AT-CuAAC reaction we carried out preliminary calculations (DFT, rB3LYP, 631G) to determine the relative energies of diastereomers **I** and the Δ_rG of the diastereomeric reactions **Ib→IIb**. It should be noted that, given the controversial nature of the cycloaddition mechanism and the size of the molecules concerned, accurately identifying the transition state energies for the cycloaddition step lies beyond the scope of this preliminary study. However, linear-free energy relationship considerations suggest that reactions proceeding *via* the same pathway but with a more negative Δ_rG of reaction can be expected to have a lower reaction barrier, although the difference in reaction energies is likely to be significantly larger than the difference in activation energies as this exoergic reaction is predicted to have an early barrier (Hammond postulate). Thus, although these calculations cannot be used to derive the difference in reaction rates for (*R*,*S*)-**Ib** → (R,S_{mt})-**IIb** and (*R*,*R*)-**Ib** → (R,R_{mt})-**IIb**, the comparison of the reaction energies gives an indication of whether a difference in reaction rates is to be expected.

Molecular models of intermediates **Ib** and **IIb** were prepared in Spartan '10 (Wavefunction Ltd) and subjected to a conformer search (MMFF).[5] The lowest energy conformer of each was selected and the coordinates obtained were transferred to GausView5 (Gaussian, Inc., Wallingford CT, 2009). Each lowest energy conformer was subjected to optimization first using semi-empirical (PM6) then DFT (rB3LYP, 631G, gas phase) using Gaussian '09 (Gaussian, Inc., Wallingford CT, 2009).[6] The energies obtained (normalized to the lowest energy of the series) are given in Table S1.

Table S1. Computed energies (normalised to 0 kJmol^{-1} for (*R*,*S*$_{mt}$)-**IIb**) of molecular models of the AT-CuAAC reaction intermediates of pre-macrocycle (*R*)-**1** and macrocycle **2b**.

Entry	Acetylide intermediate		Triazolide intermediate
1	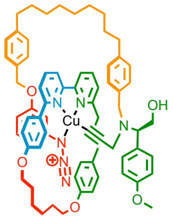 (*R*,*S*)-**Ib**<br>243.8 kJmol^{-1}	ΔG = –243.8 kJmol^{-1}<br>→	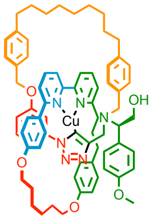 (*R*,*S*$_{mt}$)-**IIb**<br>0 kJmol^{-1}
2	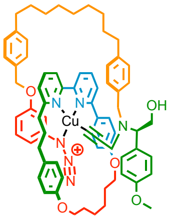 (*R*,*R*)-**Ib**<br>248.5 kJmol^{-1}	ΔG = –246.7 kJmol^{-1}<br>→	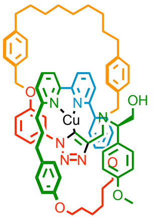 (*R*,*R*$_{mt}$)-**IIb**<br>1.8 kJmol^{-1}
ΔE =	4.7 kJmol^{-1}	–2.9 kJmol^{-1}	1.8 kJmol^{-1}

Based on the results in Table S1 the origin of the stereoselectivity is predicted to be complex as the biased pre-equilibrium, which favours (*R*,*S*)-**Ib** by approximately 5.3:1 at 337 K, is opposed by the predicted kinetic preference of the cycloaddition step, which the data suggests favours the formation of the (*R*,*R*$_{mt}$) product (Δ_rG for (*R*,*R*)-**Ib** → (*R*,*R*$_{mt}$)-**IIb** is ~2.9 kJmol^{-1} more exoergic than (*R*,*S*)-**Ib** → (*R*,*S*$_{mt}$)-**IIb**). It should be noted, however, that the predicted level of selectivity in both cases is low. Furthermore, given that the difference in ΔG$^{\pm}$ for the cycloaddition step is expected to be much lower that the difference in reaction energies (i.e. much less than 2.9 kJmol^{-1}), it seems reasonable that the slight bias in the pre-equilibrium step is sufficient to render the reaction stereoselective.

Perhaps unsurprisingly, given the small energy differences calculated, examining the models obtained does not give any significant clues as to the origin of different stabilities of the intermediates beyond the observation that all of the structures are highly sterically hindered (see space filling models in Figures S107-110) and that, thanks to the nature of the catenane architecture, this steric hindrance is expressed throughout the contact points between the two rings. The most noteworthy feature is that in all cases the large aromatic substituent of the auxiliary is projected away from the rest of the molecule.

In the case of (*R*,*R*)-**Ib** this appears to force the encircling chain (orange) of the alkyne/azide pre-macrocycle to partially straddle an aromatic ring (green) of the bipyridine macrocycle whereas in all of the other calculated structures the pre-macrocycle chain sits below the aromatic ring and mainly interacts with the alkyl region of the macrocycle. This may help account for the slight predicted destabilization of (*R*,*R*)-**Ib** vs (*R*,*S*)-**Ib** but this is clearly speculative.

It is also worth noting that in both triazolide intermediates, a hydrogen bond between the N^3 of the triazolide and the hydroxyl group is predicted. This interaction creates a cycle that includes the fixed stereogenic centre of the auxiliary and might be expected to enhance the transfer of chiral information and thus aid diastereoselection. However, given that this interaction is not presented in the predicted lowest energy conformations of the acetylide precursor (the distance N•••H-O is too great), it raises the question as to whether intermediates **Ib** and **IIb** are directly connected by a single transition state (i.e. perhaps the initially formed triazolide lacks this interaction and then undergoes conformational

rearrangement to take advantage of this stabilising contact). The favourability of this interaction is also likely to be modulated by competition with solvent, particularly relevant given the reaction is carried out in an EtOH co-solvent. Finally, it is worth noting that this H-bonding interaction is absent in the solid state structure of (R^*,S^*_{mt})-**3b**, where the OH is engaged in an H-bond with the sp^3 nitrogen instead, suggesting multiple H-bonded conformations/co-conformations are accessible.

Thus, although the calculations presented are consistent with the observed stereoselectivity, much more detailed studies are required to give a true explanation of the observed behaviour. In particular, a transition state must be found that connects the acetylide and triazolide intermediates and, given the potential role direct interactions with solvent may play, it is likely that explicit solvent will be required to gain an accurate representation of the process. These calculations lie outside the scope of this report, but we are currently working on generating predictive models that can then be tested and refined experimentally and the results of this study will be reported in due course.

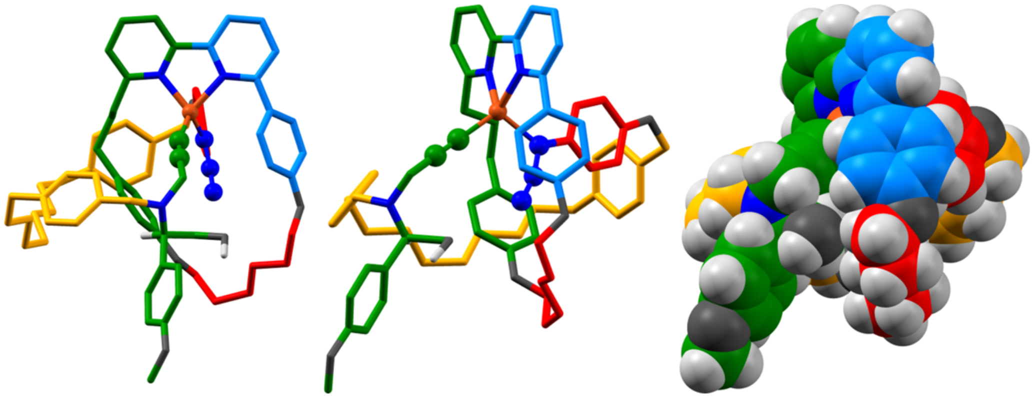

Figure S107. DFT (B3LYP, 631G, gas phase) model of intermediate (*R*,*R*)-**Ia** viewed from the front and side in capped sticks representation, and from the side in space-filling representation.

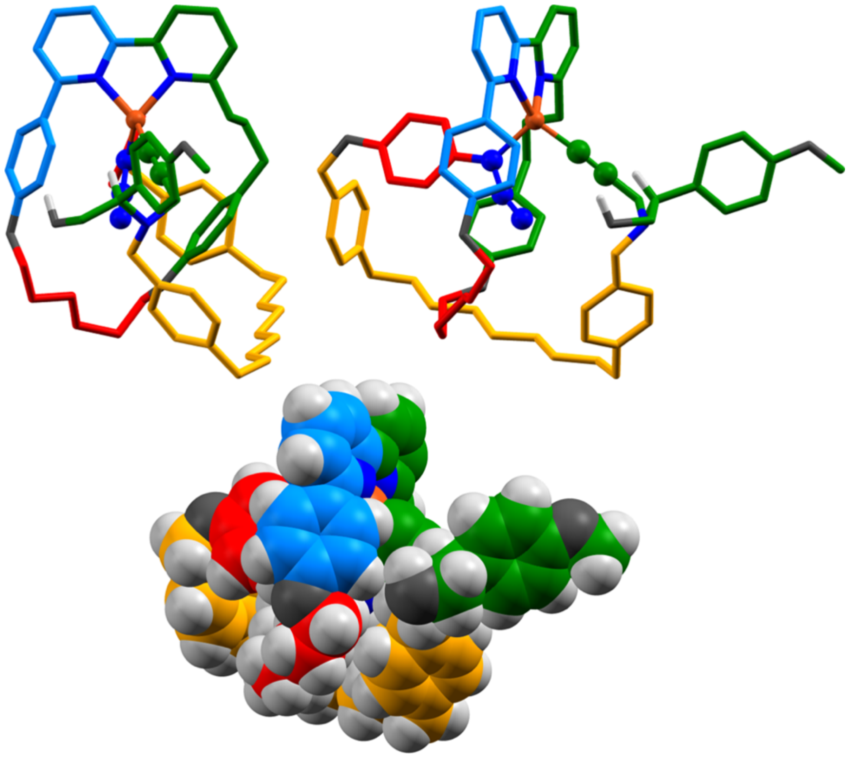

Figure S108. DFT (B3LYP, 631G, gas phase) model of intermediate (*R*,*S*)-**Ia** viewed from the front and side in capped sticks representation, and from the side in space-filling representation.

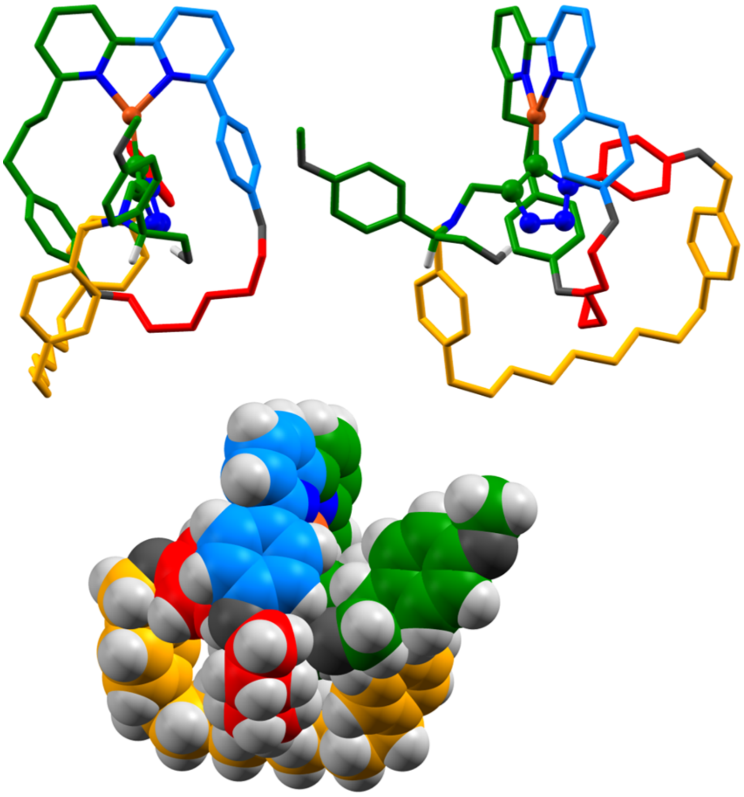

Figure S109. DFT (B3LYP, 631G, gas phase) model of intermediate (R,S_{mt})-**IIb** viewed from the front and side in capped sticks representation, and from the side in space-filling representation.

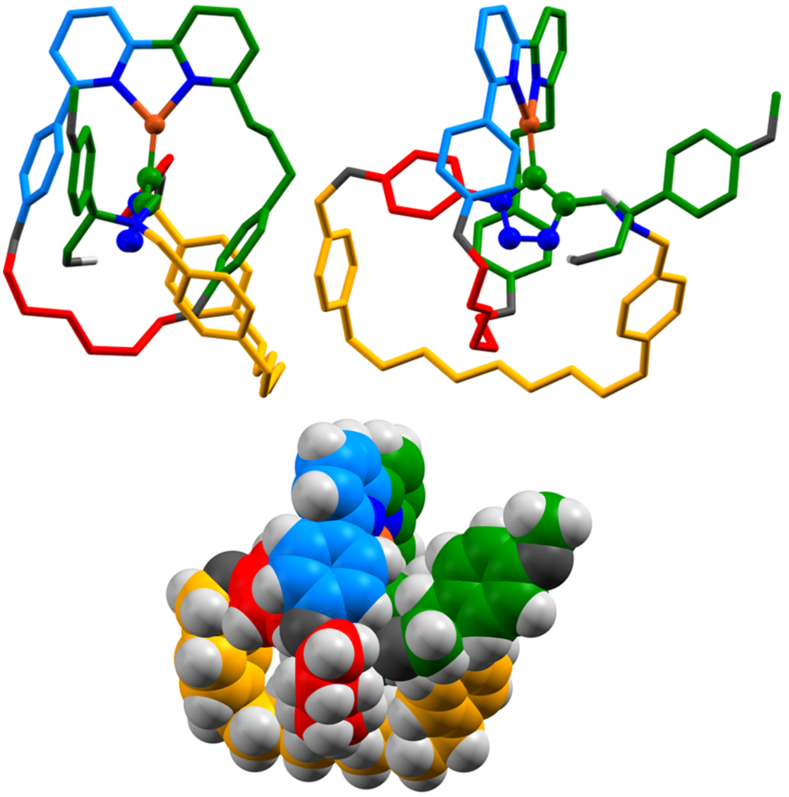

Figure S110. DFT (B3LYP, 631G, gas phase) model of intermediate (R,R_{mt})-**IIb** viewed from the front and side in capped sticks representation, and from the side in space-filling representation.

9. Supplemental References

1. Lewis, J.E.M., Bordoli, R.J., Denis, M., Fletcher, C.J., Galli, M., Neal, E.A., Rochette, E.M. and Goldup, S.M. (2016). High yielding synthesis of 2,2′-bipyridine macrocycles, versatile intermediates in the synthesis of rotaxanes. *Chem. Sci.* **7**, 3154–3161.

2. Ravi Kumar, A., Bhaskar, G., Madhan, A. and Venkateswara Rao, B. (2003). Stereoselective Synthesis of (−)-Cytoxazone and (+)-5-Epi-cytoxazone. *Synth. Commun.* **33**, 2907–2916.

3. Lewis, J.E.M., Modicom, F. and Goldup, S.M. (2018). Efficient Multicomponent Active Template Synthesis of Catenanes. *J. Am. Chem. Soc.* **140**, 4787–4791.

4. Neal, E.A. and Goldup, S.M. (2016). A Kinetic Self-Sorting Approach to Heterocircuit [3]Rotaxanes. *Angew. Chem., Int. Ed.* **55**, 12488–12493,(2015). Competitive formation of homocircuit [3]rotaxanes in synthetically useful yields in the bipyridine-mediated active template CuAAC reaction. *Chem. Sci.* **6**, 2398–2404.

5. Please note: for convenience, diastereomeric intermediates leading to (R,R_{mt})-**3b** and (S,R_{mt})-**3b** were modelled as these can be obtained by manually inverting the covalent stereocenter. Given that the intermediates *en route* to (S,R_{mt})-**3b** are enantiomeric to those leading to (R,S_{mt})-**3b** (i.e. have identical properties) the results presented here are valid for the (*R*,*S*/*R*) manifold. The coordinates of the modelled structures are available as supporting information for the manuscript (Data File 2).

6. M. J. Frisch, G. W. Trucks, H. B. Schlegel, G. E. Scuseria, M. A. Robb, J. R. Cheeseman, G. Scalmani, V. Barone, B. Mennucci, G. A. Petersson, H. Nakatsuji, M. Caricato, X. Li, H. P. Hratchian, A. F. Izmaylov, J. Bloino, G. Zheng, J. L. Sonnenberg, M. Hada, M. Ehara, K. Toyota, R. Fukuda, J. Hasegawa, M. Ishida, T. Nakajima, Y. Honda, O. Kitao, H. Nakai, T. Vreven, J. A. Montgomery, Jr., J. E. Peralta, F. Ogliaro, M. Bearpark, J. J. Heyd, E. Brothers, K. N. Kudin, V. N. Staroverov, R. Kobayashi, J. Normand, K. Raghavachari, A. Rendell, J. C. Burant, S. S. Iyengar, J. Tomasi, M. Cossi, N. Rega, J. M. Millam, M. Klene, J. E. Knox, J. B. Cross, V. Bakken, C. Adamo, J. Jaramillo, R. Gomperts, R. E. Stratmann, O. Yazyev, A. J. Austin, R. Cammi, C. Pomelli, J. W. Ochterski, R. L. Martin, K. Morokuma, V. G. Zakrzewski, G. A. Voth, P. Salvador, J. J. Dannenberg, S. Dapprich, A. D. Daniels, Ö. Farkas, J. B. Foresman, J. V. Ortiz, J. Cioslowski, and D. J. Fox, Gaussian 09 (Gaussian, Inc., Wallingford CT, 2009).

6. Sheldrick, G. M. (2015). Crystal structure refinement with SHELXL. *Acta. Cryst*. **C71**, 3-8.

7. Dolomanov, O. V., Bourhis, J. L., Gildea, J., Howard, J. A. K., and Pushmann, H. (2009). OLEX2: a complete structure solution, refinement and analysis program. *Appl. Crystallogr*. **42**, 339-341.
